# Supplementary material for: Rational design of minimal synthetic promoters for plants
Source: Nucleic Acids Res. 2020 Aug 28;48(21):11845–56. doi: 10.1093/nar/gkaa682 (PMC7708054; doi:10.1093/nar/gkaa682)

Supplementary Data 1. Table of all Minimal Synthetic Promoters (MinSyns) tested in this study. Sequence-variants of cis-regulatory elements (CREs) predicted to bind the same class of transcription factors (TFs) are identified by a subscript numerator (e.g. 3 x bHLH$_1$ and 3 x bHLH$_2$). Some CREs consist of multiple, overlapping TF binding sites indicated by a "+" followed by a list of all TFs predicted to bind to that CRE in parentheses (e.g. C2C2dof+$_2$(C2C2dof+MADS+Orphan+REM). Multiple CREs within in the same MinSyn are separated by "/". Asterisks (*) indicate that the MinSyn sequence is spilt into multiple Level 0 plasmids (e.g. distal, proximal and core regions), which are co-assembled into the expression construct.

Name	CREs in Var region	Description	Length (bp)	Link to sequence	Plasmid Code (Level 0 Phytobrick)	Plasmid Code (Expression cassette)
MinSyn_000	NONE	Control MinSyn (no CREs in variable region)	93	https://benchling.com/s/seq-0qsQE70PiNhTiNi5bHiB	pEPYC0CM0035	pEPYC1CB0007
MinSyn_001	3 x bHLH$_1$	three copies of a single CRE	107	https://benchling.com/s/seq-wZFNbyhVnAZN2L4t976N	pEPYC0CM0366	pEPYC1CB0426
MinSyn_002	3 x bZIP	three copies of a single CRE	116	https://benchling.com/s/seq-1EFNTdKRRpjTkhYw2sDh	pEPYC0CM0367	pEPYC1CB0427
MinSyn_003	3 x AP2+$_1$ (TCP)	three copies of a single CRE	150	https://benchling.com/s/seq-FgX1DQI9OQ9jr6nHEfuo	pEPYC0CM0368	pEPYC1CB0428
MinSyn_004	3 x C2C2dof+$_2$ (C2C2dof+MADS+Orphan+REM)	three copies of a single CRE	161	https://benchling.com/s/seq-Ie8CjiZ1fgHjECHX2DEb	pEPYC0CM0379	pEPYC1CB0439
MinSyn_005	3 x TCP$_2$	three copies of a single CRE	113	https://benchling.com/s/seq-k6tmMv8g91T31lMmRcyu	pEPYC0CM0380	pEPYC1CB0440
MinSyn_006	3 x MYB+ (MYB+c2c2gata+C3H)	three copies of a single CRE	167	https://benchling.com/s/seq-YiWhJ0E1fSpbn0D6zcfl	pEPYC0CM0381	pEPYC1CB0441
MinSyn_007	3 x C2H2+ (C2H2+C3H+GeBP)	three copies of a single CRE	104	https://benchling.com/s/seq-CqGe4PaJqf5EVxmf63YM	pEPYC0CM0372	pEPYC1CB0432
MinSyn_008	3 x C2C2dof+$_1$ (C2C2dof+TCP+NAC)	three copies of a single CRE	119	https://benchling.com/s/seq-Gu0h6lV0fgko9wq4Ooic	pEPYC0CM0373	pEPYC1CB0433
MinSyn_009	3 x RWPRK$_1$	three copies of a single CRE	119	https://benchling.com/s/seq-4DiYhtKMS4jTrO9z8O4t	pEPYC0CM0374	pEPYC1CB0434
MinSyn_010	3 x bHLH$_2$	three copies of a single CRE	113	https://benchling.com/s/seq-F3OqlBKFww0p5Ue40ZF1	pEPYC0CM0385	pEPYC1CB0445
MinSyn_011	3 x bHLH+	three copies of a single CRE	158	https://benchling.com/s/seq-Lf2OvoC3FHuWrlDtdO3z	pEPYC0CM0386	pEPYC1CB0446
MinSyn_012	3 x WRKY+ (C2C2dof+WRKY+ABI3VP1+RWPRK+CP P+C2H2+Orphan+REM+ARID)	three copies of a single CRE	182	https://benchling.com/s/seq-PSxlZv6z9gCLbq50TCnl	pEPYC0CM0387	pEPYC1CB0447
MinSyn_013	3 x C2H2	three copies of a single CRE	107	https://benchling.com/s/seq-9Qmk9BuaKbdFtHq2BtW6	pEPYC0CM0390	pEPYC1CB0450
MinSyn_014	3 x Homeobox	three copies of a single CRE	113	https://benchling.com/s/seq-wHkZrqd5FzLVbSi82twd	pEPYC0CM0391	pEPYC1CB0451
MinSyn_015	3 x G2like+ (G2like+ABI3VP1+NAC)	three copies of a single CRE	116	https://benchling.com/s/seq-de11VZwrJhuE9NySaMs7	pEPYC0CM0392	pEPYC1CB0452
MinSyn_016	3 x RWPRK$_2$	three copies of a single CRE	131	https://benchling.com/s/seq-OGYnTcMdmkvb2WEWlhSq	pEPYC0CM0375	pEPYC1CB0435
MinSyn_017	3 x WRKY	three copies of a single CRE	122	https://benchling.com/s/seq-Ptw5qtT6l7qAcuVQWg7q	pEPYC0CM0376	pEPYC1CB0436
MinSyn_018	3 x DBP	three copies of a single CRE	119	https://benchling.com/s/seq-PSPzNNkut5USu9SK6J0O	pEPYC0CM0377	pEPYC1CB0437
MinSyn_019	3 x MADS+$_2$ (MADS+C2C2gata)	three copies of a single CRE	146	https://benchling.com/s/seq-B9obPiQwht5tHuhS89yN	pEPYC0CM0382	pEPYC1CB0442
MinSyn_020	3 x TCP$_3$	three copies of a single CRE	119	https://benchling.com/s/seq-V34zUQX5ySxNzGN0qLOb	pEPYC0CM0383	pEPYC1CB0443
MinSyn_021	3 x G2like	three copies of a single CRE	113	https://benchling.com/s/seq-N6u8ZjGf94wXLTeNUbyn	pEPYC0CM0384	pEPYC1CB0444
MinSyn_022	3 x AP2+ (AP2+LOBAS2)	three copies of a single CRE	152	https://benchling.com/s/seq-IbRApeJR28SUd6pQg99w	pEPYC0CM0393	pEPYC1CB0453
MinSyn_023	3 x C-CRE	three copies of a single CRE	140	https://benchling.com/s/seq-PH818sWZDkwfe5LkP1MS	pEPYC0CM0365	pEPYC1CB0425
MinSyn_024	bHLH$_1$/bZIP/AP2+$_1$	three different CREs	122	https://benchling.com/s/seq-Pdin3HP2AviGfrRDnB60	pEPYC0CM0431	pEPYC1CB0493
MinSyn_026	C2C2dof+$_2$/TCP$_2$/MYB+	three different CREs	144	https://benchling.com/s/seq-HT8QBHITlc0aUofyzNtH	pEPYC0CM0435	pEPYC1CB0497
MinSyn_027	C2H2+/C2C2dof+$_1$/RWPRK$_1$	three different CREs	111	https://benchling.com/s/seq-SUOKjZKcdhbo042o130i	pEPYC0CM0433	pEPYC1CB0495
MinSyn_028	bHLH$_2$/bHLH+/WRKY+	three different CREs	148	https://benchling.com/s/seq-3JYJRKIjujcE0QF8fe36	pEPYC0CM0437	pEPYC1CB0499
MinSyn_029	C2H2/Homeobox/G2like+	three different CREs	109	https://benchling.com/s/seq-0tR8wRGTga3dWw2ojdUw	pEPYC0CM0439	pEPYC1CB0501
MinSyn_030	RWPRK$_2$/WRKY+/DBP	three different CREs	121	https://benchling.com/s/seq-QzeRW1REYLnxpLKTQ8K5	pEPYC0CM0434	pEPYC1CB0496
MinSyn_031	MADS+$_2$/TCP$_3$/G2like	three different CREs	149	https://benchling.com/s/seq-cjnH9arWrL87HofRr8p2	pEPYC0CM0436	pEPYC1CB0498
MinSyn_032	Homeobox/G2like$_2$/AP2+$_2$	three different CREs	122	https://benchling.com/s/seq-qOrS1fNh1I3sknYW7YZE	pEPYC0CM0440	pEPYC1CB0502
MinSyn_033	1 x C-CRE	single copy of the C-CRE	96	https://benchling.com/s/seq-e1nuSZ2sZzYsudP557Jh	pEPYC0CM0041	pEPYC1CB0079
MinSyn_034	AP2+$_1$/bZIP/bHLH$_1$/C-CRE	three different CREs + C-CRE	199	https://benchling.com/s/seq-esQkM6GttTF3qfAQAQND	pEPYC0CM0310	pEPYC1CB0352
MinSyn_035	AP2+$_1$/bZIP/bHLH$_1$/C-CRE	25 bp between CREs and TATA	224	https://benchling.com/s/seq-AvdnsDcbLsTGoydoVsOf	pEPYC0CM0312	pEPYC1CB0354
MinSyn_036	AP2+$_1$/bZIP/bHLH$_1$/C-CRE	50 bp between CREs and TATA	249	https://benchling.com/s/seq-i6vT6WeKtnr5SauKJDR2	pEPYC0CM0313	pEPYC1CB0355
MinSyn_037	AP2+$_1$/bZIP/bHLH$_1$/C-CRE	75 bp between CREs and TATA	274	https://benchling.com/s/seq-gskfrYVYdC3Fi8vEOL8z	pEPYC0CM0314	pEPYC1CB0356
MinSyn_038	MYB+/C2C2dof+$_2$/TCP$_2$/C-CRE	three different CREs + C-CRE	232	https://benchling.com/s/seq-apzE3SCGRK74bHdhJlvS	pEPYC0CM0225	pEPYC1CB0269
MinSyn_039	MYB+/C2C2dof+$_2$/TCP$_2$/C-CRE	25 bp between CREs and TATA	241	https://benchling.com/s/seq-Eu3N4i4XhQDJbSHC3Ovb	pEPYC0CM0209	pEPYC1CB0253
MinSyn_040	MYB+/C2C2dof+$_2$/TCP$_2$/C-CRE	50 bp between CREs and TATA	266	https://benchling.com/s/seq-3ghoZvi65XsWYie06FCb	pEPYC0CM0210	pEPYC1CB0254
MinSyn_041	MYB+/C2C2dof+$_2$/TCP$_2$/C-CRE	75 bp between CREs and TATA	291	https://benchling.com/s/seq-LGRCpftG1qJMzZj67PFb	pEPYC0CM0211	pEPYC1CB0255
MinSyn_042	C-CRE/Homeobox/C2H2	two different CREs + C-CRE	158	https://benchling.com/s/seq-Mjgku0iWBVrSI1v132qv	pEPYC0CM0311	pEPYC1CB0353
MinSyn_043	C-CRE/Homeobox/C2H2	25 bp between CREs and TATA	182	https://benchling.com/s/seq-llYzHiCBO6XzLOE1zowj	pEPYC0CM0212	pEPYC1CB0256
MinSyn_044	C-CRE/Homeobox/C2H2	50 bp between CREs and TATA	207	https://benchling.com/s/seq-qu6hTPHmly538zhUWBUe	pEPYC0CM0213	pEPYC1CB0257
MinSyn_045	C-CRE/Homeobox/C2H2	75 bp between CREs and TATA	232	https://benchling.com/s/seq-apzE3SCGRK74bHdhJlvS	pEPYC0CM0214	pEPYC1CB0258
MinSyn_046	AP2+$_1$/bZIP/bHLH$_1$/C-CRE	three different CREs + C-CRE	147	https://benchling.com/s/seq-TBv49IqInULaRzZrA6z7	pEPYC0CM0182	pEPYC1CB0187
MinSyn_048	AP2+$_1$/bZIP/bHLH$_1$/C-CRE	Random flanking sequence between CREs	203	https://benchling.com/s/seq-m70LnNmMxibPARIVXdTq	pEPYC0CM0395	pEPYC1CB0455
MinSyn_049	AP2+$_1$/bZIP/bHLH$_1$/C-CRE	Random flanking sequence between CREs	203	https://benchling.com/s/seq-hKF7cEnrzRI7ZvmT8TQH	pEPYC0CM0396	pEPYC1CB0456
MinSyn_050	bZIP/C-CRE/bHLH$_1$/AP2+$_1$	three different CREs + C-CRE	143	https://benchling.com/s/seq-JYXCbEXuLujUG4vRS6Sa	pEPYC0CM0451	pEPYC1CB0509
MinSyn_051	bHLH$_1$/AP2+$_1$/C-CRE/bZIP	three different CREs + C-CRE	143	https://benchling.com/s/seq-4ysOthO8IwMi4Gza4KvA	pEPYC0CM0452	pEPYC1CB0510
MinSyn_052	MYB+/C2C2dof+$_2$/TCP$_2$/C-CRE	three different CREs + C-CRE	169	https://benchling.com/s/seq-84vUPFSvAkNf4HlawIf2	pEPYC0CM0183	pEPYC1CB0188
MinSyn_054	MYB+/C2C2dof+$_2$/TCP$_2$/C-CRE	Random flanking sequence between CREs	226	https://benchling.com/s/seq-16dbTNGncrGBQ98PC2fq	pEPYC0CM0397	pEPYC1CB0457
MinSyn_055	MYB+/C2C2dof+$_2$/TCP$_2$/C-CRE	Random flanking sequence between CREs	226	https://benchling.com/s/seq-SLE1so0KdCvy4Ceg2GoO	pEPYC0CM0398	pEPYC1CB0458
MinSyn_056	TCP$_2$/MYB+/C-CRE/C2C2dof+$_2$	three different CREs + C-CRE	166	https://benchling.com/s/seq-p5YhEx6oyxBxS2jEiHpq	pEPYC0CM0453	pEPYC1CB0511
MinSyn_057	C2C2dof+$_2$/TCP$_2$/MYB+/C-CRE	three different CREs + C-CRE	166	https://benchling.com/s/seq-cTjP9K65UEBrXAVDv6pp	pEPYC0CM0454	pEPYC1CB0512
MinSyn_058	C-CRE/Homeobox/C2H2	two different CREs + C-CRE	120	https://benchling.com/s/seq-QSCATVwH0gJ3UiGp2JCX	pEPYC0CM0184	pEPYC1CB0189
MinSyn_060	C-CRE/Homeobox/C2H2	Random flanking sequence between CREs	159	https://benchling.com/s/seq-mGTHb9piclAMIRJRPBpW	pEPYC0CM0401	pEPYC1CB0461
MinSyn_061	C-CRE/Homeobox/C2H2	Random flanking sequence between CREs	159	https://benchling.com/s/seq-ojbsHEj0CNtVv71B8JPE	pEPYC0CM0402	pEPYC1CB0462
MinSyn_062	C2H2/Homeobox/C-CRE	two different CREs + C-CRE	117	https://benchling.com/s/seq-i7GRR4HRQxNjR00gv1Ty	pEPYC0CM0455	pEPYC1CB0513
MinSyn_063	Homeobox/C-CRE/C2H2	two different CREs + C-CRE	117	https://benchling.com/s/seq-qyco6lU97tfWXfMmb9Gp	pEPYC0CM0456	pEPYC1CB0514
MinSyn_064	1 x TALE AvrXa27 binding site	Binding site for synthetic TF	88	https://benchling.com/s/seq-MkgA2YoAajmr7xhfPyBJ	pEPAS0CMR0015	pEPKKα2KN0091/ pEPYCα2rKN0010
MinSyn_065	2 x TALE AvrXa27 binding sites	Binding site for synthetic TF	126	https://benchling.com/s/seq-slokOMWiDRAxfmqqNsjN	pEPAS0CMR0016	pEPKKα2KN0092
MinSyn_066	4 x TALE AvrXa27 binding sites	Binding site for synthetic TF	202	https://benchling.com/s/seq-gDZylOe6miWzsyrgor0n	pEPKK0CM0107	pEPKKα2KN0093/ pEPYCα2rKN0009/ pEPYCα1KN0008
MinSyn_067	2 x ϕC3 binding sites	Binding site for synthetic TF	242	https://benchling.com/s/seq-fgzjQewCH5vr3GfCEkiG	pEPKK0CM0065	pEPKKα2KN0100
MinSyn_068	4 x ϕC3 binding sites	Binding site for synthetic TF	360	https://benchling.com/s/seq-t0a3QQRnQ7uywEow0mdL https://benchling.com/s/seq-JioHeeBtqPHTH6h1YVs4	pEPKK0CM0062 + pEPKK0CM0111*	pEPKKα2KN0101

Name	CREs in Var region	Description	Length (bp)	Link to sequence	Plasmid Code (Level 0 Phytobrick)	Plasmid Code (Expression cassette)
MinSyn_069	6 x ϕC3 binding sites	Binding site for synthetic TF	478	https://benchling.com/s/seq-NpXSAnIIWwDqevLWFfSt<br>https://benchling.com/s/seq-aoVmB0iw0bGZJpfb4zu4<br>https://benchling.com/s/seq-JioHeeBtqPHTH6h1YVs4	pEPKK0cm0063 + pEPKK0CM0064 + pEPKK0CM0111*	pEPKKα2KN0102/ pEPYCα1KN0003
MinSyn_101	bHLH$_2$/bHLH$_1$/AP2+$_1$/Homeobox/WRKY	computationally designed	274	https://benchling.com/s/seq-4DGAEuNIAqGYKpgxFpuE	pEPYC0CM0407	pEPYC1CB0467
MinSyn_102	C2C2dof+/AP2+$_1$/WRKY	computationally designed	208	https://benchling.com/s/seq-8fiNn13Fn41xS0R1NwAa	pEPYC0CM0408	pEPYC1CB0468
MinSyn_103	bHLH$_1$/MYB+	computationally designed	149	https://benchling.com/s/seq-viwbvfSENd3wupQtCuh8	pEPYC0CM0409	pEPYC1CB0469
MinSyn_104	RWPRK$_1$/CCAAT+/HSF+/MADS+/AP2+$_2$/G2like/DBP/C-CRE/MYB+	computationally designed	417	https://benchling.com/s/seq-UkP4FqWF5RxWyr0d4wNf	pEPYC0CM0410	pEPYC1CB0470
MinSyn_105	bZIP/C-CRE/Homeobox/MYB+/C-CRE	computationally designed	272	https://benchling.com/s/seq-OeJ5B32kVlqfCwcM449w	pEPYC0CM0411	pEPYC1CB0471
MinSyn_106	C-CRE/AP2+$_1$/HSF+/C2C2dof+$_2$/CCAAT+/C-CRE	computationally designed	330	https://benchling.com/s/seq-MzLsjqJF4Wk33AQyMV5N	pEPYC0CM0412	pEPYC1CB0472
MinSyn_107	RWPRK$_2$/C-CRE/WRKY/G2like+/C-CRE	computationally designed	247	https://benchling.com/s/seq-kbeGUgNxVrqNfb80nFY2	pEPYC0CM0413	pEPYC1CB0473
MinSyn_108	bHLH$_1$/C2H2/AP2+$_2$/AP2+$_1$/Homeobox/C-CRE	computationally designed	272	https://benchling.com/s/seq-PLJyq4WT8G03wG3gQY8o	pEPYC0CM0414	pEPYC1CB0474/pEPYCα1KN0002/pEPYCα1KN0007
MinSyn_109	bHLH$_1$/C-CRE/MYB+/C-CRE	computationally designed	224	https://benchling.com/s/seq-vH4V5K7vNg2X7tWDlGy4	pEPYC0CM0415	pEPYC1CB0475
MinSyn_110	TCP$_2$/RWPRK$_1$/bHLH$_1$/MYB+/C-CRE/C-CRE	computationally designed	285	https://benchling.com/s/seq-sX2AR6aULzb2lyuoMLYX	pEPYC0CM0416	pEPYC1CB0476
MinSyn_111	MADS+$_1$/MYB+/C-CRE/WRKY+/MADS+$_2$/C2H2+	computationally designed	261	https://benchling.com/s/seq-pBG4wmcNc3SwSlXKuhWS	pEPYC0CM0417	pEPYC1CB0479
MinSyn_112	TCP$_3$/C2H2+/Homeobox	computationally designed	143	https://benchling.com/s/seq-VkwMb37FrE9KbWSkpxI5	pEPYC0CM0418	pEPYC1CB0480
MinSyn_113	C-CRE/Homeobox/AP2+$_2$/MADS+$_1$/WRKY/bHLH$_2$	computationally designed	258	https://benchling.com/s/seq-odcECHuFtf3378P8NuD1	pEPYC0CM0419	pEPYC1CB0481
MinSyn_114	C-CRE/C2H2+/G2like/MADS+$_1$/AP2+$_2$/bHLH$_1$/RWPRK$_2$	computationally designed	243	https://benchling.com/s/seq-1plZVxeHhgxrTP8IH7Fz	pEPYC0CM0420	pEPYC1CB0482
MinSyn_115	CCAAT+/C2H2+/DBP	computationally designed	161	https://benchling.com/s/seq-gtv2NB02AKAKqMo9z4M3	pEPYC0CM0421	pEPYC1CB0483
MinSyn_116	RWPRK$_2$/bHLH+/C-CRE/TCP$_1$/bHLH$_2$/bZIP/bHLH$_1$	computationally designed	262	https://benchling.com/s/seq-NTaiQg01dsWOX8fZ2nik	pEPYC0CM0422	pEPYC1CB0484
MinSyn_117	MADS+$_1$/bHLH$_1$/C2H2+/bHLH+/bZIP/WRKY/G2like+/TCP$_3$/AP2+$_1$	computationally designed	315	https://benchling.com/s/seq-LPnjtxz4pTd5CIrc5IAC	pEPYC0CM0423	pEPYC1CB0485
MinSyn_118	Homeobox/C-CRE	computationally designed	137	https://benchling.com/s/seq-917cevKHSyFgOWBg3jra	pEPYC0CM0424	pEPYC1CB0486
MinSyn_119	TCP$_2$/TCP$_3$/C2H2+/AP2+$_2$/DBP/C2H2/C-CRE	computationally designed	245	https://benchling.com/s/seq-1WZttWPPAJjzSQqTEmkS	pEPYC0CM0425	pEPYC1CB0487
MinSyn_120	C-CRE/HSF+	computationally designed	118	https://benchling.com/s/seq-0ngHrUjGdIIUWOUsjwBz	pEPYC0CM0426	pEPYC1CB0488
MinSyn_121	MADS+$_1$/C2C2dof+$_1$/bHLH+/TCP$_1$/MADS+$_2$/AP2+$_2$/DBP/C2H2/AP2+$_1$	computationally designed	329	https://benchling.com/s/seq-HFTwco2h8HqVVgcKtsoT	pEPYC0CM0427	pEPYC1CB0489
MinSyn_122	C2H2+/Homeobox/AP2+$_2$/AP2+$_1$/RWPRK$_2$	computationally designed	187	https://benchling.com/s/seq-bnSRl1g6bO8GMnlJlThr	pEPYC0CM0428	pEPYC1CB0490
MinSyn_123	WRKY+/Homeobox/RWPRK$_2$/CCAAT+/C2C2dof+/G2like+/DBP/C-CRE/bZIP	computationally designed	354	https://benchling.com/s/seq-n1wTXpDjDpO1EEmi2CES	pEPYC0CM0429	pEPYC1CB0491
MinSyn_124	MYB+/MADS+$_2$/AP2+$_2$/AP2+$_1$/C-CRE	computationally designed	214	https://benchling.com/s/seq-7EzWHcgHI3lvJTQBBtuB	pEPYC0CM0430	pEPYC1CB0492
MinSyn_285	bZIP/Homeobox/C2C2dof+$_2$/C-CRE	computationally designed	209	https://benchling.com/s/seq-EouughPWqfDqXnyTEFR9	pEPYC0CM0244	pEPYC1CB0286

Supplementary Data 2 List of Plasmids

1. Level 0 Phytobricks

Addgene #	Plasmid code	Part type	Description/Origin	Compatibility with Assembly Systems	Cloning overhang (top strand)		Source of plasmid
					5'	3'	
68187	GB0036	3UTR+TERM	35s (Cauliflower Mosaic Virus)	MoClo, Loop, GB	GCTT	CGCT	Vazquez-Vilar et al (2017)
-	GB0552	PROM+5UTR	35s (Cauliflower Mosaic Virus)	GB, Loop	GGAG	CCAT	Vazquez-Vilar et al (2017)
154477	pEPYC0CM0071	PROM	35s (Cauliflower Mosaic Virus)	MoClo, Loop, GB	GGAG	TACT	This study
50337	pICH41414	3UTR+TERM	35s (Cauliflower Mosaic Virus)	MoClo, Loop, GB	GCTT	CGCT	Engler et al (2014)
154598	pEPYC0CM0168	PROM	35s (ΔAP2+$_1$)	MoClo, Loop, GB	GGAG	TACT	This study
154599	pEPYC0CM0170	PROM	35s(ΔbHLH$_1$)	MoClo, Loop, GB	GGAG	TACT	This study
154600	pEPYC0CM0169	PROM	35s(ΔbZIP)	MoClo, Loop, GB	GGAG	TACT	This study
154601	pEPYC0CM0171	PROM	35s(ΔC_CRE)	MoClo, Loop, GB	GGAG	TACT	This study
154485	pEPYC0CM0281	PROM	35s(ΔC2C2dof+$_1$)	MoClo, Loop, GB	GGAG	TACT	This study
154486	pEPYC0CM0280	PROM	35s(ΔC2H2+)	MoClo, Loop, GB	GGAG	TACT	This study
154487	pEPYC0CM0285	PROM	35s(ΔDBP)	MoClo, Loop, GB	GGAG	TACT	This study
154488	pEPYC0CM0279	PROM	35s(ΔHSF+)	MoClo, Loop, GB	GGAG	TACT	This study
154489	pEPYC0CM0277	PROM	35s(ΔMADS+$_1$)	MoClo, Loop, GB	GGAG	TACT	This study
154490	pEPYC0CM0282	PROM	35s(ΔRWPRK$_1$)	MoClo, Loop, GB	GGAG	TACT	This study
154491	pEPYC0CM0283	PROM	35s(ΔRWPRK$_2$)	MoClo, Loop, GB	GGAG	TACT	This study
154492	pEPYC0CM0278	PROM	35s(ΔTCP$_1$)	MoClo, Loop, GB	GGAG	TACT	This study
154493	pEPYC0CM0284	PROM	35s(ΔWRKY)	MoClo, Loop, GB	GGAG	TACT	This study
154479	pEPYC0CM0084	PROM	CaMV35S (C-CRE relocated)	MoClo, Loop, GB	GGAG	TACT	This study
154480	pEPYC0CM0089	PROM	CaMV35S (C-CRE relocated)	MoClo, Loop, GB	GGAG	TACT	This study
154597	pICSL20002	5UTR	CPMV (CowPea Mosaic Virus)	MoClo, Loop, GB	TACT	CCAT	This study
50270	pICSL12006	PROM+5UTR	CsVMV (Cassava Vein Mosaic Virus)	MoClo, Loop, GB	GGAG	AATG	Engler et al (2014)
50308	pICSL50007	CTAG	FLAG tag	MoClo, Loop, GB	TTCG	GCTT	Engler et al (2014)
50338	pICH72400	3UTR+TERM	g7 (Agrobacterium tumefaciens)	MoClo, Loop, GB	GCTT	CGCT	Engler et al (2014)
-	GB0900	NTAG	GAL4 activation domain	MoClo, Loop, GB	CCAT	AATG	Vazquez-Vilar et al (2017)
154593	pEPYC0CM0258	NTAG	HiBit	MoClo, Loop, GB	CCAT	AATG	This study
154594	pEPAS0CM0008	CDS	LucF	MoClo, Loop, GB	AATG	TTCG	This study
154595	pEPYC0CM0133	CDS	LucN	MoClo, Loop, GB	AATG	TTCG	This study
50272	pICH85281	PROM	MAS (Agrobacterium tumefaciens)	MoClo, Loop, GB	GGAG	AATG	Engler et al (2014)
154503	pEPYC0CM0035	PROM	MinSyn_000	MoClo, Loop, GB	GGAG	TACT	This study
154504	pEPYC0CM0366	PROM	MinSyn_001	MoClo, Loop, GB	GGAG	TACT	This study
154505	pEPYC0CM0367	PROM	MinSyn_002	MoClo, Loop, GB	GGAG	TACT	This study
154506	pEPYC0CM0368	PROM	MinSyn_003	MoClo, Loop, GB	GGAG	TACT	This study
154507	pEPYC0CM0379	PROM	MinSyn_004	MoClo, Loop, GB	GGAG	TACT	This study
154508	pEPYC0CM0380	PROM	MinSyn_005	MoClo, Loop, GB	GGAG	TACT	This study
154509	pEPYC0CM0381	PROM	MinSyn_006	MoClo, Loop, GB	GGAG	TACT	This study
154510	pEPYC0CM0372	PROM	MinSyn_007	MoClo, Loop, GB	GGAG	TACT	This study
154511	pEPYC0CM0373	PROM	MinSyn_008	MoClo, Loop, GB	GGAG	TACT	This study
154512	pEPYC0CM0374	PROM	MinSyn_009	MoClo, Loop, GB	GGAG	TACT	This study
154513	pEPYC0CM0385	PROM	MinSyn_010	MoClo, Loop, GB	GGAG	TACT	This study
154514	pEPYC0CM0386	PROM	MinSyn_011	MoClo, Loop, GB	GGAG	TACT	This study
154515	pEPYC0CM0387	PROM	MinSyn_012	MoClo, Loop, GB	GGAG	TACT	This study
154516	pEPYC0CM0390	PROM	MinSyn_013	MoClo, Loop, GB	GGAG	TACT	This study
154517	pEPYC0CM0391	PROM	MinSyn_014	MoClo, Loop, GB	GGAG	TACT	This study
154518	pEPYC0CM0392	PROM	MinSyn_015	MoClo, Loop, GB	GGAG	TACT	This study
154519	pEPYC0CM0375	PROM	MinSyn_016	MoClo, Loop, GB	GGAG	TACT	This study
154520	pEPYC0CM0376	PROM	MinSyn_017	MoClo, Loop, GB	GGAG	TACT	This study
154521	pEPYC0CM0377	PROM	MinSyn_018	MoClo, Loop, GB	GGAG	TACT	This study
154522	pEPYC0CM0382	PROM	MinSyn_019	MoClo, Loop, GB	GGAG	TACT	This study
154523	pEPYC0CM0383	PROM	MinSyn_020	MoClo, Loop, GB	GGAG	TACT	This study
154524	pEPYC0CM0384	PROM	MinSyn_021	MoClo, Loop, GB	GGAG	TACT	This study
154525	pEPYC0CM0393	PROM	MinSyn_022	MoClo, Loop, GB	GGAG	TACT	This study
154526	pEPYC0CM0365	PROM	MinSyn_023	MoClo, Loop, GB	GGAG	TACT	This study
154527	pEPYC0CM0431	PROM	MinSyn_024	MoClo, Loop, GB	GGAG	TACT	This study
154528	pEPYC0CM0435	PROM	MinSyn_026	MoClo, Loop, GB	GGAG	TACT	This study
154529	pEPYC0CM0433	PROM	MinSyn_027	MoClo, Loop, GB	GGAG	TACT	This study
154530	pEPYC0CM0437	PROM	MinSyn_028	MoClo, Loop, GB	GGAG	TACT	This study
154531	pEPYC0CM0439	PROM	MinSyn_029	MoClo, Loop, GB	GGAG	TACT	This study
154532	pEPYC0CM0434	PROM	MinSyn_030	MoClo, Loop, GB	GGAG	TACT	This study
154533	pEPYC0CM0388	PROM	MinSyn_031	MoClo, Loop, GB	GGAG	TACT	This study
154534	pEPYC0CM0440	PROM	MinSyn_032	MoClo, Loop, GB	GGAG	TACT	This study
154535	pEPYC0CM0041	PROM	MinSyn_033	MoClo, Loop, GB	GGAG	TACT	This study
154536	pEPYC0CM0310	PROM	MinSyn_034	MoClo, Loop, GB	GGAG	TACT	This study
154537	pEPYC0CM0312	PROM	MinSyn_035	MoClo, Loop, GB	GGAG	TACT	This study
154538	pEPYC0CM0313	PROM	MinSyn_036	MoClo, Loop, GB	GGAG	TACT	This study
154539	pEPYC0CM0314	PROM	MinSyn_037	MoClo, Loop, GB	GGAG	TACT	This study
154540	pEPYC0CM0225	PROM	MinSyn_038	MoClo, Loop, GB	GGAG	TACT	This study
154541	pEPYC0CM0209	PROM	MinSyn_039	MoClo, Loop, GB	GGAG	TACT	This study
154542	pEPYC0CM0210	PROM	MinSyn_040	MoClo, Loop, GB	GGAG	TACT	This study
154543	pEPYC0CM0211	PROM	MinSyn_041	MoClo, Loop, GB	GGAG	TACT	This study
154544	pEPYC0CM0311	PROM	MinSyn_042	MoClo, Loop, GB	GGAG	TACT	This study
154545	pEPYC0CM0212	PROM	MinSyn_043	MoClo, Loop, GB	GGAG	TACT	This study
154546	pEPYC0CM0213	PROM	MinSyn_044	MoClo, Loop, GB	GGAG	TACT	This study
154547	pEPYC0CM0214	PROM	MinSyn_045	MoClo, Loop, GB	GGAG	TACT	This study
154609	pEPYC0CM0182	PROM	MinSyn_046	MoClo, Loop, GB	GGAG	TACT	This study
154548	pEPYC0CM0395	PROM	MinSyn_048	MoClo, Loop, GB	GGAG	TACT	This study
154549	pEPYC0CM0396	PROM	MinSyn_049	MoClo, Loop, GB	GGAG	TACT	This study
154550	pEPYC0CM0451	PROM	MinSyn_050	MoClo, Loop, GB	GGAG	TACT	This study
154551	pEPYC0CM0452	PROM	MinSyn_051	MoClo, Loop, GB	GGAG	TACT	This study
154610	pEPYC0CM0183	PROM	MinSyn_052	MoClo, Loop, GB	GGAG	TACT	This study
154552	pEPYC0CM0397	PROM	MinSyn_054	MoClo, Loop, GB	GGAG	TACT	This study
154553	pEPYC0CM0398	PROM	MinSyn_055	MoClo, Loop, GB	GGAG	TACT	This study

1. Level 0 Phytobricks

Addgene #	Plasmid code	Part type	Description/Origin	Compatibility with Assembly Systems	Cloning overhang (top strand) 5'	3'	Source of plasmid
154554	pEPYC0CM0453	PROM	MinSyn_056	MoClo, Loop, GB	GGAG	TACT	This study
154555	pEPYC0CM0454	PROM	MinSyn_057	MoClo, Loop, GB	GGAG	TACT	This study
154611	pEPYC0CM0184	PROM	MinSyn_058	MoClo, Loop, GB	GGAG	TACT	This study
154556	pEPYC0CM0401	PROM	MinSyn_060	MoClo, Loop, GB	GGAG	TACT	This study
154557	pEPYC0CM0402	PROM	MinSyn_061	MoClo, Loop, GB	GGAG	TACT	This study
154558	pEPYC0CM0455	PROM	MinSyn_062	MoClo, Loop, GB	GGAG	TACT	This study
154559	pEPYC0CM0456	PROM	MinSyn_063	MoClo, Loop, GB	GGAG	TACT	This study
154560	pEPAS0CMR0015	PROM	MinSyn_064	MoClo, Loop, GB	GGAG	AATG	This study
154561	pEPAS0CMR0016	PROM	MinSyn_065	MoClo, Loop, GB	GGAG	AATG	This study
154562	pEPKK0CM0107	PROM	MinSyn_066	MoClo, Loop, GB	GGAG	AATG	This study
154563	pEPKK0CM0065	PROM	MinSyn_067	MoClo, Loop, GB	GGAG	AATG	This study
154590	pEPKK0CM0062	DIST+PROX+CORE	MinSyn_068	MoClo, Loop, GB	GGAG	CCAT	This study
154592	pEPKK0CM0111	NTAG	MinSyn_068 and MinSyn_069	MoClo, Loop, GB	CCAT	AATG	This study
154589	pEPKK0CM0063	DIST	MinSyn_069	MoClo, Loop, GB	GGAG	TGAC	This study
154591	pEPKK0CM0064	PROX+CORE	MinSyn_069	MoClo, Loop, GB	TGAC	CCAT	This study
154564	pEPYC0CM0407	PROM	MinSyn_101	MoClo, Loop, GB	GGAG	TACT	This study
154565	pEPYC0CM0408	PROM	MinSyn_102	MoClo, Loop, GB	GGAG	TACT	This study
154566	pEPYC0CM0409	PROM	MinSyn_103	MoClo, Loop, GB	GGAG	TACT	This study
154567	pEPYC0CM0410	PROM	MinSyn_104	MoClo, Loop, GB	GGAG	TACT	This study
154568	pEPYC0CM0411	PROM	MinSyn_105	MoClo, Loop, GB	GGAG	TACT	This study
154569	pEPYC0CM0412	PROM	MinSyn_106	MoClo, Loop, GB	GGAG	TACT	This study
154570	pEPYC0CM0413	PROM	MinSyn_107	MoClo, Loop, GB	GGAG	TACT	This study
154571	pEPYC0CM0414	PROM	MinSyn_108	MoClo, Loop, GB	GGAG	TACT	This study
154572	pEPYC0CM0415	PROM	MinSyn_109	MoClo, Loop, GB	GGAG	TACT	This study
154573	pEPYC0CM0416	PROM	MinSyn_110	MoClo, Loop, GB	GGAG	TACT	This study
154574	pEPYC0CM0417	PROM	MinSyn_111	MoClo, Loop, GB	GGAG	TACT	This study
154575	pEPYC0CM0418	PROM	MinSyn_112	MoClo, Loop, GB	GGAG	TACT	This study
154576	pEPYC0CM0419	PROM	MinSyn_113	MoClo, Loop, GB	GGAG	TACT	This study
154577	pEPYC0CM0420	PROM	MinSyn_114	MoClo, Loop, GB	GGAG	TACT	This study
154578	pEPYC0CM0421	PROM	MinSyn_115	MoClo, Loop, GB	GGAG	TACT	This study
154579	pEPYC0CM0422	PROM	MinSyn_116	MoClo, Loop, GB	GGAG	TACT	This study
154580	pEPYC0CM0423	PROM	MinSyn_117	MoClo, Loop, GB	GGAG	TACT	This study
154581	pEPYC0CM0424	PROM	MinSyn_118	MoClo, Loop, GB	GGAG	TACT	This study
154582	pEPYC0CM0425	PROM	MinSyn_119	MoClo, Loop, GB	GGAG	TACT	This study
154583	pEPYC0CM0426	PROM	MinSyn_120	MoClo, Loop, GB	GGAG	TACT	This study
154584	pEPYC0CM0427	PROM	MinSyn_121	MoClo, Loop, GB	GGAG	TACT	This study
154585	pEPYC0CM0428	PROM	MinSyn_122	MoClo, Loop, GB	GGAG	TACT	This study
154586	pEPYC0CM0429	PROM	MinSyn_123	MoClo, Loop, GB	GGAG	TACT	This study
154587	pEPYC0CM0430	PROM	MinSyn_124	MoClo, Loop, GB	GGAG	TACT	This study
154588	pEPYC0CM0244	PROM	MinSyn_285	MoClo, Loop, GB	GGAG	TACT	This study
154481	pEPYC0CM0099	PROM	MMV (C-CRE relocated)	MoClo, Loop, GB	GGAG	TACT	This study
154482	pEPYC0CM0115	PROM	MMV (C-CRE relocated)	MoClo, Loop, GB	GGAG	TACT	This study
154478	pEPYC0CM0095	PROM	MMV (Mirabalis Mosaic Virus)	MoClo, Loop, GB	GGAG	TACT	This study
154496	pEPYC0CM0290	PROM	MMV(ΔbHLH+)	MoClo, Loop, GB	GGAG	TACT	This study
154497	pEPYC0CM0289	PROM	MMV(ΔbHLH$_2$)	MoClo, Loop, GB	GGAG	TACT	This study
154603	pEPYC0CM0175	PROM	MMV(ΔC_CRE)	MoClo, Loop, GB	GGAG	TACT	This study
154602	pEPYC0CM0174	PROM	MMV(ΔC2C2dof+$_2$)	MoClo, Loop, GB	GGAG	TACT	This study
154495	pEPYC0CM0292	PROM	MMV(ΔCCAAT+)	MoClo, Loop, GB	GGAG	TACT	This study
154494	pEPYC0CM0288	PROM	MMV(ΔG2like)	MoClo, Loop, GB	GGAG	TACT	This study
154498	pEPYC0CM0286	PROM	MMV(ΔMADS+$_2$)	MoClo, Loop, GB	GGAG	TACT	This study
154604	pEPYC0CM0172	PROM	MMV(ΔMYB+)	MoClo, Loop, GB	GGAG	TACT	This study
154608	pEPYC0CM0173	PROM	MMV(ΔTCP$_2$)	MoClo, Loop, GB	GGAG	TACT	This study
154502	pEPYC0CM0287	PROM	MMV(ΔTCP$_{3)}$	MoClo, Loop, GB	GGAG	TACT	This study
154499	pEPYC0CM0291	PROM	MMV(ΔWRKY+)	MoClo, Loop, GB	GGAG	TACT	This study
50339	pICH41421	3UTR+TERM	NOS (Agrobacterium tumefaciens)	MoClo, Loop, GB	GCTT	CGCT	Engler et al (2014)
50255	pICH42211	PROM	NOS (Agrobacterium tumefaciens)	MoClo, Loop, GB	GGAG	TACT	Engler et al (2014)
154484	pEPYC0CM0119	PROM	NOS (C-CRE relocated)	MoClo, Loop, GB	GGAG	TACT	This study
154483	pEPYC0CM0120	PROM	NOS (C-CRE relocated)	MoClo, Loop, GB	GGAG	TACT	This study
154500	pEPYC0CM0294	PROM	NOS(ΔAP2+$_2$)	MoClo, Loop, GB	GGAG	TACT	This study
154605	pEPYC0CM0178	PROM	NOS(ΔC_CRE)	MoClo, Loop, GB	GGAG	TACT	This study
154606	pEPYC0CM0177	PROM	NOS(ΔC2H2)	MoClo, Loop, GB	GGAG	TACT	This study
154501	pEPYC0CM0293	PROM	NOS(ΔG2like+)	MoClo, Loop, GB	GGAG	TACT	This study
154607	pEPYC0CM0176	PROM	NOS(Δhomeobox)	MoClo, Loop, GB	GGAG	TACT	This study
68257	pICSL12009	PROM	ZmUbi (Zae mays)	MoClo, Loop, GB	GGAG	AATG	Lawrenson et al (2015)
68260	pICSL80037	CDS	NptII	MoClo, Loop, GB	AATG	GCTT	Lawrenson et al (2015)
50343	pICH41432	3UTR+TERM	OCS (Agrobacterium tumefaciens)	MoClo, Loop, GB	GCTT	CGCT	Engler et al (2014)
-	GB_UD_32AB	CDS	PhiC3 binding domain	GB	AATG	GCTT	Vazquez-Vilar et al (2017)
154596	pEPKK0CM0068	CDS	TALE	MoClo, Loop, GB	AATG	GCTT	This study
50285	pICH41402	5UTR	TMV**Ω** (Tobacco Mosaic Virus)	MoClo, Loop, GB	TACT	AATG	Engler et al (2014)
50332	pICSL80016	CDS	uidA (GUS)	MoClo, Loop, GB	AATG	TTCG	Engler et al (2014)
117536	pICSL50005	CTAG	YFP	MoClo, Loop, GB	TTCG	GCTT	Raitskin et al (2019)

2. Expression cassettes

Addgene #	Plasmid code	Level 0 Parts: PROM: DIST	PROM: PROX	PROM: CORE	5UTR	NTAG	CDS	CTAG	3'UTR/TERM	Acceptor	Cloning overhang: 5'	Cloning overhang: 3'	Source
154624	pEPYC1CB0308		pICSL12006		-	-	pUAP80037	-	pICH41414	pICH47732	TGCC	GCAA	This study
154625	pEPYC1CB0305		pEPYC0CM0244		pICSL20002	pEPYC0CM0258	pICSL80016	pICSL50005	pICH41432	pICH47811	TAGT	TTGC	This study
154626	pEPYC1CB0477		pEPYC0CM0410		pICSL20002	pEPYC0CM0258	pICSL80016	pICSL50005	pICH41432	pICH47811	TAGT	TTGC	This study
154627	pEPYC1CB0478		pEPYC0CM0414		pICSL20002	pEPYC0CM0258	pICSL80016	pICSL50005	pICH41432	pICH47811	TAGT	TTGC	This study
154628	pEPYC1CB0503		pEPYC0CM0035		pICSL20002	pEPYC0CM0258	pICSL80016	pICSL50005	pICH41432	pICH47811	TAGT	TTGC	This study
154612	pEPYCα1KN0002		pEPYC0CM0414		pICSL20002	GB0900	GB_UD_32AB	-	pICH41432	pDGB3_α1	GGAG	GTCA	This study
154614	pEPYCα1KN0007		pEPYC0CM0414		pICH41402	-	pEPKK0CM0068	-	pICH41432	pDGB3_α1	GGAG	GTCA	This study
-	GB_UA_114A		GB0552		-	GB0900	GB_UD_32AB	-	GB0036	pDGB3_α1	GGAG	GTCA	Vazquez-Vilar et al (2017)
154615	pEPYCα1KN0008		pEPKK0CM0107		-	-	pEPAS0CM0008	pICSL50007	pICH72400	pDGB3_α1	GGAG	GTCA	This study
154616	pEPYCα2rKN0009		pEPKK0CM0107		-	-	pEPYC0CM0133	pICSL50007	pICH41421	pDGB3_α2R	GTCA	GGAG	This study
154617	pEPYCα2rKN0010		pEPAS0CMR0015		-	-	pEPYC0CM0133	pICSL50007	pICH41421	pDGB3_α2R	GTCA	GGAG	This study
154618	pEPKKα2KN0091		pEPAS0CMR0015		-	-	pEPYC0CM0133	pICSL50007	pICH72400	pDGB3_α1	GGAG	GTCA	This study
154619	pEPKKα2KN0092		pEPAS0CMR0016		-	-	pEPYC0CM0133	pICSL50007	pICH72400	pDGB3_α1	GGAG	GTCA	This study
154620	pEPKKα2KN0093		pEPKK0CM0107		-	-	pEPYC0CM0133	pICSL50007	pICH72400	pDGB3_α1	GGAG	GTCA	This study
154621	pEPKKα2KN0100		pEPKK0CM0065		-	-	pEPYC0CM0133	pICSL50007	pICH72400	pDGB3_α1	GGAG	GTCA	This study
154622	pEPKKα2KN0101		pEPKK0CM0062			pEPKK0CM0111	pEPYC0CM0133	pICSL50007	pICH72400	pDGB3_α1	GGAG	GTCA	This study
154623	pEPKKα2KN0102	pEPKK0CM0063	pEPKK0CM0064			pEPKK0CM0111	pEPYC0CM0133	pICSL50007	pICH72400	pDGB3_α1	GGAG	GTCA	This study
154629	pEPYC1CB0003		pICH42211		pICH41402	-	pEPAS0CM0008	pICSL50007	pICH41432	pICH47732	TGCC	GCAA	This study
154630	pEPYC1CB0007		pEPYC0CM0035		pICH41402	-	pEPAS0CM0008	pICSL50007	pICH41432	pICH47732	TGCC	GCAA	This study
154631	pEPYC1CB0079		pEPYC0CM0041		pICH41402	-	pEPAS0CM0008	pICSL50007	pICH41432	pICH47732	TGCC	GCAA	This study
154632	pEPYC1CB0109		pEPYC0CM0071		pICH41402	-	pEPAS0CM0008	pICSL50007	pICH41432	pICH47732	TGCC	GCAA	This study
154633	pEPYC1CB0122		pEPYC0CM0084		pICH41402	-	pEPAS0CM0008	pICSL50007	pICH41432	pICH47732	TGCC	GCAA	This study
154634	pEPYC1CB0127		pEPYC0CM0089		pICH41402	-	pEPAS0CM0008	pICSL50007	pICH41432	pICH47732	TGCC	GCAA	This study
154635	pEPYC1CB0133		pEPYC0CM0095		pICH41402	-	pEPAS0CM0008	pICSL50007	pICH41432	pICH47732	TGCC	GCAA	This study
154636	pEPYC1CB0137		pEPYC0CM0099		pICH41402	-	pEPAS0CM0008	pICSL50007	pICH41432	pICH47732	TGCC	GCAA	This study
154637	pEPYC1CB0153		pEPYC0CM0115		pICH41402	-	pEPAS0CM0008	pICSL50007	pICH41432	pICH47732	TGCC	GCAA	This study
154638	pEPYC1CB0157		pEPYC0CM0119		pICH41402	-	pEPAS0CM0008	pICSL50007	pICH41432	pICH47732	TGCC	GCAA	This study
154639	pEPYC1CB0158		pEPYC0CM0120		pICH41402	-	pEPAS0CM0008	pICSL50007	pICH41432	pICH47732	TGCC	GCAA	This study
154640	pEPYC1CB0173		pEPYC0CM0168		pICH41402	-	pEPAS0CM0008	pICSL50007	pICH41432	pICH47732	TGCC	GCAA	This study
154641	pEPYC1CB0174		pEPYC0CM0169		pICH41402	-	pEPAS0CM0008	pICSL50007	pICH41432	pICH47732	TGCC	GCAA	This study
154642	pEPYC1CB0175		pEPYC0CM0170		pICH41402	-	pEPAS0CM0008	pICSL50007	pICH41432	pICH47732	TGCC	GCAA	This study
154643	pEPYC1CB0176		pEPYC0CM0171		pICH41402	-	pEPAS0CM0008	pICSL50007	pICH41432	pICH47732	TGCC	GCAA	This study
154644	pEPYC1CB0177		pEPYC0CM0172		pICH41402	-	pEPAS0CM0008	pICSL50007	pICH41432	pICH47732	TGCC	GCAA	This study
154645	pEPYC1CB0178		pEPYC0CM0173		pICH41402	-	pEPAS0CM0008	pICSL50007	pICH41432	pICH47732	TGCC	GCAA	This study
154646	pEPYC1CB0179		pEPYC0CM0174		pICH41402	-	pEPAS0CM0008	pICSL50007	pICH41432	pICH47732	TGCC	GCAA	This study
154647	pEPYC1CB0180		pEPYC0CM0175		pICH41402	-	pEPAS0CM0008	pICSL50007	pICH41432	pICH47732	TGCC	GCAA	This study
154648	pEPYC1CB0181		pEPYC0CM0176		pICH41402	-	pEPAS0CM0008	pICSL50007	pICH41432	pICH47732	TGCC	GCAA	This study
154649	pEPYC1CB0182		pEPYC0CM0177		pICH41402	-	pEPAS0CM0008	pICSL50007	pICH41432	pICH47732	TGCC	GCAA	This study
154650	pEPYC1CB0183		pEPYC0CM0178		pICH41402	-	pEPAS0CM0008	pICSL50007	pICH41432	pICH47732	TGCC	GCAA	This study
154651	pEPYC1CB0187		pEPYC0CM0182		pICH41402	-	pEPAS0CM0008	pICSL50007	pICH41432	pICH47732	TGCC	GCAA	This study
154652	pEPYC1CB0188		pEPYC0CM0183		pICH41402	-	pEPAS0CM0008	pICSL50007	pICH41432	pICH47732	TGCC	GCAA	This study
154653	pEPYC1CB0189		pEPYC0CM0184		pICH41402	-	pEPAS0CM0008	pICSL50007	pICH41432	pICH47732	TGCC	GCAA	This study
154654	pEPYC1CB0197		pICH42211		pICH41402	-	pEPYC0CM0133	pICSL50007	pICH41421	pICH47732	TGCC	GCAA	This study
154655	pEPYC1CB0199		pICH85281		pICH41402	-	pEPAS0CM0008	pICSL50007	pICH41432	pICH47732	TGCC	GCAA	This study
154656	pEPYC1CB0253		pEPYC0CM0209		pICH41402	-	pEPAS0CM0008	pICSL50007	pICH41432	pICH47732	TGCC	GCAA	This study
154657	pEPYC1CB0254		pEPYC0CM0210		pICH41402	-	pEPAS0CM0008	pICSL50007	pICH41432	pICH47732	TGCC	GCAA	This study
154658	pEPYC1CB0255		pEPYC0CM0211		pICH41402	-	pEPAS0CM0008	pICSL50007	pICH41432	pICH47732	TGCC	GCAA	This study
154659	pEPYC1CB0256		pEPYC0CM0212		pICH41402	-	pEPAS0CM0008	pICSL50007	pICH41432	pICH47732	TGCC	GCAA	This study
154660	pEPYC1CB0257		pEPYC0CM0213		pICH41402	-	pEPAS0CM0008	pICSL50007	pICH41432	pICH47732	TGCC	GCAA	This study
154661	pEPYC1CB0258		pEPYC0CM0214		pICH41402	-	pEPAS0CM0008	pICSL50007	pICH41432	pICH47732	TGCC	GCAA	This study
154662	pEPYC1CB0269		pEPYC0CM0225		pICH41402	-	pEPAS0CM0008	pICSL50007	pICH41432	pICH47732	TGCC	GCAA	This study
154663	pEPYC1CB0286		pEPYC0CM0244		pICH41402	-	pEPYC0CM0133	pICSL50007	pICH41421	pICH47732	TGCC	GCAA	This study
154664	pEPYC1CB0319		pEPYC0CM0277		pICH41402	-	pEPAS0CM0008	pICSL50007	pICH41432	pICH47732	TGCC	GCAA	This study
154665	pEPYC1CB0320		pEPYC0CM0278		pICH41402	-	pEPAS0CM0008	pICSL50007	pICH41432	pICH47732	TGCC	GCAA	This study
154666	pEPYC1CB0321		pEPYC0CM0279		pICH41402	-	pEPAS0CM0008	pICSL50007	pICH41432	pICH47732	TGCC	GCAA	This study
154667	pEPYC1CB0322		pEPYC0CM0280		pICH41402	-	pEPAS0CM0008	pICSL50007	pICH41432	pICH47732	TGCC	GCAA	This study
154668	pEPYC1CB0323		pEPYC0CM0281		pICH41402	-	pEPAS0CM0008	pICSL50007	pICH41432	pICH47732	TGCC	GCAA	This study
154669	pEPYC1CB0324		pEPYC0CM0282		pICH41402	-	pEPAS0CM0008	pICSL50007	pICH41432	pICH47732	TGCC	GCAA	This study
154670	pEPYC1CB0325		pEPYC0CM0283		pICH41402	-	pEPAS0CM0008	pICSL50007	pICH41432	pICH47732	TGCC	GCAA	This study
154671	pEPYC1CB0326		pEPYC0CM0284		pICH41402	-	pEPAS0CM0008	pICSL50007	pICH41432	pICH47732	TGCC	GCAA	This study
154672	pEPYC1CB0327		pEPYC0CM0285		pICH41402	-	pEPAS0CM0008	pICSL50007	pICH41432	pICH47732	TGCC	GCAA	This study
154673	pEPYC1CB0328		pEPYC0CM0286		pICH41402	-	pEPAS0CM0008	pICSL50007	pICH41432	pICH47732	TGCC	GCAA	This study
154674	pEPYC1CB0329		pEPYC0CM0287		pICH41402	-	pEPAS0CM0008	pICSL50007	pICH41432	pICH47732	TGCC	GCAA	This study
154675	pEPYC1CB0330		pEPYC0CM0288		pICH41402	-	pEPAS0CM0008	pICSL50007	pICH41432	pICH47732	TGCC	GCAA	This study
154676	pEPYC1CB0331		pEPYC0CM0289		pICH41402	-	pEPAS0CM0008	pICSL50007	pICH41432	pICH47732	TGCC	GCAA	This study
154677	pEPYC1CB0332		pEPYC0CM0290		pICH41402	-	pEPAS0CM0008	pICSL50007	pICH41432	pICH47732	TGCC	GCAA	This study
154678	pEPYC1CB0333		pEPYC0CM0291		pICH41402	-	pEPAS0CM0008	pICSL50007	pICH41432	pICH47732	TGCC	GCAA	This study
154679	pEPYC1CB0334		pEPYC0CM0292		pICH41402	-	pEPAS0CM0008	pICSL50007	pICH41432	pICH47732	TGCC	GCAA	This study
154680	pEPYC1CB0335		pEPYC0CM0293		pICH41402	-	pEPAS0CM0008	pICSL50007	pICH41432	pICH47732	TGCC	GCAA	This study
154681	pEPYC1CB0336		pEPYC0CM0294		pICH41402	-	pEPAS0CM0008	pICSL50007	pICH41432	pICH47732	TGCC	GCAA	This study
154682	pEPYC1CB0352		pEPYC0CM0310		pICH41402	-	pEPAS0CM0008	pICSL50007	pICH41432	pICH47732	TGCC	GCAA	This study
154683	pEPYC1CB0353		pEPYC0CM0311		pICH41402	-	pEPAS0CM0008	pICSL50007	pICH41432	pICH47732	TGCC	GCAA	This study
154684	pEPYC1CB0354		pEPYC0CM0312		pICH41402	-	pEPAS0CM0008	pICSL50007	pICH41432	pICH47732	TGCC	GCAA	This study
154685	pEPYC1CB0355		pEPYC0CM0313		pICH41402	-	pEPAS0CM0008	pICSL50007	pICH41432	pICH47732	TGCC	GCAA	This study
154686	pEPYC1CB0356		pEPYC0CM0314		pICH41402	-	pEPAS0CM0008	pICSL50007	pICH41432	pICH47732	TGCC	GCAA	This study
154687	pEPYC1CB0425		pEPYC0CM0365		pICH41402	-	pEPAS0CM0008	pICSL50007	pICH41432	pICH47732	TGCC	GCAA	This study
154688	pEPYC1CB0426		pEPYC0CM0366		pICH41402	-	pEPAS0CM0008	pICSL50007	pICH41432	pICH47732	TGCC	GCAA	This study
154689	pEPYC1CB0427		pEPYC0CM0367		pICH41402	-	pEPAS0CM0008	pICSL50007	pICH41432	pICH47732	TGCC	GCAA	This study
154690	pEPYC1CB0428		pEPYC0CM0368		pICH41402	-	pEPAS0CM0008	pICSL50007	pICH41432	pICH47732	TGCC	GCAA	This study
154691	pEPYC1CB0432		pEPYC0CM0372		pICH41402	-	pEPAS0CM0008	pICSL50007	pICH41432	pICH47732	TGCC	GCAA	This study
154692	pEPYC1CB0433		pEPYC0CM0373		pICH41402	-	pEPAS0CM0008	pICSL50007	pICH41432	pICH47732	TGCC	GCAA	This study
154693	pEPYC1CB0434		pEPYC0CM0374		pICH41402	-	pEPAS0CM0008	pICSL50007	pICH41432	pICH47732	TGCC	GCAA	This study
154694	pEPYC1CB0435		pEPYC0CM0375		pICH41402	-	pEPAS0CM0008	pICSL50007	pICH41432	pICH47732	TGCC	GCAA	This study
154695	pEPYC1CB0436		pEPYC0CM0376		pICH41402	-	pEPAS0CM0008	pICSL50007	pICH41432	pICH47732	TGCC	GCAA	This study
154696	pEPYC1CB0437		pEPYC0CM0377		pICH41402	-	pEPAS0CM0008	pICSL50007	pICH41432	pICH47732	TGCC	GCAA	This study
154697	pEPYC1CB0439		pEPYC0CM0379		pICH41402	-	pEPAS0CM0008	pICSL50007	pICH41432	pICH47732	TGCC	GCAA	This study
154698	pEPYC1CB0440		pEPYC0CM0380		pICH41402	-	pEPAS0CM0008	pICSL50007	pICH41432	pICH47732	TGCC	GCAA	This study
154699	pEPYC1CB0441		pEPYC0CM0381		pICH41402	-	pEPAS0CM0008	pICSL50007	pICH41432	pICH47732	TGCC	GCAA	This study
154700	pEPYC1CB0442		pEPYC0CM0382		pICH41402	-	pEPAS0CM0008	pICSL50007	pICH41432	pICH47732	TGCC	GCAA	This study
154701	pEPYC1CB0443		pEPYC0CM0383		pICH41402	-	pEPAS0CM0008	pICSL50007	pICH41432	pICH47732	TGCC	GCAA	This study
154702	pEPYC1CB0444		pEPYC0CM0384		pICH41402	-	pEPAS0CM0008	pICSL50007	pICH41432	pICH47732	TGCC	GCAA	This study
154703	pEPYC1CB0445		pEPYC0CM0385		pICH41402	-	pEPAS0CM0008	pICSL50007	pICH41432	pICH47732	TGCC	GCAA	This study
154704	pEPYC1CB0446		pEPYC0CM0386		pICH41402	-	pEPAS0CM0008	pICSL50007	pICH41432	pICH47732	TGCC	GCAA	This study
154705	pEPYC1CB0447		pEPYC0CM0387		pICH41402	-	pEPAS0CM0008	pICSL50007	pICH41432	pICH47732	TGCC	GCAA	This study
154706	pEPYC1CB0450		pEPYC0CM0390		pICH41402	-	pEPAS0CM0008	pICSL50007	pICH41432	pICH47732	TGCC	GCAA	This study
154707	pEPYC1CB0451		pEPYC0CM0391		pICH41402	-	pEPAS0CM0008	pICSL50007	pICH41432	pICH47732	TGCC	GCAA	This study
154708	pEPYC1CB0452		pEPYC0CM0392		pICH41402	-	pEPAS0CM0008	pICSL50007	pICH41432	pICH47732	TGCC	GCAA	This study
154709	pEPYC1CB0453		pEPYC0CM0393		pICH41402	-	pEPAS0CM0008	pICSL50007	pICH41432	pICH47732	TGCC	GCAA	This study
154710	pEPYC1CB0455		pEPYC0CM0395		pICH41402	-	pEPAS0CM0008	pICSL50007	pICH41432	pICH47732	TGCC	GCAA	This study

		Level 0 Parts									Cloning overhang		
		PROM											
Addgene #	Plasmid code	DIST	PROX	CORE	5UTR	NTAG	CDS	CTAG	3'UTR/TERM	Acceptor	5'	3'	Source
154711	pEPYC1CB0456		pEPYC0CM0396		pICH41402	-	pEPAS0CM0008	pICSL50007	pICH41432	pICH47732	TGCC	GCAA	This study
154712	pEPYC1CB0457		pEPYC0CM0397		pICH41402	-	pEPAS0CM0008	pICSL50007	pICH41432	pICH47732	TGCC	GCAA	This study
154713	pEPYC1CB0458		pEPYC0CM0398		pICH41402	-	pEPAS0CM0008	pICSL50007	pICH41432	pICH47732	TGCC	GCAA	This study
154714	pEPYC1CB0461		pEPYC0CM0401		pICH41402	-	pEPAS0CM0008	pICSL50007	pICH41432	pICH47732	TGCC	GCAA	This study
154715	pEPYC1CB0462		pEPYC0CM0402		pICH41402	-	pEPAS0CM0008	pICSL50007	pICH41432	pICH47732	TGCC	GCAA	This study
154716	pEPYC1CB0467		pEPYC0CM0407		pICH41402	-	pEPAS0CM0008	pICSL50007	pICH41432	pICH47732	TGCC	GCAA	This study
154717	pEPYC1CB0468		pEPYC0CM0408		pICH41402	-	pEPAS0CM0008	pICSL50007	pICH41432	pICH47732	TGCC	GCAA	This study
154718	pEPYC1CB0469		pEPYC0CM0409		pICH41402	-	pEPAS0CM0008	pICSL50007	pICH41432	pICH47732	TGCC	GCAA	This study
154719	pEPYC1CB0470		pEPYC0CM0410		pICH41402	-	pEPAS0CM0008	pICSL50007	pICH41432	pICH47732	TGCC	GCAA	This study
154720	pEPYC1CB0471		pEPYC0CM0411		pICH41402	-	pEPAS0CM0008	pICSL50007	pICH41432	pICH47732	TGCC	GCAA	This study
154721	pEPYC1CB0472		pEPYC0CM0412		pICH41402	-	pEPAS0CM0008	pICSL50007	pICH41432	pICH47732	TGCC	GCAA	This study
154722	pEPYC1CB0473		pEPYC0CM0413		pICH41402	-	pEPAS0CM0008	pICSL50007	pICH41432	pICH47732	TGCC	GCAA	This study
154723	pEPYC1CB0474		pEPYC0CM0414		pICH41402	-	pEPAS0CM0008	pICSL50007	pICH41432	pICH47732	TGCC	GCAA	This study
154724	pEPYC1CB0475		pEPYC0CM0415		pICH41402	-	pEPAS0CM0008	pICSL50007	pICH41432	pICH47732	TGCC	GCAA	This study
154725	pEPYC1CB0476		pEPYC0CM0416		pICH41402	-	pEPAS0CM0008	pICSL50007	pICH41432	pICH47732	TGCC	GCAA	This study
154726	pEPYC1CB0479		pEPYC0CM0417		pICH41402	-	pEPAS0CM0008	pICSL50007	pICH41432	pICH47732	TGCC	GCAA	This study
154727	pEPYC1CB0480		pEPYC0CM0418		pICH41402	-	pEPAS0CM0008	pICSL50007	pICH41432	pICH47732	TGCC	GCAA	This study
154728	pEPYC1CB0481		pEPYC0CM0419		pICH41402	-	pEPAS0CM0008	pICSL50007	pICH41432	pICH47732	TGCC	GCAA	This study
154729	pEPYC1CB0482		pEPYC0CM0420		pICH41402	-	pEPAS0CM0008	pICSL50007	pICH41432	pICH47732	TGCC	GCAA	This study
154730	pEPYC1CB0483		pEPYC0CM0421		pICH41402	-	pEPAS0CM0008	pICSL50007	pICH41432	pICH47732	TGCC	GCAA	This study
154731	pEPYC1CB0484		pEPYC0CM0422		pICH41402	-	pEPAS0CM0008	pICSL50007	pICH41432	pICH47732	TGCC	GCAA	This study
154732	pEPYC1CB0485		pEPYC0CM0423		pICH41402	-	pEPAS0CM0008	pICSL50007	pICH41432	pICH47732	TGCC	GCAA	This study
154733	pEPYC1CB0486		pEPYC0CM0424		pICH41402	-	pEPAS0CM0008	pICSL50007	pICH41432	pICH47732	TGCC	GCAA	This study
154734	pEPYC1CB0487		pEPYC0CM0425		pICH41402	-	pEPAS0CM0008	pICSL50007	pICH41432	pICH47732	TGCC	GCAA	This study
154735	pEPYC1CB0488		pEPYC0CM0426		pICH41402	-	pEPAS0CM0008	pICSL50007	pICH41432	pICH47732	TGCC	GCAA	This study
154736	pEPYC1CB0489		pEPYC0CM0427		pICH41402	-	pEPAS0CM0008	pICSL50007	pICH41432	pICH47732	TGCC	GCAA	This study
154737	pEPYC1CB0490		pEPYC0CM0428		pICH41402	-	pEPAS0CM0008	pICSL50007	pICH41432	pICH47732	TGCC	GCAA	This study
154738	pEPYC1CB0491		pEPYC0CM0429		pICH41402	-	pEPAS0CM0008	pICSL50007	pICH41432	pICH47732	TGCC	GCAA	This study
154739	pEPYC1CB0492		pEPYC0CM0430		pICH41402	-	pEPAS0CM0008	pICSL50007	pICH41432	pICH47732	TGCC	GCAA	This study
154740	pEPYC1CB0493		pEPYC0CM0431		pICH41402	-	pEPAS0CM0008	pICSL50007	pICH41432	pICH47732	TGCC	GCAA	This study
154741	pEPYC1CB0495		pEPYC0CM0433		pICH41402	-	pEPAS0CM0008	pICSL50007	pICH41432	pICH47732	TGCC	GCAA	This study
154742	pEPYC1CB0496		pEPYC0CM0434		pICH41402	-	pEPAS0CM0008	pICSL50007	pICH41432	pICH47732	TGCC	GCAA	This study
154743	pEPYC1CB0497		pEPYC0CM0435		pICH41402	-	pEPAS0CM0008	pICSL50007	pICH41432	pICH47732	TGCC	GCAA	This study
154744	pEPYC1CB0498		pEPYC0CM0436		pICH41402	-	pEPAS0CM0008	pICSL50007	pICH41432	pICH47732	TGCC	GCAA	This study
154745	pEPYC1CB0499		pEPYC0CM0437		pICH41402	-	pEPAS0CM0008	pICSL50007	pICH41432	pICH47732	TGCC	GCAA	This study
154746	pEPYC1CB0501		pEPYC0CM0439		pICH41402	-	pEPAS0CM0008	pICSL50007	pICH41432	pICH47732	TGCC	GCAA	This study
154747	pEPYC1CB0502		pEPYC0CM0440		pICH41402	-	pEPAS0CM0008	pICSL50007	pICH41432	pICH47732	TGCC	GCAA	This study
154748	pEPYC1CB0509		pEPYC0CM0451		pICH41402	-	pEPAS0CM0008	pICSL50007	pICH41432	pICH47732	TGCC	GCAA	This study
154749	pEPYC1CB0510		pEPYC0CM0452		pICH41402	-	pEPAS0CM0008	pICSL50007	pICH41432	pICH47732	TGCC	GCAA	This study
154750	pEPYC1CB0511		pEPYC0CM0453		pICH41402	-	pEPAS0CM0008	pICSL50007	pICH41432	pICH47732	TGCC	GCAA	This study
154751	pEPYC1CB0512		pEPYC0CM0454		pICH41402	-	pEPAS0CM0008	pICSL50007	pICH41432	pICH47732	TGCC	GCAA	This study
154752	pEPYC1CB0513		pEPYC0CM0455		pICH41402	-	pEPAS0CM0008	pICSL50007	pICH41432	pICH47732	TGCC	GCAA	This study
154753	pEPYC1CB0514		pEPYC0CM0456		pICH41402	-	pEPAS0CM0008	pICSL50007	pICH41432	pICH47732	TGCC	GCAA	This study

Supplementary Data 3

Expression profiles of *Arabidopsis thaliana* genes encoding transcription factors predicted to bind to the following constitutive promoters:

(1) Cauliflower Mosaic Virus 35s (*CaMV35s*)
(2) Mirabilis Mosaic Virus (*MMV*)
(3) Agrobacterium tumefaciens *nopaline synthase* (*AtuNOS*)
(4) *A. thaliana* actin (*AtACT2*)
(5) *A. thaliana* ubiquitin-conjugating enzyme 9 (*AtUBC9*)
(6) *A. thaliana* polyubiquitin 10 (*AtUBI10*)
TPM = Transcripts per million

(1)

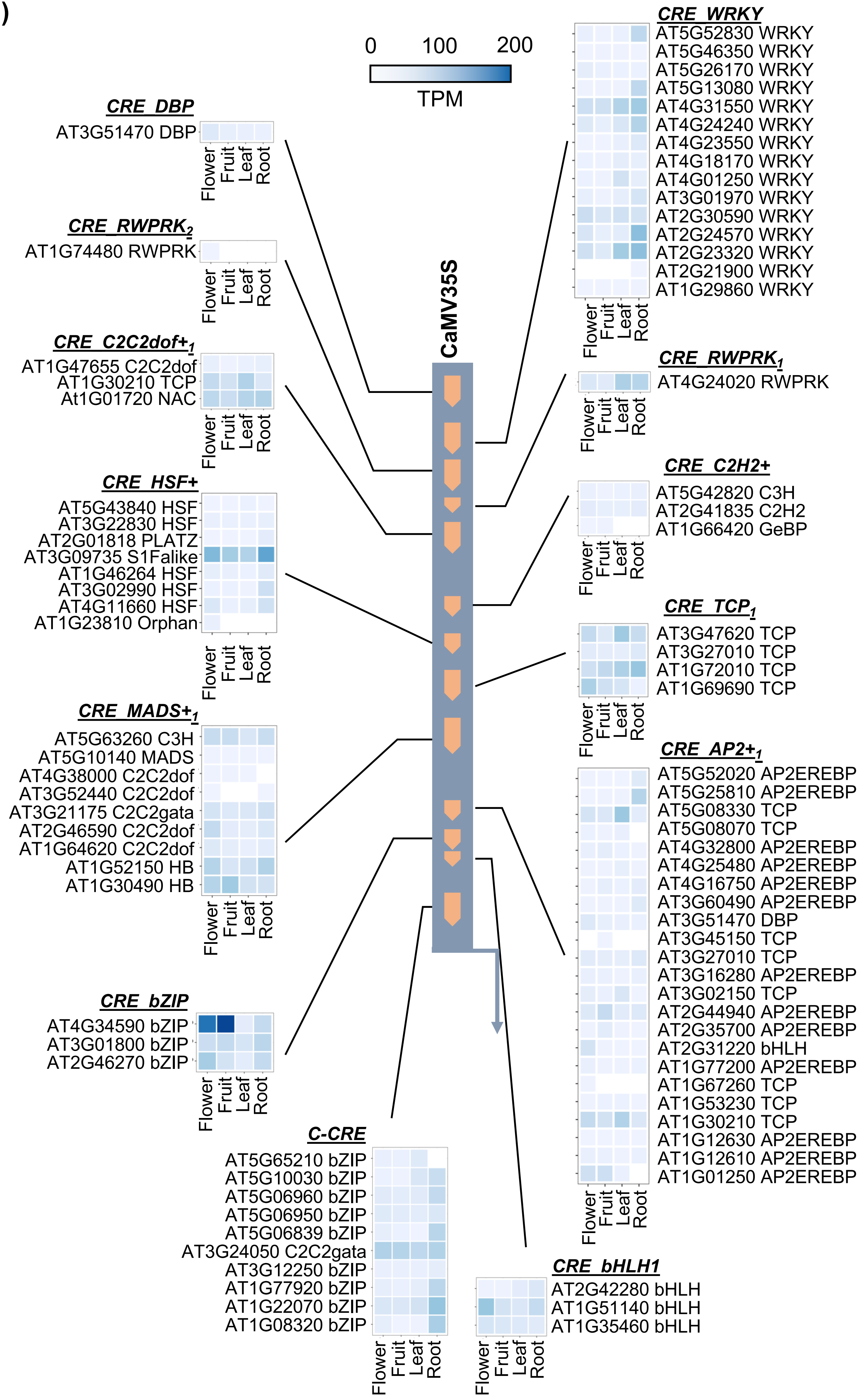

0
100
200
TPM
CaMV35S
CRE_DBP
AT3G51470 DBP
CRE_RWPRK2
AT1G74480 RWPRK
CRE_C2C2dof+1
AT1G47655 C2C2dof
AT1G30210 TCP
At1G01720 NAC
CRE_HSF+
AT5G43840 HSF
AT3G22830 HSF
AT2G01818 PLATZ
AT3G09735 S1Falike
AT1G46264 HSF
AT3G02990 HSF
AT4G11660 HSF
AT1G23810 Orphan
CRE_MADS+1
AT5G63260 C3H
AT5G10140 MADS
AT4G38000 C2C2dof
AT3G52440 C2C2dof
AT3G21175 C2C2gata
AT2G46590 C2C2dof
AT1G64620 C2C2dof
AT1G52150 HB
AT1G30490 HB
CRE_bZIP
AT4G34590 bZIP
AT3G01800 bZIP
AT2G46270 bZIP
C-CRE
AT5G65210 bZIP
AT5G10030 bZIP
AT5G06960 bZIP
AT5G06950 bZIP
AT5G06839 bZIP
AT3G24050 C2C2gata
AT3G12250 bZIP
AT1G77920 bZIP
AT1G22070 bZIP
AT1G08320 bZIP
CRE_WRKY
AT5G52830 WRKY
AT5G46350 WRKY
AT5G26170 WRKY
AT5G13080 WRKY
AT4G31550 WRKY
AT4G24240 WRKY
AT4G23550 WRKY
AT4G18170 WRKY
AT4G01250 WRKY
AT3G01970 WRKY
AT2G30590 WRKY
AT2G24570 WRKY
AT2G23320 WRKY
AT2G21900 WRKY
AT1G29860 WRKY
CRE_RWPRK1
AT4G24020 RWPRK
CRE_C2H2+
AT5G42820 C3H
AT2G41835 C2H2
AT1G66420 GeBP
CRE_TCP1
AT3G47620 TCP
AT3G27010 TCP
AT1G72010 TCP
AT1G69690 TCP
CRE_AP2+1
AT5G52020 AP2EREBP
AT5G25810 AP2EREBP
AT5G08330 TCP
AT5G08070 TCP
AT4G32800 AP2EREBP
AT4G25480 AP2EREBP
AT4G16750 AP2EREBP
AT3G60490 AP2EREBP
AT3G51470 DBP
AT3G45150 TCP
AT3G27010 TCP
AT3G16280 AP2EREBP
AT3G02150 TCP
AT2G44940 AP2EREBP
AT2G35700 AP2EREBP
AT2G31220 bHLH
AT1G77200 AP2EREBP
AT1G67260 TCP
AT1G53230 TCP
AT1G30210 TCP
AT1G12630 AP2EREBP
AT1G12610 AP2EREBP
AT1G01250 AP2EREBP
CRE_bHLH1
AT2G42280 bHLH
AT1G51140 bHLH
AT1G35460 bHLH
Flower
Fruit
Leaf
Root

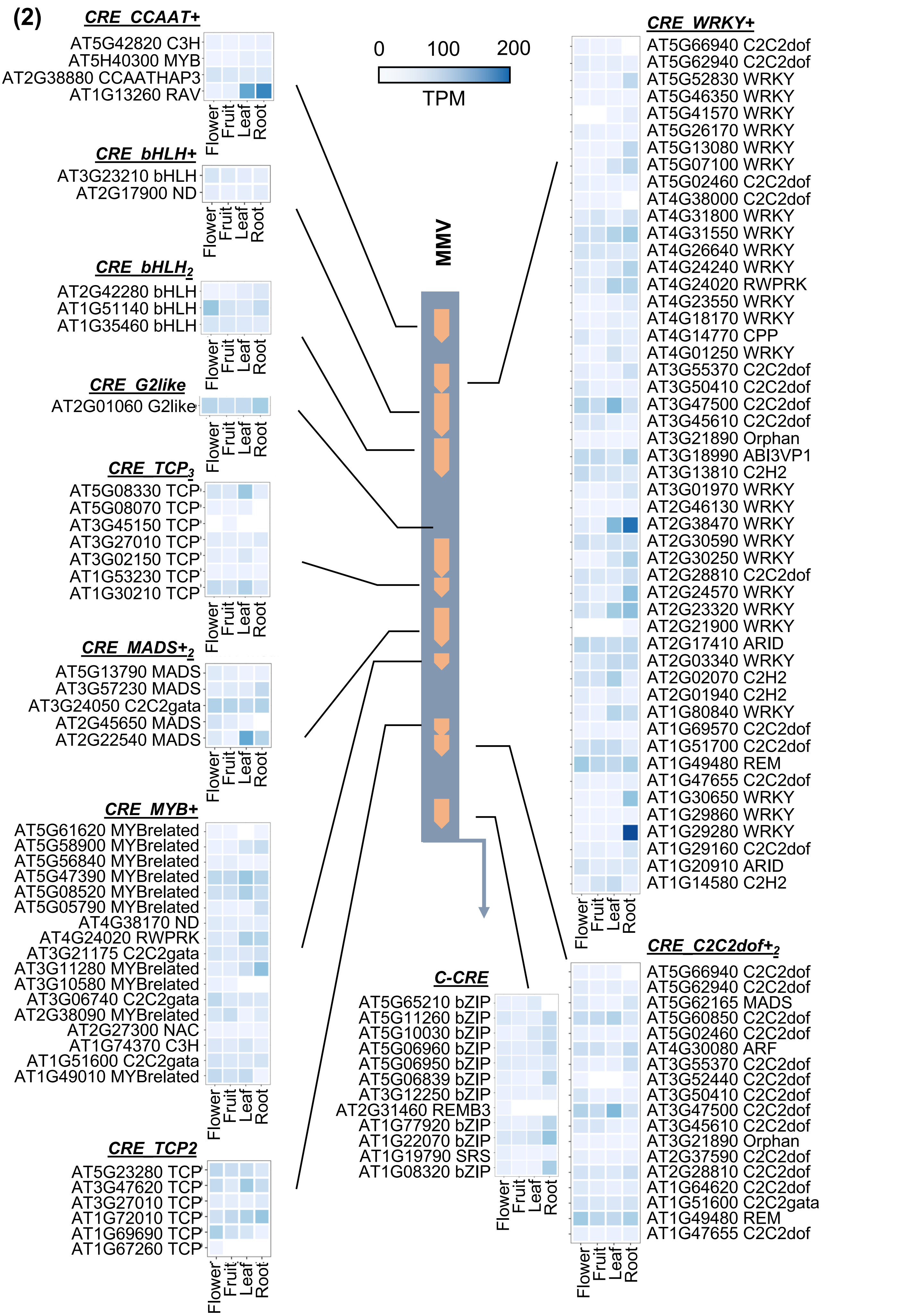

(2)
0
100
200
TPM
MMV
CRE_CCAAT+
AT5G42820 C3H
AT5H40300 MYB
AT2G38880 CCAATHAP3
AT1G13260 RAV
Flower
Fruit
Leaf
Root
CRE_bHLH+
AT3G23210 bHLH
AT2G17900 ND
CRE_bHLH2
AT2G42280 bHLH
AT1G51140 bHLH
AT1G35460 bHLH
CRE_G2like
AT2G01060 G2like
CRE_TCP3
AT5G08330 TCP
AT5G08070 TCP
AT3G45150 TCP
AT3G27010 TCP
AT3G02150 TCP
AT1G53230 TCP
AT1G30210 TCP
CRE_MADS+2
AT5G13790 MADS
AT3G57230 MADS
AT3G24050 C2C2gata
AT2G45650 MADS
AT2G22540 MADS
CRE_MYB+
AT5G61620 MYBrelated
AT5G58900 MYBrelated
AT5G56840 MYBrelated
AT5G47390 MYBrelated
AT5G08520 MYBrelated
AT5G05790 MYBrelated
AT4G38170 ND
AT4G24020 RWPRK
AT3G21175 C2C2gata
AT3G11280 MYBrelated
AT3G10580 MYBrelated
AT3G06740 C2C2gata
AT2G38090 MYBrelated
AT2G27300 NAC
AT1G74370 C3H
AT1G51600 C2C2gata
AT1G49010 MYBrelated
CRE_TCP2
AT5G23280 TCP
AT3G47620 TCP
AT3G27010 TCP
AT1G72010 TCP
AT1G69690 TCP
AT1G67260 TCP
C-CRE
AT5G65210 bZIP
AT5G11260 bZIP
AT5G10030 bZIP
AT5G06960 bZIP
AT5G06950 bZIP
AT5G06839 bZIP
AT3G12250 bZIP
AT2G31460 REMB3
AT1G77920 bZIP
AT1G22070 bZIP
AT1G19790 SRS
AT1G08320 bZIP
CRE_WRKY+
AT5G66940 C2C2dof
AT5G62940 C2C2dof
AT5G52830 WRKY
AT5G46350 WRKY
AT5G41570 WRKY
AT5G26170 WRKY
AT5G13080 WRKY
AT5G07100 WRKY
AT5G02460 C2C2dof
AT4G38000 C2C2dof
AT4G31800 WRKY
AT4G31550 WRKY
AT4G26640 WRKY
AT4G24240 WRKY
AT4G24020 RWPRK
AT4G23550 WRKY
AT4G18170 WRKY
AT4G14770 CPP
AT4G01250 WRKY
AT3G55370 C2C2dof
AT3G50410 C2C2dof
AT3G47500 C2C2dof
AT3G45610 C2C2dof
AT3G21890 Orphan
AT3G18990 ABI3VP1
AT3G13810 C2H2
AT3G01970 WRKY
AT2G46130 WRKY
AT2G38470 WRKY
AT2G30590 WRKY
AT2G30250 WRKY
AT2G28810 C2C2dof
AT2G24570 WRKY
AT2G23320 WRKY
AT2G21900 WRKY
AT2G17410 ARID
AT2G03340 WRKY
AT2G02070 C2H2
AT2G01940 C2H2
AT1G80840 WRKY
AT1G69570 C2C2dof
AT1G51700 C2C2dof
AT1G49480 REM
AT1G47655 C2C2dof
AT1G30650 WRKY
AT1G29860 WRKY
AT1G29280 WRKY
AT1G29160 C2C2dof
AT1G20910 ARID
AT1G14580 C2H2
CRE_C2C2dof+2
AT5G66940 C2C2dof
AT5G62940 C2C2dof
AT5G62165 MADS
AT5G60850 C2C2dof
AT5G02460 C2C2dof
AT4G30080 ARF
AT3G55370 C2C2dof
AT3G52440 C2C2dof
AT3G50410 C2C2dof
AT3G47500 C2C2dof
AT3G45610 C2C2dof
AT3G21890 Orphan
AT2G37590 C2C2dof
AT2G28810 C2C2dof
AT1G64620 C2C2dof
AT1G51600 C2C2gata
AT1G49480 REM
AT1G47655 C2C2dof

(3)

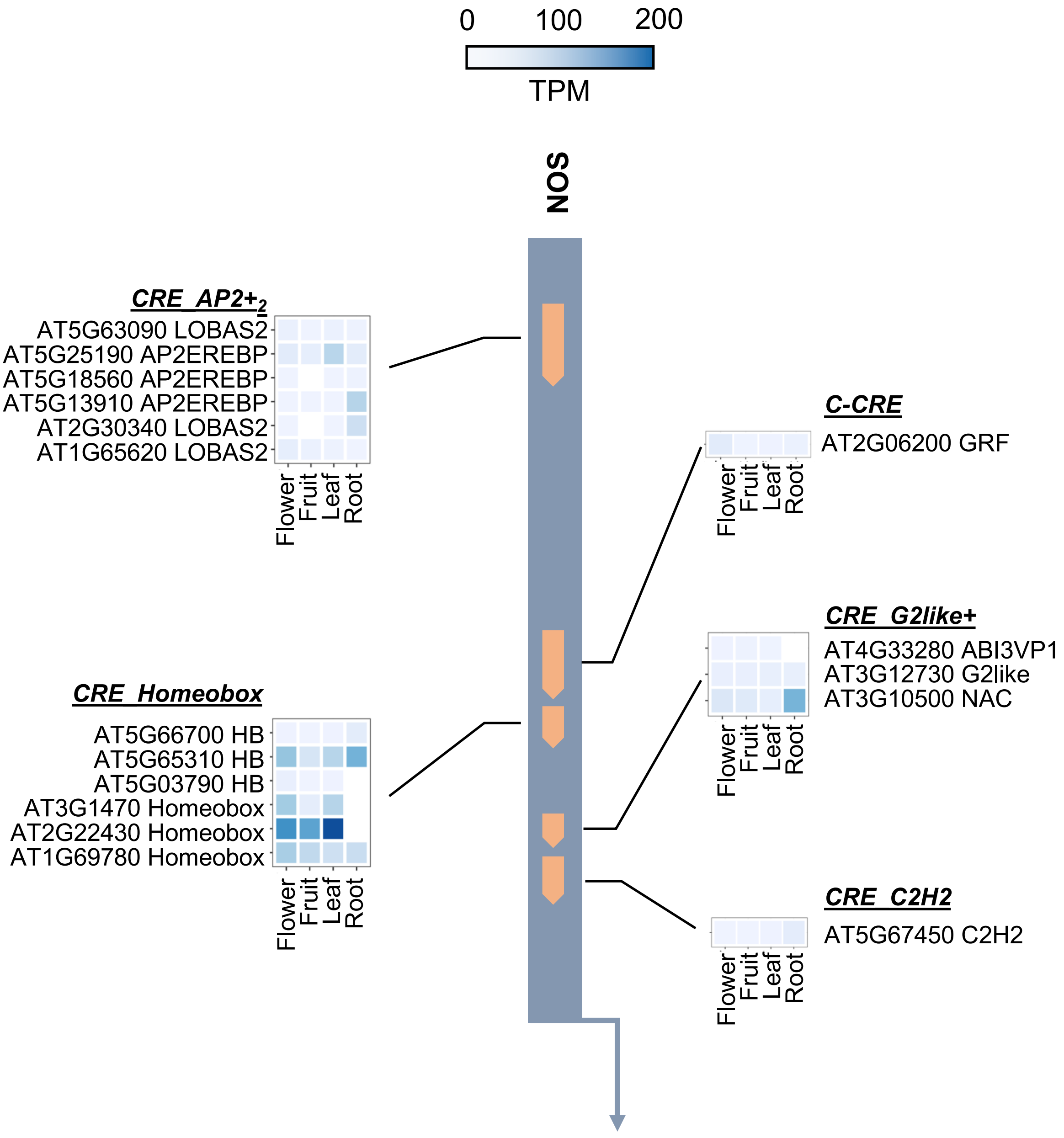
0
100
200
TPM
NOS
CRE_AP2+2
AT5G63090 LOBAS2
AT5G25190 AP2EREBP
AT5G18560 AP2EREBP
AT5G13910 AP2EREBP
AT2G30340 LOBAS2
AT1G65620 LOBAS2
Flower
Fruit
Leaf
Root
C-CRE
AT2G06200 GRF
Flower
Fruit
Leaf
Root
CRE_G2like+
AT4G33280 ABI3VP1
AT3G12730 G2like
AT3G10500 NAC
Flower
Fruit
Leaf
Root
CRE_Homeobox
AT5G66700 HB
AT5G65310 HB
AT5G03790 HB
AT3G1470 Homeobox
AT2G22430 Homeobox
AT1G69780 Homeobox
Flower
Fruit
Leaf
Root
CRE_C2H2
AT5G67450 C2H2
Flower
Fruit
Leaf
Root

(4)

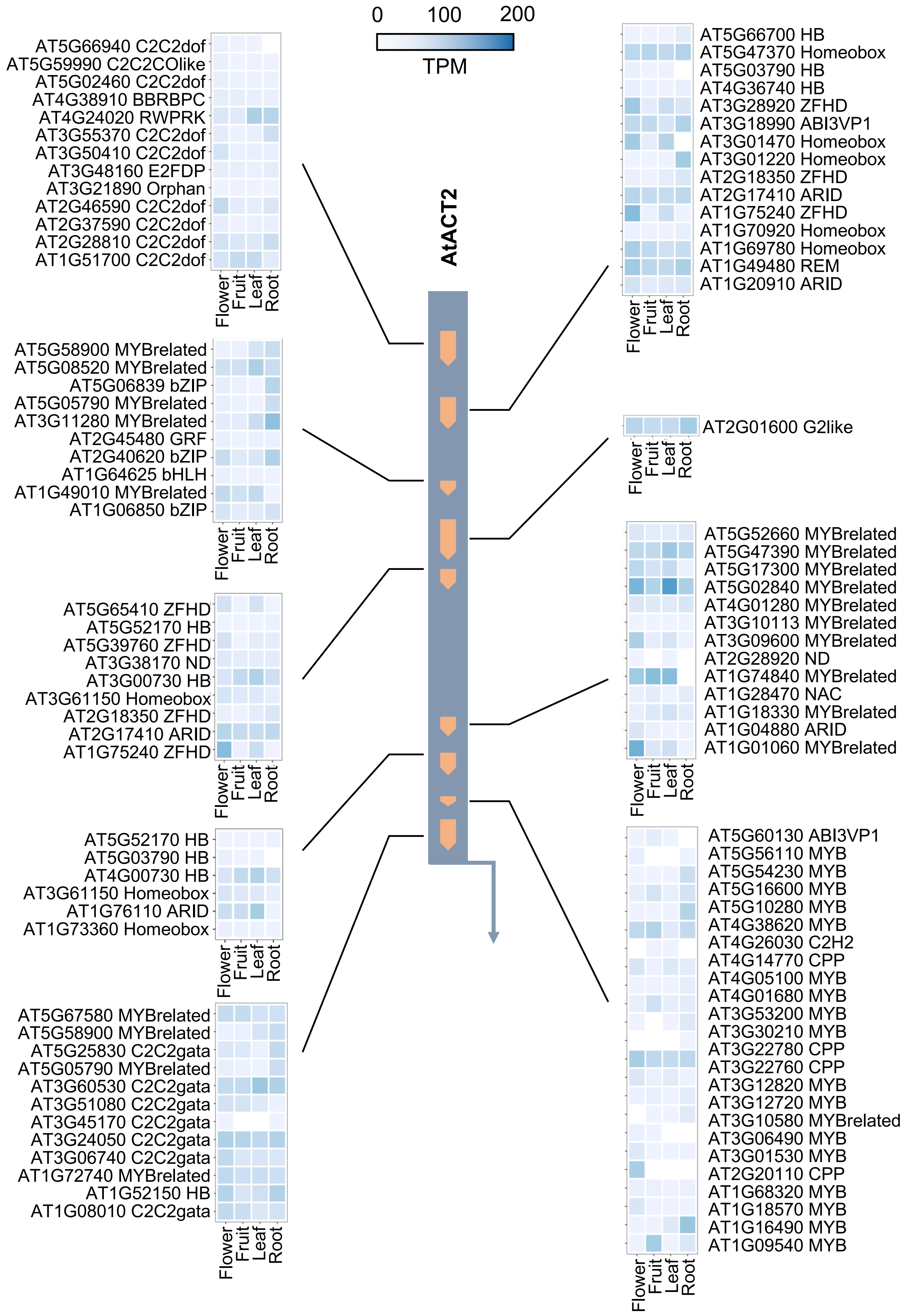
0
100
200
TPM
AtACT2
AT5G66940 C2C2dof
AT5G59990 C2C2COlike
AT5G02460 C2C2dof
AT4G38910 BBRBPC
AT4G24020 RWPRK
AT3G55370 C2C2dof
AT3G50410 C2C2dof
AT3G48160 E2FDP
AT3G21890 Orphan
AT2G46590 C2C2dof
AT2G37590 C2C2dof
AT2G28810 C2C2dof
AT1G51700 C2C2dof
Flower
Fruit
Leaf
Root
AT5G58900 MYBrelated
AT5G08520 MYBrelated
AT5G06839 bZIP
AT5G05790 MYBrelated
AT3G11280 MYBrelated
AT2G45480 GRF
AT2G40620 bZIP
AT1G64625 bHLH
AT1G49010 MYBrelated
AT1G06850 bZIP
AT5G65410 ZFHD
AT5G52170 HB
AT5G39760 ZFHD
AT3G38170 ND
AT3G00730 HB
AT3G61150 Homeobox
AT2G18350 ZFHD
AT2G17410 ARID
AT1G75240 ZFHD
AT5G52170 HB
AT5G03790 HB
AT4G00730 HB
AT3G61150 Homeobox
AT1G76110 ARID
AT1G73360 Homeobox
AT5G67580 MYBrelated
AT5G58900 MYBrelated
AT5G25830 C2C2gata
AT5G05790 MYBrelated
AT3G60530 C2C2gata
AT3G51080 C2C2gata
AT3G45170 C2C2gata
AT3G24050 C2C2gata
AT3G06740 C2C2gata
AT1G72740 MYBrelated
AT1G52150 HB
AT1G08010 C2C2gata
AT5G66700 HB
AT5G47370 Homeobox
AT5G03790 HB
AT4G36740 HB
AT3G28920 ZFHD
AT3G18990 ABI3VP1
AT3G01470 Homeobox
AT3G01220 Homeobox
AT2G18350 ZFHD
AT2G17410 ARID
AT1G75240 ZFHD
AT1G70920 Homeobox
AT1G69780 Homeobox
AT1G49480 REM
AT1G20910 ARID
AT2G01600 G2like
AT5G52660 MYBrelated
AT5G47390 MYBrelated
AT5G17300 MYBrelated
AT5G02840 MYBrelated
AT4G01280 MYBrelated
AT3G10113 MYBrelated
AT3G09600 MYBrelated
AT2G28920 ND
AT1G74840 MYBrelated
AT1G28470 NAC
AT1G18330 MYBrelated
AT1G04880 ARID
AT1G01060 MYBrelated
AT5G60130 ABI3VP1
AT5G56110 MYB
AT5G54230 MYB
AT5G16600 MYB
AT5G10280 MYB
AT4G38620 MYB
AT4G26030 C2H2
AT4G14770 CPP
AT4G05100 MYB
AT4G01680 MYB
AT3G53200 MYB
AT3G30210 MYB
AT3G22780 CPP
AT3G22760 CPP
AT3G12820 MYB
AT3G12720 MYB
AT3G10580 MYBrelated
AT3G06490 MYB
AT3G01530 MYB
AT2G20110 CPP
AT1G68320 MYB
AT1G18570 MYB
AT1G16490 MYB
AT1G09540 MYB

(5)

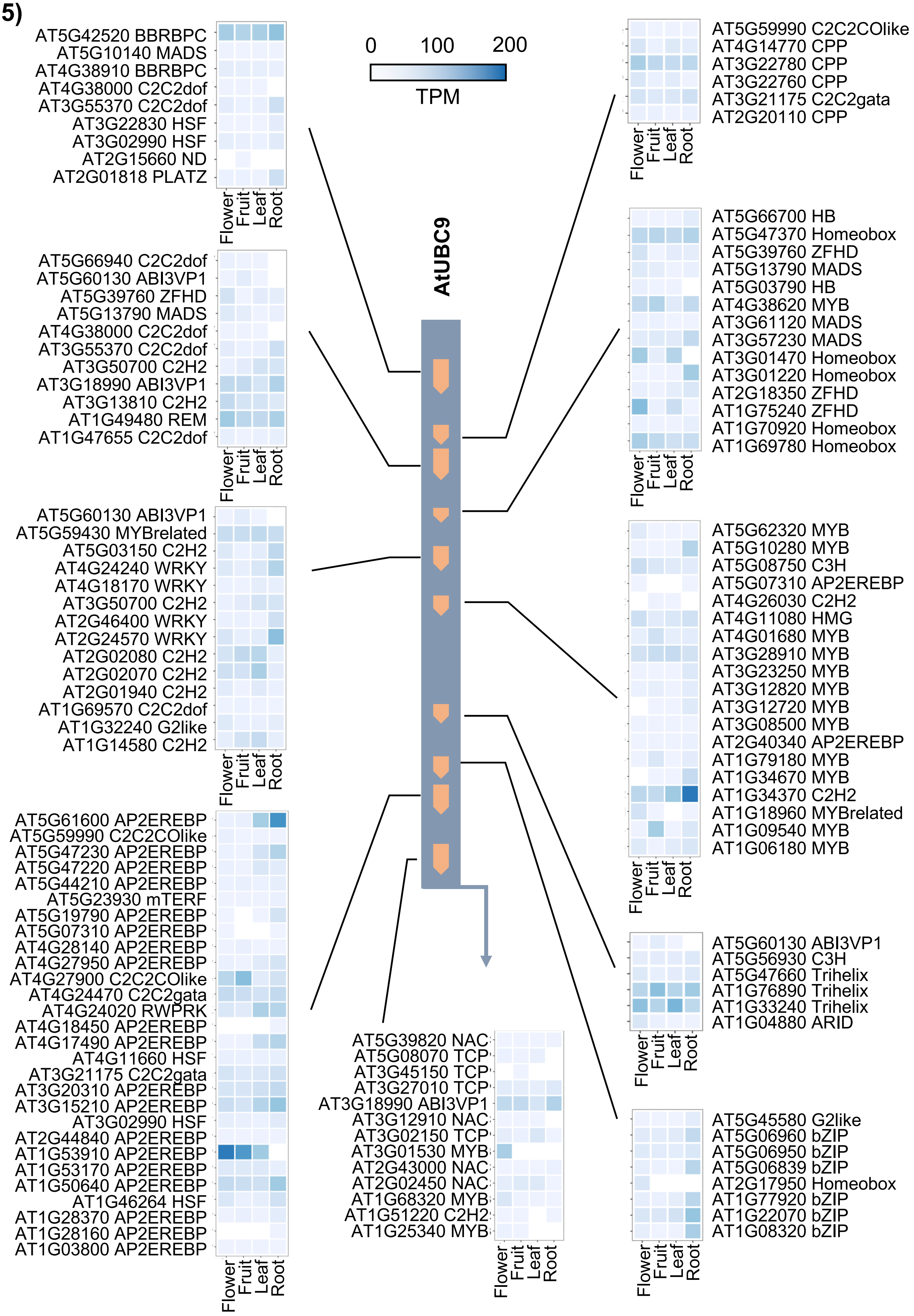

0
100
200
TPM
AtUBC9
AT5G42520 BBRBPC
AT5G10140 MADS
AT4G38910 BBRBPC
AT4G38000 C2C2dof
AT3G55370 C2C2dof
AT3G22830 HSF
AT3G02990 HSF
AT2G15660 ND
AT2G01818 PLATZ
AT5G66940 C2C2dof
AT5G60130 ABI3VP1
AT5G39760 ZFHD
AT5G13790 MADS
AT4G38000 C2C2dof
AT3G55370 C2C2dof
AT3G50700 C2H2
AT3G18990 ABI3VP1
AT3G13810 C2H2
AT1G49480 REM
AT1G47655 C2C2dof
AT5G60130 ABI3VP1
AT5G59430 MYBrelated
AT5G03150 C2H2
AT4G24240 WRKY
AT4G18170 WRKY
AT3G50700 C2H2
AT2G46400 WRKY
AT2G24570 WRKY
AT2G02080 C2H2
AT2G02070 C2H2
AT2G01940 C2H2
AT1G69570 C2C2dof
AT1G32240 G2like
AT1G14580 C2H2
AT5G61600 AP2EREBP
AT5G59990 C2C2COlike
AT5G47230 AP2EREBP
AT5G47220 AP2EREBP
AT5G44210 AP2EREBP
AT5G23930 mTERF
AT5G19790 AP2EREBP
AT5G07310 AP2EREBP
AT4G28140 AP2EREBP
AT4G27950 AP2EREBP
AT4G27900 C2C2COlike
AT4G24470 C2C2gata
AT4G24020 RWPRK
AT4G18450 AP2EREBP
AT4G17490 AP2EREBP
AT4G11660 HSF
AT3G21175 C2C2gata
AT3G20310 AP2EREBP
AT3G15210 AP2EREBP
AT3G02990 HSF
AT2G44840 AP2EREBP
AT1G53910 AP2EREBP
AT1G53170 AP2EREBP
AT1G50640 AP2EREBP
AT1G46264 HSF
AT1G28370 AP2EREBP
AT1G28160 AP2EREBP
AT1G03800 AP2EREBP
AT5G39820 NAC
AT5G08070 TCP
AT3G45150 TCP
AT3G27010 TCP
AT3G18990 ABI3VP1
AT3G12910 NAC
AT3G02150 TCP
AT3G01530 MYB
AT2G43000 NAC
AT2G02450 NAC
AT1G68320 MYB
AT1G51220 C2H2
AT1G25340 MYB
AT5G59990 C2C2COlike
AT4G14770 CPP
AT3G22780 CPP
AT3G22760 CPP
AT3G21175 C2C2gata
AT2G20110 CPP
AT5G66700 HB
AT5G47370 Homeobox
AT5G39760 ZFHD
AT5G13790 MADS
AT5G03790 HB
AT4G38620 MYB
AT3G61120 MADS
AT3G57230 MADS
AT3G01470 Homeobox
AT3G01220 Homeobox
AT2G18350 ZFHD
AT1G75240 ZFHD
AT1G70920 Homeobox
AT1G69780 Homeobox
AT5G62320 MYB
AT5G10280 MYB
AT5G08750 C3H
AT5G07310 AP2EREBP
AT4G26030 C2H2
AT4G11080 HMG
AT4G01680 MYB
AT3G28910 MYB
AT3G23250 MYB
AT3G12820 MYB
AT3G12720 MYB
AT3G08500 MYB
AT2G40340 AP2EREBP
AT1G79180 MYB
AT1G34670 MYB
AT1G34370 C2H2
AT1G18960 MYBrelated
AT1G09540 MYB
AT1G06180 MYB
AT5G60130 ABI3VP1
AT5G56930 C3H
AT5G47660 Trihelix
AT1G76890 Trihelix
AT1G33240 Trihelix
AT1G04880 ARID
AT5G45580 G2like
AT5G06960 bZIP
AT5G06950 bZIP
AT5G06839 bZIP
AT2G17950 Homeobox
AT1G77920 bZIP
AT1G22070 bZIP
AT1G08320 bZIP
Flower
Fruit
Leaf
Root

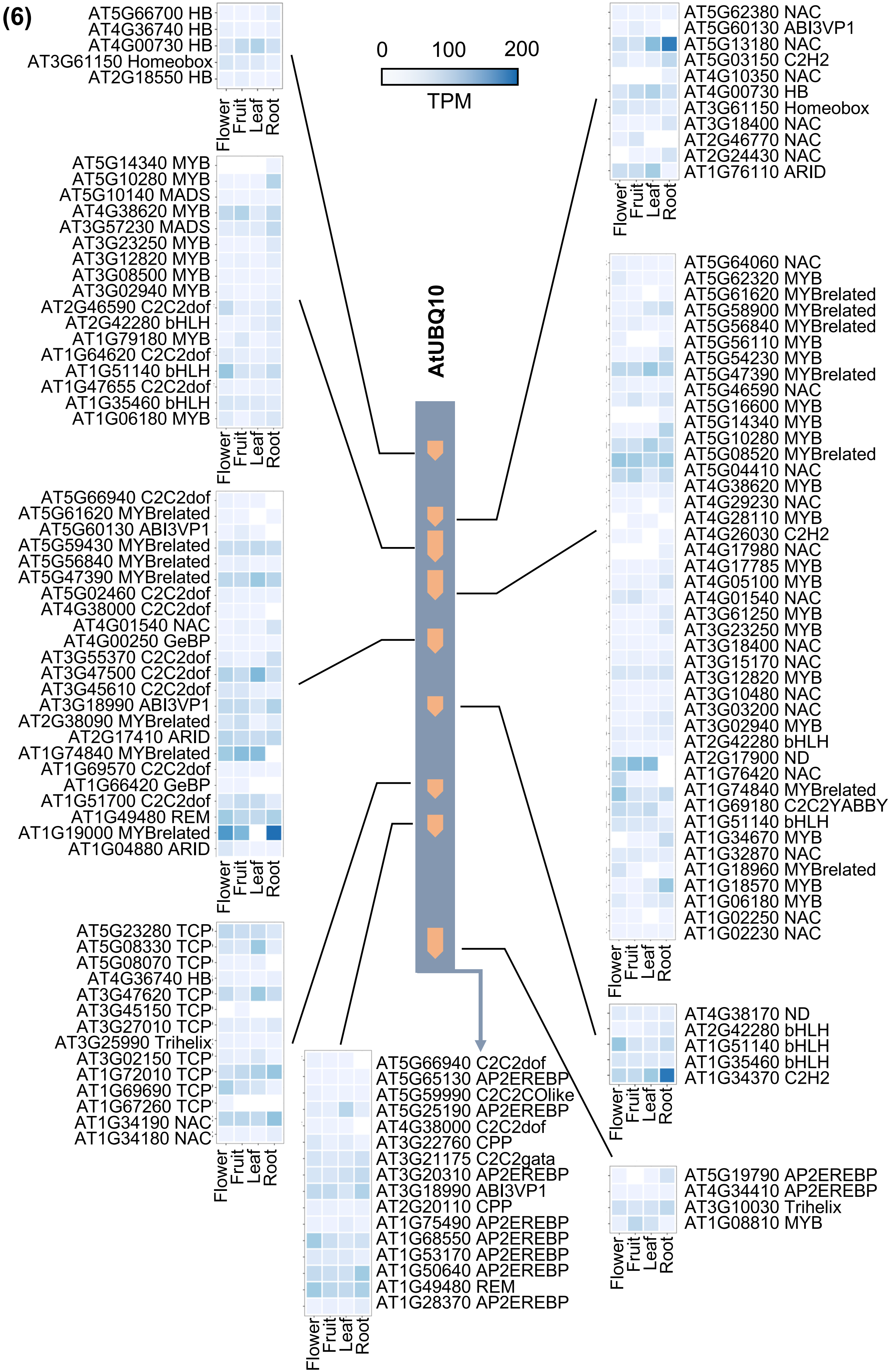

(6)
AtUBQ10
0
100
200
TPM
AT5G66700 HB
AT4G36740 HB
AT4G00730 HB
AT3G61150 Homeobox
AT2G18550 HB
AT5G14340 MYB
AT5G10280 MYB
AT5G10140 MADS
AT4G38620 MYB
AT3G57230 MADS
AT3G23250 MYB
AT3G12820 MYB
AT3G08500 MYB
AT3G02940 MYB
AT2G46590 C2C2dof
AT2G42280 bHLH
AT1G79180 MYB
AT1G64620 C2C2dof
AT1G51140 bHLH
AT1G47655 C2C2dof
AT1G35460 bHLH
AT1G06180 MYB
AT5G66940 C2C2dof
AT5G61620 MYBrelated
AT5G60130 ABI3VP1
AT5G59430 MYBrelated
AT5G56840 MYBrelated
AT5G47390 MYBrelated
AT5G02460 C2C2dof
AT4G38000 C2C2dof
AT4G01540 NAC
AT4G00250 GeBP
AT3G55370 C2C2dof
AT3G47500 C2C2dof
AT3G45610 C2C2dof
AT3G18990 ABI3VP1
AT2G38090 MYBrelated
AT2G17410 ARID
AT1G74840 MYBrelated
AT1G69570 C2C2dof
AT1G66420 GeBP
AT1G51700 C2C2dof
AT1G49480 REM
AT1G19000 MYBrelated
AT1G04880 ARID
AT5G23280 TCP
AT5G08330 TCP
AT5G08070 TCP
AT4G36740 HB
AT3G47620 TCP
AT3G45150 TCP
AT3G27010 TCP
AT3G25990 Trihelix
AT3G02150 TCP
AT1G72010 TCP
AT1G69690 TCP
AT1G67260 TCP
AT1G34190 NAC
AT1G34180 NAC
AT5G66940 C2C2dof
AT5G65130 AP2EREBP
AT5G59990 C2C2COlike
AT5G25190 AP2EREBP
AT4G38000 C2C2dof
AT3G22760 CPP
AT3G21175 C2C2gata
AT3G20310 AP2EREBP
AT3G18990 ABI3VP1
AT2G20110 CPP
AT1G75490 AP2EREBP
AT1G68550 AP2EREBP
AT1G53170 AP2EREBP
AT1G50640 AP2EREBP
AT1G49480 REM
AT1G28370 AP2EREBP
AT5G62380 NAC
AT5G60130 ABI3VP1
AT5G13180 NAC
AT5G03150 C2H2
AT4G10350 NAC
AT4G00730 HB
AT3G61150 Homeobox
AT3G18400 NAC
AT2G46770 NAC
AT2G24430 NAC
AT1G76110 ARID
AT5G64060 NAC
AT5G62320 MYB
AT5G61620 MYBrelated
AT5G58900 MYBrelated
AT5G56840 MYBrelated
AT5G56110 MYB
AT5G54230 MYB
AT5G47390 MYBrelated
AT5G46590 NAC
AT5G16600 MYB
AT5G14340 MYB
AT5G10280 MYB
AT5G08520 MYBrelated
AT5G04410 NAC
AT4G38620 MYB
AT4G29230 NAC
AT4G28110 MYB
AT4G26030 C2H2
AT4G17980 NAC
AT4G17785 MYB
AT4G05100 MYB
AT4G01540 NAC
AT3G61250 MYB
AT3G23250 MYB
AT3G18400 NAC
AT3G15170 NAC
AT3G12820 MYB
AT3G10480 NAC
AT3G03200 NAC
AT3G02940 MYB
AT2G42280 bHLH
AT2G17900 ND
AT1G76420 NAC
AT1G74840 MYBrelated
AT1G69180 C2C2YABBY
AT1G51140 bHLH
AT1G34670 MYB
AT1G32870 NAC
AT1G18960 MYBrelated
AT1G18570 MYB
AT1G06180 MYB
AT1G02250 NAC
AT1G02230 NAC
AT4G38170 ND
AT2G42280 bHLH
AT1G51140 bHLH
AT1G35460 bHLH
AT1G34370 C2H2
AT5G19790 AP2EREBP
AT4G34410 AP2EREBP
AT3G10030 Trihelix
AT1G08810 MYB
Flower
Fruit
Leaf
Root

Supplementary Data File 4

Multiple sequence alignment of a *cis*-regulatory element common to all 14 promoters from plant-infecting viruses and bacteria. The red box highlights consensus binding motifs for the TGACG-motif binding (TGA) basic-leucine zipper (B-ZIP) transcription factors.

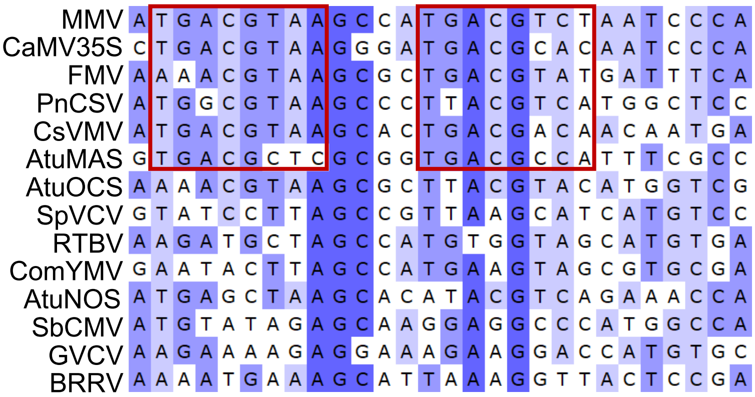

Supplementary Data File 5

Deletion of many candidate *cis*-regulatory elements (CRE) had no significant impact on expression. Error bars = 2 x standard error; P-values were calculated using unpaired two-tailed Student's t-test; *P<0.05, **P< 0.01, ***P< 0.001; n=3

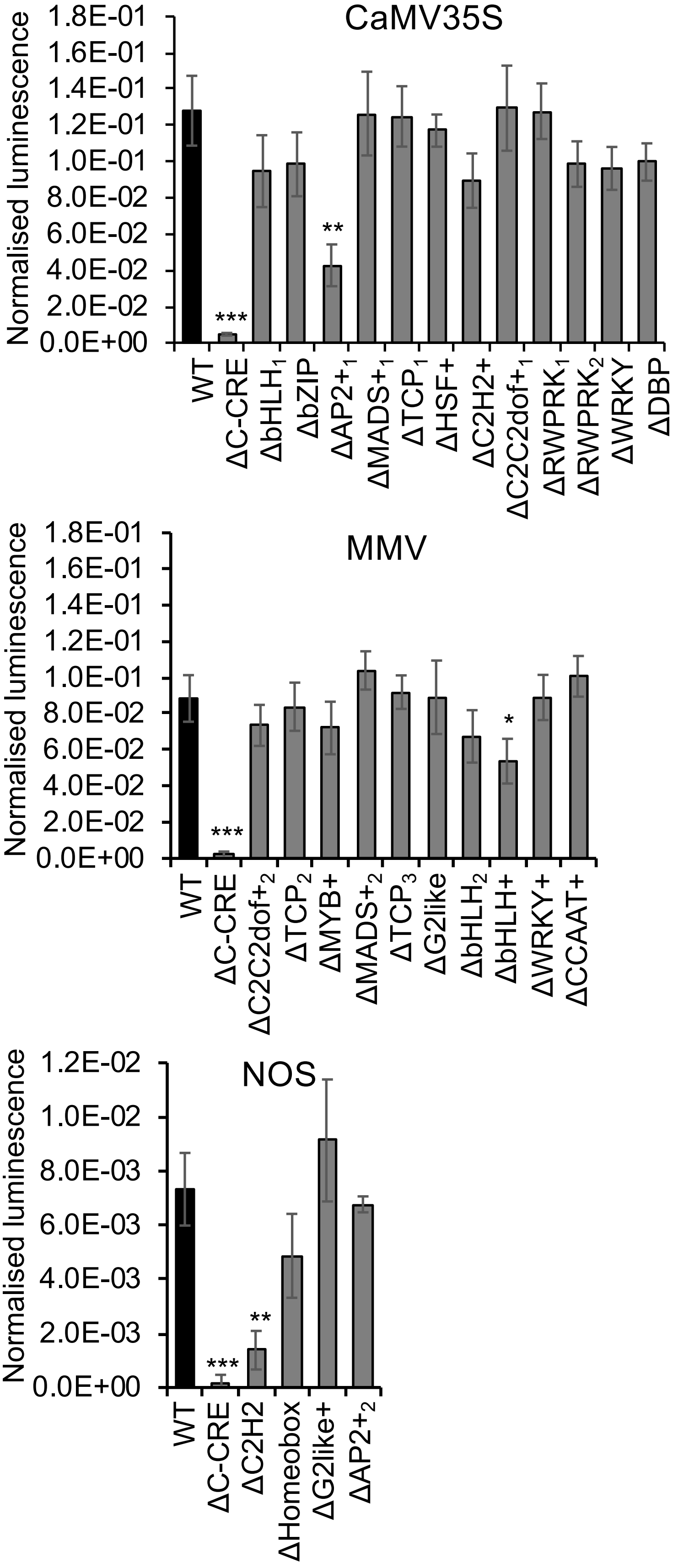

Supplementary Data 6

Combining certain *cis*-regulatory elements (CREs) into the variable regions of MinSyns did not result in significant expression

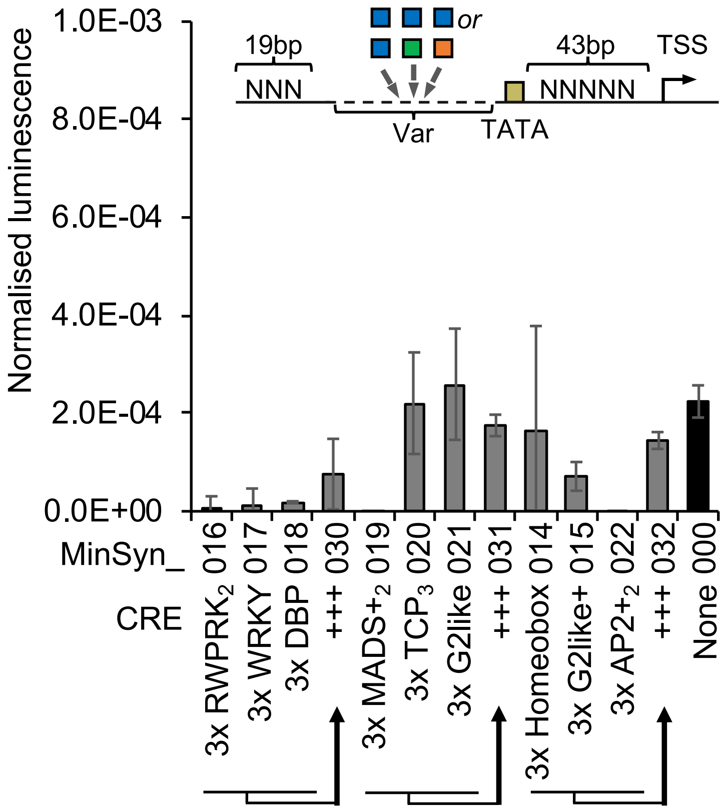

Supplementary Data 7. Sequences and predicted strengths of all computationally designed Minimal Synthetic Promoters (MinSyns)

>MinSyn_1000|Strength:0.001329763
GCGTGTCGTTTTAGTGAGGTCAGAAGATCAAAGGGCTATAGGAGGACCGATGCTGATCTTGCCTGCCA
GCCACTTGTGTCTGCTAGGAGCACACCAGCATGTGTTGATCACCAGCTGGCTCACTATATAAGGTTTT
GCTATTCATTGAAAGCAGTAGTGACTGATTTGTATATA
>MinSyn_1161|Strength:0.000223739
GCGTGTCGTTTTAGTGAGGTCAGAAGATCAAAGGGCTACACTATGCCCAATTAGGTTGTCTGCAAGCA
AGTGGATGGCTATCAGCTTAGCAAGACCTCTACAACTATATAAGGTTTTGCTATTCATTGAAAGCAGT
AGTGACTGATTTGTATATA
>MinSyn_1970|Strength:0.0003525
GCGTGTCGTTTTAGTGAGGGCTTTGTCAAAAGCTAAAAAAGATGATGCGTCTGCACCTCACATGTAGG
CTATCAGCTTCACTATCAGCCTTGTGTACAGGGCTCACTGCTATATAAGGTTTTGCTATTCATTGAAA
GCAGTAGTGACTGATTTGTATATA
>MinSyn_1951|Strength:0.000375771
GCGTGTCGTTTTAGTGAGGAATTTCGGGAAACCTCCTCGTCTACAAAACTGGTACTTTTTCAACAAGC
TTAGCAAGACCTCAAATATTTCTTGTGGTACTTGTGTACAGGGCTCACTGCTGCTTTGTCAAAAGCTA
AAAAAGATGATGCCTTGTGTACAGGGCTCACTCTATATAAGGTTTTGCTATTCATTGAAAGCAGTAGT
GACTGATTTGTATATA
>MinSyn_1490|Strength:0.000396386
GCGTGTCGTTTTAGTGAGGGCTTTGTCAAAAGCTAAAAAAGATGATGCGCACCTCACATGTAGGCTAT
CAGCTTAGGTGGCTCCTACGATCTTGCCAGCAAGTGGATGCGGTAGCTATATAAGGTTTTGCTATTCA
TTGAAAGCAGTAGTGACTGATTTGTATATA
>MinSyn_1225|Strength:0.000430833
GCGTGTCGTTTTAGTGAGGAGCAAGTGGATGCTTAGCAAGACCTCTACAAAACTTTTTCAACAAGCAA
CTATATAAGGTTTTGCTATTCATTGAAAGCAGTAGTGACTGATTTGTATATA
>MinSyn_1108|Strength:0.000437209
GCGTGTCGTTTTAGTGAGGTCACTATCAGCCTCACATGTAGGCTAGCAAGTGGATGGAGGACCGATGC
TGATCTTGCCTATATAAGGTTTTGCTATTCATTGAAAGCAGTAGTGACTGATTTGTATATA
>MinSyn_1653|Strength:0.000456944
GCGTGTCGTTTTAGTGAGGGCTTTGTCAAAAGCTAAAAAAGATGATGCATGTAGGCTATCAGCTAACC
ATTATTGCGCTCTACAAAACTGGTCTATATAAGGTTTTGCTATTCATTGAAAGCAGTAGTGACTGATT
TGTATATA
>MinSyn_1138|Strength:0.00047
GCGTGTCGTTTTAGTGAGGCAGCCACTTGTGTAGGAGGACCGATGCTGATCTTGCTTTGTCAAAAGCT
AAAAAAGATGATGCACATGTCTATATAAGGTTTTGCTATTCATTGAAAGCAGTAGTGACTGATTTGTA
TATA
>MinSyn_1093|Strength:0.000487407
GCGTGTCGTTTTAGTGAGGGCTTTGTCAAAAGCTAAAAAAGATGATGCCTGGTACTTGTGTACAGGGT
GAAGCATCTTCCGTCCTATATAAGGTTTTGCTATTCATTGAAAGCAGTAGTGACTGATTTGTATATA
>MinSyn_1141|Strength:0.000491045
GCGTGTCGTTTTAGTGAGGAGCAAGTGGATACTGCTAGGACAGCCACTTGTGTTATCAGCTTAGCAAG
ACCTCTACAAAACTCTATATAAGGTTTTGCTATTCATTGAAAGCAGTAGTGACTGATTTGTATATA
>MinSyn_1944|Strength:0.000491291
GCGTGTCGTTTTAGTGAGGAATTTCGGGAAACCTCCTCGGTCTGCACCTCAGCAAGTGGATTCGCGGT
AGGAAAAAGAAGAGGTTATGCCCAATTAGGTTGTCTGCACCTCAGCTTTGTCAAAAGCTAAAAAAGAT
GATGCGGAGGACCGATGCTGATCTTGCCTGCCTTGATCGAAAGGACAGTAGCTATCAGCTTAGCAAGC
TATATAAGGTTTTGCTATTCATTGAAAGCAGTAGTGACTGATTTGTATATA
>MinSyn_1994|Strength:0.000494737
GCGTGTCGTTTTAGTGAGGAGCAAGTGGATATGTAGGCTATCAGCTAGGTGGCTCCTACACTATGCCC
AATTAGGTTGTCTCTATATAAGGTTTTGCTATTCATTGAAAGCAGTAGTGACTGATTTGTATATA
>MinSyn_1333|Strength:0.000499115
GCGTGTCGTTTTAGTGAGGAGCAAGTGGATCTCACTATCAGCTCTTGCCTGCCTTGATGATCTATATA

AGGTTTTGCTATTCATTGAAAGCAGTAGTGACTGATTTGTATATA
>MinSyn_1463|Strength:0.000500764
GCGTGTCGTTTTAGTGAGGTTATGACCCCCGCCGATGACGCGGGACGCGGTAGGTCACGACCACTATG
CCCTTTTCAACAAGGTTGTCTGCACCTCAGCTTTGTCAAAAGCTAAAAAAGATGATGCTCAGCAAGTG
GATGAGGACCGATGCTGATCTGGTGGAGCACGACACAAAACTGGTACTTGTGTACAGGGCTCCTATAT
AAGGTTTTGCTATTCATTGAAAGCAGTAGTGACTGATTTGTATATA
>MinSyn_1474|Strength:0.000522222
GCGTGTCGTTTTAGTGAGGGCTTTGTCAAAAGCTAAAAAAGATGATGCTCTTGCCTGCCTCAGCCACT
TGTGTTCTATATAAGGTTTTGCTATTCATTGAAAGCAGTAGTGACTGATTTGTATATA
>MinSyn_1109|Strength:0.000528434
GCGTGTCGTTTTAGTGAGGTTTTCAACAACCTGCCTTGAACCATTATTGCGGGACCGATTCCATCAAC
AAATAATCCAAGTAAGACCAGCCACTTGTGTGACCACTATGCCCAATTAGGAGCAAGTGGATCTACAA
AACTGGTACTGCTTTGTCAAAAGCTAAAAAAGATGATGCTAGCTATATAAGGTTTTGCTATTCATTGA
AAGCAGTAGTGACTGATTTGTATATA
>MinSyn_1971|Strength:0.000548905
GCGTGTCGTTTTAGTGAGGCAGTGGTCCCTCCACTATGCCCAATAGCAAGTGGATAGGTTGTCTGCAC
CTCACATGTAGGCTATCCTATATAAGGTTTTGCTATTCATTGAAAGCAGTAGTGACTGATTTGTATAT
A
>MinSyn_1992|Strength:0.000553661
GCGTGTCGTTTTAGTGAGGTCCATCAACAAATAATCCAAGTAAGTTGTCTGCACCTCACATGTGCTTT
GTCAAAAGCTAAAAAAGATGATGCCGCGGTAGGTCACGACCACTATGCCCAATTGGTGGAGCACGACA
TACAAAACTGGTACTATATAAGGTTTTGCTATTCATTGAAAGCAGTAGTGACTGATTTGTATATA
>MinSyn_1293|Strength:0.000582789
GCGTGTCGTTTTAGTGAGGTCCATCAACAAATAATCCAAGTAAGATTAGGTTGAACCATTATTGCGTG
ATCTTGGCACACCAGCATGTGTTGATCACCAGCTAGGTCACGACCACTATGCCCAATTGCTTTGTCAA
AAGCTAAAAAAGATGATGCCCTCTACAAAACTGGTACTTGTCTATATAAGGTTTTGCTATTCATTGAA
AGCAGTAGTGACTGATTTGTATATA
>MinSyn_1468|Strength:0.0005875
GCGTGTCGTTTTAGTGAGGAGCAAGTGGATGACCTCTACAAAACTGGTACTTGTGGGAAAAAGAAGAG
GTAGGGCTCTATATAAGGTTTTGCTATTCATTGAAAGCAGTAGTGACTGATTTGTATATA
>MinSyn_1511|Strength:0.000590545
GCGTGTCGTTTTAGTGAGGGCACACCAGCATGTGTTGATCACCAGCTGCTGATCTTGCCTGCCTTGAC
AAATATTTCTTGTCTTAGCAAGACCTCTAGCTTTGTCAAAAGCTAAAAAAGATGATGCCTCACTGCAA
CCATTATTGCGAGGTTGTCTGCACCTCACATGTAGGCTATCTATATAAGGTTTTGCTATTCATTGAAA
GCAGTAGTGACTGATTTGTATATA
>MinSyn_1655|Strength:0.000592123
GCGTGTCGTTTTAGTGAGGTTTTCAACAACTTAGCAAGACCTCTACAAAACTGGTACTAACCACGTCT
ACAAAAACTGGTACTTGTGTACAGGGCTATATAAGGTTTTGCTATTCATTGAAAGCAGTAGTGACTGA
TTTGTATATA
>MinSyn_1053|Strength:0.000592396
GCGTGTCGTTTTAGTGAGGGCACACCAGCATGTGTTGATCACCAGCTTGTTATGACCCCCGCCGATGA
CGCGGGACTGGTACGCTTTGTCAAAAGCTAAAAAAGATGATGCTAGGTCACGACCACTATGCCCAATT
AGGTCTATATAAGGTTTTGCTATTCATTGAAAGCAGTAGTGACTGATTTGTATATA
>MinSyn_1232|Strength:0.000601829
GCGTGTCGTTTTAGTGAGGAACCATTATTGCGCCACTATGCCCAATTAGGTTGTCAAAAATGTCAAAG
ATATGATAAGCAAGTGGATCCCAATTAGGTTGTCTGCACCTCACCTATATAAGGTTTTGCTATTCATT
GAAAGCAGTAGTGACTGATTTGTATATA
>MinSyn_1677|Strength:0.000611834
GCGTGTCGTTTTAGTGAGGTGAAGCATCTTCCTCACTGCTAGGAGGACCGATGCTGATCTTGTGGTGG
AGCACGACAGGACCGATGCTGATCTTGCCTGCCTTGCTATATAAGGTTTTGCTATTCATTGAAAGCAG
TAGTGACTGATTTGTATATA
>MinSyn_1526|Strength:0.000638568
GCGTGTCGTTTTAGTGAGGTGAAGCATCTTCCGAGGACCGATGCTGATCTTGCCTGCCAAAAATGTCA
AAGATATAGGCTATCAGCTTAGCAAGACCTCTACAAAACCACGTCTACAAAGACCTCTACAAAACTGG

TACTTGTGTCTATATAAGGTTTTGCTATTCATTGAAAGCAGTAGTGACTGATTTGTATATA
>MinSyn_1142|Strength:0.000639456
GCGTGTCGTTTTAGTGAGGTCAGAAGATCAAAGGGCTAACAGGGCTCACTGCTAGGAGGACGCTTTGT
CAAAAGCTAAAAAAGATGATGCTTGATCTATATAAGGTTTTGCTATTCATTGAAAGCAGTAGTGACTG
ATTTGTATATA
>MinSyn_1899|Strength:0.000647599
GCGTGTCGTTTTAGTGAGGTCACTATCAGCTTAGCAAGACCTCTACAAAACTGGTACTTGCAGTGGTC
CCTCCACGAGGACCGATGCTGATCTTGCAGCAAGTGGATTAGGTCACGACCACTATGCCCAATTATGA
AGCATCTTCCCTTGTGTACAGGGCTCACTGCGCTTTGTCAAAAGCTAAAAAAGATGATGCTAGGTCTT
ATGACCCCCGCCGATGACGCGGGAGCCCAATTAGGTTGTCTGCACCTATATAAGGTTTTGCTATTCAT
TGAAAGCAGTAGTGACTGATTTGTATATA
>MinSyn_1404|Strength:0.000648276
GCGTGTCGTTTTAGTGAGGTGGTGGAGCACGACACCTCTACAAAACTGGTAGCAAGTGGATGCTCTAT
ATAAGGTTTTGCTATTCATTGAAAGCAGTAGTGACTGATTTGTATATA
>MinSyn_1967|Strength:0.000651825
GCGTGTCGTTTTAGTGAGGATTGCGATAAAGGAAAGGCTTGCCGCTTTGTCAAAAGCTAAAAAAGATG
ATGCAGGTCACGACCACCTATATAAGGTTTTGCTATTCATTGAAAGCAGTAGTGACTGATTTGTATAT
A
>MinSyn_1294|Strength:0.00065369
GCGTGTCGTTTTAGTGAGGGTGGGAGCCACCAAAACTGGTACTTGTGTACAGGGCTGGTGGAGCACGA
CAGCTATCAGCTTAGCAAGACCTCTACAAAGCTTTGTCAAAAGCTAAAAAAGATGATGCCAATTAGGT
TGTCTGCACCTCACATGTAGGGCACACCAGCATGTGTTGATCACCAGCTGCTATCAGCTTAGCAAGAA
GCAAGTGGATAAAACTGCAGTGGTCCCTCCACGTCACGACCACTATGCCCAATCTATATAAGGTTTTG
CTATTCATTGAAAGCAGTAGTGACTGATTTGTATATA
>MinSyn_1559|Strength:0.000657906
GCGTGTCGTTTTAGTGAGGCAGCCACTTGTGTCACGACCACTATGCCCAATTTCAGAAGATCAAAGGG
CTACTACAAAACTGGTACTTAGCAAGTGGATTCTGCACCTCACATGTAGGCTATATAAGGTTTTGCTA
TTCATTGAAAGCAGTAGTGACTGATTTGTATATA
>MinSyn_1242|Strength:0.000660391
GCGTGTCGTTTTAGTGAGGATGAGCTAAGCACATACGTCAGCCTCACATGAAAAATGTCAAAGATATT
GTCTGCACCTCACATGTACAAATATTTCTTGTCTCACATGTAAGATTGATGAAAAGTCAAAAACAAAA
ATCAATTATTGCTGATCTTAGCAAGTGGATCTGCACCTCACATGTAGGCTATCATTTTCAACAACACT
GCTAGGAGGACCGATGCTGCTTTGTCAAAAGCTAAAAAAGATGATGCTAGGAGAACCATTATTGCGGG
TTGTCTGCACCTCACATGTAGGCTATCAAGGTGGCTCCTACTGTACAGGGCTCACTGCTAGGAGGACC
CTATATAAGGTTTTGCTATTCATTGAAAGCAGTAGTGACTGATTTGTATATA
>MinSyn_1451|Strength:0.000668493
GCGTGTCGTTTTAGTGAGGAACCATTATTGCGTAGGTTGTGGTGGAGCACGACAGCTAGGAGGACCGA
TGCTGATCTTGAGCAAGTGGATTGATCTATATAAGGTTTTGCTATTCATTGAAAGCAGTAGTGACTGA
TTTGTATATA
>MinSyn_1068|Strength:0.000673512
GCGTGTCGTTTTAGTGAGGTCAGAAGATCAAAGGGCTAAGGGCTCACTGCTCACTATCAGCAACTGGT
ACTTGTGTACAGGGCTCACTGCTATTGCGATAAAGGAAAGGGTGCTTTGTCAAAAGCTAAAAAAGATG
ATGCTGCTGATCTTCTATATAAGGTTTTGCTATTCATTGAAAGCAGTAGTGACTGATTTGTATATA
>MinSyn_1039|Strength:0.000680398
GCGTGTCGTTTTAGTGAGGAAGATTGATGAAAAGTCAAAAACAAAAATCAATTATTCACATGTAGGCT
CCATCAACAAATAATCCAAGTAAGGGAGCTTTGTCAAAAGCTAAAAAAGATGATGCAAGACCTCTACA
AAACTGGTACTTGTGTATCACTATCAGCCCGATGCTGATCTTGCCTGCCTTGATGCTATATAAGGTTT
TGCTATTCATTGAAAGCAGTAGTGACTGATTTGTATATA
>MinSyn_1987|Strength:0.000681828
GCGTGTCGTTTTAGTGAGGAACCACGTCTACAACAGCTTAGCAAGACCTCTAGTGGGAGCCACCACTT
AGCAAGACCTCTACAAAAATTTCGGGAAACCTCCTCGCTTAGCAAGAGGTGGCTCCTACCCCAATTAT
TTTCAACAATAGCAAGGCTTTGTCAAAAGCTAAAAAAGATGATGCGACCGATGCTGATCTTGCCTGCC
TCTATATAAGGTTTTGCTATTCATTGAAAGCAGTAGTGACTGATTTGTATATA
>MinSyn_1055|Strength:0.000684232

GCGTGTCGTTTTAGTGAGGAACCATTATTGCGTAGGCTATCATCCTTACCGCTATGGGTAAGATTCTG
CTAGGAGGACCGATGTGGTGGAGCACGACACTGCTAGGAGGACCGTGGGAGCCACCAAGGAGGACCGA
AAAATGTCAAAGATAGCCCAATTAGGTTCAGCCACTTGTGTATCAGCTTAGCAGCTTTGTCAAAAGCT
AAAAAAGATGATGCCACTGCTAGGAGGACCGATGCTGATCTATATAAGGTTTTGCTATTCATTGAAAG
CAGTAGTGACTGATTTGTATATA
>MinSyn_1316|Strength:0.000691622
GCGTGTCGTTTTAGTGAGGTCCATCAACAAATAATCCAAGTAAGCTCACATGTAGGCTAATCCTTACC
GCTATGGGTAAGATTCATTGCGATAAAGGAAAGGGCTATCAGCTTAGCAAGACCAGCAAGTGGATTAC
TTGTGTACAGGGCTCACTGCTAGGAGGCTATATAAGGTTTTGCTATTCATTGAAAGCAGTAGTGACTG
ATTTGTATATA
>MinSyn_1214|Strength:0.000693173
GCGTGTCGTTTTAGTGAGGCAAATATTTCTTGTGGTAGTGGTGGAGCACGACATGCTACAGCCACTTG
TGTGGACCGATGCTGATCTTGCCTGCCTTGATTGCGATAAAGGAAAGGAGGACCGATGCTGATTATGA
CCCCCGCCGATGACGCGGGACACCTCACATGTAGGCTATCAGCTTGCTTTGTCAAAAGCTAAAAAAGA
TGATGCGACCGATGCCTATATAAGGTTTTGCTATTCATTGAAAGCAGTAGTGACTGATTTGTATATA
>MinSyn_1284|Strength:0.000693445
GCGTGTCGTTTTAGTGAGGAAAAATGTCAAAGATATGCTGATCTCCATCAACAAATAATCCAAGTAAG
ACCTCACATGTAGGCTGCACACCAGCATGTGTTGATCACCAGCTGCTATTGCGATAAAGGAAAGGGGA
GGACCGATGCTGATCTTTTTTCAACAACTGGTACTTGTGTACAGGGCTCACTATCGAAAGGACAGTAT
AGCAAGACCTCTACAAAAGCTTTGTCAAAAGCTAAAAAAGATGATGCCTGCACCTCACATGTAGGCTA
TCCTATATAAGGTTTTGCTATTCATTGAAAGCAGTAGTGACTGATTTGTATATA
>MinSyn_1957|Strength:0.000707062
GCGTGTCGTTTTAGTGAGGAAAAATGTCAAAGATACAGGGCTCACTGCTAGGAGTTATGACCCCCGCC
GATGACGCGGGACTATGCAGCCACTTGTGTCAAAACTGGTACTTGTGTACAAAGATTGATGAAAAGTC
AAAAACAAAAATCAATTATTTGTCTGCACCTCACATGTAGGCTAGCTTTGTCAAAAGCTAAAAAAGAT
GATGCGTCACGACCCAAATATTTCTTGTAACTGGTACTTAGCAAGTGGATAACTGGTACTTGTGTACA
GGGCTCACTCTATATAAGGTTTTGCTATTCATTGAAAGCAGTAGTGACTGATTTGTATATA
>MinSyn_1050|Strength:0.000707788
GCGTGTCGTTTTAGTGAGGAATTTCGGGAAACCTCCTCGATTAGGTTGTCTGCACCTCATCACTATCA
GCCACATGTAGGCTATCAGCTTAGCAAGACCTGAAGCATCTTCCGATCTTGCCTGCCTTGATGACAGT
GGTCCCTCCACCTGCACCTCACATGTAGGCTATCAGCTTACTATATAAGGTTTTGCTATTCATTGAAA
GCAGTAGTGACTGATTTGTATATA
>MinSyn_1433|Strength:0.000710072
GCGTGTCGTTTTAGTGAGGAGCAAGTGGATTACAGGGCTCACTGCTAGGAGGACCGATGCAATTTCGG
GAAACCTCCTCGAGGACCGCTATATAAGGTTTTGCTATTCATTGAAAGCAGTAGTGACTGATTTGTAT
ATA
>MinSyn_1410|Strength:0.00071462
GCGTGTCGTTTTAGTGAGGGCTTTGTCAAAAGCTAAAAAAGATGATGCTACTTGTGTACAGGGCTCTC
TCTGCCGACAGTGGTCCCAAACACATGTAGGCTATCAGCTTAGCAAGACCTCTATATAAGGTTTTGCT
ATTCATTGAAAGCAGTAGTGACTGATTTGTATATA
>MinSyn_1566|Strength:0.000715217
GCGTGTCGTTTTAGTGAGGGCTTTGTCAAAAGCTAAAAAAGATGATGCGACCTCTAAATTTCGGGAAA
CCTCCTCGGCAAGACCTCCTATATAAGGTTTTGCTATTCATTGAAAGCAGTAGTGACTGATTTGTATA
TA
>MinSyn_1264|Strength:0.000718824
GCGTGTCGTTTTAGTGAGGGCTTTGTCAAAAGCTAAAAAAGATGATGCCTTGTGTACAGGAGCAAGTG
GATTCTGCACCTCACATGTAGGCTTCCATCAACAAATAATCCAAGTAAGCCTATATAAGGTTTTGCTA
TTCATTGAAAGCAGTAGTGACTGATTTGTATATA
>MinSyn_1806|Strength:0.000722811
GCGTGTCGTTTTAGTGAGGTTTTCAACAATTGTCTGCACCTCACATGTAGGCTATAGCAAGTGGATGT
ACTTGTGTATCAGAAGATCAAAGGGCTATGCCTATATAAGGTTTTGCTATTCATTGAAAGCAGTAGTG
ACTGATTTGTATATA
>MinSyn_1934|Strength:0.000738027
GCGTGTCGTTTTAGTGAGGAACCATTATTGCGCTATCGAAAGGACAGTATCGCGGTAGGTCACGACCA

CCAGTGGTCCCTCCACCTGCACCTCACATGTAGGCTATCAGCTTATGAAGCATCTTCCGCGGTAGGTC
ACGACCACTATGCTATATAAGGTTTTGCTATTCATTGAAAGCAGTAGTGACTGATTTGTATATA
>MinSyn_1554|Strength:0.000738571
GCGTGTCGTTTTAGTGAGGTGAAGCATCTTCCCTCACATGTAGGCTATCAATTGCGATAAAGGAAAGG
CTCACTGCTAGGAGGACCGATGCTGATCTTCTATATAAGGTTTTGCTATTCATTGAAAGCAGTAGTGA
CTGATTTGTATATA
>MinSyn_1265|Strength:0.000742574
GCGTGTCGTTTTAGTGAGGAACCACGTCTACAACACCTCACATGTAGGCTATCAGCTTAGCACAGTGG
TCCCTCCACTATCCTTACCGCTATGGGTAAGATTCATGTAGGCTATCAGCTTAGCAAGAGGAAAAAGA
AGAGGTGATGCTGATCTTGCCTGCCTTGATAGCAAGTGGATTCTTGCCTGCCTTGCTATATAAGGTTT
TGCTATTCATTGAAAGCAGTAGTGACTGATTTGTATATA
>MinSyn_1983|Strength:0.000743086
GCGTGTCGTTTTAGTGAGGATCGAAAGGACAGTAACATGTAGGCTATCAGCTTAGCAACAGCCACTTG
TGTTAGAGCAAGTGGATAATTAGGTTGTCTGCACCTCACATGTAGGAATTTCGGGAAACCTCCTCGTA
TGCCCAATTAGCTATATAAGGTTTTGCTATTCATTGAAAGCAGTAGTGACTGATTTGTATATA
>MinSyn_1599|Strength:0.000744035
GCGTGTCGTTTTAGTGAGGTTATGACCCCCGCCGATGACGCGGGATTAGCAAGACCAACCATTATTGC
GACTTGTGTACAGGGCTCACTGCTAGGAGAGGTGGCTCCTACTACTTGTGTACAGGGCTCACTGCTAG
GCTATATAAGGTTTTGCTATTCATTGAAAGCAGTAGTGACTGATTTGTATATA
>MinSyn_1146|Strength:0.000744583
GCGTGTCGTTTTAGTGAGGTTATGACCCCCGCCGATGACGCGGGAGGCTATCAGCTTAGCAAAAAATG
TCAAAGATAGTACTTGTGTGGTGGAGCACGACAACTGCTAGGAGGACCGATGCATCGAAAGGACAGTA
GGTCACGACCGCTTTGTCAAAAGCTAAAAAAGATGATGCCACTGCTAGGAGGCTATATAAGGTTTTGC
TATTCATTGAAAGCAGTAGTGACTGATTTGTATATA
>MinSyn_1014|Strength:0.000744836
GCGTGTCGTTTTAGTGAGGATTGCGATAAAGGAAAGGCACTAAAGATTGATGAAAAGTCAAAAACAAA
AATCAATTATAGACCTCTACAAAACTGGTACTTGTGTACAAATTTCGGGAAACCTCCTCGGTACAGGG
CTCACTGCTAGGGCTTTGTCAAAAGCTAAAAAAGATGATGCACCTCTAGGAAAAAGAAGAGGTTGGTA
CTTGTGTACAGGGCTCACTGCCTATATAAGGTTTTGCTATTCATTGAAAGCAGTAGTGACTGATTTGT
ATATA
>MinSyn_1890|Strength:0.000748249
GCGTGTCGTTTTAGTGAGGATCCTTACCGCTATGGGTAAGATTGGCTATCAGCTTAGCAAGACCTGGA
AAAAGAAGAGGTTGGTACTTGTGTACAGGGCTCACTGCTAGAGCAAGTGGATAGCAAGACCTCTACAA
AACTGGTAGTGGGAGCCACCACTCACATGTAGGGCTTTGTCAAAAGCTAAAAAAGATGATGCCAGCTT
AGGTGGCTCCTACGGACCGATGCTGATCTCCATCAACAAATAATCCAAGTAAGGTCACGACCACTATG
CCCAATTAGCTATATAAGGTTTTGCTATTCATTGAAAGCAGTAGTGACTGATTTGTATATA
>MinSyn_1323|Strength:0.000757558
GCGTGTCGTTTTAGTGAGGTCACTATCAGCCTAAGATTGATGAAAAGTCAAAAACAAAAATCAATTAT
GTAGGCTATCAGCTTAGCAAGACCTGGTGGAGCACGACAGGACCGATGCTGATCTTGCCTGCCTTGAT
GAGCAAGTGGATCCGATGCTGATCTTGCCTGCCTCCATCAACAAATAATCCAAGTAAGCAATTAGGGC
TTTGTCAAAAGCTAAAAAAGATGATGCACTATATAAGGTTTTGCTATTCATTGAAAGCAGTAGTGACT
GATTTGTATATA
>MinSyn_1926|Strength:0.000764072
GCGTGTCGTTTTAGTGAGGGGAAAAAGAAGAGGTGGTGGGAGCCACCAAAAACTGGTACTTGTGTACA
GAACCATTATTGCGACTGCTAGGAGGACCGATGAGGTGGCTCCTACCGATGCTGCTTTGTCAAAAGCT
AAAAAAGATGATGCGCTCACTGCTATCGAAAGGACAGTACCTTGACTATATAAGGTTTTGCTATTCAT
TGAAAGCAGTAGTGACTGATTTGTATATA
>MinSyn_1816|Strength:0.000765068
GCGTGTCGTTTTAGTGAGGTGAAGCATCTTCCCTACAAAACTGGTACTTGTGATTGCGATAAAGGAAA
GGGCTAGGAGGACCGATGCTGATCTTCTATATAAGGTTTTGCTATTCATTGAAAGCAGTAGTGACTGA
TTTGTATATA
>MinSyn_1963|Strength:0.000769167
GCGTGTCGTTTTAGTGAGGAGGTGGCTCCTACAGGGCTCACTGCTAGGAGAAGATTGATGAAAAGTCA
AAAACAAAAATCAATTATCAAAACTGGTACTTGTGTACAGGGCTTGGTGGAGCACGACAAGGGCTATC

CTTACCGCTATGGGTAAGATTTGCCCAATTAGGTTTTCAACAACCTCTACAAAACTGGTACTTGGCTT
TGTCAAAAGCTAAAAAAGATGATGCCCTCACATGTAGCTATATAAGGTTTTGCTATTCATTGAAAGCA
GTAGTGACTGATTTGTATATA
>MinSyn_1086|Strength:0.000778243
GCGTGTCGTTTTAGTGAGGTGAAGCATCTTCCGTCACGACCACTATGCCATCCTTACCGCTATGGGTA
AGATTGGTACTTGTGTACAGGGAAAAATGTCAAAGATATACTTGTGTACAGGGCTTATGACCCCCGCC
GATGACGCGGGACAATTAGGTTGTCTGCACCTATTGCGATAAAGGAAAGGGATGCTGATCGAAAGGAC
AGTAGTAGGCTATCAGCTTAGCAGGAAAAAGAAGAGGTTTGCCTGCCTTGAGCTTTGTCAAAAGCTAA
AAAAGATGATGCGGTACTTGTGTACAGGGCTCACTGCTAGCTATATAAGGTTTTGCTATTCATTGAAA
GCAGTAGTGACTGATTTGTATATA
>MinSyn_1973|Strength:0.000781616
GCGTGTCGTTTTAGTGAGGAAAAATGTCAAAGATACTCTACAAAACTGGTACTTTCAGAAGATCAAAG
GGCTAGGAGGACCGATGCTGATCTTGCCTGCCTTGCTATATAAGGTTTTGCTATTCATTGAAAGCAGT
AGTGACTGATTTGTATATA
>MinSyn_1667|Strength:0.000784739
GCGTGTCGTTTTAGTGAGGGCACACCAGCATGTGTTGATCACCAGCTGCTTAGCAAGGTGGCTCCTAC
GTTGTCTGCACCTCACAAAAATGTCAAAGATAGCACCTCACATGTAGGATTGCGATAAAGGAAAGGTG
CTAGGAGGACCGATGCTGATCTTGTCAGAAGATCAAAGGGCTATAGGTTGTCTGCGCTTTGTCAAAAG
CTAAAAAAGATGATGCCTAGGAGGACCGATGCTATATAAGGTTTTGCTATTCATTGAAAGCAGTAGTG
ACTGATTTGTATATA
>MinSyn_1612|Strength:0.000787791
GCGTGTCGTTTTAGTGAGGAAAAATGTCAAAGATAATGTAGGCTATCAGCTTAGCGTGGGAGCCACCA
ACTATGCCCAATTAGGTTGTCTGCACCTCAAATTTCGGGAAACCTCCTCGCCACTATGCCCAATTAGG
TTGTCTGCACAGCCACTTGTGTTGCTGATCTTGCCTGCAGCAAGTGGATTAGGTTGTCTGCACCTCTT
ATGACCCCCGCCGATGACGCGGGAGCTGATCTTGCCTGCCTTGATGATTCACTATCAGCGCTAGGAGG
ACCGATGCTGATCTTGAACCATTATTGCGAAAACTGGTACTTGTGTACAGGGCTCACTTTTCAACAAG
GCTCACTGCTAGGAGGACTATATAAGGTTTTGCTATTCATTGAAAGCAGTAGTGACTGATTTGTATAT
A
>MinSyn_1321|Strength:0.000789045
GCGTGTCGTTTTAGTGAGGGGAAAAAGAAGAGGTCCAATTAGGTTGTCTGCACCTAAAAATGTCAAAG
ATATCAATCGAAAGGACAGTAAAGACCTCTACAAACAGTGGTCCCTCCACACTGCTAGGAGGACCGAT
GCTGATGCTTTGTCAAAAGCTAAAAAAGATGATGCTCTACAAAACTGGTACTTGTGTGGTGGAGCACG
ACAACCGATGCTGATCCAGCCACTTGTGTCCCAATTAGGTTGTCTGCACCTCACATGTTGAAGCATCT
TCCGACCTCTACAAAACTATATAAGGTTTTGCTATTCATTGAAAGCAGTAGTGACTGATTTGTATATA
>MinSyn_1972|Strength:0.000793954
GCGTGTCGTTTTAGTGAGGGCTTTGTCAAAAGCTAAAAAAGATGATGCGCAAGACCTCAACCACGTCT
ACAACGACCACTATGCCCAATTAGGTTGTCTGCAACCATTATTGCGCGACCACTATGCCCAATTAGGT
TGTCTAGGTGGCTCCTACTCTATATAAGGTTTTGCTATTCATTGAAAGCAGTAGTGACTGATTTGTAT
ATA
>MinSyn_1283|Strength:0.000794459
GCGTGTCGTTTTAGTGAGGAAAAATGTCAAAGATACACTAGCTTTGTCAAAAGCTAAAAAAGATGATG
CTCACATGTAGGCTATCAGCTTAGCAATTATGACCCCCGCCGATGACGCGGGAATTAGGTTGTCTGCT
ATATAAGGTTTTGCTATTCATTGAAAGCAGTAGTGACTGATTTGTATATA
>MinSyn_1941|Strength:0.000794495
GCGTGTCGTTTTAGTGAGGGTGGGAGCCACCAAAACTGGTACTTGTGTACAATTTCGGGAAACCTCCT
CGTGCTAGGAGGACCGATGCTGATCTTGCCTCTATATAAGGTTTTGCTATTCATTGAAAGCAGTAGTG
ACTGATTTGTATATA
>MinSyn_1153|Strength:0.000794944
GCGTGTCGTTTTAGTGAGGTGAAGATAAGATAATAATGTTGAAGATAAGACGGTAGGTCACGACCACT
ATGCCATTGCGATAAAGGAAAGGATGCTGATCTTGCCTGCCTAGCAAGTGGATTGTCTGCACCTCTAT
ATAAGGTTTTGCTATTCATTGAAAGCAGTAGTGACTGATTTGTATATA
>MinSyn_1210|Strength:0.000795732
GCGTGTCGTTTTAGTGAGGATCGAAAGGACAGTACAAAACTGGTACTTGTGTACAGCAGCCACTTGTG
TCGACCACTATGCCTTTTCAACAAGGTACTTGTGTACAGGGCTCACTGCTAGGACTATATAAGGTTTT

GCTATTCATTGAAAGCAGTAGTGACTGATTTGTATATA
>MinSyn_1775|Strength:0.000796736
GCGTGTCGTTTTAGTGAGGTCCATCAACAAATAATCCAAGTAAGACCTCACATGTAGGCTATCAGCTT
AGGCACACCAGCATGTGTTGATCACCAGCTACAGGGCTCACTGCTAGGAGGACAAGATTGATGAAAAG
TCAAAAACAAAAATCAATTATGGCTCACTGCTAGGAGGATGAAGATAAGATAATAATGTTGAAGATAA
GACAGCTTAGCAAGACCTCTACAAAACTGGTGCTTTGTCAAAAGCTAAAAAAGATGATGCTACTTGTG
TACAGGGCTCACTGCTATATAAGGTTTTGCTATTCATTGAAAGCAGTAGTGACTGATTTGTATATA
>MinSyn_1269|Strength:0.000802935
GCGTGTCGTTTTAGTGAGGGCACACCAGCATGTGTTGATCACCAGCTTACAGGGCAACCACGTCTACA
AAGGGCTCACTGCTGGTGGAGCACGACAATGCCCAATTAGCAAGTGGATAGGGCTCACTGCGGAAAAA
GAAGAGGTTACTTGTGTACAGGGCTCACTGCTAGGAGGCTATATAAGGTTTTGCTATTCATTGAAAGC
AGTAGTGACTGATTTGTATATA
>MinSyn_1862|Strength:0.000804795
GCGTGTCGTTTTAGTGAGGGCTTTGTCAAAAGCTAAAAAAGATGATGCGAATCCTTACCGCTATGGGT
AAGATTGGTAGGTCACGACCACTATGCTATATAAGGTTTTGCTATTCATTGAAAGCAGTAGTGACTGA
TTTGTATATA
>MinSyn_1552|Strength:0.000809195
GCGTGTCGTTTTAGTGAGGTGAAGATAAGATAATAATGTTGAAGATAAGAGTACAGGGCTCACTGCTA
GGATGAGCTAAGCACATACGTCAGATCAGCTTAGCAAGACCTCTACAAAACTTCAGAAGATCAAAGGG
CTACATGTAGGCTATCAGCTTAGCAAGACCAGGTGGCTCCTACCTACAAGCACACCAGCATGTGTTGA
TCACCAGCTGGAGGACCGATGCTGCAGCCACTTGTGTTAGGAGGACCGATGCTGATGTGGGAGCCACC
ATATGCCCAATTAGGTTGTCTTGAAGCATCTTCCACTTGTGTACAGGGCTCACTGCTAGGAGAGCAAG
TGGATCTGCACCTCACATCTATATAAGGTTTTGCTATTCATTGAAAGCAGTAGTGACTGATTTGTATA
TA
>MinSyn_1872|Strength:0.000812737
GCGTGTCGTTTTAGTGAGGAAGATTGATGAAAAGTCAAAAACAAAAATCAATTATCAGCTTAGCAAGA
CCTCTACAAAATCACTATCAGCATGCCCAATTAGGTTGTCTGCAGGAAAAAGAAGAGGTGGTAGGTCA
CGACCACTATGCCCAATTAGCTATATAAGGTTTTGCTATTCATTGAAAGCAGTAGTGACTGATTTGTA
TATA
>MinSyn_1721|Strength:0.000822457
GCGTGTCGTTTTAGTGAGGTTATGACCCCCGCCGATGACGCGGGATCGCGGTAGGGCTTTGTCAAAAG
CTAAAAAAGATGATGCGCAAGACCTCTAATTTCGGGAAACCTCCTCGCAAGACCTCCTATATAAGGTT
TTGCTATTCATTGAAAGCAGTAGTGACTGATTTGTATATA
>MinSyn_1809|Strength:0.000823514
GCGTGTCGTTTTAGTGAGGAACCATTATTGCGAAGACCTCTACTCACTATCAGCCCGATGCTGATCTT
GCCTGCCTTGATGAGGTGGCTCCTACACAGGGCTCACTGCTAGGAGTTATGACCCCCGCCGATGACGC
GGGAGCACCTCACGCTTTGTCAAAAGCTAAAAAAGATGATGCACTATATAAGGTTTTGCTATTCATTG
AAAGCAGTAGTGACTGATTTGTATATA
>MinSyn_1722|Strength:0.000823839
GCGTGTCGTTTTAGTGAGGCTGACGTAAGGGATGACGCACATTGTCTGCACCTCACATGTAGGCCAGT
GGTCCCTCCACGCTATCAGCTTAGCAAGACCTCTATCAGAAGATCAAAGGGCTATGCCCAATTAGGTT
GTCTGCACCTCACAGCAAGTGGATCGGTAGGTCACGACCACTATGCCCAATTAGTGGGAGCCACCATA
TGCCCAATTAGGTTGTCTGCACCTCACATCTCTCTGCCGACAGTGGTCCCAAAAACTGGTACTTGTGT
AGGTGGCTCCTACAGGACCGATGCTGATCTTGCCTGCCTTGATAAAAATGTCAAAGATAGCTAGGAGG
ACCGCTATATAAGGTTTTGCTATTCATTGAAAGCAGTAGTGACTGATTTGTATATA
>MinSyn_1319|Strength:0.000823855
GCGTGTCGTTTTAGTGAGGTGAAGCATCTTCCTCGCGGTAGGTCACGACCCAGTGGTCCCTCCACATA
AAAATGTCAAAGATATGATCTGGAAAAAGAAGAGGTTCAAATATTTCTTGTAGCAAGACCTCTACAAA
ACTAGCAAGTGGATCTGCTAGGAGGACCGATGCTGATCTTGCCCTATATAAGGTTTTGCTATTCATTG
AAAGCAGTAGTGACTGATTTGTATATA
>MinSyn_1117|Strength:0.000827223
GCGTGTCGTTTTAGTGAGGCAGCCACTTGTGTGATGCTGATCTTTGAAGCATCTTCCATCTTGCCTGC
CTTTATGACCCCCGCCGATGACGCGGGAATAGGTGGCTCCTACCTCACTGCTAGGAGGACCGATGCTG
AGCTTTGTCAAAAGCTAAAAAAGATGATGCCAGGAACCACGTCTACAATAGGAGGACCGATGCTGATC

ATTGCGATAAAGGAAAGGCGCGGTAGGTCACGACCACTATGCTATATAAGGTTTTGCTATTCATTGAA
AGCAGTAGTGACTGATTTGTATATA
>MinSyn_1787|Strength:0.000828614
GCGTGTCGTTTTAGTGAGGGTGGGAGCCACCACTATCAGCTTAGCGCACACCAGCATGTGTTGATCAC
CAGCTTCAGCCACTTGTGTATGCTGAGCTTTGTCAAAAGCTAAAAAAGATGATGCAAGACCTCTACAT
TGCGATAAAGGAAAGGGGCTCACTGCTAGGAGGACCGATGCCTATATAAGGTTTTGCTATTCATTGAA
AGCAGTAGTGACTGATTTGTATATA
>MinSyn_1558|Strength:0.000831852
GCGTGTCGTTTTAGTGAGGTCTCTCTGCCGACAGTGGTCCCAAAACCTCTACAAAACTGGTACTTGTG
TACAGGGCTTTGTCAAAAGCTAAAAAAGATGATGCACCACTATGCCTGGTGGAGCACGACACCTCTAC
AAAACTGGTACTTGAAGCATCTTCCAAACTGGTACTTGTGTACTCACTATCAGCACTTGCAAATATTT
CTTGTCTTGTGTACAGGGCTCACTGCTAGCTATATAAGGTTTTGCTATTCATTGAAAGCAGTAGTGAC
TGATTTGTATATA
>MinSyn_1170|Strength:0.000833212
GCGTGTCGTTTTAGTGAGGCAAATATTTCTTGTAGACCTCTACAAAACTGGTACTTGTGCAGCCACTT
GTGTAGGACCGATGCTGATCTTGCCTGCCTTTTATGACCCCCGCCGATGACGCGGGAAGCAAGACCTC
TACAAAACTGGTACTTGTGCTATATAAGGTTTTGCTATTCATTGAAAGCAGTAGTGACTGATTTGTAT
ATA
>MinSyn_1002|Strength:0.000835882
GCGTGTCGTTTTAGTGAGGTCACTATCAGCCACATGTAGGCTATCAGCTTAGCATGAAGCATCTTCCA
CCTCTACAAAACTGGTACTTGTGCAAATATTTCTTGTCACGACCACTATGCCCAATTAGCTATATAAG
GTTTTGCTATTCATTGAAAGCAGTAGTGACTGATTTGTATATA
>MinSyn_1105|Strength:0.000836902
GCGTGTCGTTTTAGTGAGGATTGCGATAAAGGAAAGGTGCTAGGAGGACCGTTATGACCCCCGCCGAT
GACGCGGGAGGTCACGACCACTATGCCCAAAGCAAGTGGATGTACTTGTGTACAGGGCTCACCTATAT
AAGGTTTTGCTATTCATTGAAAGCAGTAGTGACTGATTTGTATATA
>MinSyn_1626|Strength:0.000844379
GCGTGTCGTTTTAGTGAGGAACCACGTCTACAATGATCTTGCCTGCCTTGATGATTGGTGGAGCACGA
CAGCAAGACCTCTACAAAACTGGTACTTTGAAGATAAGATAATAATGTTGAAGATAAGAGCGGTAGGT
CAATTGCGATAAAGGAAAGGATGCCCAATTAGGTTGTCTGCACCTCACATTTTTCAACAATATCAGCT
TAGCAAGACCTCTACAAAACCTATATAAGGTTTTGCTATTCATTGAAAGCAGTAGTGACTGATTTGTA
TATA
>MinSyn_1868|Strength:0.000845771
GCGTGTCGTTTTAGTGAGGTCCATCAACAAATAATCCAAGTAAGGACCACTATGTCAGAAGATCAAAG
GGCTACACCTCACATGTTCACTATCAGCCACCTCACATGTAGGCTATCAGCTGAAGATAAGATAATAA
TGTTGAAGATAAGACACCTCACATGTAGGCTATCAGCTTAGCAACAGTGGTCCCTCCACAGACCTCTA
CAAAACTGGATCGAAAGGACAGTACTATGCCCAATTAGGTTGTCTGCACCTCAGGTGGCTCCTACCTT
GTGTACAGGGCTCACTAGCAAGTGGATGACCGACTATATAAGGTTTTGCTATTCATTGAAAGCAGTAG
TGACTGATTTGTATATA
>MinSyn_1423|Strength:0.00084728
GCGTGTCGTTTTAGTGAGGATTGCGATAAAGGAAAGGCCTGCCTTGATGATACAGCCACTTGTGTGTA
GGTCACGACCACTATGAAAAATGTCAAAGATACACTGCTAGGAGGACGCTTTGTCAAAAGCTAAAAAA
GATGATGCAGGAGGACCGATGCTGATCTTGCCTGCAATTTCGGGAAACCTCCTCGCAAGACCTCTACA
AAACTGGTACTGTGGGAGCCACCAAAGACCTCTACAAAACTGGTCCATCAACAAATAATCCAAGTAAG
AGGCTATCAGCTTAGCAAGACTATATAAGGTTTTGCTATTCATTGAAAGCAGTAGTGACTGATTTGTA
TATA
>MinSyn_1537|Strength:0.000848611
GCGTGTCGTTTTAGTGAGGGCTTTGTCAAAAGCTAAAAAAGATGATGCTACAAAACTGGTACTTTCTC
TCTGCCGACAGTGGTCCCAAACTACTATATAAGGTTTTGCTATTCATTGAAAGCAGTAGTGACTGATT
TGTATATA
>MinSyn_1237|Strength:0.000849568
GCGTGTCGTTTTAGTGAGGATTGCGATAAAGGAAAGGTAGGTTGTCTGCAAAAAATGTCAAAGATACT
GAAGCATCTTCCGGCTATCAGCTTAGCAAGACCTCTACAAAACCATTATTGCGTACTTGTGTACAGGG
CTCACCTATATAAGGTTTTGCTATTCATTGAAAGCAGTAGTGACTGATTTGTATATA

>MinSyn_1281|Strength:0.000849732
GCGTGTCGTTTTAGTGAGGGGAAAAAGAAGAGGTTACAGGGCTCACTGCTAGGAGGTCTCTCTGCCGA
CAGTGGTCCCAAAGTACTTGTGTACAGGGCTCACTGCTAGGAATTTCGGGAAACCTCCTCGCAGCTTA
GCAAGACCTCTAGCACACCAGCATGTGTTGATCACCAGCTAACTGGTACTTGTGTACAGGGCTCACTG
AAGCATCTTCCAACTGGTACTTGTGTACAGGGCTCACTGCTAACCATTATTGCGGACCACTATGCCCA
ATTAGGTTGTCTGCCTATATAAGGTTTTGCTATTCATTGAAAGCAGTAGTGACTGATTTGTATATA
>MinSyn_1465|Strength:0.000851947
GCGTGTCGTTTTAGTGAGGTGGTGGAGCACGACATAGCAAGTTTTCAACAATGCCCAATTAGGTTGTC
TGCACCTCACATGTCCATCAACAAATAATCCAAGTAAGTATGCCCAATTAGGTTGTCTGCACCTCAGA
AGATCAAAGGGCTAACAGGGCTCACTGCTAGGAGGACCGATGCTATATAAGGTTTTGCTATTCATTGA
AAGCAGTAGTGACTGATTTGTATATA
>MinSyn_1415|Strength:0.000854452
GCGTGTCGTTTTAGTGAGGAACCACGTCTACAAACGACCACTATGCCCAATTAGGTTGAAGATTGATG
AAAAGTCAAAAACAAAAATCAATTATACTTGTGTACTCACTATCAGCCCGATGCTGATCTTGCCTAGC
AAGTGGATGGTCACGACCACTATATAAGGTTTTGCTATTCATTGAAAGCAGTAGTGACTGATTTGTAT
ATA
>MinSyn_1538|Strength:0.000854746
GCGTGTCGTTTTAGTGAGGAACCACGTCTACAACGCGGTAGGTCACGACTGGTGGAGCACGACAGGCT
TTGTCAAAAGCTAAAAAAGATGATGCGCCCAATTTGAAGCATCTTCCCCTCTACAAAACTGGTACTTG
TGTACAAAAATGTCAAAGATAGATGCTGATCTTGCCTGCCTTGATGATCGAAAGGACAGTAAGGAGGA
CCGATGCCTATATAAGGTTTTGCTATTCATTGAAAGCAGTAGTGACTGATTTGTATATA
>MinSyn_1507|Strength:0.000855512
GCGTGTCGTTTTAGTGAGGAACCATTATTGCGTACAAAACTGGTACTTGTGTACAGGGCTCATCACTA
TCAGCCTGCACCTCACATGTAGGCTATCAGCTTATGGTGGAGCACGACACACTGCTATATAAGGTTTT
GCTATTCATTGAAAGCAGTAGTGACTGATTTGTATATA
>MinSyn_1888|Strength:0.000856484
GCGTGTCGTTTTAGTGAGGCAGTGGTCCCTCCACTCTTGCCTGCGCACACCAGCATGTGTTGATCACC
AGCTTCACATGTCAAATATTTCTTGTCACAAAAATGTCAAAGATAACAAAACTGGGCTTTGTCAAAAG
CTAAAAAAGATGATGCCGACCACTATGCCCAATTACTATATAAGGTTTTGCTATTCATTGAAAGCAGT
AGTGACTGATTTGTATATA
>MinSyn_1884|Strength:0.000859706
GCGTGTCGTTTTAGTGAGGATCCTTACCGCTATGGGTAAGATTAGCTTTGTCAAAAGCTAAAAAAGAT
GATGCGCTCACTGCTCCATCAACAAATAATCCAAGTAAGGCTATCAGCTTAGCAAGACCTCTACAACT
ATATAAGGTTTTGCTATTCATTGAAAGCAGTAGTGACTGATTTGTATATA
>MinSyn_1192|Strength:0.000859789
GCGTGTCGTTTTAGTGAGGCAAATATTTCTTGTCCCAATTAGGTATCCTTACCGCTATGGGTAAGATT
GAGGACCGATGCTGATCTTGCCTAATTTCGGGAAACCTCCTCGGCCCAATTAAGGTGGCTCCTACGTG
TACAGGGCTCACTGCTAGGCAGCCACTTGTGTACATGTAGGCTATCAGCTTAGCAAGACAAAAATGTC
AAAGATATTGTCTGCACCTCACATGTAGGCTATCAGCTATATAAGGTTTTGCTATTCATTGAAAGCAG
TAGTGACTGATTTGTATATA
>MinSyn_1252|Strength:0.000867721
GCGTGTCGTTTTAGTGAGGGCACACCAGCATGTGTTGATCACCAGCTGGACCGATGCTGATCTTGCCT
GCCTTGATAACCATTATTGCGGGCTCATCAGAAGATCAAAGGGCTAGTACTTGTGTACAGGCTTTGTC
AAAAGCTAAAAAAGATGATGCGCTCACAAAAATGTCAAAGATAAGGGCCTATATAAGGTTTTGCTATT
CATTGAAAGCAGTAGTGACTGATTTGTATATA
>MinSyn_1497|Strength:0.000868984
GCGTGTCGTTTTAGTGAGGTTTTCAACAATCGCCAGCCACTTGTGTAAACTGGTACTTGTGTACAGGG
CTCACTTCCATCAACAAATAATCCAAGTAAGTACAGGGCTCACTGCTAGGAGGACCGATGCCTATATA
AGGTTTTGCTATTCATTGAAAGCAGTAGTGACTGATTTGTATATA
>MinSyn_1064|Strength:0.000871774
GCGTGTCGTTTTAGTGAGGTCACTATCAGCGCTCACTGCTAGGAGGACCGATGCTGATTTTTCAACAA
TCTGCTATATAAGGTTTTGCTATTCATTGAAAGCAGTAGTGACTGATTTGTATATA
>MinSyn_1804|Strength:0.000873004
GCGTGTCGTTTTAGTGAGGTTATGACCCCCGCCGATGACGCGGGAGACCTCTACAAAACTGGTACTTG

TGCAGCCACTTGTGTCGCGGTAGGTCACGACCACTATGCCCAATCACTATCAGCCACGACCACTATGC
CCAATTAGGTTGTCTGGCACACCAGCATGTGTTGATCACCAGCTTACAGGGCTCACTGCTAGGAGGAC
CCTATATAAGGTTTTGCTATTCATTGAAAGCAGTAGTGACTGATTTGTATATA
>MinSyn_1820|Strength:0.000877083
GCGTGTCGTTTTAGTGAGGTGAAGATAAGATAATAATGTTGAAGATAAGAGGACCGATGCTGATCTTA
ATTTCGGGAAACCTCCTCGCTCACATGTAGGCTATCAGCTTAGCAGCACACCAGCATGTGTTGATCAC
CAGCTCATGTAGGCTATCAGCTTAGCAAGACCAACCATTATTGCGTGCTGATCTTGCCTGCCTTGATG
AAGCAAGTGGATACAAAACTGGTACTTGTGTACAGGGCTCTGGTGGAGCACGACAACCTCACATGTAG
GCTTTATGACCCCCGCCGATGACGCGGGAATGCTGATCTTGCCTGCCTTGATGACTATATAAGGTTTT
GCTATTCATTGAAAGCAGTAGTGACTGATTTGTATATA
>MinSyn_1710|Strength:0.000882035
GCGTGTCGTTTTAGTGAGGGGAAAAAGAAGAGGTTCTACAAAACTGGTACTTGTGTGGTGGAGCACGA
CATCTGCACCTCACATGTAGGCTATTATGACCCCCGCCGATGACGCGGGATAGAGCAAGTGGATAGGT
TGTCTGCACCTCACATGTAGGCTAGGTGGCTCCTACTGCCTGCCAACCACGTCTACAACTACAAAACT
GGTACTTGTGTACAGGCTATATAAGGTTTTGCTATTCATTGAAAGCAGTAGTGACTGATTTGTATATA
>MinSyn_1212|Strength:0.000887428
GCGTGTCGTTTTAGTGAGGATCCTTACCGCTATGGGTAAGATTGACCTCTACAAAACTGGCTTTGTCA
AAAGCTAAAAAAGATGATGCACATGTAGGAACCATTATTGCGGGCTCACTGCTCAGAAGATCAAAGGG
CTAACCTCACATGTAGGCTATCAGCTTCTATATAAGGTTTTGCTATTCATTGAAAGCAGTAGTGACTG
ATTTGTATATA
>MinSyn_1997|Strength:0.000889804
GCGTGTCGTTTTAGTGAGGTCACTATCAGCGATGCTGATCTTGCCTGCCTTGATGATTGCGATAAAGG
AAAGGTAGGCTATCAGCTTAGCAAGACCTCTACAAAAAATGTCAAAGATACCAATTAGGTTGTCAACC
ATTATTGCGATGCTGATCTTGCCTGCCTTGACTATATAAGGTTTTGCTATTCATTGAAAGCAGTAGTG
ACTGATTTGTATATA
>MinSyn_1083|Strength:0.000890152
GCGTGTCGTTTTAGTGAGGGTGGGAGCCACCATCACATGTAGGCTATCAGCTTAGCAAGATTTTCAAC
AAATGCTGATCTCTATATAAGGTTTTGCTATTCATTGAAAGCAGTAGTGACTGATTTGTATATA
>MinSyn_1920|Strength:0.000890409
GCGTGTCGTTTTAGTGAGGCAAATATTTCTTGTTAGGTCACGACCACTAGGAAAAAGAAGAGGTCGTG
GGAGCCACCAAGGACCGATGCTGATCTTGCCTGCCATTGCGATAAAGGAAAGGTTAGGTTGTCTGCAC
CTCACATGCTATATAAGGTTTTGCTATTCATTGAAAGCAGTAGTGACTGATTTGTATATA
>MinSyn_1378|Strength:0.000891386
GCGTGTCGTTTTAGTGAGGTTTTCAACAATGTACAGGGCTCACTGCTAGGAGGAGGTGGCTCCTACGA
GGACCGATGCTGATCTCACTATCAGCAGCAAGACCTCTACACAGCCACTTGTGTAATTAGGTTGTCCT
ATATAAGGTTTTGCTATTCATTGAAAGCAGTAGTGACTGATTTGTATATA
>MinSyn_1194|Strength:0.000891485
GCGTGTCGTTTTAGTGAGGGGAAAAAGAAGAGGTCGAAACCATTATTGCGGACCGATGCTGATCTTGC
CTGCCTCAGAAGATCAAAGGGCTACCTCTACAAAACTGGTACTTCTATATAAGGTTTTGCTATTCATT
GAAAGCAGTAGTGACTGATTTGTATATA
>MinSyn_1326|Strength:0.000891738
GCGTGTCGTTTTAGTGAGGCAGTGGTCCCTCCACCTATGCCCAATTAGGTTGGCTTTGTCAAAAGCTA
AAAAAGATGATGCTACTTGTGTACAGGGCTCAGCACACCAGCATGTGTTGATCACCAGCTCACCTCAC
ATGTGAAGCATCTTCCGTACAGGGCTCACTGCTAGGAAACCATTATTGCGTGTACAGGGCTCACTGCT
ACTATATAAGGTTTTGCTATTCATTGAAAGCAGTAGTGACTGATTTGTATATA
>MinSyn_1632|Strength:0.000891779
GCGTGTCGTTTTAGTGAGGGCACACCAGCATGTGTTGATCACCAGCTATAAACCACGTCTACAAACTA
TGCCCAATTAGGTTGTCTGCCAAATATTTCTTGTCTCTACAAAACTGGTACTTGTGTCTATATAAGGT
TTTGCTATTCATTGAAAGCAGTAGTGACTGATTTGTATATA
>MinSyn_1701|Strength:0.000891971
GCGTGTCGTTTTAGTGAGGATCCTTACCGCTATGGGTAAGATTATTAGGTTGTCTGCACCTCACATGT
AGCAGTGGTCCCTCCACCGCGGTGCACACCAGCATGTGTTGATCACCAGCTGCGGTAGGTCACGACCA
CTATGCCAAGATTGATGAAAAGTCAAAAACAAAAATCAATTATCTTAGCAAGACCTCTACAAAACTGG
TACTCAGAAGATCAAAGGGCTATTAGGCTTTGTCAAAAGCTAAAAAAGATGATGCATCAGCTTAGCAA

CAGCCACTTGTGTAGGAGGACCGATGCTGATCTTGCCTAACCATTATTGCGCACGACCACTATGCCCA
ATTAGGTTCACTATCAGCGTCTGCACCTCACATGTAGGCTATATAAGGTTTTGCTATTCATTGAAAGC
AGTAGTGACTGATTTGTATATA
>MinSyn_1990|Strength:0.000892088
GCGTGTCGTTTTAGTGAGGCAGCCACTTGTGTGAGGACCGATGCTGATCTTGTCACTATCAGCGCTGA
TCTTGCCTGCCTAGGTGGCTCCTACACAAAACTGGTACTTGTGTTCTCTCTGCCGACAGTGGTCCCAA
ACTGGTACTTGTGTACAGGGCTCACTGCTCTATATAAGGTTTTGCTATTCATTGAAAGCAGTAGTGAC
TGATTTGTATATA
>MinSyn_1026|Strength:0.000892658
GCGTGTCGTTTTAGTGAGGAACCACGTCTACAACTGTGAAGATAAGATAATAATGTTGAAGATAAGAA
CTATGCCCAGGTGGCTCCTACCCAATTAGGTTGTCTGCACCTCACATGTAGAAAAATGTCAAAGATAT
GTACAGGGCTCACTGCTAGGAGGACCGCTATATAAGGTTTTGCTATTCATTGAAAGCAGTAGTGACTG
ATTTGTATATA
>MinSyn_1475|Strength:0.000893662
GCGTGTCGTTTTAGTGAGGATCGAAAGGACAGTACAAGACCTCTACAAAACTGGTTTTCAACAACAGC
TTAGCAAGACCTCTACAAAACTCTATATAAGGTTTTGCTATTCATTGAAAGCAGTAGTGACTGATTTG
TATATA
>MinSyn_1387|Strength:0.000896947
GCGTGTCGTTTTAGTGAGGTTTTCAACAATGCTAGGAGGACCGATGCTGATCTTGCAACCATTATTGC
GCCCAATTAGGCTATATAAGGTTTTGCTATTCATTGAAAGCAGTAGTGACTGATTTGTATATA
>MinSyn_1889|Strength:0.000899401
GCGTGTCGTTTTAGTGAGGCAGTGGTCCCTCCACCTGCTAGGAGGCACACCAGCATGTGTTGATCACC
AGCTCTCACATGTAGGCTATCAGCTTAGTTATGACCCCCGCCGATGACGCGGGATCACTGCTAGAGCA
AGTGGATGCACCTCACATGTAGGCTATCAGCTTACTATATAAGGTTTTGCTATTCATTGAAAGCAGTA
GTGACTGATTTGTATATA
>MinSyn_1887|Strength:0.00090247
GCGTGTCGTTTTAGTGAGGCAGTGGTCCCTCCACCTATCAGCTTATCCATCAACAAATAATCCAAGTA
AGTGTAGGCTATCAGCTTAGCAAGACCTCTACGCTTTGTCAAAAGCTAAAAAAGATGATGCGTACAGG
GCTCACTGCTAGGAGGAAGGTGGCTCCTACGGACCGATGCTGATCTTGCCTGTTATGACCCCCGCCGA
TGACGCGGGATATCCTATATAAGGTTTTGCTATTCATTGAAAGCAGTAGTGACTGATTTGTATATA
>MinSyn_1151|Strength:0.00090269
GCGTGTCGTTTTAGTGAGGTTTTCAACAACTGCACCTCACATTGAAGATAAGATAATAATGTTGAAGA
TAAGAACATGTAGGCTATCAGCTTAGCAAGACCAAATATTTCTTGTCTTGAAGCATCTTCCCTATGCC
CAATTAGGTTGTAGGTGGCTCCTACGCGGTAGGTCACGACCACTATGCCCAATTCTATATAAGGTTTT
GCTATTCATTGAAAGCAGTAGTGACTGATTTGTATATA
>MinSyn_1966|Strength:0.000903778
GCGTGTCGTTTTAGTGAGGCAGTGGTCCCTCCACGCTCACTGCTAAGCAAGTGGATTCACATGTAGGA
ACCACGTCTACAAGGAGGACCCAGCCACTTGTGTCCCAATTAGGTTGTCTGCACCTATATAAGGTTTT
GCTATTCATTGAAAGCAGTAGTGACTGATTTGTATATA
>MinSyn_1456|Strength:0.000904591
GCGTGTCGTTTTAGTGAGGTTATGACCCCCGCCGATGACGCGGGAAGCAAGACCTCTACAAATCCATC
AACAAATAATCCAAGTAAGCTATGCCCAATTAGGTTAACCACGTCTACAATATCAGCTTAGCAAGACC
TCTACAAAACTGCTATATAAGGTTTTGCTATTCATTGAAAGCAGTAGTGACTGATTTGTATATA
>MinSyn_1933|Strength:0.000905563
GCGTGTCGTTTTAGTGAGGATTGCGATAAAGGAAAGGCAGCTTAGCAAGACCTCTCTCTGCCGACAGT
GGTCCCAAAACATGTAGGCTATCAGCTTAGCAAGACCTAGGTGGCTCCTACGTCTGCACCTCACATGT
TTATGACCCCCGCCGATGACGCGGGATGTGTACAGGGCTCACTGCTAGGAGGAGCAAGTGGATGTCTA
TATAAGGTTTTGCTATTCATTGAAAGCAGTAGTGACTGATTTGTATATA
>MinSyn_1930|Strength:0.000908403
GCGTGTCGTTTTAGTGAGGTGAAGCATCTTCCCTTGTGTACAGGAACCATTATTGCGAGGTTGTCTGC
ACCTCACATGTAGGCTTGGTGGAGCACGACACCGATGCATCGAAAGGACAGTAATCAAAAAATGTCAA
AGATATCACATGTAGGCTATCAGCTTAGCAAGAGGTGGCTCCTACCTAGGAGGACCGATGCTGATCTC
TATATAAGGTTTTGCTATTCATTGAAAGCAGTAGTGACTGATTTGTATATA
>MinSyn_1304|Strength:0.000909298

GCGTGTCGTTTTAGTGAGGGCACACCAGCATGTGTTGATCACCAGCTCATGTAGGCTATCAGCTTAGC
AAGACCTGAAGCATCTTCCCACCTCACATGTAGGCTATCAGCTTAGTTTTCAACAAGCCTTGACTATA
TAAGGTTTTGCTATTCATTGAAAGCAGTAGTGACTGATTTGTATATA
>MinSyn_1931|Strength:0.000911489
GCGTGTCGTTTTAGTGAGGTCCATCAACAAATAATCCAAGTAAGACGACCACTATGCCCAATTTCAGA
AGATCAAAGGGCTAGCAAGAATCGAAAGGACAGTAGCTTAGCAAGACCTGAAGCATCTTCCTGCTGAT
CTTGCCAGGTGGCTCCTACTACAGGGCTCACTGCTAGGAGGAGCACACCAGCATGTGTTGATCACCAG
CTCCCAATTAGGTTGTCTGCACCTCACAAGCAAGTGGATAGGAAAAAGAAGAGGTCTATGCCCAATTA
GGTTGTCTGCACCTCACTATATAAGGTTTTGCTATTCATTGAAAGCAGTAGTGACTGATTTGTATATA
>MinSyn_1557|Strength:0.000914765
GCGTGTCGTTTTAGTGAGGAGCAAGTGGATAGGTCACGAGCACACCAGCATGTGTTGATCACCAGCTG
CTTAGCAAGACCTCTACAAAACTGGTACTCTATATAAGGTTTTGCTATTCATTGAAAGCAGTAGTGAC
TGATTTGTATATA
>MinSyn_1532|Strength:0.000919201
GCGTGTCGTTTTAGTGAGGTCCATCAACAAATAATCCAAGTAAGGACCACTATGCCCAATTAGGTTGT
CACTATCAGCTGATCTTGCCTGCCTTGATGATACAGCCACTTGTGTTTGTCTGCACCTCCTATATAAG
GTTTTGCTATTCATTGAAAGCAGTAGTGACTGATTTGTATATA
>MinSyn_1648|Strength:0.000920046
GCGTGTCGTTTTAGTGAGGTCAGAAGATCAAAGGGCTACCGATGCTGATCTTGCCTGCCTTGGGAAAA
AGAAGAGGTTGCTGATCTTGCCTGCCTTGCACACCAGCATGTGTTGATCACCAGCTACCACTATGCCC
AATTAGGTTGGTGGGAGCCACCACTCACTGCTAGGAGGACCGATGCTGATCTTTTTTCAACAAGTAGG
TCACGACCTATATAAGGTTTTGCTATTCATTGAAAGCAGTAGTGACTGATTTGTATATA
>MinSyn_1712|Strength:0.000921395
GCGTGTCGTTTTAGTGAGGGGAAAAAGAAGAGGTCGGTAGGTCACGAAGGTGGCTCCTACTGTCTGCA
CCTCACATGTAGGCTATCAGCACACCAGCATGTGTTGATCACCAGCTACTATGCCCAATTAGGTTGTC
TGCACCTCGCTTTGTCAAAAGCTAAAAAAGATGATGCGAGGACCGATGCTGATCTTGCCTCAGAAGAT
CAAAGGGCTAACCTCACATGTCTCTCTGCCGACAGTGGTCCCAAACTATGCCCAATTAGGTTGGTGGG
AGCCACCACCCAATTAGGTTGTCTGCACTGAAGCATCTTCCGTACAGGGCTCACTGCTCTATATAAGG
TTTTGCTATTCATTGAAAGCAGTAGTGACTGATTTGTATATA
>MinSyn_1734|Strength:0.000923066
GCGTGTCGTTTTAGTGAGGATCGAAAGGACAGTACTTGAAGATAAGATAATAATGTTGAAGATAAGAT
CTACAAAACTGGTACTTGTGTAGCAAGTGGATTGCTGATCAGTGGTCCCTCCACGTACAGGGCTCACT
GGGAAAAAGAAGAGGTAGGGCTCACAAGATTGATGAAAAGTCAAAAACAAAAATCAATTATGCTGATC
TTGCCTGCTTTGTCAAAAGCTAAAAAAGATGATGCGGACCGATGCTGATCTTGTCACTATCAGCTCTA
CAACTATATAAGGTTTTGCTATTCATTGAAAGCAGTAGTGACTGATTTGTATATA
>MinSyn_1921|Strength:0.000924322
GCGTGTCGTTTTAGTGAGGGTGGGAGCCACCAACTATGCCCAATTAGGTTGTCTGCTGGTGGAGCACG
ACAACCTCTACAAAACCAAATATTTCTTGTACCTCTACAAAACTGGTACTTGTGTACTATATAAGGTT
TTGCTATTCATTGAAAGCAGTAGTGACTGATTTGTATATA
>MinSyn_1162|Strength:0.000926081
GCGTGTCGTTTTAGTGAGGTGGTGGAGCACGACAGCTTAGCGTGGGAGCCACCATTAGCAAGACCTCT
ACAAAACTGGTACTTTCACTATCAGCGCCTGCCTTGATCTATATAAGGTTTTGCTATTCATTGAAAGC
AGTAGTGACTGATTTGTATATA
>MinSyn_1219|Strength:0.000926277
GCGTGTCGTTTTAGTGAGGTTTTCAACAATACTTGTGTACCAGTGGTCCCTCCACACAGGGCTCACTG
CTAGGAGGACCGATGCTCTATATAAGGTTTTGCTATTCATTGAAAGCAGTAGTGACTGATTTGTATAT
A
>MinSyn_1405|Strength:0.000934001
GCGTGTCGTTTTAGTGAGGATTGCGATAAAGGAAAGGACAGGGCTCACTGCTAGGAGGACCGATGCTC
AAATATTTCTTGTGTAGGTCACGACCACTATGCCCAATTACAGTGGTCCCTCCACCGCGGTAGCTATA
TAAGGTTTTGCTATTCATTGAAAGCAGTAGTGACTGATTTGTATATA
>MinSyn_1159|Strength:0.000934146
GCGTGTCGTTTTAGTGAGGGGAAAAAGAAGAGGTAGGGCTCACTGCTAGGAGGACCGATGCTTCACTA
TCAGCAGACCTCTAGCTTTGTCAAAAGCTAAAAAAGATGATGCTACAGGGCTCACTGCTAGGAGGAAG

CAAGTGGATCTCACTGCTAGGAGGACCGATGCTAAGATTGATGAAAAGTCAAAAACAAAAATCAATTA
TACGAACCATTATTGCGTGCCCAATTAGGTTCTATATAAGGTTTTGCTATTCATTGAAAGCAGTAGTG
ACTGATTTGTATATA
>MinSyn_1898|Strength:0.00094
GCGTGTCGTTTTAGTGAGGAGGTGGCTCCTACCGACCACTATGCCCAATTAGGTTGTCTGCAAACCAT
TATTGCGTTGTCTGCACCTCCTATATAAGGTTTTGCTATTCATTGAAAGCAGTAGTGACTGATTTGTA
TATA
>MinSyn_1485|Strength:0.00094031
GCGTGTCGTTTTAGTGAGGATCCTTACCGCTATGGGTAAGATTTCACATGTAGGCTATCAGCTTAGCA
AAACCATTATTGCGTGTACATCACTATCAGCGGCTATCAGCTTAGCAAGACCTCCTATATAAGGTTTT
GCTATTCATTGAAAGCAGTAGTGACTGATTTGTATATA
>MinSyn_1771|Strength:0.000942817
GCGTGTCGTTTTAGTGAGGAACCACGTCTACAAACTGCTAGCAGCCACTTGTGTTCAGCTTAGTCAGA
AGATCAAAGGGCTAGCTAGGAGGACCGATGTCACTATCAGCGCTAGGAGGACCGATGCTGATCTTGCA
ATTTCGGGAAACCTCCTCGGCTAGGAGGACCGATGCTGATCTTGCCTCAGTGGTCCCTCCACACCGAT
GCTGATCTTGCCTGCCTTGATGATTGCGATAAAGGAAAGGTTGCCTGCCTTGATTTTCAACAAGTCAC
GACCACTATGCCCTATATAAGGTTTTGCTATTCATTGAAAGCAGTAGTGACTGATTTGTATATA
>MinSyn_1952|Strength:0.000943963
GCGTGTCGTTTTAGTGAGGTGGTGGAGCACGACAGTACTTGTGTAACCATTATTGCGCAATTAGGGGA
AAAAGAAGAGGTCGGTAGGTTCCATCAACAAATAATCCAAGTAAGGCAAGACCTCTACGCTTTGTCAA
AAGCTAAAAAAGATGATGCCTGCACCTCACAATTTCGGGAAACCTCCTCGCAGCTTAGCAAGACCTCT
ACAAAACTGGTAAAAATGTCAAAGATAAAACTGGTACTTGATCCTTACCGCTATGGGTAAGATTGCAA
GACCTCTACAAAACTGGTACTTGTGCTATATAAGGTTTTGCTATTCATTGAAAGCAGTAGTGACTGAT
TTGTATATA
>MinSyn_1725|Strength:0.000944355
GCGTGTCGTTTTAGTGAGGGGAAAAAGAAGAGGTCCTCTAAATTTCGGGAAACCTCCTCGTACAGGGC
TCACTGCAACCACGTCTACAAACATGTAGGCTATCAGCTTAGCATCAGAAGATCAAAGGGCTAAGCAA
GACCTCTACAAAACTCTATATAAGGTTTTGCTATTCATTGAAAGCAGTAGTGACTGATTTGTATATA
>MinSyn_1038|Strength:0.000944508
GCGTGTCGTTTTAGTGAGGGCTTTGTCAAAAGCTAAAAAAGATGATGCCAATTAGGTTGTCTGCACCT
CAGTGGTCCCTCCACCCAATTAGGTTGTGAAGATAAGATAATAATGTTGAAGATAAGATTGTCTGCAC
CTCACATGTAGGCTACTATATAAGGTTTTGCTATTCATTGAAAGCAGTAGTGACTGATTTGTATATA
>MinSyn_1634|Strength:0.000945909
GCGTGTCGTTTTAGTGAGGAACCATTATTGCGTCTGAAGATAAGATAATAATGTTGAAGATAAGACAC
TATGCCCAATTAGGTTGTCTGCTCTCTCTGCCGACAGTGGTCCCAAAAGACCTCTACAAAACTGGTAC
AATTTCGGGAAACCTCCTCGCTATCAGCTTAGCAAGACCTCTACAAGTGGGAGCCACCACCTCACATG
TAGGCTATCAGCTTCTATATAAGGTTTTGCTATTCATTGAAAGCAGTAGTGACTGATTTGTATATA
>MinSyn_1717|Strength:0.000947593
GCGTGTCGTTTTAGTGAGGATTGCGATAAAGGAAAGGTAGGTTGTCTGCACCTCACATGTAGGCTAAC
CACGTCTACAAAGGTCACGAAGATTGATGAAAAGTCAAAAACAAAAATCAATTATGTAGGCTATCAGC
TTAGCAACAGCCACTTGTGTACCACTATGCCCAATTCACTATCAGCATCAGCTTAGCAAGACCTCTAC
AAAACTAAAAATGTCAAAGATATGCCCAATTAGGTTGTCTGCATTTTCAACAAACCACTATGCCCAAT
TAGGTTGCTATATAAGGTTTTGCTATTCATTGAAAGCAGTAGTGACTGATTTGTATATA
>MinSyn_1250|Strength:0.000948398
GCGTGTCGTTTTAGTGAGGTGGTGGAGCACGACACAGGGCTCACTGCTAGGAGGACCGATGGGAAAAA
GAAGAGGTGCCTGCCTTTTTTCAACAATTGTCTGCACCTCTATATAAGGTTTTGCTATTCATTGAAAG
CAGTAGTGACTGATTTGTATATA
>MinSyn_1110|Strength:0.000952099
GCGTGTCGTTTTAGTGAGGGCACACCAGCATGTGTTGATCACCAGCTTGATCAGAAGATCAAAGGGCT
ATCTGCACCTCACAATCGAAAGGACAGTAACAAAACTGGTACTTGTGTACAGGGCTAAGATTGATGAA
AAGTCAAAAACAAAAATCAATTATTTTATGACCCCCGCCGATGACGCGGGAGTACTATCCTTACCGCT
ATGGGTAAGATTAAGACCTCCAGCCACTTGTGTTTAGGTTGTCTGCACCTCACAACCACGTCTACAAT
GTACAGGGCTCACTGCTAGGGCTTTGTCAAAAGCTAAAAAAGATGATGCCGGTAGGTCACGACCACTA
TGCCCAACTATATAAGGTTTTGCTATTCATTGAAAGCAGTAGTGACTGATTTGTATATA

>MinSyn_1054|Strength:0.000954328
GCGTGTCGTTTTAGTGAGGTGAAGCATCTTCCCAAAACTGGTACTTGTGTCAGCCACTTGTGTCCACT
ATGCCCAATTAGGTTGTCTGCACCTTATGACCCCCGCCGATGACGCGGGAAAACTGATCCTTACCGCT
ATGGGTAAGATTTCGCGGTAGGTCACAGCAAGTGGATCTCACATGTAGGCTATCAGCTTATCGAAAGG
ACAGTATGCCTCTATATAAGGTTTTGCTATTCATTGAAAGCAGTAGTGACTGATTTGTATATA
>MinSyn_1382|Strength:0.000954832
GCGTGTCGTTTTAGTGAGGTGGTGGAGCACGACAGTCACGACCACTATGCCCAATTAGGTAATTTCGG
GAAACCTCCTCGCTATAGGTGGCTCCTACGCTAGGAGGACCGATGCTGATCTTGCCTGCAGCCACTTG
TGTAACTGGTACTTGTGTACCTATATAAGGTTTTGCTATTCATTGAAAGCAGTAGTGACTGATTTGTA
TATA
>MinSyn_1462|Strength:0.000955701
GCGTGTCGTTTTAGTGAGGAAAAATGTCAAAGATAGTCACGACCACTATGCCCAATTAGGTCTCTCTG
CCGACAGTGGTCCCAAAGTCACGACCACTATGCCCACTATATAAGGTTTTGCTATTCATTGAAAGCAG
TAGTGACTGATTTGTATATA
>MinSyn_1179|Strength:0.000958548
GCGTGTCGTTTTAGTGAGGTCACTATCAGCACTGCTAGGAGGACCGATGCCAGTGGTCCCTCCACGCT
ATCAGCTTAGCAAGACCTCTACGTGGGAGCCACCACCAATTAGCTATATAAGGTTTTGCTATTCATTG
AAAGCAGTAGTGACTGATTTGTATATA
>MinSyn_1727|Strength:0.000959517
GCGTGTCGTTTTAGTGAGGCAGCCACTTGTGTATGTAGGCTATCAGCTTAGCAAGACCTCGCACACCA
GCATGTGTTGATCACCAGCTTATGCCCAATTAGGTTGTCTGCCTATATAAGGTTTTGCTATTCATTGA
AAGCAGTAGTGACTGATTTGTATATA
>MinSyn_1607|Strength:0.000961321
GCGTGTCGTTTTAGTGAGGAGGTGGCTCCTACAAACTGGTACTTGTGTACAGGGCAAATATTTCTTGT
TTAGGTTCAGAAGATCAAAGGGCTAGCTATCAGCTTAGAATTTCGGGAAACCTCCTCGTGCCTGCCTT
GATGCAGTGGTCCCTCCACGCAAGACCTCTAAGCAAGTGGATGTACTTGTGTACAGGGCTCACTGCTA
GGAGCAGCCACTTGTGTTCTACAAAACTGGTACTTGTGTACAGTCTCTCTGCCGACAGTGGTCCCAAA
TGTACAGGGCTCACTGCTAGGAGGACCGGTGGGAGCCACCAACTATGCCTATATAAGGTTTTGCTATT
CATTGAAAGCAGTAGTGACTGATTTGTATATA
>MinSyn_1165|Strength:0.000961364
GCGTGTCGTTTTAGTGAGGTTTTCAACAAGCAAGACCTCTACGGAAAAAGAAGAGGTCGACCACTATG
CCCAATTAGGTTCTATATAAGGTTTTGCTATTCATTGAAAGCAGTAGTGACTGATTTGTATATA
>MinSyn_1363|Strength:0.00096184
GCGTGTCGTTTTAGTGAGGTCACTATCAGCAGGGCTCATCAGAAGATCAAAGGGCTAGACCGATGCTG
ATCTTGCCTGCCTTGATGTCCATCAACAAATAATCCAAGTAAGTGCCCAATTAGGTTGTCTGCCTATA
TAAGGTTTTGCTATTCATTGAAAGCAGTAGTGACTGATTTGTATATA
>MinSyn_1266|Strength:0.000963272
GCGTGTCGTTTTAGTGAGGTGAAGCATCTTCCTAGGCTATCAGCTTATTGCGATAAAGGAAAGGCGAG
CAAGTGGATGGCTATCAGCTTAGCAAGACCTCTACAAAATCCATCAACAAATAATCCAAGTAAGCTCC
TATATAAGGTTTTGCTATTCATTGAAAGCAGTAGTGACTGATTTGTATATA
>MinSyn_1205|Strength:0.000964954
GCGTGTCGTTTTAGTGAGGGCTTTGTCAAAAGCTAAAAAAGATGATGCTATCAGCTTAGCAAGACCTC
TACAAAACTAGGTGGCTCCTACCACATGTAGTCCATCAACAAATAATCCAAGTAAGTGCCTTGAAACC
ACGTCTACAAACAGGGCTCACTGCTAGGAGGACCAACCATTATTGCGCAAAACTGGTACTTGTCTATA
TAAGGTTTTGCTATTCATTGAAAGCAGTAGTGACTGATTTGTATATA
>MinSyn_1719|Strength:0.000966253
GCGTGTCGTTTTAGTGAGGTGGTGGAGCACGACACCCAATTAGGTTGTCTGGTGGGAGCCACCAGCTA
TCAGCTTAGCAAGACCTCTTCACTATCAGCGGTAGGTCACGACCACTATGAATTTCGGGAAACCTCCT
CGTGTACAGGGCTCACTGCTATATAAGGTTTTGCTATTCATTGAAAGCAGTAGTGACTGATTTGTATA
TA
>MinSyn_1248|Strength:0.000968293
GCGTGTCGTTTTAGTGAGGGCTTTGTCAAAAGCTAAAAAAGATGATGCCTGCACCTCACATGTAGGCT
ATCACAAATATTTCTTGTCTCACATGTAGGCTATCAGCTTAGCAAAACCATTATTGCGTAGGAGGACC
GATCAGTGGTCCCTCCACAATCCTTACCGCTATGGGTAAGATTGTAGGCTATTTTTCAACAATAGGAG

GACCGATGCTGATCTTATTGCGATAAAGGAAAGGAGGTCACGACCACTATGCCCAATTAGCTATATAA
GGTTTTGCTATTCATTGAAAGCAGTAGTGACTGATTTGTATATA
>MinSyn_1035|Strength:0.000973405
GCGTGTCGTTTTAGTGAGGGCTTTGTCAAAAGCTAAAAAAGATGATGCCCTGGCACACCAGCATGTGT
TGATCACCAGCTTGTACAGGGCTCACTTGAAGCATCTTCCTGTCTGCACCTCACATGTAGGCTATCAG
GGAAAAAGAAGAGGTCCTGCCTTGATCTATATAAGGTTTTGCTATTCATTGAAAGCAGTAGTGACTGA
TTTGTATATA
>MinSyn_1703|Strength:0.000974815
GCGTGTCGTTTTAGTGAGGAACCATTATTGCGGTCTGCACCTCACATGTAGGCTAGGTGGCTCCTACG
ATGCTGATCTTGCCTCTATATAAGGTTTTGCTATTCATTGAAAGCAGTAGTGACTGATTTGTATATA
>MinSyn_1149|Strength:0.000976114
GCGTGTCGTTTTAGTGAGGAATTTCGGGAAACCTCCTCGAGCAAGACCTCTACAAAACAAAAATGTCA
AAGATATGCTGATCTTGCCGCACACCAGCATGTGTTGATCACCAGCTGATGCTGAGCAAGTGGATACC
TCTACAAAACTGGTACTCTATATAAGGTTTTGCTATTCATTGAAAGCAGTAGTGACTGATTTGTATAT
A
>MinSyn_1922|Strength:0.000977309
GCGTGTCGTTTTAGTGAGGTCACTATCAGCTGCCTGCATCGAAAGGACAGTACTGGTACTTGTGTACA
GGGCTCACTGCTGTGGGAGCCACCATACAAAACTGGTACTTGTCAAATATTTCTTGTGGCTATCAGCT
TAGCATCCATCAACAAATAATCCAAGTAAGCGATGCTGATCTTGCCTGCCCTATATAAGGTTTTGCTA
TTCATTGAAAGCAGTAGTGACTGATTTGTATATA
>MinSyn_1647|Strength:0.000977852
GCGTGTCGTTTTAGTGAGGATTGCGATAAAGGAAAGGCAGGGCTCACTGCTAGGAGTCACTATCAGCC
TGCTAGGAGGACCGATGCTGATCTTGCCTCTATATAAGGTTTTGCTATTCATTGAAAGCAGTAGTGAC
TGATTTGTATATA
>MinSyn_1706|Strength:0.000980234
GCGTGTCGTTTTAGTGAGGGCACACCAGCATGTGTTGATCACCAGCTTACTTGTGTACAGGATCGAAA
GGACAGTAGACCGATGCAAATATTTCTTGTGGCTATCAGCTTAGCAAGACCTCTACAATCAGAAGATC
AAAGGGCTATCACTGCTAGGAGGACCGCTATATAAGGTTTTGCTATTCATTGAAAGCAGTAGTGACTG
ATTTGTATATA
>MinSyn_1388|Strength:0.00098125
GCGTGTCGTTTTAGTGAGGCAGTGGTCCCTCCACACTGGTACTTGTGTACAGGAACCATTATTGCGCA
AGACCTCTACAAAACTGGTACTTGTTTATGACCCCCGCCGATGACGCGGGAGCTATCAGCTTACTATA
TAAGGTTTTGCTATTCATTGAAAGCAGTAGTGACTGATTTGTATATA
>MinSyn_1540|Strength:0.000983007
GCGTGTCGTTTTAGTGAGGAGCAAGTGGATCACATGTAGGCCAGTGGTCCCTCCACTGATCTTGCCTG
CCTGGTGGAGCACGACACCGATGCTGATCTTGCCTATATAAGGTTTTGCTATTCATTGAAAGCAGTAG
TGACTGATTTGTATATA
>MinSyn_1015|Strength:0.000985196
GCGTGTCGTTTTAGTGAGGTCCATCAACAAATAATCCAAGTAAGAAAAAGATTGATGAAAAGTCAAAA
ACAAAAATCAATTATTACTTGTGTACAGGGCTCACTGGCTTTGTCAAAAGCTAAAAAAGATGATGCTA
GCAAGACCTCTACAAAAATGTCAAAGATATCAGCTTAGCAAGACCTGGTGGAGCACGACATACAAAAC
CTATATAAGGTTTTGCTATTCATTGAAAGCAGTAGTGACTGATTTGTATATA
>MinSyn_1577|Strength:0.000988161
GCGTGTCGTTTTAGTGAGGAGGTGGCTCCTACCTCACTGCTAGGAGGACCGATGCGCTTTGTCAAAAG
CTAAAAAAGATGATGCAAGTCTCTCTGCCGACAGTGGTCCCAAAGTGTACAGTGGTGGAGCACGACAC
CACTATGCCCAATTAGGTTGTCTGCCTATATAAGGTTTTGCTATTCATTGAAAGCAGTAGTGACTGAT
TTGTATATA
>MinSyn_1676|Strength:0.000989401
GCGTGTCGTTTTAGTGAGGGGAAAAAGAAGAGGTATCCATCAACAAATAATCCAAGTAAGTTAGCAAG
TGGATACTGGTGCTTTGTCAAAAGCTAAAAAAGATGATGCACAAAACTGGTACTTGTGTACAGGGCTT
TTCAACAATGCACCTCACATGTAGGCTTCTCTCTGCCGACAGTGGTCCCAAAACTGCTAGATTGCGAT
AAAGGAAAGGAGGTTGTCTGCACCTCACATGTCTATATAAGGTTTTGCTATTCATTGAAAGCAGTAGT
GACTGATTTGTATATA
>MinSyn_1683|Strength:0.000990577

GCGTGTCGTTTTAGTGAGGATGAGCTAAGCACATACGTCAGAGGACCGGAAAAAGAAGAGGTTATGCC
CAATTAGGTTGTCTGCACCTCACCAAATATTTCTTGTAGCAAGACCTCTACAAAACTGGTACTTTCAC
TATCAGCGCAGTGGTCCCTCCACACAGGGCTCACTGCTAGGAGGACCGATTGAAGCATCTTCCCCTCC
ATCAACAAATAATCCAAGTAAGCTAGGAGGACCGATGCTGAATTGCGATAAAGGAAAGGCAATTAGGT
TGTCTGCACCTCTTTTCAACAAACAAAACTGGTACTTGTGTACAGGGCCTATATAAGGTTTTGCTATT
CATTGAAAGCAGTAGTGACTGATTTGTATATA
>MinSyn_1130|Strength:0.000991146
GCGTGTCGTTTTAGTGAGGGCACACCAGCATGTGTTGATCACCAGCTCGATGCTGATCTTGCCTGCCT
TTATGACCCCCGCCGATGACGCGGGAACCTCACATGTAGGCTACAAATATTTCTTGTGTTGTCTGCAC
CTCACATGTAGGTCCATCAACAAATAATCCAAGTAAGAGCTTAGCAAGAGCTTTGTCAAAAGCTAAAA
AAGATGATGCGGTACTTGTGTACAGGGCTCACTGCTAGTCTCTCTGCCGACAGTGGTCCCAAATCACA
TGTAGGCTATCAGCTTAGCTGGTGGAGCACGACAGGAGGACTCAGAAGATCAAAGGGCTATCACATGT
AGGCAACCATTATTGCGTATGCCCAATTAGGTTGTCTGCACCTCTATATAAGGTTTTGCTATTCATTG
AAAGCAGTAGTGACTGATTTGTATATA
>MinSyn_1243|Strength:0.000991743
GCGTGTCGTTTTAGTGAGGTCACTATCAGCCACGACCACTATGCTTTTCAACAAACTCTATATAAGGT
TTTGCTATTCATTGAAAGCAGTAGTGACTGATTTGTATATA
>MinSyn_1977|Strength:0.000992454
GCGTGTCGTTTTAGTGAGGCAAATATTTCTTGTACTGCTAGGAGGACCGATGCATCCTTACCGCTATG
GGTAAGATTCTCTACAAAACTGGTACTTGTGTACAGGGCCAGCCACTTGTGTCCTCTACGGAAAAAGA
AGAGGTGACCGATGCTGATCTTGTGAAGATAAGATAATAATGTTGAAGATAAGACACCTCACATGTAG
GCTATCAGCTTAGCTGAAGCATCTTCCTAGGCTATCAGCTTAGCAAGATGGTGGAGCACGACATGCCC
AATTAGGTTGTCTGCACCTCACTTATGACCCCCGCCGATGACGCGGGACTCACTGCTAGGAGGACCGT
TTTCAACAAGCGGTAGGTCACGACCACTATGCCCAATTCTATATAAGGTTTTGCTATTCATTGAAAGC
AGTAGTGACTGATTTGTATATA
>MinSyn_1815|Strength:0.000993654
GCGTGTCGTTTTAGTGAGGTTATGACCCCCGCCGATGACGCGGGAAGGGCTCACTGCTGGTGGAGCAC
GACATGCACCTCACATGTAGGCATCCTTACCGCTATGGGTAAGATTTGTAGGCTATCAGCTTAGCAAG
ACCTCTACCTATATAAGGTTTTGCTATTCATTGAAAGCAGTAGTGACTGATTTGTATATA
>MinSyn_1373|Strength:0.000994991
GCGTGTCGTTTTAGTGAGGTCTCTCTGCCGACAGTGGTCCCAAAGACCACTATGCCCAATTAGGTCAG
CCACTTGTGTACAAAACTGGTACTTGTGTACAGGGCATCCTTACCGCTATGGGTAAGATTATCTTGCC
TGCCTCTATATAAGGTTTTGCTATTCATTGAAAGCAGTAGTGACTGATTTGTATATA
>MinSyn_1356|Strength:0.000996292
GCGTGTCGTTTTAGTGAGGGCACACCAGCATGTGTTGATCACCAGCTACATGTAGGCTATCAGCTTAG
CAAGACCGCTTTGTCAAAAGCTAAAAAAGATGATGCGCACCTCACATGTAGGCTGAAGCATCTTCCTG
ATCTTGCCTGTCACTATCAGCCTGCACCTCACATGTAGGCTATCAGCTTAAAAAATGTCAAAGATAAC
TATGCCCAATTAGATCCTTACCGCTATGGGTAAGATTCATGTAGGCTATTGCGATAAAGGAAAGGAGG
ATCAGAAGATCAAAGGGCTAGTAGGTCACGACCACTATGCCCAATCAGTGGTCCCTCCACACCTCTAC
AAAACTGGTACTTGTGTACCTATATAAGGTTTTGCTATTCATTGAAAGCAGTAGTGACTGATTTGTAT
ATA
>MinSyn_1106|Strength:0.000996677
GCGTGTCGTTTTAGTGAGGGCACACCAGCATGTGTTGATCACCAGCTACAAATCGAAAGGACAGTAAA
ACTGGTACTTGTTGAAGCATCTTCCACCACTAAAAATGTCAAAGATAAGGTTGTCTGCACCTCACATG
TAGGCTATCAGGTGGCTCCTACTGCTGAAATTTCGGGAAACCTCCTCGCCAATTAGGTTGTCTGGCTT
TGTCAAAAGCTAAAAAAGATGATGCTTAGGTTGTCTGCACCTCACATGAAGATAAGATAATAATGTTG
AAGATAAGACTAGGAGGACTATATAAGGTTTTGCTATTCATTGAAAGCAGTAGTGACTGATTTGTATA
TA
>MinSyn_1473|Strength:0.000998978
GCGTGTCGTTTTAGTGAGGTCCATCAACAAATAATCCAAGTAAGTTAAACCACGTCTACAAACATGTA
GGCTATCAGCTTAGCAAGACCATCCTTACCGCTATGGGTAAGATTGGACCGATGCTGATCTTGCCTGC
CTTGCAGCCACTTGTGTAAACTGGTCAGTGGTCCCTCCACGGACCGATGCTGATCTTGCCTTCACTAT
CAGCTTGTCTGCACCTCACATGCTATATAAGGTTTTGCTATTCATTGAAAGCAGTAGTGACTGATTTG
TATATA

>MinSyn_1476|Strength:0.001002206
GCGTGTCGTTTTAGTGAGGCAAATATTTCTTGTCGCGGTAGGTCACGACCACTATGAGGTGGCTCCTA
CGGTAGGTCACGACCACTATATAAGGTTTTGCTATTCATTGAAAGCAGTAGTGACTGATTTGTATATA
>MinSyn_1845|Strength:0.001004088
GCGTGTCGTTTTAGTGAGGAGGTGGCTCCTACGGTAGGTCACGACCACTATGCCCAATTAGATTGCGA
TAAAGGAAAGGGAGGACCGATGCTGATCTTGCCTGCCTGAAGATAAGATAATAATGTTGAAGATAAGA
CGATGCTGATCTTGCCTGCCTTCACTATCAGCGAGCAAGTGGATTCACGACCACTATGCCCAATTAGG
TTGAAGATTGATGAAAAGTCAAAAACAAAAATCAATTATACCACTATGCCCAATTAGGTTGTCCTATA
TAAGGTTTTGCTATTCATTGAAAGCAGTAGTGACTGATTTGTATATA
>MinSyn_1832|Strength:0.001005226
GCGTGTCGTTTTAGTGAGGATGACGTAAGCCATGACGTCTAACTTGTGTACAGGGCTCACTGCTAGGA
GGAAGCAAGTGGATCCACTATGCCCAATTATTTTCAACAACCTCACATGTAGGCTATCAGCTTAGCAG
GTGGCTCCTACCAATTAGGTTGTCTGCACCTCATCAGAAGATCAAAGGGCTAAGACCTCTACAAAACC
ATTATTGCGCACTGCTAGGAGGCACACCAGCATGTGTTGATCACCAGCTCCTCACATGTAAAAAATGT
CAAAGATATATGCCCAATTAGGTTGTCTGCACTCTCTCTGCCGACAGTGGTCCCAAATGCTAGGAGGA
CCGATGCTGATCTTGCCTCTATATAAGGTTTTGCTATTCATTGAAAGCAGTAGTGACTGATTTGTATA
TA
>MinSyn_1596|Strength:0.001006079
GCGTGTCGTTTTAGTGAGGATCCTTACCGCTATGGGTAAGATTTGTAGGCTATCAGCTTCTCTCTGCC
GACAGTGGTCCCAAAAAAACTGGTACTTGTGTACAGGGCTCACTGTCACTATCAGCGTACAGGGCTCA
CTGCTGCACACCAGCATGTGTTGATCACCAGCTTACAGGGCTCACTGCTAGGAGGACCGACTATATAA
GGTTTTGCTATTCATTGAAAGCAGTAGTGACTGATTTGTATATA
>MinSyn_1985|Strength:0.00100963
GCGTGTCGTTTTAGTGAGGAACCATTATTGCGCACCTCACAAACCACGTCTACAAGTAGGCTATCAGC
TTAGCAAGACCTCTACTATATAAGGTTTTGCTATTCATTGAAAGCAGTAGTGACTGATTTGTATATA
>MinSyn_1455|Strength:0.00101028
GCGTGTCGTTTTAGTGAGGTCACTATCAGCTTTTTCAACAATCTGCACCTCACATCTATATAAGGTTT
TGCTATTCATTGAAAGCAGTAGTGACTGATTTGTATATA
>MinSyn_1007|Strength:0.001011806
GCGTGTCGTTTTAGTGAGGTGGTGGAGCACGACATCAGCTTAGCAAGACCCAAATATTTCTTGTGCTC
ACTGCTAGGAGGACCGATGCTGATCTATATAAGGTTTTGCTATTCATTGAAAGCAGTAGTGACTGATT
TGTATATA
>MinSyn_1249|Strength:0.001012206
GCGTGTCGTTTTAGTGAGGCAGTGGTCCCTCCACCAAGACCTCTACAGGTGGCTCCTACACCGATGCT
GATCTTGCCTGCCGCACACCAGCATGTGTTGATCACCAGCTTATCAGCTTAGCAAGACCTCTATTGCG
ATAAAGGAAAGGCACCTCACATGTAGGCTATCAGCTTAGCAAACCACGTCTACAACAGGGCTCACTGC
TATCAGAAGATCAAAGGGCTAACCTCTACAAAACTGGTACTTGTCTATATAAGGTTTTGCTATTCATT
GAAAGCAGTAGTGACTGATTTGTATATA
>MinSyn_1195|Strength:0.001013584
GCGTGTCGTTTTAGTGAGGATCCTTACCGCTATGGGTAAGATTGTAGGTCACGACCACTATGCCCAAA
CCACGTCTACAAACAAAACTGGTACTTGTGTTGAAGCATCTTCCTGTGTACAGGGCTATATAAGGTTT
TGCTATTCATTGAAAGCAGTAGTGACTGATTTGTATATA
>MinSyn_1137|Strength:0.001013703
GCGTGTCGTTTTAGTGAGGTTATGACCCCCGCCGATGACGCGGGAGAGGACCGATGCTGATCTTGCCT
GCCTTGAGCACACCAGCATGTGTTGATCACCAGCTGTCTCTCTGCCGACAGTGGTCCCAAAGGTAGGT
CACGGCTTTGTCAAAAGCTAAAAAAGATGATGCAGGTCACGACTATATAAGGTTTTGCTATTCATTGA
AAGCAGTAGTGACTGATTTGTATATA
>MinSyn_1591|Strength:0.001014042
GCGTGTCGTTTTAGTGAGGCAGCCACTTGTGTGTGTACAGGGCTCACTGCTTTATGACCCCCGCCGAT
GACGCGGGAGGACCGATGCTGATCTTGCTATATAAGGTTTTGCTATTCATTGAAAGCAGTAGTGACTG
ATTTGTATATA
>MinSyn_1199|Strength:0.00101415
GCGTGTCGTTTTAGTGAGGTGGTGGAGCACGACACATGTAGGCTATCAGCTTAGCAATCCATCAACAA
ATAATCCAAGTAAGTTAGGTTGTCTAGCAAGTGGATAGGGCTCACTGCTAGGAGGATCCTTACCGCTA

TGGGTAAGATTGCCTGCCAGGTGGCTCCTACAATTAGGTTGTCTGCACCTCACATGTCTATATAAGGT
TTTGCTATTCATTGAAAGCAGTAGTGACTGATTTGTATATA
>MinSyn_1879|Strength:0.00101447
GCGTGTCGTTTTAGTGAGGTCACTATCAGCCTACAAAGCACACCAGCATGTGTTGATCACCAGCTATT
AGGTTGTCTAGGTGGCTCCTACTATGCCCAATTAGGTTGTCTGCACCTCACATGAAGCATCTTCCATC
TTGCCTGCCTTGCTATATAAGGTTTTGCTATTCATTGAAAGCAGTAGTGACTGATTTGTATATA
>MinSyn_1400|Strength:0.001014501
GCGTGTCGTTTTAGTGAGGAGCAAGTGGATCTATGCCCAATTAGGTTGTCTGCACCTCACGCTTTGTC
AAAAGCTAAAAAAGATGATGCGTCTGCACCTCACATGTAGGTGGGAGCCACCACATCCATCAACAAAT
AATCCAAGTAAGCTGTGGTGGAGCACGACAGACCGATGCTGATCTTGCCCTATATAAGGTTTTGCTAT
TCATTGAAAGCAGTAGTGACTGATTTGTATATA
>MinSyn_1918|Strength:0.001016175
GCGTGTCGTTTTAGTGAGGGCACACCAGCATGTGTTGATCACCAGCTAGGTTGTCTGCACCTCACATG
TAGAGCAAGTGGATCAGCTTAGCAAGACCTCTACAAAACTGGTAAGATTGATGAAAAGTCAAAAACAA
AAATCAATTATAAAACTGGTACTTGTGTACAGGGCCAAATATTTCTTGTTATCAGCTTAGCATCACTA
TCAGCACCGATGCTGATCTTCCATCAACAAATAATCCAAGTAAGCGATGCTGATCTTGCCTGCCTTGA
TCTATATAAGGTTTTGCTATTCATTGAAAGCAGTAGTGACTGATTTGTATATA
>MinSyn_1560|Strength:0.001018824
GCGTGTCGTTTTAGTGAGGTGGTGGAGCACGACATCTGCACCTCACATGTAGGCTATCAATCGAAAGG
ACAGTAAGACCTCTACAAAACAAATATTTCTTGTCTCACTATCAGCCCAATTAGGTTGTCTGCACCTC
ACTCAGAAGATCAAAGGGCTATCTACAAAACTGGTACTTATGACCCCCGCCGATGACGCGGGAACCTC
TACAAAACTGGTACTTGTGTACAGGCTATATAAGGTTTTGCTATTCATTGAAAGCAGTAGTGACTGAT
TTGTATATA
>MinSyn_1742|Strength:0.001019111
GCGTGTCGTTTTAGTGAGGAGGTGGCTCCTACCGATGCTGATCTTGTTATGACCCCCGCCGATGACGC
GGGAAGGAGGACCGATGTCTCTCTGCCGACAGTGGTCCCAAAACATGTAGGCTATCAGCTTATCACTA
TCAGCGGTACTTGTGTACAGGGCTCACTTTTTCAACAACACTATGCCCACTATATAAGGTTTTGCTAT
TCATTGAAAGCAGTAGTGACTGATTTGTATATA
>MinSyn_1471|Strength:0.001019895
GCGTGTCGTTTTAGTGAGGTCCATCAACAAATAATCCAAGTAAGCTTGCCTGCCTTGATGATATCTCT
CTGCCGACAGTGGTCCCAAATGTGTACAGGGCTCACTGCTAGGAGGACCATTGCGATAAAGGAAAGGC
TACAAACCACGTCTACAAGTCACGACCACTATGAACCATTATTGCGTACTTGTGTACAGGGCTCACTG
CTAGGAGGCTATATAAGGTTTTGCTATTCATTGAAAGCAGTAGTGACTGATTTGTATATA
>MinSyn_1334|Strength:0.001021756
GCGTGTCGTTTTAGTGAGGATGAGCTAAGCACATACGTCAGTGTCTGCACCTCACATGTAGGCTATAA
CCACGTCTACAAACAAAACTGGTCCATCAACAAATAATCCAAGTAAGACTGCTAGGAGGACCGATGCT
GATCTGCTTTGTCAAAAGCTAAAAAAGATGATGCTGCCTGCCTTGATTTTTCAACAATCTGCACCTCA
CATGTAGGCTATCAGCACACCAGCATGTGTTGATCACCAGCTTCTTGCCTGCCTCTCTCTGCCGACAG
TGGTCCCAAACTACAAAACTGGTACTTGTGTACAGGATTGCGATAAAGGAAAGGTACTTGTGTACACT
ATATAAGGTTTTGCTATTCATTGAAAGCAGTAGTGACTGATTTGTATATA
>MinSyn_1092|Strength:0.001023812
GCGTGTCGTTTTAGTGAGGTTTTCAACAACAATTTCGGGAAACCTCCTCGTCACTGCTAGGAGGACCG
ATGCTGGAAAAAGAAGAGGTCGCGGTAGGCAAATATTTCTTGTGGAGGACCGATGCTGATAGGTGGCT
CCTACGGTAGGTCACGACCAAGATTGATGAAAAGTCAAAAACAAAAATCAATTATTCGCGGTAGGTCA
CGACCACTATCAGCCACTTGTGTAGCAAGACCTCTACAAAACTGGTACTTGCTATATAAGGTTTTGCT
ATTCATTGAAAGCAGTAGTGACTGATTTGTATATA
>MinSyn_1470|Strength:0.001026056
GCGTGTCGTTTTAGTGAGGATCGAAAGGACAGTATCTGCACCTCACAAACCACGTCTACAATACAAAA
CTGGTACTTGTGTACAGGGCTCCTATATAAGGTTTTGCTATTCATTGAAAGCAGTAGTGACTGATTTG
TATATA
>MinSyn_1886|Strength:0.001026387
GCGTGTCGTTTTAGTGAGGTTTTCAACAAACATGTAGGCTATCAGCTTAGCAAGACCTAACCATTATT
GCGCTCTACAAAAAAATGTCAAAGATACGATGCTGATCTATATAAGGTTTTGCTATTCATTGAAAGCAG
TAGTGACTGATTTGTATATA

>MinSyn_1066|Strength:0.001027709
GCGTGTCGTTTTAGTGAGGATCGAAAGGACAGTATCACTGCTAGGAGGACCGATGCTGATCAAAAATG
TCAAAGATATAGGAGGACCGATGCAAGATTGATGAAAAGTCAAAAACAAAAATCAATTATACCTCACA
TGTAGGCTATCAGCTTAGAATTTCGGGAAACCTCCTCGCCAATTAGGTTGTCTGCACCTCACTATATA
AGGTTTTGCTATTCATTGAAAGCAGTAGTGACTGATTTGTATATA
>MinSyn_1245|Strength:0.001030027
GCGTGTCGTTTTAGTGAGGAACCATTATTGCGTCAGCTTAGCTTTTCAACAACGATGCTGATCTAATT
TCGGGAAACCTCCTCGATGTAGGCTATCAGCTTTTATGACCCCCGCCGATGACGCGGGAACAGGGCTC
ACTGCTAGGAGGACCGATATTGCGATAAAGGAAAGGATCAGCTTAGCAAGACCTCTACAAAAGCACAC
CAGCATGTGTTGATCACCAGCTGGTTGTCTGCACCTCACATGTAGGCTCTATATAAGGTTTTGCTATT
CATTGAAAGCAGTAGTGACTGATTTGTATATA
>MinSyn_1024|Strength:0.001030622
GCGTGTCGTTTTAGTGAGGATCCTTACCGCTATGGGTAAGATTGGACCGATGCTGATCTTGCCAGCAA
GTGGATTTGTGTACAGGGCTCACTGCTATCACTATCAGCTGATCTTGCCTGCCTTGATGAAGCATCTT
CCGACCTCTACAAAACTGGTACTTGGAAAAAGAAGAGGTGAGGACCGATGCTGATCTTGCCTGCAAGA
TTGATGAAAAGTCAAAAACAAAAATCAATTATGCTTAGCAAGACCTCTAAAAATGTCAAAGATAGAGG
ACCGATGCTGAAGGTGGCTCCTACACGACCACTATGCCCAATTAGCTATATAAGGTTTTGCTATTCAT
TGAAAGCAGTAGTGACTGATTTGTATATA
>MinSyn_1296|Strength:0.00103128
GCGTGTCGTTTTAGTGAGGCAAATATTTCTTGTCATCGAAAGGACAGTAGCAGCCACTTGTGTGTACT
TGTGTACAGGGCTCACTGCTAGGATTTTCAACAAAGGGCTCACTGCTAGGAGGACCGATGCTATTGCG
ATAAAGGAAAGGGGACCGATGCTGATCTTGCCTGCCTATCCTTACCGCTATGGGTAAGATTATTAGGT
TGTCTGCACCTCAGGTGGCTCCTACGCTCACTGCTAGCTATATAAGGTTTTGCTATTCATTGAAAGCA
GTAGTGACTGATTTGTATATA
>MinSyn_1328|Strength:0.001031308
GCGTGTCGTTTTAGTGAGGAGGTGGCTCCTACGCCGTGGGAGCCACCAGTAGGTCACGACCACTATGC
CCAATTGCACACCAGCATGTGTTGATCACCAGCTCTGGTACTTGTGTACAGGGCCTATATAAGGTTTT
GCTATTCATTGAAAGCAGTAGTGACTGATTTGTATATA
>MinSyn_1379|Strength:0.001032382
GCGTGTCGTTTTAGTGAGGAGCAAGTGGATGGTCACGACCACTAATTGCGATAAAGGAAAGGGACCTC
TACAAAACTGGTACTTGTGTACATTATGACCCCCGCCGATGACGCGGGATGTCTGCACCTCACATGTT
CAGAAGATCAAAGGGCTACCTCTACAAAACTGGTTCCATCAACAAATAATCCAAGTAAGCAATTCAGC
CACTTGTGTATGCTGATCTTGCCTGCCTTGATGCAGTGGTCCCTCCACCACCTCACATGTAGGCTATC
AGCTCTATATAAGGTTTTGCTATTCATTGAAAGCAGTAGTGACTGATTTGTATATA
>MinSyn_1258|Strength:0.001033333
GCGTGTCGTTTTAGTGAGGATCGAAAGGACAGTAGTGTACAGGGCTCACTGCTAGGAGGACCAACCAC
GTCTACAATCAGCTTAGCAAGCTATATAAGGTTTTGCTATTCATTGAAAGCAGTAGTGACTGATTTGT
ATATA
>MinSyn_1329|Strength:0.001037241
GCGTGTCGTTTTAGTGAGGTGGTGGAGCACGACAATCTTGCCTGCCTTGATGATGGAAAAAGAAGAGG
TCTAGGAGGACCGATGCTGATCTTGCTATATAAGGTTTTGCTATTCATTGAAAGCAGTAGTGACTGAT
TTGTATATA
>MinSyn_1298|Strength:0.001038432
GCGTGTCGTTTTAGTGAGGTGAAGATAAGATAATAATGTTGAAGATAAGATGTGTACAGGGCTCACTG
CTAGGAGGACCAACCATTATTGCGTACTTGTGTACAGTTTTCAACAACACTATCTATATAAGGTTTTG
CTATTCATTGAAAGCAGTAGTGACTGATTTGTATATA
>MinSyn_1960|Strength:0.001038463
GCGTGTCGTTTTAGTGAGGCAGCCACTTGTGTCACTAACCACGTCTACAAGAATTTCGGGAAACCTCC
TCGGGACCGATGCTGATCTTGCCTGCCGTGGGAGCCACCACACTGCTAGGAGGACCGATGCTGATCTT
GCGGAAAAAGAAGAGGTACTTTGAAGCATCTTCCAGACCTCTACAAAACTCTATATAAGGTTTTGCTA
TTCATTGAAAGCAGTAGTGACTGATTTGTATATA
>MinSyn_1466|Strength:0.001038565
GCGTGTCGTTTTAGTGAGGTGAAGATAAGATAATAATGTTGAAGATAAGAGACCGATCTCTCTGCCGA
CAGTGGTCCCAAAACTTGTAGGTGGCTCCTACCTGATGTGGGAGCCACCAACAGGGCTCACTGCGGAA

AAAGAAGAGGTAGCTTAGCAAGACCTCTACAAAACTGTTTTCAACAAGCTAGGAGGACCGATGCTGAT
CTTGATCCTTACCGCTATGGGTAAGATTTATCAGCTTAGCAAGACCTCTACAACTATATAAGGTTTTG
CTATTCATTGAAAGCAGTAGTGACTGATTTGTATATA
>MinSyn_1627|Strength:0.001040714
GCGTGTCGTTTTAGTGAGGCAGCCACTTGTGTATCAGCTTAGCAAGACCTCTACAAAAAAAAATGTCA
AAGATAATGCTGATCTTGCCCTATATAAGGTTTTGCTATTCATTGAAAGCAGTAGTGACTGATTTGTA
TATA
>MinSyn_1175|Strength:0.001041216
GCGTGTCGTTTTAGTGAGGATCGAAAGGACAGTACAATGGTGGAGCACGACAACAAAACTGGTACTTG
TGTACAGGGCTTCTCTCTGCCGACAGTGGTCCCAAAGACCTCTACAAAACCTATATAAGGTTTTGCTA
TTCATTGAAAGCAGTAGTGACTGATTTGTATATA
>MinSyn_1273|Strength:0.001042256
GCGTGTCGTTTTAGTGAGGATCCTTACCGCTATGGGTAAGATTCCCAATTAGGTTGTCTGCACCTCAC
ATGATCGAAAGGACAGTATACAGGGCTCACTGCTAGGAGGATGAAGCATCTTCCATGCTCACTATCAG
CCTCACTGCTAGGAGGACCGATGCTGATCTAAGATTGATGAAAAGTCAAAAACAAAAATCAATTATCA
CTGCTAGGAGGACCGATGCTGATCAATTTCGGGAAACCTCCTCGGCTAGGAGGACCGATGCTGATCTT
GCAACCACGTCTACAACGGTAGGTCACGACCACTCTATATAAGGTTTTGCTATTCATTGAAAGCAGTA
GTGACTGATTTGTATATA
>MinSyn_1366|Strength:0.001042843
GCGTGTCGTTTTAGTGAGGGTGGGAGCCACCACAGGGCTCACTGCTAGGCTTTGTCAAAAGCTAAAAA
AGATGATGCACTGCTAGGAGCAAGTGGATACATGTAACCATTATTGCGGAGGACCGATGCTCACTATC
AGCATCAGCTTAGGAAAAAGAAGAGGTACATGTAGGCTATCAGCTTAGCAATGAAGATAAGATAATAA
TGTTGAAGATAAGATATCAGCTTAGCAAGACCTCTACAAAACTTTATGACCCCCGCCGATGACGCGGG
AACAGGGCTCACTGCTAGGAGGACCGATGCCAGTGGTCCCTCCACGTAGGTCACCTATATAAGGTTTT
GCTATTCATTGAAAGCAGTAGTGACTGATTTGTATATA
>MinSyn_1984|Strength:0.001043173
GCGTGTCGTTTTAGTGAGGTCCATCAACAAATAATCCAAGTAAGTGTCTGCACCAACCACGTCTACAA
ACAGGGCTCACTGCAATTTCGGGAAACCTCCTCGGCACCTCACATGTAGGCTAGCTTTGTCAAAAGCT
AAAAAAGATGATGCCTACAAAACTGAACCATTATTGCGGTGTACAGGGCTCACTGCTAGGAGGAAAAA
GAAGAGGTGTGTACAGGGTTATGACCCCCGCCGATGACGCGGGAAGGCTATCAGCTTAGCAAGACCTC
TTCAGAAGATCAAAGGGCTAATGTAGGCTATCAGCTCTATATAAGGTTTTGCTATTCATTGAAAGCAG
TAGTGACTGATTTGTATATA
>MinSyn_1371|Strength:0.001043408
GCGTGTCGTTTTAGTGAGGAACCACGTCTACAAGTAGGTCACGACCACTATGCCCATCCTTACCGCTA
TGGGTAAGATTTGCTAGGAGGACCGATGCTGATCTTGCCTTGAAGATAAGATAATAATGTTGAAGATA
AGAATGCCCAATTTATGACCCCCGCCGATGACGCGGGAGCTATCAGCTTAGCAAGACCTCATCGAAAG
GACAGTAAATTAGGTTGTCTGCACCTCACATGTAGCTATATAAGGTTTTGCTATTCATTGAAAGCAGT
AGTGACTGATTTGTATATA
>MinSyn_1825|Strength:0.001043524
GCGTGTCGTTTTAGTGAGGGCTTTGTCAAAAGCTAAAAAAGATGATGCGATGCTGATCTTGCCTGCCT
TGATTCAGAAGATCAAAGGGCTAGTTGTCTGCACCTCTCTCTCTGCCGACAGTGGTCCCAAACAGCTT
AGCAAGACCTCTACAGCCACTTGTGTCAGCTTAGCAAGACTATATAAGGTTTTGCTATTCATTGAAAG
CAGTAGTGACTGATTTGTATATA
>MinSyn_1770|Strength:0.001044078
GCGTGTCGTTTTAGTGAGGAATTTCGGGAAACCTCCTCGGTTGTCTGTCACTATCAGCCCTCTAGGAA
AAAGAAGAGGTCAATTAGGTTGTCTGCACTCTCTCTGCCGACAGTGGTCCCAAAGATGCTGATCTTGC
CTGCCTTGATGATCTATATAAGGTTTTGCTATTCATTGAAAGCAGTAGTGACTGATTTGTATATA
>MinSyn_1976|Strength:0.001044985
GCGTGTCGTTTTAGTGAGGAACCACGTCTACAAACAGGGCTCACTGCTAGGAGTCCATCAACAAATAA
TCCAAGTAAGCAAGACCTCTACAAAACTGGTACTTGTGTTGAAGCATCTTCCCTGCAGCCACTTGTGT
CATCCTTACCGCTATGGGTAAGATTCGACCACTATGCCCAATTAGGTTGTCTTTTTCAACAATCGCGG
TAGGTCACGACCACTATGCCCAATTCTCTCTGCCGACAGTGGTCCCAAACTATCAGCTTAGCAAGACT
ATATAAGGTTTTGCTATTCATTGAAAGCAGTAGTGACTGATTTGTATATA
>MinSyn_1262|Strength:0.001045603

GCGTGTCGTTTTAGTGAGGCAGCCACTTGTGTAGGGCTCACTGCTAGGAGGAAAAAATGTCAAAGATA
GGCTATCAGCTTAGCAAGCAGTGGTCCCTCCACGGTCACGACCACTCTATATAAGGTTTTGCTATTCA
TTGAAAGCAGTAGTGACTGATTTGTATATA
>MinSyn_1189|Strength:0.001045763
GCGTGTCGTTTTAGTGAGGTTATGACCCCCGCCGATGACGCGGGAAGGTCACGACCACTATGCCCAAT
GAAGCATCTTCCAGCATTTTCAACAATGTCTGCACCTCACATCTATATAAGGTTTTGCTATTCATTGA
AAGCAGTAGTGACTGATTTGTATATA
>MinSyn_1781|Strength:0.001047461
GCGTGTCGTTTTAGTGAGGTCCATCAACAAATAATCCAAGTAAGTTCTCTCTGCCGACAGTGGTCCCA
AAACCACTATGCCCAATTAGGTTGTCTGCAAGGTGGCTCCTACTAGGTCACGACCACAAAAATGTCAA
AGATAGGCTCACTGCTAGGAGGACCGATGCTGATCAACCACGTCTACAACGGTATCGAAAGGACAGTA
GACCGATGCTGATCTTGCCTGCTATATAAGGTTTTGCTATTCATTGAAAGCAGTAGTGACTGATTTGT
ATATA
>MinSyn_1659|Strength:0.001049596
GCGTGTCGTTTTAGTGAGGAGCAAGTGGATAGGTCACGAGGTGGCTCCTACCTCACTGCTAGGAGGAC
CGATGTGAAGCATCTTCCGATCAGAAGATCAAAGGGCTAACGACCACTATGTTTTCAACAACTTAGCA
AGACCTCTACAAAACTGGGCTTTGTCAAAAGCTAAAAAAGATGATGCCAGGGCTCACTGCTAGGAGTG
AAGATAAGATAATAATGTTGAAGATAAGACAGGGCAGTGGTCCCTCCACGTCTGCTATATAAGGTTTT
GCTATTCATTGAAAGCAGTAGTGACTGATTTGTATATA
>MinSyn_1439|Strength:0.001051705
GCGTGTCGTTTTAGTGAGGTCCATCAACAAATAATCCAAGTAAGGTGTACATCAGAAGATCAAAGGGC
TATCTGCACCTCACATCACTATCAGCCTCACATGTAGGCTATCATTTTCAACAATTGTGTACAGGGCC
TATATAAGGTTTTGCTATTCATTGAAAGCAGTAGTGACTGATTTGTATATA
>MinSyn_1167|Strength:0.001052239
GCGTGTCGTTTTAGTGAGGGGAAAAAGAAGAGGTCATGTAGGCTATCAGCTTAACCATTATTGCGGAT
GCTGATCTTGCCTGCTATATAAGGTTTTGCTATTCATTGAAAGCAGTAGTGACTGATTTGTATATA
>MinSyn_1529|Strength:0.001054817
GCGTGTCGTTTTAGTGAGGTGAAGATAAGATAATAATGTTGAAGATAAGAATCAGCTGGAAAAAGAAG
AGGTTTAGGTTGTCTGCACCTCACATGTAGGCTAGCACACCAGCATGTGTTGATCACCAGCTCTATCA
GCTTAGCAAGAAGATTGATGAAAAGTCAAAAACAAAAATCAATTATCTTGCCTGCCTTGGCTTTGTCA
AAAGCTAAAAAAGATGATGCTACTTGTGTACACTATATAAGGTTTTGCTATTCATTGAAAGCAGTAGT
GACTGATTTGTATATA
>MinSyn_1354|Strength:0.001055236
GCGTGTCGTTTTAGTGAGGTCTCTCTGCCGACAGTGGTCCCAAACAATTAGGTTGTCTGCACCTTCCA
TCAACAAATAATCCAAGTAAGGCTATCAGCTTAGCAAGACCTTATGACCCCCGCCGATGACGCGGGAG
TGTACAGGGCTCACTGCTAGGAGGACCATCCTTACCGCTATGGGTAAGATTTCACTGCTAGGAGGACC
GATGCTCTATATAAGGTTTTGCTATTCATTGAAAGCAGTAGTGACTGATTTGTATATA
>MinSyn_1545|Strength:0.001055513
GCGTGTCGTTTTAGTGAGGATCCTTACCGCTATGGGTAAGATTGTTGTCTGCACCTCACATGTAGGCG
GAAAAAGAAGAGGTCTTGTCTCTCTGCCGACAGTGGTCCCAAAATGCTGATCTTGCCTGCCTTCAGCC
ACTTGTGTGGGCTCACTGCTAGGAGGACCGTTATGACCCCCGCCGATGACGCGGGACCAATTAGGTTG
TCTGCACCTCACATGTGTGGGAGCCACCACACGTTTTCAACAAGCCCAATTAGGTTGTCTGCACCTCA
CTGGTGGAGCACGACAACCGATGCTGATCCTATATAAGGTTTTGCTATTCATTGAAAGCAGTAGTGAC
TGATTTGTATATA
>MinSyn_1842|Strength:0.001056306
GCGTGTCGTTTTAGTGAGGCAGTGGTCCCTCCACTAGGAGGACCGATGCTGATCTGCACACCAGCATG
TGTTGATCACCAGCTTACTTGTGTACAGGGCTCCTATATAAGGTTTTGCTATTCATTGAAAGCAGTAG
TGACTGATTTGTATATA
>MinSyn_1679|Strength:0.001056359
GCGTGTCGTTTTAGTGAGGAAGATTGATGAAAAGTCAAAAACAAAAATCAATTATATCAGCTTAGCAA
GACCTCTAGCAAGTGGATTATCAGCTTAGCAAGACTTATGACCCCCGCCGATGACGCGGGATGTACAG
GGCTCACAACCATTATTGCGCTCACTGCTAGGAGGACCGATGTCACTATCAGCGGAATTGCGATAAAG
GAAAGGCTATGCCCAATTAGGTTGTCTGCCTATATAAGGTTTTGCTATTCATTGAAAGCAGTAGTGAC
TGATTTGTATATA

>MinSyn_1902|Strength:0.001056813
GCGTGTCGTTTTAGTGAGGAGGTGGCTCCTACGACCACTATGCCCAATTAGGATCCTTACCGCTATGG
GTAAGATTGCTGATCTTGCCTGCCAGCCACTTGTGTGACCTCTACAAAACTGGTACTTGTGGGAGCCA
CCATTGATGATTGGTGGAGCACGACAAGCAAGACCTCTACACTATATAAGGTTTTGCTATTCATTGAA
AGCAGTAGTGACTGATTTGTATATA
>MinSyn_1568|Strength:0.001058797
GCGTGTCGTTTTAGTGAGGTGAAGATAAGATAATAATGTTGAAGATAAGATTGTCTGCACCAAATATT
TCTTGTCTACAAAACTGGTACTTGTGTACAAAAAATGTCAAAGATACTCACATGTAGGCTATCAGCTT
AGCAAAAGATTGATGAAAAGTCAAAAACAAAAATCAATTATGTCTGCACCTCACATGTAGGCTATCAG
CTTATCGAAAGGACAGTATGCCCAATTAGGTTGTCTGCACCTCACATCACTATCAGCGCTATATAAGG
TTTTGCTATTCATTGAAAGCAGTAGTGACTGATTTGTATATA
>MinSyn_1315|Strength:0.001061578
GCGTGTCGTTTTAGTGAGGTCAGAAGATCAAAGGGCTAACATGTAGGCTATCAGCTTAGCAAGATGAA
GCATCTTCCGGCTCACTGCTAGGAGGACCGATAGCAAGTGGATTACTTGTGTACAGGGCTCACTGCTA
AACCACGTCTACAATGATCTTGCCTGCCTTGATAACCATTATTGCGATGATTATGACCCCCGCCGATG
ACGCGGGAACAACAAATATTTCTTGTGGTCACGACCACTATGCCTCTCTCTGCCGACAGTGGTCCCAA
AGTGTACAGGGCTCACTGCTAGGAGGCTATATAAGGTTTTGCTATTCATTGAAAGCAGTAGTGACTGA
TTTGTATATA
>MinSyn_1198|Strength:0.001062643
GCGTGTCGTTTTAGTGAGGTTTTCAACAAGATAGGTGGCTCCTACGTAGGCTATCAGCTTAGCAAGAC
CTCTCAGTGGTCCCTCCACCTATCAGCTTCACTATCAGCATTCTATATAAGGTTTTGCTATTCATTGA
AAGCAGTAGTGACTGATTTGTATATA
>MinSyn_1218|Strength:0.001063852
GCGTGTCGTTTTAGTGAGGTTATGACCCCCGCCGATGACGCGGGAGCTTAGCAAGACCTCTACAAAAC
AGCAAGTGGATGACCACTCACTATCAGCGTGTTTTTCAACAACTAGTCTCTCTGCCGACAGTGGTCCC
AAATGTCTGCACCTCACATGTAGGCTATCAGATCGAAAGGACAGTAACATGTAGGCTATAACCATTAT
TGCGACCTCTACAAAACTGCTATATAAGGTTTTGCTATTCATTGAAAGCAGTAGTGACTGATTTGTAT
ATA
>MinSyn_1052|Strength:0.001064499
GCGTGTCGTTTTAGTGAGGTTATGACCCCCGCCGATGACGCGGGAGCTATCAGCTTAGCAAGAACCAT
TATTGCGATCTTGCCTGAGCAAGTGGATCTAGGAGGACCGATGCTGATCTTCTCTCTGCCGACAGTGG
TCCCAAATACAGGGCTCACTGCTAGGAGGTGAAGCATCTTCCCTTGCCTGCCTTATTGCGATAAAGGA
AAGGCACCTCACCTATATAAGGTTTTGCTATTCATTGAAAGCAGTAGTGACTGATTTGTATATA
>MinSyn_1257|Strength:0.001064875
GCGTGTCGTTTTAGTGAGGATGAGCTAAGCACATACGTCAGACTGGTACTTGTGTACAGGCTTTGTCA
AAAGCTAAAAAAGATGATGCAAACTGGTACAAAAATGTCAAAGATACTTGTGTACAGTCCATCAACAA
ATAATCCAAGTAAGTACAAAACTGGTACTTGTGTACAGGGCTCAGCAAGTGGATTCAGCTTAGCAATC
CTTACCGCTATGGGTAAGATTCCAATAACCACGTCTACAATGGTACTTGTGTACTCAGAAGATCAAAG
GGCTAGGGCTCACTGCAACCATTATTGCGAGGAGGACCGACTATATAAGGTTTTGCTATTCATTGAAA
GCAGTAGTGACTGATTTGTATATA
>MinSyn_1594|Strength:0.001064948
GCGTGTCGTTTTAGTGAGGTGGTGGAGCACGACACTGCTAGGAGGACCGATGCTGATCTTGCCTAATT
TCGGGAAACCTCCTCGGTAGGTCACGACCACTATGCCCAATTAATCCTTACCGCTATGGGTAAGATTT
TAGGTTGTCTGCACCTCACATGTATCGAAAGGACAGTAGATCTTGCAGGTGGCTCCTACTTAGGTTGT
AACCACGTCTACAAGGAGGACCGATGCTGATCTATATAAGGTTTTGCTATTCATTGAAAGCAGTAGTG
ACTGATTTGTATATA
>MinSyn_1116|Strength:0.001065382
GCGTGTCGTTTTAGTGAGGAAAAATGTCAAAGATACACCTCACATGTAGGCTGTGGGAGCCACCAGTT
GTCTGCACCTCACATGTAAGGTGGCTCCTACCTGGTACTTGTGTACAGGGCTCACTGCTAGGAAAAAG
AAGAGGTATGCTGATCTTGCCAACCATTATTGCGCTATCACTATCAGCTGCTAGGAGGACCGATGCTG
ATCTTGCCTCAGCCACTTGTGTGCAAATTTCGGGAAACCTCCTCGAGCAAGACCTCTACAACTATATA
AGGTTTTGCTATTCATTGAAAGCAGTAGTGACTGATTTGTATATA
>MinSyn_1446|Strength:0.0010654
GCGTGTCGTTTTAGTGAGGTTATGACCCCCGCCGATGACGCGGGACCAATTAGGTTGTCTGATCCTTA

CCGCTATGGGTAAGATTTATGCCTCACTATCAGCTCACATGTAGGCTATCAGGTGGCTCCTACGTAGC
AAGTGGATTTTTTCAACAAGGTAGGTCACGACCACTATCTATATAAGGTTTTGCTATTCATTGAAAGC
AGTAGTGACTGATTTGTATATA
>MinSyn_1702|Strength:0.001068182
GCGTGTCGTTTTAGTGAGGCAGTGGTCCCTCCACGGGCTCACTGCTAGGAGGACCGATGCTGATGTGG
GAGCCACCAGCGCTATATAAGGTTTTGCTATTCATTGAAAGCAGTAGTGACTGATTTGTATATA
>MinSyn_1140|Strength:0.001070521
GCGTGTCGTTTTAGTGAGGTCAGAAGATCAAAGGGCTACCTCTACAAAACTGGTCAGTGGTCCCTCCA
CTGTAGGCTATCATCTCTCTGCCGACAGTGGTCCCAAAACCACTATGCCCAATTAGGATCCTTACCGC
TATGGGTAAGATTTGAAAAAATGTCAAAGATAGCTAGGAGGCTTTGTCAAAAGCTAAAAAAGATGATG
CTAGGCAGCAAGTGGATGAGGACTCCATCAACAAATAATCCAAGTAAGTGTCTATATAAGGTTTTGCT
ATTCATTGAAAGCAGTAGTGACTGATTTGTATATA
>MinSyn_1678|Strength:0.001070648
GCGTGTCGTTTTAGTGAGGCAGTGGTCCCTCCACTTAGGTTGTCTGCAGGAAAAAGAAGAGGTTCTGC
ACCTCACATGAATTTCGGGAAACCTCCTCGCCGATGCTGATCTTGTGAAGCATCTTCCTCTACAGCTT
TGTCAAAAGCTAAAAAAGATGATGCAGGCTATCAGCTTAGCAAGACCTCTACTTATGACCCCCGCCGA
TGACGCGGGACTATGCCCAATTAGGTTGTCTGTCTCTCTGCCGACAGTGGTCCCAAACTGCTAGGAGG
ACCGATGCTTCAGAAGATCAAAGGGCTAACTATGCCCAATTAGGTTGTCTGCGCACACCAGCATGTGT
TGATCACCAGCTGTAGGTCACGACCTATATAAGGTTTTGCTATTCATTGAAAGCAGTAGTGACTGATT
TGTATATA
>MinSyn_1113|Strength:0.00107331
GCGTGTCGTTTTAGTGAGGAAAAATGTCAAAGATATCACTGCTAGGAGGACCGATGCTGAAACCACGT
CTACAATACAGGGCTTCTCTCTGCCGACAGTGGTCCCAAACTACAAAACTGGTACTTGTGTACAGGGC
TCCAGCCACTTGTGTGTACTTGTGTACAGGGCTCACTGCAAATATTTCTTGTAAGACCTCTACAAAAT
CCATCAACAAATAATCCAAGTAAGTCACTGCTAGGAGGACCGAGGTGGCTCCTACGTTGTCTGCACCT
CACATGAATTTCGGGAAACCTCCTCGGCAAGACCTCTACAAAACTATATAAGGTTTTGCTATTCATTG
AAAGCAGTAGTGACTGATTTGTATATA
>MinSyn_1022|Strength:0.001074265
GCGTGTCGTTTTAGTGAGGAACCATTATTGCGATCAGCTTAGCAAGACCTCTACAATTATGACCCCCG
CCGATGACGCGGGACTTAGCTTTGTCAAAAGCTAAAAAAGATGATGCGGTCCAAATATTTCTTGTTGT
GTACATCTCTCTGCCGACAGTGGTCCCAAACATGTAGGCTATCAGCTTAGCAACTATATAAGGTTTTG
CTATTCATTGAAAGCAGTAGTGACTGATTTGTATATA
>MinSyn_1616|Strength:0.001074662
GCGTGTCGTTTTAGTGAGGGCACACCAGCATGTGTTGATCACCAGCTCCTCTACAAAATTTTCAACAA
CTTGCCTGCCTTGATGATAAACCACGTCTACAAGCCCAATTAGGTTCTATATAAGGTTTTGCTATTCA
TTGAAAGCAGTAGTGACTGATTTGTATATA
>MinSyn_1429|Strength:0.001077025
GCGTGTCGTTTTAGTGAGGTGAAGATAAGATAATAATGTTGAAGATAAGAGCTCACTGCTAGGAGGAC
CGATGCTGATCTAACCACGTCTACAATGGGAAAAAGAAGAGGTTAGCAAGACCTCTACAAAACTGGTC
TATATAAGGTTTTGCTATTCATTGAAAGCAGTAGTGACTGATTTGTATATA
>MinSyn_1094|Strength:0.001078509
GCGTGTCGTTTTAGTGAGGAATTTCGGGAAACCTCCTCGTGCTGATCTTGCTTATGACCCCCGCCGAT
GACGCGGGACCTCACATGTAGGCTATCAGCTTACTATATAAGGTTTTGCTATTCATTGAAAGCAGTAG
TGACTGATTTGTATATA
>MinSyn_1160|Strength:0.001078689
GCGTGTCGTTTTAGTGAGGATCGAAAGGACAGTATCTACTCACTATCAGCAAACTGGTACTTGTGTAC
AGCTATATAAGGTTTTGCTATTCATTGAAAGCAGTAGTGACTGATTTGTATATA
>MinSyn_1629|Strength:0.001079957
GCGTGTCGTTTTAGTGAGGTCTCTCTGCCGACAGTGGTCCCAAAGTCTCAAATATTTCTTGTTACAGG
GCTCACTGGGAAAAAGAAGAGGTTACAAAACTGGTACAGCCACTTGTGTTGTCTGCACCTATCCTTAC
CGCTATGGGTAAGATTTAGGTTGTCTGTTTTCAACAATAATTTCGGGAAACCTCCTCGGACCTCTACA
AAACTGGTACTTGTGTCAGTGGTCCCTCCACACCTCTACAAAACTGGTACTTGTGTACAGCTATATAA
GGTTTTGCTATTCATTGAAAGCAGTAGTGACTGATTTGTATATA
>MinSyn_1793|Strength:0.001084549

GCGTGTCGTTTTAGTGAGGAACCATTATTGCGTCACTGCTAGGAGGACCGATGCTGATCTTGTTTTCA
ACAATGCCCAATTAGGTTGTCTCTCTCTGCCGACAGTGGTCCCAAACTATCAGCTTCTATATAAGGTT
TTGCTATTCATTGAAAGCAGTAGTGACTGATTTGTATATA
>MinSyn_1938|Strength:0.001084615
GCGTGTCGTTTTAGTGAGGGGAAAAAGAAGAGGTAGGCTAACCATTATTGCGTCTACAAAACTGGTAC
TTGTGTACAGCTATATAAGGTTTTGCTATTCATTGAAAGCAGTAGTGACTGATTTGTATATA
>MinSyn_1919|Strength:0.001085472
GCGTGTCGTTTTAGTGAGGATCGAAAGGACAGTAGCTAGGAGGACCGATGCTGTTATGACCCCCGCCG
ATGACGCGGGAGACAAATATTTCTTGTATGTAGGCTATCAGCTTAGCAAGAAGCAAGTGGATTGATCT
TGTGAAGCATCTTCCAGGTTGTCTGCACCTCACATGTAGGCTCCATCAACAAATAATCCAAGTAAGCA
AAACTATATAAGGTTTTGCTATTCATTGAAAGCAGTAGTGACTGATTTGTATATA
>MinSyn_1720|Strength:0.001089835
GCGTGTCGTTTTAGTGAGGTTTTCAACAACTGGTACTTGTGTATCGAAAGGACAGTATAGGTCACGAC
CGCTTTGTCAAAAGCTAAAAAAGATGATGCATTAGGTTGTCTGCACCTCACATGTAGGCTTGGTGGAG
CACGACATCAATTGCGATAAAGGAAAGGGGGCTCAAAAATGTCAAAGATACTGAAGATAAGATAATAA
TGTTGAAGATAAGAGCACCTCACATGTAGGCTATCAAGCAAGTGGATGACTATATAAGGTTTTGCTAT
TCATTGAAAGCAGTAGTGACTGATTTGTATATA
>MinSyn_1505|Strength:0.001090518
GCGTGTCGTTTTAGTGAGGAGGTGGCTCCTACTGCTAGGAGGACCGATGCTGATGAAGATAAGATAAT
AATGTTGAAGATAAGAGCTGTCAGAAGATCAAAGGGCTAAAACTGGTACTTGTGGGAAAAAGAAGAGG
TGTAGGCTATCAGCTTAGCAAGACCTTGAAGCATCTTCCTTGTGTACAGGGCTCACTGCTAGCAGCCA
CTTGTGTACCACTCTATATAAGGTTTTGCTATTCATTGAAAGCAGTAGTGACTGATTTGTATATA
>MinSyn_1695|Strength:0.001091509
GCGTGTCGTTTTAGTGAGGCAGCCACTTGTGTCTGCTAAACCATTATTGCGTACAAAATCCTTACCGC
TATGGGTAAGATTCTGCACCTCACATGTTGGTGGAGCACGACAGCTAGGAGGACCGATGCATTGCGAT
AAAGGAAAGGTCACATGTAGGCTATCAGCTTATGAAGCATCTTCCAGGTCACGACCACAGGTGGCTCC
TACAGGTCACGACCACCTATATAAGGTTTTGCTATTCATTGAAAGCAGTAGTGACTGATTTGTATATA
>MinSyn_1183|Strength:0.001092203
GCGTGTCGTTTTAGTGAGGTCTCTCTGCCGACAGTGGTCCCAAACTTGTGTACAGGGCTCACTGCTAG
GAGAATTTCGGGAAACCTCCTCGGGACCGATGCTATTGCGATAAAGGAAAGGATTAGGTTGTCTGCAC
CTCACATGTGGTGGAGCACGACAATGTAGGCTATCAGCAAAAATGTCAAAGATATCTACAAAACTGGT
ACTTGTAGGTGGCTCCTACATGCCCAATTAGGTTGTCTGCACCTCTGAAGATAAGATAATAATGTTGA
AGATAAGATACAGGGCTCACTGCTATATAAGGTTTTGCTATTCATTGAAAGCAGTAGTGACTGATTTG
TATATA
>MinSyn_1301|Strength:0.001093806
GCGTGTCGTTTTAGTGAGGAACCACGTCTACAAGATCTTGCCTGCCTTGATGAGCACACCAGCATGTG
TTGATCACCAGCTTTAGGTTGTCTGCACTATATAAGGTTTTGCTATTCATTGAAAGCAGTAGTGACTG
ATTTGTATATA
>MinSyn_1435|Strength:0.001095891
GCGTGTCGTTTTAGTGAGGTCCATCAACAAATAATCCAAGTAAGACAAAACTGGTACTTGTGTACACA
GCCACTTGTGTACGACCACTATGCCCAATTAGGTTGTATCGAAAGGACAGTACCGATGCTGATCTTGC
CTGCCTATCCTTACCGCTATGGGTAAGATTACTGGTACTTGTGTACAGGGCAACCATTATTGCGATCA
GCTTAGCAAGTGAAGATAAGATAATAATGTTGAAGATAAGAGCTAGGAGGACCGATGCTGATCTTGCC
TTCACTATCAGCCAAAACTGGTACTTGTGTAGGTGGCTCCTACGCTCGCACACCAGCATGTGTTGATC
ACCAGCTGCTTAGCAAGACCTCTACAAAACTGGTACTCTATATAAGGTTTTGCTATTCATTGAAAGCA
GTAGTGACTGATTTGTATATA
>MinSyn_1654|Strength:0.00109866
GCGTGTCGTTTTAGTGAGGGGAAAAAGAAGAGGTTGTACAGGGCTCACTGCTAGGAGCAAGTGGATCA
ATTGCACACCAGCATGTGTTGATCACCAGCTCTTGCCTAAGATTGATGAAAAGTCAAAAACAAAAATC
AATTATGGCTCACTGCTAGGAGGACCGATGCTGTTTTCAACAAGACCTCTACAAAACTGGTACTTGTG
AGGTGGCTCCTACCGCGGCTATATAAGGTTTTGCTATTCATTGAAAGCAGTAGTGACTGATTTGTATA
TA
>MinSyn_1125|Strength:0.001099116
GCGTGTCGTTTTAGTGAGGTCTCTCTGCCGACAGTGGTCCCAAACACGACCACTATGCCCAATTATCC

TTACCGCTATGGGTAAGATTTCTGCACCTCACATGTAGGCTATCAGATCGAAAGGACAGTAACTTGTG
TATTGCGATAAAGGAAAGGAGGGCTCACTGCTAGGAGGACCGCTATATAAGGTTTTGCTATTCATTGA
AAGCAGTAGTGACTGATTTGTATATA
>MinSyn_1389|Strength:0.001100543
GCGTGTCGTTTTAGTGAGGGGAAAAAGAAGAGGTGGTACTTGTGTACAGGGAAAAATGTCAAAGATAG
CTGATCTTGCCTGCGCACACCAGCATGTGTTGATCACCAGCTGGTTGTCTGCACCTCACACTATATAA
GGTTTTGCTATTCATTGAAAGCAGTAGTGACTGATTTGTATATA
>MinSyn_1247|Strength:0.001100585
GCGTGTCGTTTTAGTGAGGATCGAAAGGACAGTATGTGTACAGGGTCACTATCAGCTTGTGTACAGGG
CTCACTGCTAGGATTTTCAACAACACATGTAGGCTATCATCCATCAACAAATAATCCAAGTAAGAGGG
CTCACTGCTAGGAATTGCGATAAAGGAAAGGACAAAACTGGTACTTGTGTACAGCTATATAAGGTTTT
GCTATTCATTGAAAGCAGTAGTGACTGATTTGTATATA
>MinSyn_1615|Strength:0.001100901
GCGTGTCGTTTTAGTGAGGTCACTATCAGCGGTCACAACCATTATTGCGCACTGCTAGGCTATATAAG
GTTTTGCTATTCATTGAAAGCAGTAGTGACTGATTTGTATATA
>MinSyn_1765|Strength:0.001101563
GCGTGTCGTTTTAGTGAGGAACCATTATTGCGGACCGATGCTGATCTTGCCTGCCTTGACAGTGGTCC
CTCCACTCCTATATAAGGTTTTGCTATTCATTGAAAGCAGTAGTGACTGATTTGTATATA
>MinSyn_1836|Strength:0.00110268
GCGTGTCGTTTTAGTGAGGGGAAAAAGAAGAGGTCTGCACCTCACATGTAGGCTATCAGTGGTCCCTC
CACTGCCATTGCGATAAAGGAAAGGGTAGGCTATCAGCTTAGCAAGACCCTATATAAGGTTTTGCTAT
TCATTGAAAGCAGTAGTGACTGATTTGTATATA
>MinSyn_1453|Strength:0.001102726
GCGTGTCGTTTTAGTGAGGAAAAATGTCAAAGATATGTCTGCACCTCACATGGTGGAGCACGACAGGT
AGGTCACGACCATCGAAAGGACAGTATGGTACTTGTGTACAGGGCTCACTGATCCTTACCGCTATGGG
TAAGATTCTATCGCTTTGTCAAAAGCTAAAAAAGATGATGCAACTGGTACTTGTGTACAGGGCTCAAC
CACGTCTACAAGGGCTGCACACCAGCATGTGTTGATCACCAGCTGCCTATATAAGGTTTTGCTATTCA
TTGAAAGCAGTAGTGACTGATTTGTATATA
>MinSyn_1099|Strength:0.001102732
GCGTGTCGTTTTAGTGAGGAACCATTATTGCGTATGCCCAATTAGGTTGTCTGCACCTCTCACTATCA
GCTAGGTCACGACCACATCGAAAGGACAGTAACAAATATTTCTTGTGTACTCCATCAACAAATAATCC
AAGTAAGGCTCACTGCTAGGAGGAATCCTTACCGCTATGGGTAAGATTTGCTGATCTTGAGCAAGTGG
ATTAGGAGGACCGATGCTGGCACACCAGCATGTGTTGATCACCAGCTACTTGTGTACAGGGCTCACTG
CCTATATAAGGTTTTGCTATTCATTGAAAGCAGTAGTGACTGATTTGTATATA
>MinSyn_1528|Strength:0.001103357
GCGTGTCGTTTTAGTGAGGTCTCTCTGCCGACAGTGGTCCCAAAACCTCACATGTAGGCTATCAGCTT
ATCCTTACCGCTATGGGTAAGATTCACGATTATGACCCCCGCCGATGACGCGGGAGTACAGGGCTCAC
TGCTAGGAGGATCAGAAGATCAAAGGGCTACTCACATGTAGGCTATCCATCAACAAATAATCCAAGTA
AGTCTGCACCTCACATGTAGGCTATCAGCTTCAGTGGTCCCTCCACAGGCTATCAGCTTAGCAAGACC
TTGAAGATAAGATAATAATGTTGAAGATAAGAATCAGCTTAGCAAGACCTCTACAAAACTGCTATATA
AGGTTTTGCTATTCATTGAAAGCAGTAGTGACTGATTTGTATATA
>MinSyn_1005|Strength:0.00110521
GCGTGTCGTTTTAGTGAGGTGAAGATAAGATAATAATGTTGAAGATAAGACTTGTGTACAGGGCATCC
TTACCGCTATGGGTAAGATTACCACTATGCCCAATTAGGTTGTCTGCTATATAAGGTTTTGCTATTCA
TTGAAAGCAGTAGTGACTGATTTGTATATA
>MinSyn_1646|Strength:0.001105393
GCGTGTCGTTTTAGTGAGGAATTTCGGGAAACCTCCTCGACTTGTGTACAGGGCTCACTGCTAGAACC
ACGTCTACAATCTACAGGTGGCTCCTACTATGCCCAATTAGGTTGTCTGTCAGAAGATCAAAGGGCTA
GTAGGCTATCAGCTTAGCAAGACCCAAATATTTCTTGTCTGCACCAGCAAGTGGATACTCCATCAACA
AATAATCCAAGTAAGAAAACTGGTACTTGTGTACAGGGCTCTCTCTCTGCCGACAGTGGTCCCAAAGA
CCACTATGCCCAATAAAAATGTCAAAGATAGTTGTCTGCACCTCACATGCTATATAAGGTTTTGCTAT
TCATTGAAAGCAGTAGTGACTGATTTGTATATA
>MinSyn_1728|Strength:0.001105791
GCGTGTCGTTTTAGTGAGGTCTCTCTGCCGACAGTGGTCCCAAACCAATTAGGTTGTCTGCACCTCAT

CCTTACCGCTATGGGTAAGATTCCAGCCACTTGTGTTTGTCTGCACTGAAGATAAGATAATAATGTTG
AAGATAAGATTAGGTTGTCTGCACCTCACATGTAGGCATCGAAAGGACAGTACTAGGAGGACCGATGC
TGATCCTATATAAGGTTTTGCTATTCATTGAAAGCAGTAGTGACTGATTTGTATATA
>MinSyn_1766|Strength:0.001105882
GCGTGTCGTTTTAGTGAGGTGAAGCATCTTCCGCTGATCTTGGTGGGAGCCACCATCACTGCTAGGAC
TATATAAGGTTTTGCTATTCATTGAAAGCAGTAGTGACTGATTTGTATATA
>MinSyn_1251|Strength:0.00110594
GCGTGTCGTTTTAGTGAGGATGAGCTAAGCACATACGTCAGTCTTGCCAACCATTATTGCGTTGAAGA
TAAGATAATAATGTTGAAGATAAGAAGCAAGACCTCTACAAAACTGGTACTGGTGGAGCACGACACTC
ACTGCTAGGAGGACCGATGCTGATCAGGTGGCTCCTACAAGACCTCTACAAAACTGGTACTTGTGTAT
CGAAAGGACAGTAAGGCTATCACAGTGGTCCCTCCACTACAAGCTTTGTCAAAAGCTAAAAAAGATGA
TGCAATTAGGTTGTCTGCACCTCACACTATATAAGGTTTTGCTATTCATTGAAAGCAGTAGTGACTGA
TTTGTATATA
>MinSyn_1136|Strength:0.00110793
GCGTGTCGTTTTAGTGAGGAAAAATGTCAAAGATAAATTAGGTTGTCTGCACCTCACATGTCAGCCAC
TTGTGTCCAATTAGGTTATCGAAAGGACAGTATTAGCAAGACCTCTACATGGTGGAGCACGACAGCCC
TATATAAGGTTTTGCTATTCATTGAAAGCAGTAGTGACTGATTTGTATATA
>MinSyn_1386|Strength:0.001109961
GCGTGTCGTTTTAGTGAGGCAGCCACTTGTGTATTAGGTGGGAGCCACCACACGACCACTATGCCCCA
GTGGTCCCTCCACGTGTACAGGGCTCACTATATAAGGTTTTGCTATTCATTGAAAGCAGTAGTGACTG
ATTTGTATATA
>MinSyn_1993|Strength:0.001111612
GCGTGTCGTTTTAGTGAGGCAAATATTTCTTGTAAAACTGGTACTTGTGTACAGGGCTAAAAATGTCA
AAGATAGCCTTGATGAACCATTATTGCGGGCTCTATATAAGGTTTTGCTATTCATTGAAAGCAGTAGT
GACTGATTTGTATATA
>MinSyn_1794|Strength:0.001113158
GCGTGTCGTTTTAGTGAGGGCTTTGTCAAAAGCTAAAAAAGATGATGCACAACCACGTCTACAACAGG
GCTCACTGCTAGGAGGACCGATGCTGGTGGGAGCCACCACTTAGCATGAAGCATCTTCCAAGACCTAG
GTGGCTCCTACGTACACTATATAAGGTTTTGCTATTCATTGAAAGCAGTAGTGACTGATTTGTATATA
>MinSyn_1033|Strength:0.001114038
GCGTGTCGTTTTAGTGAGGTGGTGGAGCACGACATGTCTGCACCTCACATGTAGGCAAAAATGTCAAA
GATATTGTGTACAGGGCTCTCACTATCAGCTAAAGATTGATGAAAAGTCAAAAACAAAAATCAATTAT
GTAGGTCACGACCACTATGCCCAATTAAATTTCGGGAAACCTCCTCGATTAGGTTGTGAAGCATCTTC
CGCCCAATTAGGTTGTCAGCCACTTGTGTCTTGTGTACAGGGCTCACTGCTAGGAGGACGGAAAAAGA
AGAGGTGGAGGACCGATCTATATAAGGTTTTGCTATTCATTGAAAGCAGTAGTGACTGATTTGTATAT
A
>MinSyn_1393|Strength:0.001114074
GCGTGTCGTTTTAGTGAGGTCCATCAACAAATAATCCAAGTAAGGTTGTCTGCACCTCACATGGTGGA
GCACGACAAGGCTATCAGCTTAGCATCACTATCAGCTAGCAACTATATAAGGTTTTGCTATTCATTGA
AAGCAGTAGTGACTGATTTGTATATA
>MinSyn_1829|Strength:0.001115254
GCGTGTCGTTTTAGTGAGGTGAAGCATCTTCCAGGAGGTGGCTCCTACAGGCTATCAGCTTAGCAACT
ATATAAGGTTTTGCTATTCATTGAAAGCAGTAGTGACTGATTTGTATATA
>MinSyn_1417|Strength:0.001115557
GCGTGTCGTTTTAGTGAGGAAAAATGTCAAAGATATAGGTTGTCTGCACCTTGGTGGAGCACGACAGA
CCACTATGCCCAATTAGGTTGTCTGTCTCTCTGCCGACAGTGGTCCCAAATATGCCCAATTAGGTTAA
CCATTATTGCGCACCTCACATGTACTATATAAGGTTTTGCTATTCATTGAAAGCAGTAGTGACTGATT
TGTATATA
>MinSyn_1030|Strength:0.001118412
GCGTGTCGTTTTAGTGAGGTTATGACCCCCGCCGATGACGCGGGACTGCACCTCAGCAAGTGGATATC
AGAAGATCAAAGGGCTAACAAAACTGGTACAGGTGGCTCCTACGCCCAATTAAATTTCGGGAAACCTC
CTCGTCTACAAAACTGGTACTTGTGTACAGGGCTATATAAGGTTTTGCTATTCATTGAAAGCAGTAGT
GACTGATTTGTATATA
>MinSyn_1197|Strength:0.001118766

GCGTGTCGTTTTAGTGAGGTGGTGGAGCACGACAGCCCAATTAGGTTGTCTGCACCTCACAGCACACC
AGCATGTGTTGATCACCAGCTGGTCACTATCAGCCCGATGCTGATCTTGCCTGCCAGTGGTCCCTCCA
CGTAGGCTATCAGCTTCTATATAAGGTTTTGCTATTCATTGAAAGCAGTAGTGACTGATTTGTATATA
>MinSyn_1535|Strength:0.001119058
GCGTGTCGTTTTAGTGAGGAGGTGGCTCCTACTCTACAAAACTGCTTTGTCAAAAGCTAAAAAAGATG
ATGCGGTTGTCTGCACCTCACGCACACCAGCATGTGTTGATCACCAGCTACCTCTACTCTCTCTGCCG
ACAGTGGTCCCAAACTCATCACTATCAGCTGCTGGAAAAAGAAGAGGTCCTTGATGATCAGAAGATCA
AAGGGCTACCCAATTAGGTTGTCTGCACCTCAATTTCGGGAAACCTCCTCGGCTATCAGCTTAGCAAG
ACCTCTACAATTATGACCCCCGCCGATGACGCGGGATAGGCTATCAGCTTAGCAAGACCTCTACAACT
ATATAAGGTTTTGCTATTCATTGAAAGCAGTAGTGACTGATTTGTATATA
>MinSyn_1638|Strength:0.001120774
GCGTGTCGTTTTAGTGAGGTCAGAAGATCAAAGGGCTAAGCAAGACCTCTACAAAACTAACCACGTCT
ACAACTAGGAGGACCGATGCTGATCTTGCCTGCCAAAAATGTCAAAGATAACTGAAGATAAGATAATA
ATGTTGAAGATAAGATCAGCTTAGCAAGAGCTTTGTCAAAAGCTAAAAAAGATGATGCACTGGTACTT
GTGTACAGGGCTCACTGATCGAAAGGACAGTAGGTACTTGTGTACAGGGCTCCAGTGGTCCCTCCACA
CTGCTAGGAGGACCGATGCTGATCTTAAGATTGATGAAAAGTCAAAAACAAAAATCAATTATGTAGGT
CAATCCTTACCGCTATGGGTAAGATTGACCTCTACAAAACTGGTACTTGCTATATAAGGTTTTGCTAT
TCATTGAAAGCAGTAGTGACTGATTTGTATATA
>MinSyn_1713|Strength:0.001122618
GCGTGTCGTTTTAGTGAGGTCTCTCTGCCGACAGTGGTCCCAAACCAATTAGGTTGTCTGCACCTCAC
ATGTAGTCACTATCAGCTGTAGGCTAATCGAAAGGACAGTAGCACCTCACATGCACACCAGCATGTGT
TGATCACCAGCTTCACATGTAGGCTATCAGCTTAGCCAGCCACTTGTGTGTCTGCACCTCACATGTAG
GCTATCAGCTTGGTGGAGCACGACAATCTTGCCATTGCGATAAAGGAAAGGTCTTGCCTGCCTATATA
AGGTTTTGCTATTCATTGAAAGCAGTAGTGACTGATTTGTATATA
>MinSyn_1041|Strength:0.001123009
GCGTGTCGTTTTAGTGAGGTGGTGGAGCACGACACGATGCTGATTTTTCAACAATACTTGTCTATATA
AGGTTTTGCTATTCATTGAAAGCAGTAGTGACTGATTTGTATATA
>MinSyn_1355|Strength:0.001123894
GCGTGTCGTTTTAGTGAGGAAGATTGATGAAAAGTCAAAAACAAAAATCAATTATTAAAAATGTCAAA
GATAGTCACGACCACTATGCCCAATTAGGTTGGTGGAGCACGACATAGGCTATCAGCTTTCAGAAGAT
CAAAGGGCTAATCAAATATTTCTTGTTCTACAAAACTGGTACTCACTATCAGCATGCTGATCTTGCCT
GTGAAGCATCTTCCTTAGGTTGTCTGCACCTCACACTATATAAGGTTTTGCTATTCATTGAAAGCAGT
AGTGACTGATTTGTATATA
>MinSyn_1847|Strength:0.001124128
GCGTGTCGTTTTAGTGAGGATTGCGATAAAGGAAAGGGCAAGACCTCTAATCCTTACCGCTATGGGTA
AGATTGTGTACAGGGCTCACTGCTAGGAGGTCTCTCTGCCGACAGTGGTCCCAAAGCCTTGATGATAC
TATATAAGGTTTTGCTATTCATTGAAAGCAGTAGTGACTGATTTGTATATA
>MinSyn_1079|Strength:0.001125086
GCGTGTCGTTTTAGTGAGGTCACTATCAGCCTCTACAAAACTGGTACTTGTGTACATCCATCAACAAA
TAATCCAAGTAAGGACCGATGCTGATCTTAATTTCGGGAAACCTCCTCGGACCCAGTGGTCCCTCCAC
AAAACTGGTACTTGTGTACAGGGCTCACTCTATATAAGGTTTTGCTATTCATTGAAAGCAGTAGTGAC
TGATTTGTATATA
>MinSyn_1542|Strength:0.001127273
GCGTGTCGTTTTAGTGAGGATCGAAAGGACAGTACCTTCTCTCTGCCGACAGTGGTCCCAAATGTCTG
CACCTCACAATTGCGATAAAGGAAAGGGGTCACGACCACTATGCCCAATTAGGTTGTGAAGCATCTTC
CGCATCAGAAGATCAAAGGGCTATAGGTTGTCTGCACCTCACATGCTATATAAGGTTTTGCTATTCAT
TGAAAGCAGTAGTGACTGATTTGTATATA
>MinSyn_1361|Strength:0.001128668
GCGTGTCGTTTTAGTGAGGATCCTTACCGCTATGGGTAAGATTGTGTACAGCAGCCACTTGTGTAGGA
GGACCGATGCTAACCACGTCTACAAATTAGGTTGTCTGCACCAGTGGTCCCTCCACTACAGGGCTCAC
TCTATATAAGGTTTTGCTATTCATTGAAAGCAGTAGTGACTGATTTGTATATA
>MinSyn_1709|Strength:0.001130137
GCGTGTCGTTTTAGTGAGGATTGCGATAAAGGAAAGGGTAGGTCACGACCACTATGCCCAGCTTTGTC
AAAAGCTAAAAAAGATGATGCGCAAGACCTCTCAGTGGTCCCTCCACTGGTACTTGTGTACAGGGCAG

GTGGCTCCTACGCAAGACCTCTACAATGAAGATAAGATAATAATGTTGAAGATAAGATTAGGTTGTCT
GCACCTCACATGTTCAGAAGATCAAAGGGCTAGTGTACACTATATAAGGTTTTGCTATTCATTGAAAG
CAGTAGTGACTGATTTGTATATA
>MinSyn_1814|Strength:0.001130228
GCGTGTCGTTTTAGTGAGGTTATGACCCCCGCCGATGACGCGGGAAGGTCACGACCACTATGCAACCA
CGTCTACAATAGGTTGTCTGAAGATAAGATAATAATGTTGAAGATAAGATGCTGATCTTGCCTGCCTT
GATGAATCCTTACCGCTATGGGTAAGATTGTCTGCACCTCACATGTAGGCTATCAGCTCAAATATTTC
TTGTCAGCTTAGCAAGACCTCTACAAATGAAGCATCTTCCAGGTTGTCTGCACCTCAATTTCGGGAAA
CCTCCTCGCTCTACAAAGGTGGCTCCTACTTGTCTGCACCTCACTATATAAGGTTTTGCTATTCATTG
AAAGCAGTAGTGACTGATTTGTATATA
>MinSyn_1726|Strength:0.001131373
GCGTGTCGTTTTAGTGAGGTTTTCAACAAGACCACTATGCCCAATTAGATCCTTACCGCTATGGGTAA
GATTATGTAGGCTATCAGCTTAGCAAAGATTGATGAAAAGTCAAAAACAAAAATCAATTATGCCTTGA
TGATATCTCTCTGCCGACAGTGGTCCCAAAGTGTACAGGGCTCACTGCTAGGAGGACCTCAGAAGATC
AAAGGGCTACTGGTACTTGTGTGGAAAAAGAAGAGGTGGTCACGACCACTATGCCCAATTAGGTTGCT
ATATAAGGTTTTGCTATTCATTGAAAGCAGTAGTGACTGATTTGTATATA
>MinSyn_1999|Strength:0.001133957
GCGTGTCGTTTTAGTGAGGCAGTGGTCCCTCCACTCACTGCGCTTTGTCAAAAGCTAAAAAAGATGAT
GCCGATGCTGATCTTGCCTGCCTTGATCACTATCAGCCCGATGCTGATCTTGCCTGCCTTAATTTCGG
GAAACCTCCTCGAGATCCTTACCGCTATGGGTAAGATTAGGCTATCAGCTCAGAAGATCAAAGGGCTA
ACTATGCCCAATTAGGTTGTCCTATATAAGGTTTTGCTATTCATTGAAAGCAGTAGTGACTGATTTGT
ATATA
>MinSyn_1305|Strength:0.001134023
GCGTGTCGTTTTAGTGAGGTCCATCAACAAATAATCCAAGTAAGGTACAGGGCTCACTGCTAGAACCA
TTATTGCGCACTATGCCCAATTAGGTTGTCTGCACTTTTCAACAAGGAGGACCGATGCTGATCTTGGC
ACACCAGCATGTGTTGATCACCAGCTTACTTGTGTACAGGGCTCACTGCAACCACGTCTACAACTCAC
ATGTTTATGACCCCCGCCGATGACGCGGGACAAGACCTCTACCAGTGGTCCCTCCACTCAGCTTAGCA
AGACCTCTACAAAACCTATATAAGGTTTTGCTATTCATTGAAAGCAGTAGTGACTGATTTGTATATA
>MinSyn_1060|Strength:0.001134681
GCGTGTCGTTTTAGTGAGGAACCACGTCTACAACCTCTACAAAACTGGTACTTGTGTACTTTTCAACA
AGCCTGCCTTGATCAAATATTTCTTGTCTGGTACTTGTGTACAGGGCTCACTGCTATCTCTCTGCCGA
CAGTGGTCCCAAAGCTCTATATAAGGTTTTGCTATTCATTGAAAGCAGTAGTGACTGATTTGTATATA
>MinSyn_1306|Strength:0.001137132
GCGTGTCGTTTTAGTGAGGGTGGGAGCCACCACTGCTAGGAGGACCAATTTCGGGAAACCTCCTCGCT
ATCAGCATCGAAAGGACAGTACTCAGTGGTCCCTCCACGCTTAGCAAGACCTCTACAAAACTGGTACT
CTATATAAGGTTTTGCTATTCATTGAAAGCAGTAGTGACTGATTTGTATATA
>MinSyn_1156|Strength:0.001137203
GCGTGTCGTTTTAGTGAGGGCTTTGTCAAAAGCTAAAAAAGATGATGCGCACCTCACATGTAGGCTAT
CTCCATCAACAAATAATCCAAGTAAGACTATGCCCAATTAGGTTTCAGAAGATCAAAGGGCTAAGGGC
TCACTGCTAGGAGGACTCTCTCTGCCGACAGTGGTCCCAAATAGCAAGACCTCTACTATATAAGGTTT
TGCTATTCATTGAAAGCAGTAGTGACTGATTTGTATATA
>MinSyn_1733|Strength:0.001140441
GCGTGTCGTTTTAGTGAGGAAAAATGTCAAAGATAAGGTCACGACCACTATGCCCAATTAGGAAAAAG
AAGAGGTTGGTACTTGCTATATAAGGTTTTGCTATTCATTGAAAGCAGTAGTGACTGATTTGTATATA
>MinSyn_1084|Strength:0.001142361
GCGTGTCGTTTTAGTGAGGAATTTCGGGAAACCTCCTCGCTTAGCAAGACCTCTACAAAAGGTGGCTC
CTACAGGGCTCACTGCTAGGAGGACTATATAAGGTTTTGCTATTCATTGAAAGCAGTAGTGACTGATT
TGTATATA
>MinSyn_1478|Strength:0.001142794
GCGTGTCGTTTTAGTGAGGTTATGACCCCCGCCGATGACGCGGGAGCACCTCACATGTAGGCTATCAG
CTTAGCAGCCACTTGTGTCCTTGTGAAGCATCTTCCATGTACTATATAAGGTTTTGCTATTCATTGAA
AGCAGTAGTGACTGATTTGTATATA
>MinSyn_1469|Strength:0.001142949
GCGTGTCGTTTTAGTGAGGAAAAATGTCAAAGATATTGTCTGCACCTCACATGTATCAGAAGATCAAA

GGGCTAGTCTGCACCTCAGCCACTTGTGTCTATCAGCTTAGCAAGACCTAGCAAGTGGATTGGTACTT
GTGTACAGGGCTCCATCAACAAATAATCCAAGTAAGGCTCACTGCTAGGAGGACCGATGCTGATTCAC
TATCAGCGCGGTAGGTCAATCCTTACCGCTATGGGTAAGATTGTACTTGTGTACAGGGCAAGATTGAT
GAAAAGTCAAAAACAAAAATCAATTATATCTTGCCTGCCTTGATCTATATAAGGTTTTGCTATTCATT
GAAAGCAGTAGTGACTGATTTGTATATA
>MinSyn_1369|Strength:0.001144036
GCGTGTCGTTTTAGTGAGGGGAAAAAGAAGAGGTACGACCACTATGCCCAATTAGTCAGAAGATCAAA
GGGCTATGCTAGGAGGACCGAAGATTGATGAAAAGTCAAAAACAAAAATCAATTATGTGTACAGGGCT
CACTGCTAGGAGGACCGACTATATAAGGTTTTGCTATTCATTGAAAGCAGTAGTGACTGATTTGTATA
TA
>MinSyn_1813|Strength:0.001144756
GCGTGTCGTTTTAGTGAGGATCGAAAGGACAGTATGCTAGGAGGACCGATGCTGATCTTATGACCCCC
GCCGATGACGCGGGAACTGGTACTTGTAACCACGTCTACAATGTGGAAAAAGAAGAGGTGACCTCTAC
AAAACTGGTACTTGTCTATATAAGGTTTTGCTATTCATTGAAAGCAGTAGTGACTGATTTGTATATA
>MinSyn_1656|Strength:0.001145503
GCGTGTCGTTTTAGTGAGGGGAAAAAGAAGAGGTGATCTTGCCTGCCTCAGCCACTTGTGTTACTTGT
TATGACCCCCGCCGATGACGCGGGACAAGACCTCTACAAATCTCTCTGCCGACAGTGGTCCCAAAAGG
GCTCACTGCTAGGAGGCAGTGGTCCCTCCACCGATGCTGATCTAGGTGGCTCCTACCAGGGCTCACTG
CTAGGAGGACTATATAAGGTTTTGCTATTCATTGAAAGCAGTAGTGACTGATTTGTATATA
>MinSyn_1588|Strength:0.001145519
GCGTGTCGTTTTAGTGAGGTCACTATCAGCACAAAACTGGTAATCGAAAGGACAGTAGATGCTGGTGG
AGCACGACAACTTGTGTACTTATGACCCCCGCCGATGACGCGGGAGTCCATCAACAAATAATCCAAGT
AAGTAGCAAGACCTCATTGCGATAAAGGAAAGGACCTGCTTTGTCAAAAGCTAAAAAAGATGATGCTC
TTGCAACCATTATTGCGGCAAGACCTCTACAAAACGTGGGAGCCACCAGCCTTGACTATATAAGGTTT
TGCTATTCATTGAAAGCAGTAGTGACTGATTTGTATATA
>MinSyn_1090|Strength:0.001146956
GCGTGTCGTTTTAGTGAGGAGGTGGCTCCTACCGGTAGGTCACGAATCCTTACCGCTATGGGTAAGAT
TATGTAGGCTATCAGCTTAGCAAGACCTCAAAAATGTCAAAGATACAAGAGGAAAAAGAAGAGGTCCA
AATATTTCTTGTACCGATGCTGATCTTGAGCAAGTGGATGATCTTTTCAACAACATGTAGGCTAATCG
AAAGGACAGTATCTACAAAACTGGTACTTGTGTATGAAGATAAGATAATAATGTTGAAGATAAGAACT
GCTAGGAGGACTATATAAGGTTTTGCTATTCATTGAAAGCAGTAGTGACTGATTTGTATATA
>MinSyn_1364|Strength:0.001150221
GCGTGTCGTTTTAGTGAGGGTGGGAGCCACCAGACCACTATGCCCAATTAGGTTGTCTGCACAGCAAG
TGGATCTGCCTTGATGATTGGTGGAGCACGACACAGCTTAGCAAGACCTCTACAAAACTGGTAATTTC
GGGAAACCTCCTCGGCTCACTGCTAGGAGGATCGAAAGGACAGTAGATTTTTCAACAATTGCCTATTG
CGATAAAGGAAAGGCCAATTAGGTTCTATATAAGGTTTTGCTATTCATTGAAAGCAGTAGTGACTGAT
TTGTATATA
>MinSyn_1838|Strength:0.001150521
GCGTGTCGTTTTAGTGAGGTGAAGCATCTTCCCTATCAGCTTAGCAAAGGTGGCTCCTACACTTGTGT
ACAGGGCTCACTGCTAGGATCGAAAGGACAGTAGAGGACCGATGCTGATCTTGCTCTCTCTGCCGACA
GTGGTCCCAAATGCACCTCACATGTAGGCTATCAATCCTTACCGCTATGGGTAAGATTGACAAAAATG
TCAAAGATACGGTAGGTCACGACCACTATGCCCTATATAAGGTTTTGCTATTCATTGAAAGCAGTAGT
GACTGATTTGTATATA
>MinSyn_1350|Strength:0.001151157
GCGTGTCGTTTTAGTGAGGTTATGACCCCCGCCGATGACGCGGGATAGGAGGACCGATGCTGATCTTC
AGTGGTCCCTCCACATCAGCTTAGCAAGAAACCATTATTGCGCCTTCTATATAAGGTTTTGCTATTCA
TTGAAAGCAGTAGTGACTGATTTGTATATA
>MinSyn_1686|Strength:0.001152609
GCGTGTCGTTTTAGTGAGGAGGTGGCTCCTACAGGTGGTGGAGCACGACAATGCCCAATTAGGTTGTC
TGCTCACTATCAGCGTCTTGAAGATAAGATAATAATGTTGAAGATAAGAGCTCACTGCTAGGAGGAAT
CCTTACCGCTATGGGTAAGATTCACGACCACTATGCAAATATTTCTTGTTGTACAGGGCTTTTCAACA
ATGTACAGGGCTCACTGCTAGGAGGACTATATAAGGTTTTGCTATTCATTGAAAGCAGTAGTGACTGA
TTTGTATATA
>MinSyn_1100|Strength:0.001153486

GCGTGTCGTTTTAGTGAGGTTTTCAACAAACCACTATGCATTGCGATAAAGGAAAGGGGAGGACCGAT
GCTGATCTTGCCTGCTTATGACCCCCGCCGATGACGCGGGAAGGGCTCACTGCTAGGAGGACCGATCC
ATCAACAAATAATCCAAGTAAGGTACTTGGAAAAAGAAGAGGTGATCTTGCCTGCCTTGATGATGAAG
CATCTTCCACCACTATGCCCCTATATAAGGTTTTGCTATTCATTGAAAGCAGTAGTGACTGATTTGTA
TATA
>MinSyn_1272|Strength:0.001153486
GCGTGTCGTTTTAGTGAGGCAAATATTTCTTGTAGGCTATCAGGAAAAAGAAGAGGTCTCACTGCTAT
CACTATCAGCGCCCAATTAGGTTGTCTGCACCTCATGGTGGAGCACGACAGCGGTAGGTCACGACCAC
TATGCCCAATTATCCATCAACAAATAATCCAAGTAAGACTATATAAGGTTTTGCTATTCATTGAAAGC
AGTAGTGACTGATTTGTATATA
>MinSyn_1953|Strength:0.001154386
GCGTGTCGTTTTAGTGAGGTGAAGATAAGATAATAATGTTGAAGATAAGAGCAGTGGTCCCTCCACCT
GGTACTTGTGTACAGGGCTCTCACTATCAGCACCACTATGCCCAATTAGGTTGTCTCAAATATTTCTT
GTCTCACTCAGAAGATCAAAGGGCTAGGGCTCACTGCTAGGAGGACCGATGCTGATTTATGACCCCCG
CCGATGACGCGGGACTATCAGCTTAGCAAGACCTCTACAAGGAAAAAGAAGAGGTTGCACCTCGTGGG
AGCCACCATAGGTCTATATAAGGTTTTGCTATTCATTGAAAGCAGTAGTGACTGATTTGTATATA
>MinSyn_1903|Strength:0.001157778
GCGTGTCGTTTTAGTGAGGGCACACCAGCATGTGTTGATCACCAGCTATGTAGGCTATCAGCTAATTT
CGGGAAACCTCCTCGGCTGATCTTGCCTGCCTTGATGAGGTGGCTCCTACTCTTGCCTGCCTTGCTAT
ATAAGGTTTTGCTATTCATTGAAAGCAGTAGTGACTGATTTGTATATA
>MinSyn_1437|Strength:0.001160473
GCGTGTCGTTTTAGTGAGGTTATGACCCCCGCCGATGACGCGGGATCGCGGTAGGTCATCGAAAGGAC
AGTATGATCTTGCCTGCCTTGCAGTGGTCCCTCCACCGATGCTGATCTTGCCTGCCTTGATGATTGCG
ATAAAGGAAAGGAACTGGTACTTGTGTACAGGGCTCTCTCTCTGCCGACAGTGGTCCCAAAACAAATA
TTTCTTGTACTATGCCCAATTAGGTTGTCCTATATAAGGTTTTGCTATTCATTGAAAGCAGTAGTGAC
TGATTTGTATATA
>MinSyn_1209|Strength:0.001160608
GCGTGTCGTTTTAGTGAGGTCAGAAGATCAAAGGGCTATGCCTTGAGGAAAAAGAAGAGGTTTAGGTT
GTCTGCACTCCATCAACAAATAATCCAAGTAAGCGGTATGAAGCATCTTCCTTAGCAAGACCTCTACA
AAACTGGTACTTGTTATGACCCCCGCCGATGACGCGGGACTGCTAGGAAACCATTATTGCGACATGTA
GCACACCAGCATGTGTTGATCACCAGCTACAAAACTGGTACTTGTGTACAGGGCAAAAATGTCAAAGA
TATGCACCTCACATGTAGCAGTGGTCCCTCCACTACAGGGCTCACTGCTAGGAGGACCCTATATAAGG
TTTTGCTATTCATTGAAAGCAGTAGTGACTGATTTGTATATA
>MinSyn_1735|Strength:0.001162694
GCGTGTCGTTTTAGTGAGGCAAATATTTCTTGTATCTTGCCTGCCTTGATGATCCTTACCGCTATGGG
TAAGATTTAAGATTGATGAAAAGTCAAAAACAAAAATCAATTATCTGCTAGGAGGACCGATGCTGATC
AGGTGGCTCCTACACATGTAGGCTATCAGTCAGAAGATCAAAGGGCTAAGCTTAGCAAGACCTCTAAC
CACGTCTACAAGTACTTGTGTACAGGGCTCACTGCTAGTGAAGATAAGATAATAATGTTGAAGATAAG
AAGCTTAGCAAGACCTCTACCTATATAAGGTTTTGCTATTCATTGAAAGCAGTAGTGACTGATTTGTA
TATA
>MinSyn_1624|Strength:0.001163901
GCGTGTCGTTTTAGTGAGGTGAAGATAAGATAATAATGTTGAAGATAAGACAGGTGGCTCCTACCACC
TCACATGTAGGCTAGCAAGTGGATGTACAGGGCTCACTGCTAGGAGGACCAACCATTATTGCGCTCAC
ATGTAGGTCTCTCTGCCGACAGTGGTCCCAAAGCTCACTGCTAGGAGGACCGATGCAATTTCGGGAAA
CCTCCTCGAAGACCCTATATAAGGTTTTGCTATTCATTGAAAGCAGTAGTGACTGATTTGTATATA
>MinSyn_1289|Strength:0.001165272
GCGTGTCGTTTTAGTGAGGATCGAAAGGACAGTAAGCTTAGCAAGACCTCTACAAATCCTTACCGCTA
TGGGTAAGATTCCGATGCTGATAAGATTGATGAAAAGTCAAAAACAAAAATCAATTATGACCGATGCT
GATCTTGCCTTTATGACCCCCGCCGATGACGCGGGACTGCTAGGAGGACCGATGCTGATCTTGCCCTA
TATAAGGTTTTGCTATTCATTGAAAGCAGTAGTGACTGATTTGTATATA
>MinSyn_1563|Strength:0.001165289
GCGTGTCGTTTTAGTGAGGCAGTGGTCCCTCCACTGCTGATAGGTGGCTCCTACATCTTGCCTGCCTT
GCTATATAAGGTTTTGCTATTCATTGAAAGCAGTAGTGACTGATTTGTATATA
>MinSyn_1773|Strength:0.0011656

GCGTGTCGTTTTAGTGAGGAACCATTATTGCGTTGCCTGCCTTAAAAATGTCAAAGATAACTGCTAGG
AGGACCTATATAAGGTTTTGCTATTCATTGAAAGCAGTAGTGACTGATTTGTATATA
>MinSyn_1392|Strength:0.001166774
GCGTGTCGTTTTAGTGAGGGCACACCAGCATGTGTTGATCACCAGCTTATCAGCTTAGCAAGACCAAA
AATGTCAAAGATACCCAATTAGGTTGTCTGCACCTCACATTTTTCAACAAGTACCAGTGGTCCCTCCA
CGGTCACGACCACTATGCCCAATTAGTGAAGATAAGATAATAATGTTGAAGATAAGACTTGTGAACCA
TTATTGCGAACTGGTACTTGTGTACAGGTGGGAGCCACCAACTTGTGTACAGCTATATAAGGTTTTGC
TATTCATTGAAAGCAGTAGTGACTGATTTGTATATA
>MinSyn_1025|Strength:0.001168407
GCGTGTCGTTTTAGTGAGGAGCAAGTGGATTTGATGATTGGTGGAGCACGACAGCTCACTGCTCCATC
AACAAATAATCCAAGTAAGGCTTAGCAAGACCTCTACAAAACTTGAAGCATCTTCCCTCACTGCTAGG
AGGACCGATGCTGATCTTAAGATTGATGAAAAGTCAAAAACAAAAATCAATTATCACTATGCTCACTA
TCAGCTGATCATTGCGATAAAGGAAAGGTGATCTTGCCTGGGAAAAAGAAGAGGTCTGCTAGGAGGAC
CGATGCTGATCTTGCCTCTATATAAGGTTTTGCTATTCATTGAAAGCAGTAGTGACTGATTTGTATAT
A
>MinSyn_1501|Strength:0.001168911
GCGTGTCGTTTTAGTGAGGCAAATATTTCTTGTATCAGCAGGTGGCTCCTACGATCTTGCCTGCCTTG
AAAGATTGATGAAAAGTCAAAAACAAAAATCAATTATGTAGGCTATCAGCTTAGCAAGACATTGCGAT
AAAGGAAAGGGCTGATCTTGCCTGCCTTGATGATCAGAAGATCAAAGGGCTAAAGCAAGTGGATGCCC
ACTATATAAGGTTTTGCTATTCATTGAAAGCAGTAGTGACTGATTTGTATATA
>MinSyn_1642|Strength:0.001171816
GCGTGTCGTTTTAGTGAGGTCCATCAACAAATAATCCAAGTAAGTTAGCACAGCCACTTGTGTGTTGT
CAAATATTTCTTGTAAGACCTCTACAAAACTGGTACTTGAGGTGGCTCCTACCTGCCTTGATCAGAAG
ATCAAAGGGCTAGGCTCACTGCTAGGAGCTATATAAGGTTTTGCTATTCATTGAAAGCAGTAGTGACT
GATTTGTATATA
>MinSyn_1180|Strength:0.001171913
GCGTGTCGTTTTAGTGAGGTCACTATCAGCCACTGCTAGGAGGACCGATGCTCAGAAGATCAAAGGGC
TACTCTACAATGGTGGAGCACGACAATGCCCAATTAGGCTATATAAGGTTTTGCTATTCATTGAAAGC
AGTAGTGACTGATTTGTATATA
>MinSyn_1843|Strength:0.001172035
GCGTGTCGTTTTAGTGAGGAGGTGGCTCCTACCTAAAAATGTCAAAGATACTTGCGGAAAAAGAAGAG
GTCACCTCACATGTAGGCTATCAAACCACGTCTACAAACAAAACTGGTACTTGTGTACAGGGCTCAAA
GATTGATGAAAAGTCAAAAACAAAAATCAATTATACGACCACTATGTGGGAGCCACCAAACTGGTACT
TGTGTACAGGGCTCACTGCTCTATATAAGGTTTTGCTATTCATTGAAAGCAGTAGTGACTGATTTGTA
TATA
>MinSyn_1312|Strength:0.001172528
GCGTGTCGTTTTAGTGAGGCAAATATTTCTTGTCAAAACTCCATCAACAAATAATCCAAGTAAGTTAG
GTTGTAGGTGGCTCCTACCTCACATGTAGGCTATCAGCTTAGCAAAATTTCGGGAAACCTCCTCGCTC
ACTGCTAGGAGGACTCTCTCTGCCGACAGTGGTCCCAAACACGAACCACGTCTACAATACAAAACTGG
TACTTGTGTACAGGGCTCACTATATAAGGTTTTGCTATTCATTGAAAGCAGTAGTGACTGATTTGTAT
ATA
>MinSyn_1150|Strength:0.001173184
GCGTGTCGTTTTAGTGAGGTCTCTCTGCCGACAGTGGTCCCAAAATGCCTGAAGATAAGATAATAATG
TTGAAGATAAGACTGGTACCAGTGGTCCCTCCACACCTCAATTTCGGGAAACCTCCTCGTAGGAGCAC
ACCAGCATGTGTTGATCACCAGCTTTGCCTGCCTTGATGATAAGCAAGTGGATCTACAAAACTGGTAC
TTGTGTACAGCTATATAAGGTTTTGCTATTCATTGAAAGCAGTAGTGACTGATTTGTATATA
>MinSyn_1144|Strength:0.001173861
GCGTGTCGTTTTAGTGAGGAAGATTGATGAAAAGTCAAAAACAAAAATCAATTATGTAGGCTATCAGC
TTAGCAAGTCTCTCTGCCGACAGTGGTCCCAAATGCTGATCTTGCCTGCCTTCTATATAAGGTTTTGC
TATTCATTGAAAGCAGTAGTGACTGATTTGTATATA
>MinSyn_1704|Strength:0.001175
GCGTGTCGTTTTAGTGAGGCAGCCACTTGTGTACTTGTGTACAGGGCTCAAAAAATGTCAAAGATAGC
CTTGCTATATAAGGTTTTGCTATTCATTGAAAGCAGTAGTGACTGATTTGTATATA
>MinSyn_1904|Strength:0.001175894

GCGTGTCGTTTTAGTGAGGATTGCGATAAAGGAAAGGTAGGTTGTCTGCACCTTATGACCCCCGCCGA
TGACGCGGGAATGTAGGCTATCAGCTTAGCAAGAATCCTTACCGCTATGGGTAAGATTCCACTATGCC
CAATTTCCATCAACAAATAATCCAAGTAAGGAGGAGCACACCAGCATGTGTTGATCACCAGCTGCTAG
GAGGACCGATGCTGATCTTTTTTCAACAAGCCCAATTAGGTTGTCTGCACCTCACATGTCACTATCAG
CCCTATATAAGGTTTTGCTATTCATTGAAAGCAGTAGTGACTGATTTGTATATA
>MinSyn_1145|Strength:0.001176612
GCGTGTCGTTTTAGTGAGGTGAAGCATCTTCCTTAGGTTGTCTGCACCTCACATCCTTACCGCTATGG
GTAAGATTGGGCTCACTGCTAGGAGGACCGATGCAATTTCGGGAAACCTCCTCGCAGGGCTCACTTGA
AGATAAGATAATAATGTTGAAGATAAGAATGCTGATCTTGCCTGCCTCTATATAAGGTTTTGCTATTC
ATTGAAAGCAGTAGTGACTGATTTGTATATA
>MinSyn_1486|Strength:0.001179716
GCGTGTCGTTTTAGTGAGGTTTTCAACAATAGGTCACGTGGGAGCCACCAAGGTCACGACCACTATGC
GCACACCAGCATGTGTTGATCACCAGCTCTTGCCTAATTTCGGGAAACCTCCTCGACTATGCCCAATT
AGGTTGTCTGCACCAACCACGTCTACAATCGCGGTAGGTCACAAAAATGTCAAAGATACATGTAGGTG
GCTCCTACTACTTGTGTACAGGGGAAAAAGAAGAGGTTCGCGGTAGTGGTGGAGCACGACAACTTGTG
TACAGGGCTCACTGCTATATAAGGTTTTGCTATTCATTGAAAGCAGTAGTGACTGATTTGTATATA
>MinSyn_1128|Strength:0.001184252
GCGTGTCGTTTTAGTGAGGGGAAAAAGAAGAGGTACCACTATGCCCAATTAGGTTGTTGGTGGAGCAC
GACACTCCTATATAAGGTTTTGCTATTCATTGAAAGCAGTAGTGACTGATTTGTATATA
>MinSyn_1297|Strength:0.001184755
GCGTGTCGTTTTAGTGAGGTGAAGCATCTTCCGCGGTAGGTCAATCCTTACCGCTATGGGTAAGATTT
GCTAGGAGGACCGATGCTGAAAGATTGATGAAAAGTCAAAAACAAAAATCAATTATTACAAAACAGCA
AGTGGATCTAGGAGGACCGATGCTGATCTTGCGCACACCAGCATGTGTTGATCACCAGCTACCTCTAC
AAAACTGGTACTTGTGTAATTTCGGGAAACCTCCTCGATGTAGGCTATCTATATAAGGTTTTGCTATT
CATTGAAAGCAGTAGTGACTGATTTGTATATA
>MinSyn_1360|Strength:0.001184792
GCGTGTCGTTTTAGTGAGGGCACACCAGCATGTGTTGATCACCAGCTACCTCTACAAAATGAAGATAA
GATAATAATGTTGAAGATAAGATCTACAAAACTGGTACTTGTGTACAGGGCTCTATATAAGGTTTTGC
TATTCATTGAAAGCAGTAGTGACTGATTTGTATATA
>MinSyn_1016|Strength:0.001185336
GCGTGTCGTTTTAGTGAGGAAAAATGTCAAAGATAACTATGCCCAATTAGTGGTGGAGCACGACAACA
AATATTTCTTGTACCTCTACAAAACTGGTACTTGTGTAACCACGTCTACAAATAATTGCGATAAAGGA
AAGGTAGGAGGACCGATGCTGATCTTGCCTGCCATCCTTACCGCTATGGGTAAGATTGTCTGCCAGTG
GTCCCTCCACAGCTTAGTCCATCAACAAATAATCCAAGTAAGCACTGCTAGGAGGACCGATGCTGATC
TTGCTATATAAGGTTTTGCTATTCATTGAAAGCAGTAGTGACTGATTTGTATATA
>MinSyn_1496|Strength:0.001185586
GCGTGTCGTTTTAGTGAGGCAGTGGTCCCTCCACCCGATGCTAAAAATGTCAAAGATACACTATGCCC
AATTAGGTTGAGCAAGTGGATCTATGCCCATTATGACCCCCGCCGATGACGCGGGAAGGGCTCACTGT
CAGAAGATCAAAGGGCTAAGCAAGACCTCTACAACTATATAAGGTTTTGCTATTCATTGAAAGCAGTA
GTGACTGATTTGTATATA
>MinSyn_1187|Strength:0.001186972
GCGTGTCGTTTTAGTGAGGTGAAGATAAGATAATAATGTTGAAGATAAGACTCACATGTAGGCTATCA
GCCAGTGGTCCCTCCACGGCACACCAGCATGTGTTGATCACCAGCTATCAGCTTAGCAAGACGCTTTG
TCAAAAGCTAAAAAAGATGATGCCTAGGAGGACCGATGCTGATCATTGCGATAAAGGAAAGGACAGGG
CTCACTGCTAGGAAGATTGATGAAAAGTCAAAAACAAAAATCAATTATTAGGCTATCAGCTTAGCAAG
ACCAATTTCGGGAAACCTCCTCGCAATTAGGTTGTCTATATAAGGTTTTGCTATTCATTGAAAGCAGT
AGTGACTGATTTGTATATA
>MinSyn_1450|Strength:0.001187181
GCGTGTCGTTTTAGTGAGGAAAAATGTCAAAGATAAGGAGGACCTCAGAAGATCAAAGGGCTACAAGA
CCTCTACAAAACTAATTTCGGGAAACCTCCTCGGTCTGATTGCGATAAAGGAAAGGATGCCCAATTAG
GTTGTCTGCAATCGAAAGGACAGTAGCGGTAGGTCACGACCTATATAAGGTTTTGCTATTCATTGAAA
GCAGTAGTGACTGATTTGTATATA
>MinSyn_1658|Strength:0.00118726
GCGTGTCGTTTTAGTGAGGGCACACCAGCATGTGTTGATCACCAGCTTGCCCAATTAGGTTGTCTGCA

CCTCACTCCATCAACAAATAATCCAAGTAAGCTATCAGCTTAGCAAGACCTCTACAAAACAATTTCGG
GAAACCTCCTCGATTAGGTTTCACTATCAGCTACTTGTGAAGATTGATGAAAAGTCAAAAACAAAAAT
CAATTATCGCGGTAGGTCACGACCACTATGCCCTTTTCAACAAACCACTATGCCCTTATGACCCCCGC
CGATGACGCGGGAGCAAGACCTCTACAAAACTGGTACTTGTATCCTTACCGCTATGGGTAAGATTCAA
TTAGGTTGTCTCAAATATTTCTTGTACGACCACTATGCCCAATTAGCTATATAAGGTTTTGCTATTCA
TTGAAAGCAGTAGTGACTGATTTGTATATA
>MinSyn_1968|Strength:0.001187915
GCGTGTCGTTTTAGTGAGGAGGTGGCTCCTACTAGGTTGTCTGCTCAGAAGATCAAAGGGCTAGGGCT
CACTCAGTGGTCCCTCCACCTAGGAGGACCGATGCTGATCTATATAAGGTTTTGCTATTCATTGAAAG
CAGTAGTGACTGATTTGTATATA
>MinSyn_1567|Strength:0.00119055
GCGTGTCGTTTTAGTGAGGGTGGGAGCCACCACTCTACAAAACTGGTACTTGTAAAAATGTCAAAGAT
AGTCTGCAATTTCGGGAAACCTCCTCGATGCCCAATTAGGTTGTCTGCGCACACCAGCATGTGTTGAT
CACCAGCTGTTCTCTCTGCCGACAGTGGTCCCAAATGGTACTTGTGTACAGGGCTCACTGCTGAAGCA
TCTTCCTAGGTTGTCTGCACCTCACATGTAGTGAAGATAAGATAATAATGTTGAAGATAAGACACTAT
GCCCAATTAGGTTGTCGGAAAAAGAAGAGGTGGCTCACTGCTAGGAGGACCCTATATAAGGTTTTGCT
ATTCATTGAAAGCAGTAGTGACTGATTTGTATATA
>MinSyn_1132|Strength:0.00119218
GCGTGTCGTTTTAGTGAGGAACCACGTCTACAAAGGTCACGACCACTATGCCCAATGTGGGAGCCACC
AGCTGATCTTGCCCAAATATTTCTTGTGATGCACACCAGCATGTGTTGATCACCAGCTCGGTAGGTCA
CGACCACAATTTCGGGAAACCTCCTCGACCTCACATGTAGGCTATCAGCTTAGAGGTGGCTCCTACAG
CTTAGCAAGATCCTTACCGCTATGGGTAAGATTCTGCTAGGAGGACCGATGCTGATCAGAAGATCAAA
GGGCTACGACCACTATTTTTCAACAATCTTGCCTGCCCTATATAAGGTTTTGCTATTCATTGAAAGCA
GTAGTGACTGATTTGTATATA
>MinSyn_1867|Strength:0.001193813
GCGTGTCGTTTTAGTGAGGCAAATATTTCTTGTACCAAATTTCGGGAAACCTCCTCGCTGATCTTGCC
TGCCTTGATGATATTTTCAACAAATGCCCAATTAGGTTGTCTGCACCTCACGGAAAAAGAAGAGGTGC
TATCAGCTTAGCAAGATGAAGCATCTTCCTATCAGCTTAGCAATCACTATCAGCAGGGCTCACTGCTA
TCTCTCTGCCGACAGTGGTCCCAAACCAATTAGGAAAAATGTCAAAGATATGCTAGGAGGACCGATGC
TGATATTGCGATAAAGGAAAGGGCTATATAAGGTTTTGCTATTCATTGAAAGCAGTAGTGACTGATTT
GTATATA
>MinSyn_1601|Strength:0.001194577
GCGTGTCGTTTTAGTGAGGCAGTGGTCCCTCCACGGGCTCAACCACGTCTACAAGACCTCTACAAAAC
TGGTACTTGTGTACAGAAAAAATGTCAAAGATACTATGCCTTTTCAACAATGCCCAAAACCATTATTGC
GTCACATGTAGGCCTATATAAGGTTTTGCTATTCATTGAAAGCAGTAGTGACTGATTTGTATATA
>MinSyn_1493|Strength:0.001194786
GCGTGTCGTTTTAGTGAGGTCACTATCAGCTGGTACTTGTGTACAGCAGCCACTTGTGTTGATCAGTG
GTCCCTCCACTAGGCTATCAGCTTAAAAAATGTCAAAGATAATCTTGCCTAATTTCGGGAAACCTCCT
CGACGACCACTATGCCCAATTAGGTTGTCTGCACACCAGCATGTGTTGATCACCAGCTCACTATGCCT
ATATAAGGTTTTGCTATTCATTGAAAGCAGTAGTGACTGATTTGTATATA
>MinSyn_1357|Strength:0.001196069
GCGTGTCGTTTTAGTGAGGAATTTCGGGAAACCTCCTCGATGCTGATCAGTGGTCCCTCCACGTACAG
GGCTCACTGCTAGGAGGACTGAAGCATCTTCCAGGAGGACCGATTCTCTCTGCCGACAGTGGTCCCAA
AACCTCTACAAAACTGCTATATAAGGTTTTGCTATTCATTGAAAGCAGTAGTGACTGATTTGTATATA
>MinSyn_1271|Strength:0.001196343
GCGTGTCGTTTTAGTGAGGTTTTCAACAACCCAATTAGGTTGTCTGCACCTCAAATTTCGGGAAACCT
CCTCGTAGCAAGACCTCTCAAATATTTCTTGTGGACCGATTTATGACCCCCGCCGATGACGCGGGACT
GGTACTTGTGTACAAGGTGGCTCCTACAATTAGGTTGTCTGGTGGGAGCCACCATATGCCCCTATATA
AGGTTTTGCTATTCATTGAAAGCAGTAGTGACTGATTTGTATATA
>MinSyn_1089|Strength:0.001203319
GCGTGTCGTTTTAGTGAGGTCTCTCTGCCGACAGTGGTCCCAAAGCACCTCATTTTCAACAATTGTGT
ACAGGGCTCACTGCTAGGAGGACATCCTTACCGCTATGGGTAAGATTTCTACAAAACTGGTACTTGTG
TACTGAAGATAAGATAATAATGTTGAAGATAAGATTAGGTTGTCTGCACCTCACATGTAGCACACCAG
CATGTGTTGATCACCAGCTCTTGTGTACCTATATAAGGTTTTGCTATTCATTGAAAGCAGTAGTGACT

GATTTGTATATA
>MinSyn_1385|Strength:0.001203817
GCGTGTCGTTTTAGTGAGGTTTTCAACAAGCCTGCCTTGATCCATCAACAAATAATCCAAGTAAGGCT
TAGCAAGACCTCTGTGGGAGCCACCATGGTACTTGTGTACAGGGCTCCTATATAAGGTTTTGCTATTC
ATTGAAAGCAGTAGTGACTGATTTGTATATA
>MinSyn_1010|Strength:0.001203877
GCGTGTCGTTTTAGTGAGGTGAAGCATCTTCCCTACAAAACTGGTACTTGTGTACAGAGGTGGCTCCT
ACCACTATGCCCAATTAGGTTGTCCAGCCACTTGTGTCTGCTAGGAGGACCGTGAAGATAAGATAATA
ATGTTGAAGATAAGACGCGGTAGGTCCTATATAAGGTTTTGCTATTCATTGAAAGCAGTAGTGACTGA
TTTGTATATA
>MinSyn_1427|Strength:0.0012047
GCGTGTCGTTTTAGTGAGGAACCACGTCTACAAACAAAACTGGTACTTGTGTACAGGGCTCACAGGTG
GCTCCTACGGACCGATGCTGATCTTGCCTGTTTTCAACAATGTACAGGGGGAAAAAGAAGAGGTAGGT
GGTGGAGCACGACAATCTTCTATATAAGGTTTTGCTATTCATTGAAAGCAGTAGTGACTGATTTGTAT
ATA
>MinSyn_1670|Strength:0.001204723
GCGTGTCGTTTTAGTGAGGATTGCGATAAAGGAAAGGAGGGCTCACTGCTAGAACCATTATTGCGCTC
ACTGCTAGGACAGTGGTCCCTCCACCCTCTACAAAACTGGTACTTGTGTACAGCAAATATTTCTTGTG
GAGGACCGATGCTGATCTATCGAAAGGACAGTAATGTAGGCTATCTCCATCAACAAATAATCCAAGTA
AGTGCTATATAAGGTTTTGCTATTCATTGAAAGCAGTAGTGACTGATTTGTATATA
>MinSyn_1791|Strength:0.001206829
GCGTGTCGTTTTAGTGAGGAACCACGTCTACAAAACTGGTACTTGTGTACAGGGCTCACTGAGCAAGT
GGATAGGTTGTCTGCACCTCAGCTTTGTCAAAAGCTAAAAAAGATGATGCACCTCACATGTTCTCTCT
GCCGACAGTGGTCCCAAACCTCTACTCACTATCAGCCCTGCCTTGATGATTCCATCAACAAATAATCC
AAGTAAGCTAGGAGGACCGATGCTGATCTTGCCTGAAGATTGATGAAAAGTCAAAAACAAAAATCAAT
TATTGCACCTCACAGGTGGCTCCTACGTGTCTATATAAGGTTTTGCTATTCATTGAAAGCAGTAGTGA
CTGATTTGTATATA
>MinSyn_1191|Strength:0.001207204
GCGTGTCGTTTTAGTGAGGGCACACCAGCATGTGTTGATCACCAGCTTCTACAAAACTGGTACTTGTG
CAAATATTTCTTGTGTTGTCTGCACCTCACAAGGTGGCTCCTACCGTCTCTCTGCCGACAGTGGTCCC
AAACTTGTGTACAGGGCTCACTAACCACGTCTACAATAGGAGGACCGATGCTGATCCTATATAAGGTT
TTGCTATTCATTGAAAGCAGTAGTGACTGATTTGTATATA
>MinSyn_1339|Strength:0.001207311
GCGTGTCGTTTTAGTGAGGCAGTGGTCCCTCCACGGCTATCCATCAACAAATAATCCAAGTAAGGGAC
CGATGCTGATCTTGCCTGCCTTGAAATTTCGGGAAACCTCCTCGCTATGCCCAATTATCAGAAGATCA
AAGGGCTAGATGAGCAAGTGGATTACTATATAAGGTTTTGCTATTCATTGAAAGCAGTAGTGACTGAT
TTGTATATA
>MinSyn_1699|Strength:0.001208571
GCGTGTCGTTTTAGTGAGGTCAGAAGATCAAAGGGCTACAATTAGGTTGGAAAAAGAAGAGGTCAAGA
CCTCTACAAAACTGGTACTTCTATATAAGGTTTTGCTATTCATTGAAAGCAGTAGTGACTGATTTGTA
TATA
>MinSyn_1880|Strength:0.001209116
GCGTGTCGTTTTAGTGAGGGGAAAAAGAAGAGGTAAGACCTCTACAAAACTGGTACAGCCACTTGTGT
GAGGGTGGGAGCCACCAGGAGGACCGATCCATCAACAAATAATCCAAGTAAGCCTCACATGTAGGCTA
TCAGCAAGATTGATGAAAAGTCAAAAACAAAAATCAATTATTGCTGATCTTGCCTGCCTTGATGATAC
TATATAAGGTTTTGCTATTCATTGAAAGCAGTAGTGACTGATTTGTATATA
>MinSyn_1581|Strength:0.001211663
GCGTGTCGTTTTAGTGAGGAATTTCGGGAAACCTCCTCGGCCTGCCTTCAGCCACTTGTGTACCTCTA
CAGGAAAAAGAAGAGGTGTTGTCTGGTGGGAGCCACCAGCTATCAGCTTAGCAAGACCTCTTGAAGAT
AAGATAATAATGTTGAAGATAAGATAGCAAATATTTCTTGTCTATGCCCAATTAGGTTGTCATCCTTA
CCGCTATGGGTAAGATTTGTACAGGGCTCACTGCTAGGCTATATAAGGTTTTGCTATTCATTGAAAGC
AGTAGTGACTGATTTGTATATA
>MinSyn_1127|Strength:0.001214045
GCGTGTCGTTTTAGTGAGGAAAAATGTCAAAGATATCTGCACCTCACATGTAGGCTATCAGCAGGTGG

CTCCTACACCGATGTGGGAGCCACCAGGACCGATGCTGATAAGATTGATGAAAAGTCAAAAACAAAAA
TCAATTATCTGGTACTTGTGTACAGGCTATATAAGGTTTTGCTATTCATTGAAAGCAGTAGTGACTGA
TTTGTATATA
>MinSyn_1348|Strength:0.001218745
GCGTGTCGTTTTAGTGAGGGTGGGAGCCACCATGTGTACAGGGCTAAGATTGATGAAAAGTCAAAAAC
AAAAATCAATTATGCTAGGAGGACGCACACCAGCATGTGTTGATCACCAGCTGACCGATGCTGATCTT
GCCTGCCTTGATGCTATATAAGGTTTTGCTATTCATTGAAAGCAGTAGTGACTGATTTGTATATA
>MinSyn_1946|Strength:0.001222589
GCGTGTCGTTTTAGTGAGGGCTTTGTCAAAAGCTAAAAAAGATGATGCTAGGAGGACCGATGCTGATC
TCTCTCTGCCGACAGTGGTCCCAAAGTGAAGCATCTTCCCACGACCACTGGAAAAAGAAGAGGTCGGT
AACCACGTCTACAACTGCACCTCACATCAGAAGATCAAAGGGCTAAGGACCGATGCTGATCTTCTATA
TAAGGTTTTGCTATTCATTGAAAGCAGTAGTGACTGATTTGTATATA
>MinSyn_1531|Strength:0.001222751
GCGTGTCGTTTTAGTGAGGAACCATTATTGCGCAATTAGGTCAGCCACTTGTGTCTTGTGTAAGATTG
ATGAAAAGTCAAAAACAAAAATCAATTATTCACTGCTAGGATGAAGCATCTTCCCTACAAAACTGGTA
CTTGTGTACAGGGCAGTGGTCCCTCCACTGATCTTGCCTGCCTCTCTCTGCCGACAGTGGTCCCAAAC
ACTATGCCCAATTAGGTTCTATATAAGGTTTTGCTATTCATTGAAAGCAGTAGTGACTGATTTGTATA
TA
>MinSyn_1381|Strength:0.001224474
GCGTGTCGTTTTAGTGAGGTCAGAAGATCAAAGGGCTATCTGTGAAGATAAGATAATAATGTTGAAGA
TAAGAACAGGGCTCACTGCTAGGAGGACCGATCTATATAAGGTTTTGCTATTCATTGAAAGCAGTAGT
GACTGATTTGTATATA
>MinSyn_1729|Strength:0.001224865
GCGTGTCGTTTTAGTGAGGAACCACGTCTACAAAGCTTAGCAAGACCTCTACAAAGCAAGTGGATAGG
CTATCAGCTTAGCAAGACAAAAATGTCAAAGATAATTAGGTTGTCTGCACCTCAAGATTGATGAAAAG
TCAAAAACAAAAATCAATTATGGCTATCAGCTTAGGGAAAAAGAAGAGGTCGCGGTAGGTTATGACCC
CCGCCGATGACGCGGGATCACTGCTAGGAGGACCGATGCTGATTCCATCAACAAATAATCCAAGTAAG
GTACAACCATTATTGCGGATCTTGCCTGCCTTGATGATACTATATAAGGTTTTGCTATTCATTGAAAG
CAGTAGTGACTGATTTGTATATA
>MinSyn_1510|Strength:0.00122806
GCGTGTCGTTTTAGTGAGGGCACACCAGCATGTGTTGATCACCAGCTGGTAGGTCACGACCACTATGC
AACCATTATTGCGCGGTAGGTCACGTCTCTCTGCCGACAGTGGTCCCAAACACATGTAGGCTATCAGC
TTAGCCAAATATTTCTTGTTGTGTACAGGGCTCAATTTCGGGAAACCTCCTCGCTGATCCTATATAAG
GTTTTGCTATTCATTGAAAGCAGTAGTGACTGATTTGTATATA
>MinSyn_1796|Strength:0.001229908
GCGTGTCGTTTTAGTGAGGTCCATCAACAAATAATCCAAGTAAGCTCTACAATGGTGGAGCACGACAT
ACAAAACTGGTACTTGTGTACAGGGCTCAAGATTGATGAAAAGTCAAAAACAAAAATCAATTATACAA
AACTGGTACTTCTATATAAGGTTTTGCTATTCATTGAAAGCAGTAGTGACTGATTTGTATATA
>MinSyn_1586|Strength:0.001230952
GCGTGTCGTTTTAGTGAGGAAAAATGTCAAAGATACCACTATGCCCAATGGTGGAGCACGACACACAT
GTAGGCCTATATAAGGTTTTGCTATTCATTGAAAGCAGTAGTGACTGATTTGTATATA
>MinSyn_1776|Strength:0.001231724
GCGTGTCGTTTTAGTGAGGAAAAATGTCAAAGATAGGTAGGTCACGAAATTTCGGGAAACCTCCTCGG
CTAGGAGGACCGATGCTGATCTTGCCTATATAAGGTTTTGCTATTCATTGAAAGCAGTAGTGACTGAT
TTGTATATA
>MinSyn_1029|Strength:0.001232497
GCGTGTCGTTTTAGTGAGGTTATGACCCCCGCCGATGACGCGGGATTCTCTCTGCCGACAGTGGTCCC
AAACGACCACTATGCATCCTTACCGCTATGGGTAAGATTATTAGGTTGTCTGCACCTCACATGAATTT
CGGGAAACCTCCTCGTGGTACTTGTTTTCAACAATAGGTCACGACCACTATCTATATAAGGTTTTGCT
ATTCATTGAAAGCAGTAGTGACTGATTTGTATATA
>MinSyn_1425|Strength:0.001234153
GCGTGTCGTTTTAGTGAGGCAAATATTTCTTGTTTAGGTTGTCTGCACAATTTCGGGAAACCTCCTCG
TCACTGCTAGGAGGACCGATGCTGATTGAAGATAAGATAATAATGTTGAAGATAAGACAGCTTAGCAA
AAATGTCAAAGATATGATCTTGCCTGTTATGACCCCCGCCGATGACGCGGGACTCTACAAAACTGGGA

AAAAGAAGAGGTCTTGTGTACAGGGCTCACTGCTAGGAGGCTATATAAGGTTTTGCTATTCATTGAAA
GCAGTAGTGACTGATTTGTATATA
>MinSyn_1950|Strength:0.001238784
GCGTGTCGTTTTAGTGAGGGGAAAAAGAAGAGGTTGCCTGCCTTTGAAGATAAGATAATAATGTTGAA
GATAAGAGCTCACTGCTAGGAGGCAAATATTTCTTGTTAGCAAGACCTCTACAAAACTGGTAGTGGGA
GCCACCATAGCAAGACTCCATCAACAAATAATCCAAGTAAGTACTTGTGTACAGCTATATAAGGTTTT
GCTATTCATTGAAAGCAGTAGTGACTGATTTGTATATA
>MinSyn_1480|Strength:0.001239296
GCGTGTCGTTTTAGTGAGGCAAATATTTCTTGTGGTCACGACCAGCAAGTGGATCACATGTAGGCTAA
CCACGTCTACAAGCCCAATTAGGTTGTCTGCACCTCAGCACACCAGCATGTGTTGATCACCAGCTGTC
AGAAGATCAAAGGGCTATAGGCTGGTGGAGCACGACAGGTTGTCTGCACCTCACATTGAAGCATCTTC
CAAACTGGTACTTGTGTACAGGGCTCAAATTTCGGGAAACCTCCTCGTCTATATAAGGTTTTGCTATT
CATTGAAAGCAGTAGTGACTGATTTGTATATA
>MinSyn_1443|Strength:0.001240917
GCGTGTCGTTTTAGTGAGGAACCATTATTGCGGCCCAATTAGGTTGTCTCAAATATTTCTTGTACCAC
TATGCCCAATTATTTTCAACAAGGCCAGTGGTCCCTCCACTGATCTTGCCTGCCTTGATGAGGTGGCT
CCTACTGCCTGCCTTAAGATTGATGAAAAGTCAAAAACAAAAATCAATTATGGGCTCACTGCTAGGAG
GACCGATGCGTGGGAGCCACCACACCTCACATGTCTATATAAGGTTTTGCTATTCATTGAAAGCAGTA
GTGACTGATTTGTATATA
>MinSyn_1309|Strength:0.001241396
GCGTGTCGTTTTAGTGAGGGCTTTGTCAAAAGCTAAAAAAGATGATGCAAACTGGTTCACTATCAGCC
ACGACCAGTGGGAGCCACCAGCTTAGCAAGACCTCAGCAAGTGGATCTTGGCACACCAGCATGTGTTG
ATCACCAGCTATGTAGGCTATCAGCTTAGCAAGAAAGATTGATGAAAAGTCAAAAACAAAAATCAATT
ATGGAGCAGCCACTTGTGTTGTGTACAGGGCTCACTCTATATAAGGTTTTGCTATTCATTGAAAGCAG
TAGTGACTGATTTGTATATA
>MinSyn_1856|Strength:0.001245289
GCGTGTCGTTTTAGTGAGGAAAAATGTCAAAGATATCAGCTTAGCAAGACCTCTACAAAACTGTCCAT
CAACAAATAATCCAAGTAAGGGTTGTCTGCACCTCTGAAGCATCTTCCTTCTATATAAGGTTTTGCTA
TTCATTGAAAGCAGTAGTGACTGATTTGTATATA
>MinSyn_1841|Strength:0.001247467
GCGTGTCGTTTTAGTGAGGTCAGAAGATCAAAGGGCTAACTGGTACTTGTGTACAGGATCCTTACCGC
TATGGGTAAGATTTACAGGGCAACCATTATTGCGGGACCGATGCTGATCTTCTATATAAGGTTTTGCT
ATTCATTGAAAGCAGTAGTGACTGATTTGTATATA
>MinSyn_1807|Strength:0.001248041
GCGTGTCGTTTTAGTGAGGAAAAATGTCAAAGATAGCCTGCCTTGATTGCGATAAAGGAAAGGCTATC
AGCTTAGCAAGACCTCTACAAAACTAGGTGGCTCCTACCACATGTAGGCTATTTATGACCCCCGCCGA
TGACGCGGGAACCTATATAAGGTTTTGCTATTCATTGAAAGCAGTAGTGACTGATTTGTATATA
>MinSyn_1268|Strength:0.001250065
GCGTGTCGTTTTAGTGAGGTTTTCAACAACTATCAGCTTAGCAAGACCTCTACAAAACAACCATTATT
GCGGTGAACCACGTCTACAATATGCCCAATTAGGTTGTGCACACCAGCATGTGTTGATCACCAGCTCG
TGGGAGCCACCACTATGCCCAATTAGGTTGTCTGCACCTCTCAGAAGATCAAAGGGCTATCTTCTATA
TAAGGTTTTGCTATTCATTGAAAGCAGTAGTGACTGATTTGTATATA
>MinSyn_1051|Strength:0.001251079
GCGTGTCGTTTTAGTGAGGAGCAAGTGGATACTTGTGTACAGGGCTCAAAGATTGATGAAAAGTCAAA
AACAAAAATCAATTATCAACTATATAAGGTTTTGCTATTCATTGAAAGCAGTAGTGACTGATTTGTAT
ATA
>MinSyn_1164|Strength:0.001253891
GCGTGTCGTTTTAGTGAGGATGAGCTAAGCACATACGTCAGTCAGCTTAGCAAGACCTCTACAAAACT
GGTAACCATTATTGCGACCTCTACAAAACTGTGAAGATAAGATAATAATGTTGAAGATAAGAAAACTG
GTACCAGTGGTCCCTCCACAAACTGGTACTTGTGTATTGCGATAAAGGAAAGGGGTTGTCTGCACCTC
ACTTATGACCCCCGCCGATGACGCGGGAAATGGAAAAAGAAGAGGTGTCAGAAGATCAAAGGGCTAGG
TACTTGTGTACAGTCACTATCAGCAAGACCTCTACAAAACTGGTACTTGCTATATAAGGTTTTGCTAT
TCATTGAAAGCAGTAGTGACTGATTTGTATATA
>MinSyn_1611|Strength:0.001253974

GCGTGTCGTTTTAGTGAGGGCACACCAGCATGTGTTGATCACCAGCTCCTCTACAAAACTGGTACTTG
TGTTCAGAAGATCAAAGGGCTACCTGCCTATCCTTACCGCTATGGGTAAGATTTTGTGTACAGGGCTC
ACTGGGAAAAAGAAGAGGTATCTTGCCTGCCTTGACTATATAAGGTTTTGCTATTCATTGAAAGCAGT
AGTGACTGATTTGTATATA
>MinSyn_1974|Strength:0.001254936
GCGTGTCGTTTTAGTGAGGTGAAGATAAGATAATAATGTTGAAGATAAGACGCGGTAGGTCACGACCA
CTATATCGAAAGGACAGTAACCTCTACAAAACTGGTACTTGTGTGGAAAAAGAAGAGGTCTGGTACTT
CAGAAGATCAAAGGGCTATTATCTCTCTGCCGACAGTGGTCCCAAACCAATTAGAACCACGTCTACAA
TGTACAGGGCTCACTGCTGCACACCAGCATGTGTTGATCACCAGCTTCAGCTTAGTCACTATCAGCAC
CACTATGCCCAATTAGGTTGTCTGCACCCTATATAAGGTTTTGCTATTCATTGAAAGCAGTAGTGACT
GATTTGTATATA
>MinSyn_1171|Strength:0.001258361
GCGTGTCGTTTTAGTGAGGAATTTCGGGAAACCTCCTCGCCAGCCACTTGTGTTTAGGTTGTCTGCAC
CTCACATGAAAAATGTCAAAGATAGGTCACGACCACTATGCCTCCATCAACAAATAATCCAAGTAAGG
TACAGGGCTCACCTATATAAGGTTTTGCTATTCATTGAAAGCAGTAGTGACTGATTTGTATATA
>MinSyn_1431|Strength:0.00125863
GCGTGTCGTTTTAGTGAGGCAGCCACTTGTGTGCAAGACAACCATTATTGCGTGTCTGCACCTCACAT
GTTCTCTCTGCCGACAGTGGTCCCAAACGCGGTAGGGTGGGAGCCACCAAATTAGGTTGTCTGCACCT
CACATGAAGCATCTTCCTATCGAAAGGACAGTATAGGTCACGACCACTATGCAACCACGTCTACAATA
GCAAGACTATATAAGGTTTTGCTATTCATTGAAAGCAGTAGTGACTGATTTGTATATA
>MinSyn_1226|Strength:0.00125968
GCGTGTCGTTTTAGTGAGGTCACTATCAGCCTATGAACCATTATTGCGTTAGGTGAAGATAAGATAAT
AATGTTGAAGATAAGAATGCTGATCTTGCCTGCCTTGAGCACACCAGCATGTGTTGATCACCAGCTTG
GTACTTATCGAAAGGACAGTAAGGACCGATGCTGATCTTGCGGAAAAAGAAGAGGTCAGCTTAGCAAG
ACCTCTACAAAAAAGATTGATGAAAAGTCAAAAACAAAAATCAATTATCGCGGTAGGTCACGACCCTA
TATAAGGTTTTGCTATTCATTGAAAGCAGTAGTGACTGATTTGTATATA
>MinSyn_1989|Strength:0.001261901
GCGTGTCGTTTTAGTGAGGGGAAAAAGAAGAGGTTTGATGATAATCGAAAGGACAGTAGTACTTGTGT
ACAGGGCTCACTGCTAGAACCACGTCTACAATGATCCTTACCGCTATGGGTAAGATTACAAAACTGGT
ACTATATAAGGTTTTGCTATTCATTGAAAGCAGTAGTGACTGATTTGTATATA
>MinSyn_1641|Strength:0.001262996
GCGTGTCGTTTTAGTGAGGATGACGTAAGCCATGACGTCTAATTAGGTTGTCTGCACCTCACTTTTCA
ACAAGTGTACAGGGCTCACTGCTAGGAGAAGATTGATGAAAAGTCAAAAACAAAAATCAATTATTACT
TGTGTACAGGGCTCACTGCTAGCACACCAGCATGTGTTGATCACCAGCTGCCTGCCTTCCATCAACAA
ATAATCCAAGTAAGCCTCTACAAAACTGGTACTTGAAGATAAGATAATAATGTTGAAGATAAGAGGTA
GGTCACGACCACTATCGAAAGGACAGTATGTGTACAGGGCTCACTGCGGAAAAAGAAGAGGTACTATG
ATTGCGATAAAGGAAAGGCTACAAAACTGGTACTTGCTATATAAGGTTTTGCTATTCATTGAAAGCAG
TAGTGACTGATTTGTATATA
>MinSyn_1571|Strength:0.001263076
GCGTGTCGTTTTAGTGAGGGCTTTGTCAAAAGCTAAAAAAGATGATGCCCCAGCACACCAGCATGTGT
TGATCACCAGCTTAGGTCACGACCACCAGCCACTTGTGTATCAGTCCATCAACAAATAATCCAAGTAA
GCCTCATCACTATCAGCGGACCGATGCTGATCTTGCCTATATAAGGTTTTGCTATTCATTGAAAGCAG
TAGTGACTGATTTGTATATA
>MinSyn_1365|Strength:0.001266062
GCGTGTCGTTTTAGTGAGGTGGTGGAGCACGACATAGTTATGACCCCCGCCGATGACGCGGGAGAGGA
CCGATGCTGATCTTGCCATCGAAAGGACAGTATCTACATCCATCAACAAATAATCCAAGTAAGCGCGG
TAGGTCACGACCACTATGCCCCTATATAAGGTTTTGCTATTCATTGAAAGCAGTAGTGACTGATTTGT
ATATA
>MinSyn_1657|Strength:0.001269417
GCGTGTCGTTTTAGTGAGGGTGGGAGCCACCACTGATCTTGCCTGCCTTGATGATCAGAAGATCAAAG
GGCTAATCAGCTTAGCAAGACGGAAAAAGAAGAGGTCCACTATGCCCAATTAGGTTGTCTGCAGCAAG
TGGATTGGTACTTGTGTAAAGATTGATGAAAAGTCAAAAACAAAAATCAATTATCTATCAGCTTAGCT
CTCTCTGCCGACAGTGGTCCCAAATATTGCGATAAAGGAAAGGCCCAATTAGGTTGTCTGCACACCAG
CATGTGTTGATCACCAGCTTCACTGCTAGGAGGACCGTGAAGCATCTTCCTGCTGATCTTGCCTGCCC

TATATAAGGTTTTGCTATTCATTGAAAGCAGTAGTGACTGATTTGTATATA
>MinSyn_1541|Strength:0.001269606
GCGTGTCGTTTTAGTGAGGAAAAATGTCAAAGATAGGGCTCTGAAGATAAGATAATAATGTTGAAGAT
AAGACTCACTCCATCAACAAATAATCCAAGTAAGGTACAGGGCTCACTGAAGATTGATGAAAAGTCAA
AAACAAAAATCAATTATCAATTAAGCAAGTGGATAGCTTAGCAAGACCTCTACAAACTATATAAGGTT
TTGCTATTCATTGAAAGCAGTAGTGACTGATTTGTATATA
>MinSyn_1337|Strength:0.001270212
GCGTGTCGTTTTAGTGAGGCAAATATTTCTTGTACTATGCCCAATCAGAAGATCAAAGGGCTACACCT
CACATCCTTACCGCTATGGGTAAGATTTTGTCTGCACCTCACAGGTGGCTCCTACTGGTAAACCATTA
TTGCGTCTACAAAACTGGTACTTGTGTACTGAAGATAAGATAATAATGTTGAAGATAAGAGGGCTCAC
TGCTTTTTCAACAAATCTTGCCTAATTTCGGGAAACCTCCTCGCAGCTTAGCAAGACCTCCTATATAA
GGTTTTGCTATTCATTGAAAGCAGTAGTGACTGATTTGTATATA
>MinSyn_1837|Strength:0.001271311
GCGTGTCGTTTTAGTGAGGATTGCGATAAAGGAAAGGCCAATTAGGTGGGAGCCACCAAGGAGGACCG
ATCTATATAAGGTTTTGCTATTCATTGAAAGCAGTAGTGACTGATTTGTATATA
>MinSyn_1891|Strength:0.001272347
GCGTGTCGTTTTAGTGAGGAACCACGTCTACAACACCTCACATGTAGGCTATCAGCTGTGGGAGCCAC
CACACCTCACATGTAGGCTATCAGCTTTCTCTCTGCCGACAGTGGTCCCAAATCTTGCCTAAAAATGT
CAAAGATAAACTGGTACTTGTGTACAGGGCTGCTTTGTCAAAAGCTAAAAAAGATGATGCTAGCACAC
CAGCATGTGTTGATCACCAGCTCTGCACCTCACATGTAATCCTTACCGCTATGGGTAAGATTAGCTTA
GCAAGACTGAAGATAAGATAATAATGTTGAAGATAAGATACTTGTCTATATAAGGTTTTGCTATTCAT
TGAAAGCAGTAGTGACTGATTTGTATATA
>MinSyn_1863|Strength:0.001273701
GCGTGTCGTTTTAGTGAGGTGGTGGAGCACGACAGGCTATCAGCTTAGCAAGACAAGATTGATGAAAA
GTCAAAAACAAAAATCAATTATACATGTAGTCTCTCTGCCGACAGTGGTCCCAAAAATTAGGTTGTCT
GCACCTCACATGTCCATCAACAAATAATCCAAGTAAGGATCTTGCCTGCCTATATAAGGTTTTGCTAT
TCATTGAAAGCAGTAGTGACTGATTTGTATATA
>MinSyn_1335|Strength:0.001275725
GCGTGTCGTTTTAGTGAGGTCACTATCAGCTCTGCACCTCAAACCACGTCTACAACTATCCATCAACA
AATAATCCAAGTAAGCACTGCTAGGAGGACCGATGCTGATCTTCTATATAAGGTTTTGCTATTCATTG
AAAGCAGTAGTGACTGATTTGTATATA
>MinSyn_1008|Strength:0.001277468
GCGTGTCGTTTTAGTGAGGATCGAAAGGACAGTAAGACCTCTACAAAACTGGTACTTGGAAAAAGAAG
AGGTCTTAGCAAGACCTCTACAAAACTGGTACTCAGAAGATCAAAGGGCTATGCCCAATTAGGTTGTC
TGCATTATGACCCCCGCCGATGACGCGGGAAGTGGGAGCCACCAACAAAACTGAACCATTATTGCGAA
CTGGTACTTGTGTACAGGGCTCCATCAACAAATAATCCAAGTAAGCGGTAGCTATATAAGGTTTTGCT
ATTCATTGAAAGCAGTAGTGACTGATTTGTATATA
>MinSyn_1892|Strength:0.001279776
GCGTGTCGTTTTAGTGAGGATCGAAAGGACAGTATAGGTTGTCTGAGGTGGCTCCTACTGGTACTTGT
GTGCACACCAGCATGTGTTGATCACCAGCTTGGTACTTGTGTAAGATTGATGAAAAGTCAAAAACAAA
AATCAATTATTCGCGGTAGGTCACGACCACTATGCCCCTATATAAGGTTTTGCTATTCATTGAAAGCA
GTAGTGACTGATTTGTATATA
>MinSyn_1556|Strength:0.00128026
GCGTGTCGTTTTAGTGAGGGTGGGAGCCACCAGGTACTAACCATTATTGCGAAGACAGCAAGTGGATC
ACTATTGCGATAAAGGAAAGGAATCCTTACCGCTATGGGTAAGATTACTATGCCCAATTAGGTTGTCT
GCACCTATATAAGGTTTTGCTATTCATTGAAAGCAGTAGTGACTGATTTGTATATA
>MinSyn_1372|Strength:0.001280806
GCGTGTCGTTTTAGTGAGGGTGGGAGCCACCAATCAGCTTAGCATCACTATCAGCGCTGATCTAAGAT
TGATGAAAAGTCAAAAACAAAAATCAATTATTATTTTCAACAAAGGCTATCAGCTTAGCAGGTGGCTC
CTACACTATGCCCAATTAGGTTCAGTGGTCCCTCCACCATGTAGGCTATCAATCCTTACCGCTATGGG
TAAGATTTGCCCAATTAGGTTGTCTATATAAGGTTTTGCTATTCATTGAAAGCAGTAGTGACTGATTT
GTATATA
>MinSyn_1310|Strength:0.001281282
GCGTGTCGTTTTAGTGAGGAACCACGTCTACAACTTCTCTCTGCCGACAGTGGTCCCAAACTGGTACT

TGTGTACAGGGCTGTGGGAGCCACCAATGAAAAATGTCAAAGATATCTGCACCTCCTATATAAGGTTT
TGCTATTCATTGAAAGCAGTAGTGACTGATTTGTATATA
>MinSyn_1397|Strength:0.001285156
GCGTGTCGTTTTAGTGAGGATTGCGATAAAGGAAAGGCTCTACAAAACTGGTACTTGTGCAGTGGTCC
CTCCACGTCTATATAAGGTTTTGCTATTCATTGAAAGCAGTAGTGACTGATTTGTATATA
>MinSyn_1543|Strength:0.001285156
GCGTGTCGTTTTAGTGAGGGGAAAAAGAAGAGGTGCACCTCACATTGCGATAAAGGAAAGGTTGTCTG
CACCTCACCTATATAAGGTTTTGCTATTCATTGAAAGCAGTAGTGACTGATTTGTATATA
>MinSyn_1592|Strength:0.001285786
GCGTGTCGTTTTAGTGAGGTGAAGCATCTTCCGCTAGGAGGACTTATGACCCCCGCCGATGACGCGGG
AATGCTGATCTTGCCTGCCTTGATGATATTGCGATAAAGGAAAGGTTATCACTATCAGCGGCTCACTG
CTAGGAGGACCGAACCATTATTGCGTGCCTATATAAGGTTTTGCTATTCATTGAAAGCAGTAGTGACT
GATTTGTATATA
>MinSyn_1424|Strength:0.001285875
GCGTGTCGTTTTAGTGAGGGTGGGAGCCACCACTATGCCCAATTAGGTTGTCTGCACCTCAGCAAGTG
GATAATCCTTACCGCTATGGGTAAGATTAGGAGGACCGATGCTGATCTTGCAGCCACTTGTGTACAAA
ACTGGTACTTGGGAAAAAGAAGAGGTTTGTGTACAGGGCTCTCCATCAACAAATAATCCAAGTAAGAG
GAGGAATCGAAAGGACAGTAAGGCTATCAGCTTAAAGATTGATGAAAAGTCAAAAACAAAAATCAATT
ATCCTGCCTAAAAATGTCAAAGATATAGGAGGACCGATGCTGATCTTGCCTGCCCTATATAAGGTTTT
GCTATTCATTGAAAGCAGTAGTGACTGATTTGTATATA
>MinSyn_1991|Strength:0.001287024
GCGTGTCGTTTTAGTGAGGATCGAAAGGACAGTAACAAAACTGGTACTTGTGTACAGGCAGTGGTCCC
TCCACCTGCATCCTTACCGCTATGGGTAAGATTACTGCTAGGAGGACCTATATAAGGTTTTGCTATTC
ATTGAAAGCAGTAGTGACTGATTTGTATATA
>MinSyn_1513|Strength:0.001287468
GCGTGTCGTTTTAGTGAGGGTGGGAGCCACCATTGACAAATATTTCTTGTAGGCTATCAGCTTAGCAA
GACCTCTTCAGAAGATCAAAGGGCTAACCACTATGCCCAAAACCATTATTGCGTCAGCTTAGCAAGAC
CTAGGTGGCTCCTACGCTATATAAGGTTTTGCTATTCATTGAAAGCAGTAGTGACTGATTTGTATATA
>MinSyn_1324|Strength:0.001290833
GCGTGTCGTTTTAGTGAGGTCAGAAGATCAAAGGGCTAACAGGGCTAGGTGGCTCCTACATTAGGTTG
TCTGCACCTCTGAAGCATCTTCCTAGGAGGACCGATGAACCATTATTGCGTGCTGATCTTGCCTGCCA
AAAATGTCAAAGATAAGTCTCTCTGCCGACAGTGGTCCCAAATAGGTTGTCTGCACCTCCATCAACAA
ATAATCCAAGTAAGCACTATGCCCAATTAGGTTGTCTGCTGGTGGAGCACGACAAGGCTCTATATAAG
GTTTTGCTATTCATTGAAAGCAGTAGTGACTGATTTGTATATA
>MinSyn_1622|Strength:0.0012925
GCGTGTCGTTTTAGTGAGGATTGCGATAAAGGAAAGGGCAAGTGGGAGCCACCATATCAGCTTAGCAA
CTATATAAGGTTTTGCTATTCATTGAAAGCAGTAGTGACTGATTTGTATATA
>MinSyn_1032|Strength:0.001293426
GCGTGTCGTTTTAGTGAGGAATTTCGGGAAACCTCCTCGCTCTACAAAACTGGTACTTGTGTACTGAA
GCATCTTCCTCTACAAAATTGCGATAAAGGAAAGGCTGCTAGGAGGACCGATTTATGACCCCCGCCGA
TGACGCGGGACACTGCTAGGAGGACCGATGAAGATAAGATAATAATGTTGAAGATAAGAGCCCAATCC
ATCAACAAATAATCCAAGTAAGAGGAGGACCGATGCTGATCTTGCACACCAGCATGTGTTGATCACCA
GCTTAGCAAGACCTCTACAAAACTGCTATATAAGGTTTTGCTATTCATTGAAAGCAGTAGTGACTGAT
TTGTATATA
>MinSyn_1644|Strength:0.001294564
GCGTGTCGTTTTAGTGAGGTTTTCAACAATGTACAGGGCTCACTGCTATCCATCAACAAATAATCCAA
GTAAGGTCTGCACCTCACATGTAAAAATGTCAAAGATAAGACAACCATTATTGCGAGGCTATCAGCTT
AGCAAGACCTCAGCCACTTGTGTACCTCTACAAAACTGGTAGGTGGCTCCTACCACGACCACTATGCC
CAATTAAAGATTGATGAAAAGTCAAAAACAAAAATCAATTATCCTCTACAAAACTGGTACTTGTGTAC
ATGAAGATAAGATAATAATGTTGAAGATAAGATAGGATCCTTACCGCTATGGGTAAGATTCACCTCAC
ATGTAGGCTATCAGCTCTATATAAGGTTTTGCTATTCATTGAAAGCAGTAGTGACTGATTTGTATATA
>MinSyn_1546|Strength:0.001296145
GCGTGTCGTTTTAGTGAGGTTTTCAACAAATGTAGGCTATCAGCTTAGCAAGACCTCTCTCTGCCGAC
AGTGGTCCCAAAACCACTAAAGATTGATGAAAAGTCAAAAACAAAAATCAATTATGCTGATCTTGCCT

GCCTTGACTATATAAGGTTTTGCTATTCATTGAAAGCAGTAGTGACTGATTTGTATATA
>MinSyn_1234|Strength:0.001296597
GCGTGTCGTTTTAGTGAGGAGGTGGCTCCTACACATGGAAAAAGAAGAGGTGCTATCAGCTTAGCAAG
TGGGAGCCACCAAACTGGTATGAAGATAAGATAATAATGTTGAAGATAAGATCACGAAGCAAGTGGAT
TAAAAATGTCAAAGATATAGGAGGACCGATGCTGATCTTCTATATAAGGTTTTGCTATTCATTGAAAG
CAGTAGTGACTGATTTGTATATA
>MinSyn_1217|Strength:0.0012971
GCGTGTCGTTTTAGTGAGGAAAAATGTCAAAGATACTAGGAGGACCGATGCTGAAGATTGATGAAAAG
TCAAAAACAAAAATCAATTATTAGGTCACGACCACCTATATAAGGTTTTGCTATTCATTGAAAGCAGT
AGTGACTGATTTGTATATA
>MinSyn_1525|Strength:0.001299025
GCGTGTCGTTTTAGTGAGGTCAGAAGATCAAAGGGCTAGGACCGATGCTGATCGGAAAAAGAAGAGGT
GTTATGACCCCCGCCGATGACGCGGGACTCTACAAAACTCCATCAACAAATAATCCAAGTAAGTACAG
GGCTCACTGCTAGGAAAGATTGATGAAAAGTCAAAAACAAAAATCAATTATGGACCGATGCTGATCTT
GCCTGCCTTGATGTGAAGATAAGATAATAATGTTGAAGATAAGAACTATGCCCAATTAGGTCTATATA
AGGTTTTGCTATTCATTGAAAGCAGTAGTGACTGATTTGTATATA
>MinSyn_1907|Strength:0.001299065
GCGTGTCGTTTTAGTGAGGCAAATATTTCTTGTGAGGACCGATGCTGATCTTGTCCATCAACAAATAA
TCCAAGTAAGGATGCTGATCTTGCCTGCCTTGATGAACCATTATTGCGATGAAGATTGATGAAAAGTC
AAAAACAAAAATCAATTATCCTCTACAAAACTGGTACTTTATGACCCCCGCCGATGACGCGGGATATG
CCCAATTAGGTTGTCTGCACCTCACAGTGGTCCCTCCACCAGGGCTCACTGCTAGGAGGACCGATGCT
GAAGATAAGATAATAATGTTGAAGATAAGATCACTGCTATCCTTACCGCTATGGGTAAGATTACCTCA
CATGTAGGCTATCAGCTTAGCATTGCGATAAAGGAAAGGTACTATATAAGGTTTTGCTATTCATTGAA
AGCAGTAGTGACTGATTTGTATATA
>MinSyn_1120|Strength:0.001306601
GCGTGTCGTTTTAGTGAGGTTATGACCCCCGCCGATGACGCGGGAGTTGTCTGCACCTCACATGTAGG
CTATCAGTCTCTCTGCCGACAGTGGTCCCAAATCGCGGTAGGTCATCCATCAACAAATAATCCAAGTA
AGGTACAGGGCTCACTGCTAACCATTATTGCGTGCTGATCTATCCTTACCGCTATGGGTAAGATTTTG
CCTGCCTTGATGAAAGATTGATGAAAAGTCAAAAACAAAAATCAATTATATCTTGCCTGCCTTGAGCA
CACCAGCATGTGTTGATCACCAGCTGACCACTATGCCCAATTAGGTTGTCCTATATAAGGTTTTGCTA
TTCATTGAAAGCAGTAGTGACTGATTTGTATATA
>MinSyn_1981|Strength:0.00130703
GCGTGTCGTTTTAGTGAGGTCTCTCTGCCGACAGTGGTCCCAAATAAGATTGATGAAAAGTCAAAAAC
AAAAATCAATTATAACTGGTACTTGTGTACAGGGCTCTGAAGATAAGATAATAATGTTGAAGATAAGA
GTGTATTATGACCCCCGCCGATGACGCGGGAGACCTCTACAAAACTGGTACTTGTGTACAAAAATGTC
AAAGATAATGTAGGCTATCAGTGGGAGCCACCAGTAGGTCACGACTATATAAGGTTTTGCTATTCATT
GAAAGCAGTAGTGACTGATTTGTATATA
>MinSyn_1345|Strength:0.001307826
GCGTGTCGTTTTAGTGAGGGGAAAAAGAAGAGGTTGTGTACAGGATCGAAAGGACAGTACCAACTATA
TAAGGTTTTGCTATTCATTGAAAGCAGTAGTGACTGATTTGTATATA
>MinSyn_1519|Strength:0.001308464
GCGTGTCGTTTTAGTGAGGGGAAAAAGAAGAGGTGTGTACAGGGCTCACTGCTAGGAGGTTTTCAACA
ATTGTGTACAGTCCATCAACAAATAATCCAAGTAAGAGACCCAAATATTTCTTGTCTTGCCTGCTATA
TAAGGTTTTGCTATTCATTGAAAGCAGTAGTGACTGATTTGTATATA
>MinSyn_1895|Strength:0.001308707
GCGTGTCGTTTTAGTGAGGTTTTCAACAAGGCTCACTGCTAAATTTCGGGAAACCTCCTCGATTCCAT
CAACAAATAATCCAAGTAAGCTGGTACTTGTGTACAGGCACACCAGCATGTGTTGATCACCAGCTATG
CTGATCTTGCCTGCCTTGATGAAGCATCTTCCGCCTGCCTTGATCTATATAAGGTTTTGCTATTCATT
GAAAGCAGTAGTGACTGATTTGTATATA
>MinSyn_1910|Strength:0.001309498
GCGTGTCGTTTTAGTGAGGAAGATTGATGAAAAGTCAAAAACAAAAATCAATTATGCTGATCTTGCCT
GCCTTGATGATAAATTTCGGGAAACCTCCTCGGCTGATCTTGCCTGCCTTGATGTGAAGATAAGATAA
TAATGTTGAAGATAAGATTGCAGTGGTCCCTCCACATTAGGTTGTCTGCACCTCACATGTCTATATAA
GGTTTTGCTATTCATTGAAAGCAGTAGTGACTGATTTGTATATA

>MinSyn_1481|Strength:0.001314328
GCGTGTCGTTTTAGTGAGGAAAAATGTCAAAGATAGGAGGACCGATGCTGATCTTGCCTAACCACGTC
TACAAAGGAGGACCGATGCTGATCTTGCCTTTTTCAACAAGTACAGTTATGACCCCCGCCGATGACGC
GGGATGTAGGCTATCAGCTTAGCATGAAGCATCTTCCAAAACTGGTATGGTGGAGCACGACAGATGCT
GATCTTGCCTGCCTTGATGAACCATTATTGCGCAAGACCTCTACAAAACTGGTACTTCTCTCTGCCGA
CAGTGGTCCCAAAGTTGAAGATAAGATAATAATGTTGAAGATAAGACCTCACATCTATATAAGGTTTT
GCTATTCATTGAAAGCAGTAGTGACTGATTTGTATATA
>MinSyn_1623|Strength:0.001314331
GCGTGTCGTTTTAGTGAGGGCACACCAGCATGTGTTGATCACCAGCTCGCGGTAGGTCACGATCCTTA
CCGCTATGGGTAAGATTGGTCACGGTGGGAGCCACCAACTGCTAGGAGGACCAAAAATGTCAAAGATA
TATCAGCTCCATCAACAAATAATCCAAGTAAGAGGTCTGAAGCATCTTCCATTAGGTTGTCTGCACCT
CACATGAACCACGTCTACAATACAGGGCTCAAATATTTCTTGTCCAATTAGCTATATAAGGTTTTGCT
ATTCATTGAAAGCAGTAGTGACTGATTTGTATATA
>MinSyn_1572|Strength:0.001314334
GCGTGTCGTTTTAGTGAGGTCAGAAGATCAAAGGGCTAATGTAGGCTATCAGCTTAGCAAGACCATTG
CGATAAAGGAAAGGATGCCCAATTAGGTTGTCTGCACCTCACATCTCTCTGCCGACAGTGGTCCCAAA
AGGACCGATGCTGATCCATCAACAAATAATCCAAGTAAGCTATCCTATATAAGGTTTTGCTATTCATT
GAAAGCAGTAGTGACTGATTTGTATATA
>MinSyn_1585|Strength:0.001316878
GCGTGTCGTTTTAGTGAGGAACCATTATTGCGGACCGATGCTGATCTTCACTATCAGCTGCACCTCAC
ATGTAGCAAATATTTCTTGTCTAGGCAGCCACTTGTGTCCTCTATATAAGGTTTTGCTATTCATTGAA
AGCAGTAGTGACTGATTTGTATATA
>MinSyn_1680|Strength:0.001317221
GCGTGTCGTTTTAGTGAGGATCGAAAGGACAGTAGTCTGCACCTCACATGTAGGTCTCTCTGCCGACA
GTGGTCCCAAATCAGCTTAGCAAGACCTCTACAAAACTAGGTGGCTCCTACTGCTGATCTTGCCTGCC
TTGATAACCACGTCTACAATTAGCAAGACCTCTACAGGAAAAAGAAGAGGTCTATGCCCAATTAGGTT
GTCTGCACCTCACTGAAGATAAGATAATAATGTTGAAGATAAGACTTGCAAATATTTCTTGTGGGCTC
ACTGCATTGCGATAAAGGAAAGGACAGGGCTCACTGCACACCAGCATGTGTTGATCACCAGCTATCAG
CTTCTATATAAGGTTTTGCTATTCATTGAAAGCAGTAGTGACTGATTTGTATATA
>MinSyn_1743|Strength:0.001318078
GCGTGTCGTTTTAGTGAGGTCAGAAGATCAAAGGGCTACTATCAGCTTAGCAAGACCTCTACAAAACC
ACGTCTACAACACTATGCCCAATTAGGTTGTCTGCACCTCCATCAACAAATAATCCAAGTAAGTGTGT
ACAGGTGAAGCATCTTCCTGCCTTGATGCAGCCACTTGTGTTGCTATATAAGGTTTTGCTATTCATTG
AAAGCAGTAGTGACTGATTTGTATATA
>MinSyn_1570|Strength:0.001318705
GCGTGTCGTTTTAGTGAGGGTGGGAGCCACCATGTCTGCACCTCACATATCCTTACCGCTATGGGTAA
GATTATGCCCAATTAGGTTCTATATAAGGTTTTGCTATTCATTGAAAGCAGTAGTGACTGATTTGTAT
ATA
>MinSyn_1201|Strength:0.001320226
GCGTGTCGTTTTAGTGAGGAACCACGTCTACAAGCCTGCCTTGATGATAATCGAAAGGACAGTACTCT
ACAAAATGAAGATAAGATAATAATGTTGAAGATAAGAAGACCTCTTGAAGCATCTTCCTCACTGCTAA
ATTTCGGGAAACCTCCTCGGCCCAATTAGGTTGTCTGCTATATAAGGTTTTGCTATTCATTGAAAGCA
GTAGTGACTGATTTGTATATA
>MinSyn_1102|Strength:0.001325641
GCGTGTCGTTTTAGTGAGGCAGCCACTTGTGTAATTGCGATAAAGGAAAGGTGTCTGCACCTCACCTA
TATAAGGTTTTGCTATTCATTGAAAGCAGTAGTGACTGATTTGTATATA
>MinSyn_1995|Strength:0.001325765
GCGTGTCGTTTTAGTGAGGCAGCCACTTGTGTCACTGCTAGGAGGTGGCTCCTACAATCACTATCAGC
ACTCTCTCTGCCGACAGTGGTCCCAAAGCTAGGAAGCAAGTGGATCACTGCCTATATAAGGTTTTGCT
ATTCATTGAAAGCAGTAGTGACTGATTTGTATATA
>MinSyn_1349|Strength:0.001326771
GCGTGTCGTTTTAGTGAGGTGGTGGAGCACGACACGATGCTTTTCAACAAGCTATCAGGGAAAAAGAA
GAGGTTACTTGTGTACACAAATATTTCTTGTGATGCTTCTCTCTGCCGACAGTGGTCCCAAAGTTGTC
TGCACCTCACATGTGGGAGCCACCATCTACAAAACTGGTACTTGTGTACAGGGCAAAAATGTCAAAGA

TACTGCCTATCCTTACCGCTATGGGTAAGATTCCTATATAAGGTTTTGCTATTCATTGAAAGCAGTAG
TGACTGATTTGTATATA
>MinSyn_1736|Strength:0.001337891
GCGTGTCGTTTTAGTGAGGCAAATATTTCTTGTAGCAAGATTGATGAAAAGTCAAAAACAAAAATCAA
TTATCAGGGCTCACTGCTAGGAGGACCGATTTTCAACAAACCGATGCTGATCTTGCCTGCCTCTATAT
AAGGTTTTGCTATTCATTGAAAGCAGTAGTGACTGATTTGTATATA
>MinSyn_1882|Strength:0.001338194
GCGTGTCGTTTTAGTGAGGTCTCTCTGCCGACAGTGGTCCCAAAGGTTGTCTGCACCTAACCACGTCT
ACAATCTGCACCTCACATGTAGGCCTATATAAGGTTTTGCTATTCATTGAAAGCAGTAGTGACTGATT
TGTATATA
>MinSyn_1850|Strength:0.001339636
GCGTGTCGTTTTAGTGAGGTGAAGCATCTTCCATCTTGCCTGCCTTGATATCCTTACCGCTATGGGTA
AGATTCTTGCCTGCCTTGATGATGAAGATAAGATAATAATGTTGAAGATAAGACATGTAGGCTATCAG
CTTTGGTGGAGCACGACAGACCTCTACAAAACTTTTCAACAAGGTCACGACCATCTCTCTGCCGACAG
TGGTCCCAAACTGCTATATAAGGTTTTGCTATTCATTGAAAGCAGTAGTGACTGATTTGTATATA
>MinSyn_1527|Strength:0.001340654
GCGTGTCGTTTTAGTGAGGGGAAAAAGAAGAGGTTACTTGTGTAGGTGGCTCCTACGTCTCTCTGCCG
ACAGTGGTCCCAAAAGGGCTCACTGCTAGGAGGTGGGAGCCACCACTTAGCAAGACCTCTACAAAACT
GGTAAGATTGATGAAAAGTCAAAAACAAAAATCAATTATTGCACCTCACATGTAGGAAAAATGTCAAA
GATAGGTACTTGTGTACTCCATCAACAAATAATCCAAGTAAGGTTATGACCCCCGCCGATGACGCGGG
AAGGGCTCACTGCTAGGAGGACCGATGCTCAGTGGTCCCTCCACCACTGCTAGGAGCTATATAAGGTT
TTGCTATTCATTGAAAGCAGTAGTGACTGATTTGTATATA
>MinSyn_1259|Strength:0.001349365
GCGTGTCGTTTTAGTGAGGTCACTATCAGCCAAAACTGGTACTATTGCGATAAAGGAAAGGGGTAGGT
CACGACCACTATGCCCAAGCACACCAGCATGTGTTGATCACCAGCTCATCGAAAGGACAGTAAGCAAG
ACCTCTCTATATAAGGTTTTGCTATTCATTGAAAGCAGTAGTGACTGATTTGTATATA
>MinSyn_1672|Strength:0.00135362
GCGTGTCGTTTTAGTGAGGAAAAATGTCAAAGATATATCAGCTTAGCAAGACCTCTACAAAATGAAGA
TAAGATAATAATGTTGAAGATAAGAAGCTTAGTCAGAAGATCAAAGGGCTAGGGCTCACTGCTAGGAG
GACCGATCTATATAAGGTTTTGCTATTCATTGAAAGCAGTAGTGACTGATTTGTATATA
>MinSyn_1539|Strength:0.001356504
GCGTGTCGTTTTAGTGAGGGCTTTGTCAAAAGCTAAAAAAGATGATGCCACCTCACATGTAGGCTATG
TGGGAGCCACCAACTGCTATCGAAAGGACAGTATGCACCTGGAAAAAGAAGAGGTTGGTACTTGTGTA
CATGAAGATAAGATAATAATGTTGAAGATAAGACTTGCCTGTCTCTCTGCCGACAGTGGTCCCAAATA
CATCCTTACCGCTATGGGTAAGATTACTTGTGTACAGGGCTCACTGCTAGTGAAGCATCTTCCTTGAT
GATACTATATAAGGTTTTGCTATTCATTGAAAGCAGTAGTGACTGATTTGTATATA
>MinSyn_1318|Strength:0.001359504
GCGTGTCGTTTTAGTGAGGAACCATTATTGCGGTACAGGGAATTTCGGGAAACCTCCTCGGCTGATCT
TCTATATAAGGTTTTGCTATTCATTGAAAGCAGTAGTGACTGATTTGTATATA
>MinSyn_1009|Strength:0.001360771
GCGTGTCGTTTTAGTGAGGTCTCTCTGCCGACAGTGGTCCCAAACACCTCACATGTATCGAAAGGACA
GTATGCCCAATTAGGTATTGCGATAAAGGAAAGGATTAGGTTGTCAAAAATGTCAAAGATAATTCTAT
ATAAGGTTTTGCTATTCATTGAAAGCAGTAGTGACTGATTTGTATATA
>MinSyn_1399|Strength:0.001363436
GCGTGTCGTTTTAGTGAGGTTTTCAACAAGTCACGACCCAAATATTTCTTGTCCCAATTAGAAGATTG
ATGAAAAGTCAAAAACAAAAATCAATTATCTGCCTTGATGAAGATAAGATAATAATGTTGAAGATAAG
AATCAGCTTAGCAAGACCTCTAAACCACGTCTACAACTGCACCTCACACAGCCACTTGTGTCAGGGCT
CACTGCTAGGAGGACCGTTATGACCCCCGCCGATGACGCGGGAATGCTATATAAGGTTTTGCTATTCA
TTGAAAGCAGTAGTGACTGATTTGTATATA
>MinSyn_1384|Strength:0.001363626
GCGTGTCGTTTTAGTGAGGATCCTTACCGCTATGGGTAAGATTTGCACCTCACATGTAGGCTATCAAA
AAATGTCAAAGATAGTCTGCACCTCACACAAATATTTCTTGTACTATGCCCAATTCAGCCACTTGTGT
TGCCTTATGACCCCCGCCGATGACGCGGGAACAAAACTGGTACTTGTGTAAATTTCGGGAAACCTCCT
CGATCTTGCCTGCCTATTGCGATAAAGGAAAGGACTATATAAGGTTTTGCTATTCATTGAAAGCAGTA

GTGACTGATTTGTATATA
>MinSyn_1881|Strength:0.001365307
GCGTGTCGTTTTAGTGAGGATCGAAAGGACAGTATCTGCACCTCACATGTAGGCTATCAGTGGTGGAG
CACGACATTGTCTGCACCTCACATCAGCCACTTGTGTAATTAGGTTGTTGAAGCATCTTCCTATCAGC
TTAGCAAGACCTCTACAACCATTATTGCGGCCTGCCTTGATGATAAATTTCGGGAAACCTCCTCGTAG
CAAGACAAGATTGATGAAAAGTCAAAAACAAAAATCAATTATTCACTATATAAGGTTTTGCTATTCAT
TGAAAGCAGTAGTGACTGATTTGTATATA
>MinSyn_1239|Strength:0.001368524
GCGTGTCGTTTTAGTGAGGGTGGGAGCCACCATCTGCACCTCACATGTAGGCTATCATCGAAAGGACA
GTAGTTGTCTGCACCTCAAATTTCGGGAAACCTCCTCGCTCACATGTAGGTGAAGCATCTTCCGGCTA
TCACAGCCACTTGTGTCTGCTAGGAGGCAGTGGTCCCTCCACTATTGCGATAAAGGAAAGGCGATGCT
GATCTTGCCTTCACTATCAGCGACTATATAAGGTTTTGCTATTCATTGAAAGCAGTAGTGACTGATTT
GTATATA
>MinSyn_1126|Strength:0.001372878
GCGTGTCGTTTTAGTGAGGTGAAGATAAGATAATAATGTTGAAGATAAGAAGGAGGACCGATAAAAAT
GTCAAAGATACTACAAAACTGGTACTTGTGGCACACCAGCATGTGTTGATCACCAGCTTGCTAGTCCA
TCAACAAATAATCCAAGTAAGAGGAGGACCGATGCTGATCTTGCAAGATTGATGAAAAGTCAAAAACA
AAAATCAATTATTTAGGTTGTCTGCACCCTATATAAGGTTTTGCTATTCATTGAAAGCAGTAGTGACT
GATTTGTATATA
>MinSyn_1317|Strength:0.001375497
GCGTGTCGTTTTAGTGAGGTGAAGCATCTTCCCTTAGCAAGACCTCTACAAAACTGGTACTTATGACC
CCCGCCGATGACGCGGGAAGCTTAGCAAGACCTCTACAAAAATTTCGGGAAACCTCCTCGGAGGACCT
TTTCAACAATATCAGCTTAGCAAGACCTCTACAGCCACTTGTGTATCTTGCTGAAGATAAGATAATAA
TGTTGAAGATAAGATAGGTCACGACCACTATGCAAATATTTCTTGTGCTTAGCAAGGCACACCAGCAT
GTGTTGATCACCAGCTGGCTCTATATAAGGTTTTGCTATTCATTGAAAGCAGTAGTGACTGATTTGTA
TATA
>MinSyn_1534|Strength:0.001376174
GCGTGTCGTTTTAGTGAGGGTGGGAGCCACCAAGGTCACGACCACTATGCCCAATTAACCATTATTGC
GCCTGCCTTGATGATATTATGACCCCCGCCGATGACGCGGGAATGCTGATCTTGCCTGCCTTGCACAC
CAGCATGTGTTGATCACCAGCTCACATGTAGGCTATATAAGGTTTTGCTATTCATTGAAAGCAGTAGT
GACTGATTTGTATATA
>MinSyn_1789|Strength:0.001376429
GCGTGTCGTTTTAGTGAGGAACCATTATTGCGGAAACCACGTCTACAACTCACATGTTCACTATCAGC
CTTAGCAAGACCTCTACAAACTATATAAGGTTTTGCTATTCATTGAAAGCAGTAGTGACTGATTTGTA
TATA
>MinSyn_1346|Strength:0.001377545
GCGTGTCGTTTTAGTGAGGGTGGGAGCCACCAGGAGGACCGCAGCCACTTGTGTCGCGGTAGGTCACG
AAACCATTATTGCGAGACCTCTACAGTGGTCCCTCCACATTATGACCCCCGCCGATGACGCGGGATAC
TTGTGTACAAACCACGTCTACAACAAAACTGGTACTTGTGTACAGGGCTTGGTGGAGCACGACAGACC
ACTATCCTTACCGCTATGGGTAAGATTGCTTCCATCAACAAATAATCCAAGTAAGAGCTTAGCAAGAC
CTCTACAAAACTATATAAGGTTTTGCTATTCATTGAAAGCAGTAGTGACTGATTTGTATATA
>MinSyn_1828|Strength:0.001378694
GCGTGTCGTTTTAGTGAGGTGGTGGAGCACGACATGTGTACAGGGTCCATCAACAAATAATCCAAGTA
AGGCCTGCCTTCAGAAGATCAAAGGGCTACCGATGCTGATCTAAGATTGATGAAAAGTCAAAAACAAA
AATCAATTATCAGCTTAGCCAAATATTTCTTGTCACTATGCCCAATTAGGTTGTCTGCACCTCCTATA
TAAGGTTTTGCTATTCATTGAAAGCAGTAGTGACTGATTTGTATATA
>MinSyn_1070|Strength:0.001382353
GCGTGTCGTTTTAGTGAGGAGGTGGCTCCTACGTACAGGGCTCATCTCTCTGCCGACAGTGGTCCCAA
AACATGTAGGCTATCACTATATAAGGTTTTGCTATTCATTGAAAGCAGTAGTGACTGATTTGTATATA
>MinSyn_1211|Strength:0.001386601
GCGTGTCGTTTTAGTGAGGAGGTGGCTCCTACCAATTAGGTTGTCTGCACCTCACTGAAGATAAGATA
ATAATGTTGAAGATAAGAGATTTATGACCCCCGCCGATGACGCGGGAAACTGGTACTTGTGTACAGGG
CTCACTGCAATTTCGGGAAACCTCCTCGTGCTAGGAGGACCGATAAGATTGATGAAAAGTCAAAAACA
AAAATCAATTATATCAGCTTAGCAAGAACCATTATTGCGGTAGGTCACCTATATAAGGTTTTGCTATT

CATTGAAAGCAGTAGTGACTGATTTGTATATA
>MinSyn_1280|Strength:0.00138751
GCGTGTCGTTTTAGTGAGGAGGTGGCTCCTACGGTAGGTCAAGATTGATGAAAAGTCAAAAACAAAAA
TCAATTATGACATCGAAAGGACAGTAGCCCAATTAGGTTGTGGTGGAGCACGACATGTGTACAGGGCT
CCTATATAAGGTTTTGCTATTCATTGAAAGCAGTAGTGACTGATTTGTATATA
>MinSyn_1285|Strength:0.001388297
GCGTGTCGTTTTAGTGAGGATCCTTACCGCTATGGGTAAGATTAAAACTGGTACTTGTGTACTCACTA
TCAGCCAGCTTAGCAAGACCTCTACAAAACTGATTGCGATAAAGGAAAGGAAACCATTATTGCGTGCA
TCGAAAGGACAGTACCTCACATGCACACCAGCATGTGTTGATCACCAGCTCCACTATGCCCAATTAGG
TTGTCTGCAAGATTGATGAAAAGTCAAAAACAAAAATCAATTATTCAGCTTAGCAGCCACTTGTGTGG
TAGGTCACGACCACTATATAAGGTTTTGCTATTCATTGAAAGCAGTAGTGACTGATTTGTATATA
>MinSyn_1620|Strength:0.001391135
GCGTGTCGTTTTAGTGAGGGGAAAAAGAAGAGGTTGTCTGCACCTCACATGGCACACCAGCATGTGTT
GATCACCAGCTACGACCACTATGCCCAATTTATGACCCCCGCCGATGACGCGGGAGGTAGGTCACGAC
CACTATAAAAATGTCAAAGATAGCTTAGCAAGACCTCTACTGGTGGAGCACGACAACGTGAAGATAAG
ATAATAATGTTGAAGATAAGAGGACCGATGCTTCCATCAACAAATAATCCAAGTAAGTGTAGGCTATC
AGCTTAGCAAGACCTATATAAGGTTTTGCTATTCATTGAAAGCAGTAGTGACTGATTTGTATATA
>MinSyn_1518|Strength:0.001396735
GCGTGTCGTTTTAGTGAGGTTATGACCCCCGCCGATGACGCGGGAGCCCAATTAGGTTGTCTGATCGA
AAGGACAGTAACAAAGGTGGCTCCTACCTATGCCTGGTGGAGCACGACAGTTGTCTGCACCTTTTTCA
ACAAGAGGACCGTCCATCAACAAATAATCCAAGTAAGTAGGCTCTATATAAGGTTTTGCTATTCATTG
AAAGCAGTAGTGACTGATTTGTATATA
>MinSyn_1578|Strength:0.00141
GCGTGTCGTTTTAGTGAGGTCAGAAGATCAAAGGGCTACGATGCTGATATCCTTACCGCTATGGGTAA
GATTCACTGCTAGGAGGACCGATGCTGATCCTATATAAGGTTTTGCTATTCATTGAAAGCAGTAGTGA
CTGATTTGTATATA
>MinSyn_1235|Strength:0.001418006
GCGTGTCGTTTTAGTGAGGGGAAAAAGAAGAGGTAATTCACTATCAGCGAGGACCGATGCTGATCTTG
CATTGCGATAAAGGAAAGGCGACCACTATGCCCAATTTGAAGATAAGATAATAATGTTGAAGATAAGA
CTCTACACTATATAAGGTTTTGCTATTCATTGAAAGCAGTAGTGACTGATTTGTATATA
>MinSyn_1666|Strength:0.001422533
GCGTGTCGTTTTAGTGAGGAGGTGGCTCCTACCCTCACATGTAGGCTATCAGCTTAGCATCTCTCTGC
CGACAGTGGTCCCAAAGCCTCAGTGGTCCCTCCACGTAGGTCACGACCACATTGCGATAAAGGAAAGG
GCTGATCTTGCCTTATGACCCCCGCCGATGACGCGGGAGGGCTCACTAACCATTATTGCGACCTATAT
AAGGTTTTGCTATTCATTGAAAGCAGTAGTGACTGATTTGTATATA
>MinSyn_1489|Strength:0.001423239
GCGTGTCGTTTTAGTGAGGAACCATTATTGCGCGCGGTAGGTCACGACCACGCACACCAGCATGTGTT
GATCACCAGCTATCAGCTTAGCCTATATAAGGTTTTGCTATTCATTGAAAGCAGTAGTGACTGATTTG
TATATA
>MinSyn_1878|Strength:0.001424307
GCGTGTCGTTTTAGTGAGGGCACACCAGCATGTGTTGATCACCAGCTATTAGGTTGTCAAGATTGATG
AAAAGTCAAAAACAAAAATCAATTATCGACCACTATGCCCAATGGAAAAAGAAGAGGTACTGCTAGGA
GGAAAAATGTCAAAGATACTGCCTATATAAGGTTTTGCTATTCATTGAAAGCAGTAGTGACTGATTTG
TATATA
>MinSyn_1549|Strength:0.00142526
GCGTGTCGTTTTAGTGAGGTCTCTCTGCCGACAGTGGTCCCAAAAGGCAGTGGTCCCTCCACCACCTC
ACATGTAGGCTATCAGCTATCCTTACCGCTATGGGTAAGATTGGTTGAACCATTATTGCGGCCTGCAA
ATATTTCTTGTTAGGTTGTCTGCACCTCACATCTATATAAGGTTTTGCTATTCATTGAAAGCAGTAGT
GACTGATTTGTATATA
>MinSyn_1448|Strength:0.001426437
GCGTGTCGTTTTAGTGAGGTGAAGATAAGATAATAATGTTGAAGATAAGACCGATGCTGATCTTGCCT
GCCTTGATGTTATGACCCCCGCCGATGACGCGGGAAACTGGTACGGAAAAAGAAGAGGTCCTCTACAA
AACTGGTAATCCTTACCGCTATGGGTAAGATTTTGTCTGCACCTGAAGCATCTTCCTTAGGTAAGATT
GATGAAAAGTCAAAAACAAAAATCAATTATACATCAGCCACTTGTGTCTACAAAACTGGTACTTGTGT

ACAGGAAAAATGTCAAAGATAGTACAGTCTCTCTGCCGACAGTGGTCCCAAATGCACCTCACATGTCT
ATATAAGGTTTTGCTATTCATTGAAAGCAGTAGTGACTGATTTGTATATA
>MinSyn_1412|Strength:0.001430075
GCGTGTCGTTTTAGTGAGGATCCTTACCGCTATGGGTAAGATTTTGTCTGCACCTCACCAGTGGTCCC
TCCACTACTTGTGGTGGAGCACGACAAGACCTCTACAAAACTAGGTGGCTCCTACGTAGCTATATAAG
GTTTTGCTATTCATTGAAAGCAGTAGTGACTGATTTGTATATA
>MinSyn_1230|Strength:0.001430435
GCGTGTCGTTTTAGTGAGGTCACTATCAGCCGCGGTAGGTCACGACCACTATGGGAAAAAGAAGAGGT
CAGGTGGCTCCTACGTAGCTATATAAGGTTTTGCTATTCATTGAAAGCAGTAGTGACTGATTTGTATA
TA
>MinSyn_1940|Strength:0.001430435
GCGTGTCGTTTTAGTGAGGATTGCGATAAAGGAAAGGAATGGTGGAGCACGACAAAGACCTCTCTATA
TAAGGTTTTGCTATTCATTGAAAGCAGTAGTGACTGATTTGTATATA
>MinSyn_1674|Strength:0.00143262
GCGTGTCGTTTTAGTGAGGGCTTTGTCAAAAGCTAAAAAAGATGATGCTGCCTTATGACCCCCGCCGA
TGACGCGGGAGCTTAGCAAGACCTAGGTGGCTCCTACTCAGTGGTCCCTCCACCACATGTAGGCTATC
TATATAAGGTTTTGCTATTCATTGAAAGCAGTAGTGACTGATTTGTATATA
>MinSyn_1169|Strength:0.001441601
GCGTGTCGTTTTAGTGAGGTGGTGGAGCACGACAAATTTTCAACAAGGGCTCACTGATTGCGATAAAG
GAAAGGGTACTTGTGTAACCATTATTGCGTGTGTACAGGGCTCACTGCTTGAAGATAAGATAATAATG
TTGAAGATAAGATGGCTATATAAGGTTTTGCTATTCATTGAAAGCAGTAGTGACTGATTTGTATATA
>MinSyn_1213|Strength:0.001443571
GCGTGTCGTTTTAGTGAGGAACCACGTCTACAAGCCTGCCTTGACAGCCACTTGTGTTGTACAGAGGT
GGCTCCTACCACGACCACTACTATATAAGGTTTTGCTATTCATTGAAAGCAGTAGTGACTGATTTGTA
TATA
>MinSyn_1649|Strength:0.001444946
GCGTGTCGTTTTAGTGAGGCAGTGGTCCCTCCACGGACCGATGCTGATCTTGAAGATAAGATAATAAT
GTTGAAGATAAGATTATCGAAAGGACAGTACTAGGAGGACCGATGCTGATCCTATATAAGGTTTTGCT
ATTCATTGAAAGCAGTAGTGACTGATTTGTATATA
>MinSyn_1553|Strength:0.00144513
GCGTGTCGTTTTAGTGAGGTGAAGATAAGATAATAATGTTGAAGATAAGAGCGGTAGGTCTTTTCAAC
AAGATGCTGATCTTGCCTATCGAAAGGACAGTACCACTATGCCCAATTATGACCCCCGCCGATGACGC
GGGACGTGGTGGAGCACGACAAATTAATCCTTACCGCTATGGGTAAGATTCGATGCTGATCTTGCCTG
CCTTGATAACCACGTCTACAATCTATATAAGGTTTTGCTATTCATTGAAAGCAGTAGTGACTGATTTG
TATATA
>MinSyn_1685|Strength:0.001451513
GCGTGTCGTTTTAGTGAGGGTGGGAGCCACCAACCTCACTTTTCAACAAAGCTTAGCAAGACCTCTAC
AAAACTATTGCGATAAAGGAAAGGGAAAGATTGATGAAAAGTCAAAAACAAAAATCAATTATGCCTTG
ATCGAAAGGACAGTAAGGTCACGACCAAGCAAGTGGATAGGATCCTTACCGCTATGGGTAAGATTTAC
TTGTCAGAAGATCAAAGGGCTATTTATGACCCCCGCCGATGACGCGGGAAGGACCGATGCTGATCTTG
CCTGCTATATAAGGTTTTGCTATTCATTGAAAGCAGTAGTGACTGATTTGTATATA
>MinSyn_1300|Strength:0.001452033
GCGTGTCGTTTTAGTGAGGTTTTCAACAAGATGCTGATCTTTATGACCCCCGCCGATGACGCGGGACT
GCTCTATATAAGGTTTTGCTATTCATTGAAAGCAGTAGTGACTGATTTGTATATA
>MinSyn_1275|Strength:0.001473729
GCGTGTCGTTTTAGTGAGGTCCATCAACAAATAATCCAAGTAAGATGTAGGCTTTTTCAACAAGGTCT
ATATAAGGTTTTGCTATTCATTGAAAGCAGTAGTGACTGATTTGTATATA
>MinSyn_1123|Strength:0.001477798
GCGTGTCGTTTTAGTGAGGAACCACGTCTACAAGTACAGGGCTCAAGATTGATGAAAAGTCAAAAACA
AAAATCAATTATAGGACCGATGCCAGCCACTTGTGTTCACTGCTAGGAGGACCGACTATATAAGGTTT
TGCTATTCATTGAAAGCAGTAGTGACTGATTTGTATATA
>MinSyn_1311|Strength:0.001492174
GCGTGTCGTTTTAGTGAGGTTATGACCCCCGCCGATGACGCGGGACTCACTGCTAGGAGGTGAAGCAT
CTTCCTAGGTCACGACCACTATGCTGAAGATAAGATAATAATGTTGAAGATAAGAAAACTGGAACCAT

TATTGCGGATCTTGCCTGCCTTGATGACAGTGGTCCCTCCACTAGGCTATATAAGGTTTTGCTATTCA
TTGAAAGCAGTAGTGACTGATTTGTATATA
>MinSyn_1080|Strength:0.001493798
GCGTGTCGTTTTAGTGAGGCAAATATTTCTTGTCTATCAGCTTAGCAAGACTCTCTCTGCCGACAGTG
GTCCCAAAGCTATATAAGGTTTTGCTATTCATTGAAAGCAGTAGTGACTGATTTGTATATA
>MinSyn_1112|Strength:0.001495455
GCGTGTCGTTTTAGTGAGGTGGTGGAGCACGACAAGGAGGACCGATTCCATCAACAAATAATCCAAGT
AAGCCTCACATGCTATATAAGGTTTTGCTATTCATTGAAAGCAGTAGTGACTGATTTGTATATA
>MinSyn_1426|Strength:0.00150514
GCGTGTCGTTTTAGTGAGGAAGATTGATGAAAAGTCAAAAACAAAAATCAATTATTACAAAACTGGTA
TCGAAAGGACAGTAGACCTCTACAAAACTGGTACTTGTGTACAGAAAAATGTCAAAGATAGACATCCT
TACCGCTATGGGTAAGATTACATGTAGCACACCAGCATGTGTTGATCACCAGCTCGACCACTATGCCT
ATATAAGGTTTTGCTATTCATTGAAAGCAGTAGTGACTGATTTGTATATA
>MinSyn_1905|Strength:0.001516186
GCGTGTCGTTTTAGTGAGGTCAGAAGATCAAAGGGCTAAGCAAGACCTCATTGCGATAAAGGAAAGGC
GACCACTATGCCCAATTAGGTTGTCTGCATTATGACCCCCGCCGATGACGCGGGACTGATCTTGAATT
TCGGGAAACCTCCTCGGGAGGACCGGTGGGAGCCACCAGACTATATAAGGTTTTGCTATTCATTGAAA
GCAGTAGTGACTGATTTGTATATA
>MinSyn_1414|Strength:0.001516778
GCGTGTCGTTTTAGTGAGGATCCTTACCGCTATGGGTAAGATTGCTCACTGCTAGGAGGACCGATGCT
GATCTCTCTGCCGACAGTGGTCCCAAAACTGCTAGTTATGACCCCCGCCGATGACGCGGGAAGGACCG
ATGCTGATCTTTCCATCAACAAATAATCCAAGTAAGGAGCTATATAAGGTTTTGCTATTCATTGAAAG
CAGTAGTGACTGATTTGTATATA
>MinSyn_1184|Strength:0.001522488
GCGTGTCGTTTTAGTGAGGATCCTTACCGCTATGGGTAAGATTCTGGTACTTGCACACCAGCATGTGT
TGATCACCAGCTGTGTACAGGGCTCACTGCTAGGAGGTGGGAGCCACCACTGCTAGGCAGTGGTCCCT
CCACACATGAAGCATCTTCCCATGTAGGCTTTTCAACAAATCTATATAAGGTTTTGCTATTCATTGAA
AGCAGTAGTGACTGATTTGTATATA
>MinSyn_1978|Strength:0.001528429
GCGTGTCGTTTTAGTGAGGTCCATCAACAAATAATCCAAGTAAGCCTATCGAAAGGACAGTAGCCCAA
TTAGGTTGTCTGCACCTCACAAAGATTGATGAAAAGTCAAAAACAAAAATCAATTATGAGGACCGATA
TTGCGATAAAGGAAAGGACTGGTACCTATATAAGGTTTTGCTATTCATTGAAAGCAGTAGTGACTGAT
TTGTATATA
>MinSyn_1380|Strength:0.001529024
GCGTGTCGTTTTAGTGAGGAAGATTGATGAAAAGTCAAAAACAAAAATCAATTATACCGCACACCAGC
ATGTGTTGATCACCAGCTCACTATGCCCAATTAGGTTGTCTGCACAACCACGTCTACAACTAGCTATA
TAAGGTTTTGCTATTCATTGAAAGCAGTAGTGACTGATTTGTATATA
>MinSyn_1440|Strength:0.001530233
GCGTGTCGTTTTAGTGAGGAAAAATGTCAAAGATATTGCCTGCATCCTTACCGCTATGGGTAAGATTT
GTACAGGGCCTATATAAGGTTTTGCTATTCATTGAAAGCAGTAGTGACTGATTTGTATATA
>MinSyn_1662|Strength:0.001531061
GCGTGTCGTTTTAGTGAGGTTATGACCCCCGCCGATGACGCGGGACTTGTGTACAGGGCTCATCGAAA
GGACAGTATGCCCTATATAAGGTTTTGCTATTCATTGAAAGCAGTAGTGACTGATTTGTATATA
>MinSyn_1133|Strength:0.001540984
GCGTGTCGTTTTAGTGAGGTGAAGCATCTTCCTACTTGTTCTCTCTGCCGACAGTGGTCCCAAAACCT
CTCTATATAAGGTTTTGCTATTCATTGAAAGCAGTAGTGACTGATTTGTATATA
>MinSyn_1236|Strength:0.001553959
GCGTGTCGTTTTAGTGAGGATTGCGATAAAGGAAAGGCGGAACCATTATTGCGCTTGTGTACTGAAGA
TAAGATAATAATGTTGAAGATAAGATAGGTCACGACCACCTATATAAGGTTTTGCTATTCATTGAAAG
CAGTAGTGACTGATTTGTATATA
>MinSyn_1874|Strength:0.001554615
GCGTGTCGTTTTAGTGAGGTTATGACCCCCGCCGATGACGCGGGAGATCGAAAGGACAGTACTTAGCA
AGACCTCTACCTATATAAGGTTTTGCTATTCATTGAAAGCAGTAGTGACTGATTTGTATATA
>MinSyn_1143|Strength:0.001555396

GCGTGTCGTTTTAGTGAGGAACCATTATTGCGAGCAAGTGAAGATAAGATAATAATGTTGAAGATAAG
ACAGCTTAGCAAGACCTCTCTATATAAGGTTTTGCTATTCATTGAAAGCAGTAGTGACTGATTTGTAT
ATA
>MinSyn_1929|Strength:0.001556146
GCGTGTCGTTTTAGTGAGGAAAAATGTCAAAGATATGTCTCTCTGCCGACAGTGGTCCCAAATTGGTG
GGAGCCACCATGCCTGCCTTGATGATTCAGAAGATCAAAGGGCTATTAGGTAATTTCGGGAAACCTCC
TCGGTCCTATATAAGGTTTTGCTATTCATTGAAAGCAGTAGTGACTGATTTGTATATA
>MinSyn_1477|Strength:0.001556808
GCGTGTCGTTTTAGTGAGGAATTTCGGGAAACCTCCTCGTACAGGGCTCACTGCTAGGAGGACCGATC
AGAAGATCAAAGGGCTACGATGCTGATCTTGAAGATTGATGAAAAGTCAAAAACAAAAATCAATTATA
TGCTATATAAGGTTTTGCTATTCATTGAAAGCAGTAGTGACTGATTTGTATATA
>MinSyn_1718|Strength:0.001557292
GCGTGTCGTTTTAGTGAGGTGAAGATAAGATAATAATGTTGAAGATAAGAAACTGGTTCACTATCAGC
ACTATGCCCAATTTCCATCAACAAATAATCCAAGTAAGCTCACATTTATGACCCCCGCCGATGACGCG
GGAACTTGTGTACAGGGCTCTATATAAGGTTTTGCTATTCATTGAAAGCAGTAGTGACTGATTTGTAT
ATA
>MinSyn_1416|Strength:0.001567477
GCGTGTCGTTTTAGTGAGGTCCATCAACAAATAATCCAAGTAAGTCAGCTTTCTCTCTGCCGACAGTG
GTCCCAAAGGGAAAAAGAAGAGGTGTACAGGGCTCACTGCTAGGAGAAGATTGATGAAAAGTCAAAAA
CAAAAATCAATTATCTCAGAAGATCAAAGGGCTAACCGATGCTGATCTTGCCTGCCTATATAAGGTTT
TGCTATTCATTGAAAGCAGTAGTGACTGATTTGTATATA
>MinSyn_1096|Strength:0.001577622
GCGTGTCGTTTTAGTGAGGGCACACCAGCATGTGTTGATCACCAGCTCGATGCTGATCTTGCATTGCG
ATAAAGGAAAGGCTGCACCTCACCTATATAAGGTTTTGCTATTCATTGAAAGCAGTAGTGACTGATTT
GTATATA
>MinSyn_1732|Strength:0.001577631
GCGTGTCGTTTTAGTGAGGAAGATTGATGAAAAGTCAAAAACAAAAATCAATTATATTAGGTTGTCTT
CCATCAACAAATAATCCAAGTAAGAGGAGGAAACCACGTCTACAATAGGAGGACCGGCACACCAGCAT
GTGTTGATCACCAGCTCAAAACTGGCTATATAAGGTTTTGCTATTCATTGAAAGCAGTAGTGACTGAT
TTGTATATA
>MinSyn_1188|Strength:0.001596257
GCGTGTCGTTTTAGTGAGGTGAAGCATCTTCCCCAAGATTGATGAAAAGTCAAAAACAAAAATCAATT
ATCCTCTACAAAACAATTTCGGGAAACCTCCTCGCGCGGTAGGTCACGACCACTATGCCCACTATATA
AGGTTTTGCTATTCATTGAAAGCAGTAGTGACTGATTTGTATATA
>MinSyn_1785|Strength:0.0015981
GCGTGTCGTTTTAGTGAGGATCCTTACCGCTATGGGTAAGATTTAGGTCACGACCACTATGCCCAATT
AGTTATGACCCCCGCCGATGACGCGGGAGATGCTGAAAAAATGTCAAAGATATCGCGAGGTGGCTCCT
ACTCTCTATATAAGGTTTTGCTATTCATTGAAAGCAGTAGTGACTGATTTGTATATA
>MinSyn_1573|Strength:0.001608725
GCGTGTCGTTTTAGTGAGGAAGATTGATGAAAAGTCAAAAACAAAAATCAATTATCAAGACCTCTACA
AAACTGGTATGAAGCATCTTCCGGTACTTCTATATAAGGTTTTGCTATTCATTGAAAGCAGTAGTGAC
TGATTTGTATATA
>MinSyn_1932|Strength:0.001612409
GCGTGTCGTTTTAGTGAGGAACCACGTCTACAACCTCACATGTAGGCTATAAAAATGTCAAAGATACG
GCAAATATTTCTTGTGCCTATATAAGGTTTTGCTATTCATTGAAAGCAGTAGTGACTGATTTGTATAT
A
>MinSyn_1491|Strength:0.001617532
GCGTGTCGTTTTAGTGAGGTGAAGATAAGATAATAATGTTGAAGATAAGATAGGTCACGACCACTATG
CCCAATTAAATTTCGGGAAACCTCCTCGATCTTGCTATATAAGGTTTTGCTATTCATTGAAAGCAGTA
GTGACTGATTTGTATATA
>MinSyn_1222|Strength:0.001618033
GCGTGTCGTTTTAGTGAGGCAGTGGTCCCTCCACTGCACCTCTCCATCAACAAATAATCCAAGTAAGC
TCCTATATAAGGTTTTGCTATTCATTGAAAGCAGTAGTGACTGATTTGTATATA
>MinSyn_1547|Strength:0.001618889

GCGTGTCGTTTTAGTGAGGTGAAGATAAGATAATAATGTTGAAGATAAGAGCTGATCTTGCCTGCCTT
GATGTGGTGGAGCACGACACTCTACAAAACTGGTACTTGTGTACAGTCCATCAACAAATAATCCAAGT
AAGTAGGAGGACCGATATCGAAAGGACAGTACCGCACACCAGCATGTGTTGATCACCAGCTTCTATAT
AAGGTTTTGCTATTCATTGAAAGCAGTAGTGACTGATTTGTATATA
>MinSyn_1610|Strength:0.001629839
GCGTGTCGTTTTAGTGAGGAAAAATGTCAAAGATAGGACCGATTCTCTCTGCCGACAGTGGTCCCAAA
AGGCCTATATAAGGTTTTGCTATTCATTGAAAGCAGTAGTGACTGATTTGTATATA
>MinSyn_1848|Strength:0.001631405
GCGTGTCGTTTTAGTGAGGCAGTGGTCCCTCCACGCGGTATCTCTCTGCCGACAGTGGTCCCAAAATG
CCTATATAAGGTTTTGCTATTCATTGAAAGCAGTAGTGACTGATTTGTATATA
>MinSyn_1498|Strength:0.001648052
GCGTGTCGTTTTAGTGAGGATCCTTACCGCTATGGGTAAGATTCTGATCTTGCCTGCAACCACGTCTA
CAAACAGAACCATTATTGCGCTCTACAAAACTGGCTATATAAGGTTTTGCTATTCATTGAAAGCAGTA
GTGACTGATTTGTATATA
>MinSyn_1715|Strength:0.001649669
GCGTGTCGTTTTAGTGAGGTTATGACCCCCGCCGATGACGCGGGAACTGGTACTTGTGTACAGGGCTC
CATCAACAAATAATCCAAGTAAGAGGCTATCCTATATAAGGTTTTGCTATTCATTGAAAGCAGTAGTG
ACTGATTTGTATATA
>MinSyn_1597|Strength:0.001681434
GCGTGTCGTTTTAGTGAGGTCCATCAACAAATAATCCAAGTAAGTTAGGTTTCAGAAGATCAAAGGGC
TATCACTGCTAGGAGGACCGTGAAGATAAGATAATAATGTTGAAGATAAGAGCTATATAAGGTTTTGC
TATTCATTGAAAGCAGTAGTGACTGATTTGTATATA
>MinSyn_1483|Strength:0.00169109
GCGTGTCGTTTTAGTGAGGCTGACGTAAGGGATGACGCACATGTGTATTATGACCCCCGCCGATGACG
CGGGATTGTGGGAGCCACCACCACTATGCCCAATTAGGTTGTCATTGCGATAAAGGAAAGGCTTGCCT
GCCTTGACAGCCACTTGTGTATGAAGATAAGATAATAATGTTGAAGATAAGAATTAGGTTGTCTGCAC
CTCACATATCCTTACCGCTATGGGTAAGATTTTAGCAAGACCTCTACAAAGGTGGCTCCTACCCACTA
TGCCCAATTAGGTTGTCTGCAACCACGTCTACAAACCTCACATGTAGGCTATCAGCTTAGCACTATAT
AAGGTTTTGCTATTCATTGAAAGCAGTAGTGACTGATTTGTATATA
>MinSyn_1738|Strength:0.001698319
GCGTGTCGTTTTAGTGAGGTTATGACCCCCGCCGATGACGCGGGAGTGTGGAAAAAGAAGAGGTGCTC
TATATAAGGTTTTGCTATTCATTGAAAGCAGTAGTGACTGATTTGTATATA
>MinSyn_1550|Strength:0.001709091
GCGTGTCGTTTTAGTGAGGAACCACGTCTACAATCACTGCTAGGAGGACCAAGATTGATGAAAAGTCA
AAAACAAAAATCAATTATCACTACTATATAAGGTTTTGCTATTCATTGAAAGCAGTAGTGACTGATTT
GTATATA
>MinSyn_1682|Strength:0.001709091
GCGTGTCGTTTTAGTGAGGTGAAGATAAGATAATAATGTTGAAGATAAGACACCTCACATGTTCAGAA
GATCAAAGGGCTACCAATTAGGTCTATATAAGGTTTTGCTATTCATTGAAAGCAGTAGTGACTGATTT
GTATATA
>MinSyn_1797|Strength:0.001715328
GCGTGTCGTTTTAGTGAGGAATTTCGGGAAACCTCCTCGACCTCAGCACACCAGCATGTGTTGATCAC
CAGCTACGACCACTATGCTATATAAGGTTTTGCTATTCATTGAAAGCAGTAGTGACTGATTTGTATAT
A
>MinSyn_1220|Strength:0.001726531
GCGTGTCGTTTTAGTGAGGATCCTTACCGCTATGGGTAAGATTGCTGATCTTGGCACACCAGCATGTG
TTGATCACCAGCTACTTGTGTACAGGGCTATATAAGGTTTTGCTATTCATTGAAAGCAGTAGTGACTG
ATTTGTATATA
>MinSyn_1561|Strength:0.001728503
GCGTGTCGTTTTAGTGAGGTTATGACCCCCGCCGATGACGCGGGAATCTTGCCTGAAAAATGTCAAAG
ATATAGGTGAAGATAAGATAATAATGTTGAAGATAAGATGCACCTATATAAGGTTTTGCTATTCATTG
AAAGCAGTAGTGACTGATTTGTATATA
>MinSyn_1643|Strength:0.001741958
GCGTGTCGTTTTAGTGAGGAATTTCGGGAAACCTCCTCGGTCTGCACCTCACATGTGAAGATAAGATA

ATAATGTTGAAGATAAGATGATACTATATAAGGTTTTGCTATTCATTGAAAGCAGTAGTGACTGATTT
GTATATA
>MinSyn_1870|Strength:0.001758015
GCGTGTCGTTTTAGTGAGGATCGAAAGGACAGTAACAATTGCGATAAAGGAAAGGTGTACAGGGTGGG
AGCCACCAGTCCTATATAAGGTTTTGCTATTCATTGAAAGCAGTAGTGACTGATTTGTATATA
>MinSyn_1504|Strength:0.001777342
GCGTGTCGTTTTAGTGAGGCAGCCACTTGTGTACTGCTAGAATTTCGGGAAACCTCCTCGCAAAACTG
AAGATAAGATAATAATGTTGAAGATAAGATGGCTATATAAGGTTTTGCTATTCATTGAAAGCAGTAGT
GACTGATTTGTATATA
>MinSyn_1444|Strength:0.001777612
GCGTGTCGTTTTAGTGAGGTTATGACCCCCGCCGATGACGCGGGACTCTACAAAACTTTTTCAACAAG
GTGAAGCATCTTCCCGCACACCAGCATGTGTTGATCACCAGCTGCAAGACCTATATAAGGTTTTGCTA
TTCATTGAAAGCAGTAGTGACTGATTTGTATATA
>MinSyn_1637|Strength:0.001805298
GCGTGTCGTTTTAGTGAGGTCCATCAACAAATAATCCAAGTAAGCGCAAATATTTCTTGTGTCACGAC
CACTATGCCCAAAAAATGTCAAAGATAGGTCCTATATAAGGTTTTGCTATTCATTGAAAGCAGTAGTG
ACTGATTTGTATATA
>MinSyn_1407|Strength:0.001807692
GCGTGTCGTTTTAGTGAGGTCTCTCTGCCGACAGTGGTCCCAAATAGCACACCAGCATGTGTTGATCA
CCAGCTTCAGCTTAGCAAGACCTCTATATAAGGTTTTGCTATTCATTGAAAGCAGTAGTGACTGATTT
GTATATA
>MinSyn_1661|Strength:0.001832768
GCGTGTCGTTTTAGTGAGGAATTTCGGGAAACCTCCTCGTTTGAAGATAAGATAATAATGTTGAAGAT
AAGACTTGTGTACAGGGCTTCCATCAACAAATAATCCAAGTAAGCCCCTATATAAGGTTTTGCTATTC
ATTGAAAGCAGTAGTGACTGATTTGTATATA
>MinSyn_1185|Strength:0.001833828
GCGTGTCGTTTTAGTGAGGTGGTGGAGCACGACACCTCTACAAAACTAAAAATGTCAAAGATAATCAG
CCACTTGTGTTAATTTCGGGAAACCTCCTCGTGTACAGTCTCTCTGCCGACAGTGGTCCCAAATAGGC
TATATAAGGTTTTGCTATTCATTGAAAGCAGTAGTGACTGATTTGTATATA
>MinSyn_1747|Strength:0.001852555
GCGTGTCGTTTTAGTGAGGTTATGACCCCCGCCGATGACGCGGGACTTTTCAACAACCGATGCTGATC
TGGAAAAAGAAGAGGTACTATATAAGGTTTTGCTATTCATTGAAAGCAGTAGTGACTGATTTGTATAT
A
>MinSyn_1067|Strength:0.001909375
GCGTGTCGTTTTAGTGAGGCAAATATTTCTTGTACAAGATTGATGAAAAGTCAAAAACAAAAATCAAT
TATTTGTGCTATATAAGGTTTTGCTATTCATTGAAAGCAGTAGTGACTGATTTGTATATA
>MinSyn_1413|Strength:0.001958333
GCGTGTCGTTTTAGTGAGGGCACACCAGCATGTGTTGATCACCAGCTACTTGTGTCCATCAACAAATA
ATCCAAGTAAGGCTATATAAGGTTTTGCTATTCATTGAAAGCAGTAGTGACTGATTTGTATATA
>MinSyn_1313|Strength:0.001960571
GCGTGTCGTTTTAGTGAGGGTGGGAGCCACCATCATCCTTACCGCTATGGGTAAGATTTGGATTGCGA
TAAAGGAAAGGAGACAACCATTATTGCGAGCTATATAAGGTTTTGCTATTCATTGAAAGCAGTAGTGA
CTGATTTGTATATA
>MinSyn_1751|Strength:0.002088889
GCGTGTCGTTTTAGTGAGGATCCTTACCGCTATGGGTAAGATTCGTCACTATCAGCGTACCAGTGGTC
CCTCCACACAGGGCTAACCACGTCTACAATACTCTATATAAGGTTTTGCTATTCATTGAAAGCAGTAG
TGACTGATTTGTATATA
>MinSyn_1502|Strength:0.002184507
GCGTGTCGTTTTAGTGAGGAGGTGGCTCCTACCAATCGAAAGGACAGTATTTTTCAACAACTGGTATC
CTTACCGCTATGGGTAAGATTACTATATAAGGTTTTGCTATTCATTGAAAGCAGTAGTGACTGATTTG
TATATA
>MinSyn_1182|Strength:0.002802625
GCGTGTCGTTTTAGTGAGGATGAGCTAAGCACATACGTCAGGGGCTCACTGCTAGGAGGACCGATGCT
GTCCATCAACAAATAATCCAAGTAAGGCTATCAAAAAATGTCAAAGATACCAATTAGGTTGTCTGCAC

TTATGACCCCCGCCGATGACGCGGGATCACTGCTAGGAGGACCGATGCTGATCTTAAGATTGATGAAA
AGTCAAAAACAAAAATCAATTATCAATCGAAAGGACAGTACAAGACCTCTACAAAACTGGTACTCACT
ATCAGCGTACTTGTGTCTATATAAGGTTTTGCTATTCATTGAAAGCAGTAGTGACTGATTTGTATATA
>MinSyn_1795|Strength:0.003231172
GCGTGTCGTTTTAGTGAGGCTGACGTAAGGGATGACGCACAGGTACTTGTGTACAGGGCTCACTGAGC
AAGTGGATTTAGGTTGTCTGCACCTCACATGTAGGCTTTATGACCCCCGCCGATGACGCGGGAACTGG
TACTTGTGAACCATTATTGCGATTAGGTTGGCTTTGTCAAAAGCTAAAAAAGATGATGCTAGCAAGAC
CTCTGCACACCAGCATGTGTTGATCACCAGCTGGTCACGACCACTCAGTGGTCCCTCCACGACCACTA
TGCCCAATTAGGTTGTCTGCACTGAAGCATCTTCCACAAAACTGGCTATATAAGGTTTTGCTATTCAT
TGAAAGCAGTAGTGACTGATTTGTATATA
>MinSyn_1442|Strength:0.004153684
GCGTGTCGTTTTAGTGAGGCAGTGGTCCCTCCACTTAGGTTGTCTGCACCTCACATGACGTAAGCCAT
GACGTCTAGGACCGATGCTGATAAGATTGATGAAAAGTCAAAAACAAAAATCAATTATCTATATTGCG
ATAAAGGAAAGGTTAGCAAGACCTCTACAAAACTAACCACGTCTACAACAATTAGGTTGTCTGCACCT
CACATCGAAAGGACAGTACCTCTACAAAACTGGTACTTGCACACCAGCATGTGTTGATCACCAGCTAG
GTCACGACCACTATGCCCAATTAGGTTGTGAAGCATCTTCCTGCCCAATTAGGTTGTCTGCACCTCAC
ACTATATAAGGTTTTGCTATTCATTGAAAGCAGTAGTGACTGATTTGTATATA
>MinSyn_1244|Strength:0.004264256
GCGTGTCGTTTTAGTGAGGAGCAAGTGGATTACTTCTGACGTAAGGGATGACGCACAGTTGTCTGCAC
TTTTCAACAACAAAACTGAACCATTATTGCGCTGCTAGGAGGACCGATGCTGACAAATATTTCTTGTT
GCTGATCTTGCCTGCCTTGAATTTCGGGAAACCTCCTCGGTGTACAGGGCTCACTGCTCAGCCACTTG
TGTACCGATGCTGATCTTGCCTGCCTTGTGAAGATAAGATAATAATGTTGAAGATAAGAGCGGTAGGT
CACGACCACTGTGGGAGCCACCACACTATGCCCAATTAGGTTGTCTGCACCCTATATAAGGTTTTGCT
ATTCATTGAAAGCAGTAGTGACTGATTTGTATATA
>MinSyn_1652|Strength:0.0044974
GCGTGTCGTTTTAGTGAGGAAAAATGTCAAAGATATTAGCAAGACCTATGACGTAAGCCATGACGTCT
AAATTTTATGACCCCCGCCGATGACGCGGGAAGGTCACGACCACTATGCCCAAACCACGTCTACAAAA
CTGGTACTTGTGTACAGGTGAAGCATCTTCCGCAAGACCTCTACAAGCACACCAGCATGTGTTGATCA
CCAGCTTTGTGTACAGGGCTCACTGCTAGGAGGCTTTGTCAAAAGCTAAAAAAGATGATGCCACGACC
ACTAATCCTTACCGCTATGGGTAAGATTCCGATGCTGATCTTGCCTGCCTTGATGATACTATATAAGG
TTTTGCTATTCATTGAAAGCAGTAGTGACTGATTTGTATATA
>MinSyn_1062|Strength:0.004752577
GCGTGTCGTTTTAGTGAGGATGACGTAAGCCATGACGTCTATACAAAACTGGTACTTGTGTAGCTTTG
TCAAAAGCTAAAAAAGATGATGCACCGATGCTGATCAAAAATGTCAAAGATACACATGTAGGCTATCA
GCTTTGGTGGAGCACGACATGTGTACAGGGCCAGCCACTTGTGTTTAGGTTGTCTGCACCTCATCCAT
CAACAAATAATCCAAGTAAGCGATGCTGATCTTGCCTGCCTGAAGCATCTTCCGACTCAGAAGATCAA
AGGGCTATACTTGTGTACAGGGCTCACTGCTACTATATAAGGTTTTGCTATTCATTGAAAGCAGTAGT
GACTGATTTGTATATA
>MinSyn_1186|Strength:0.00510202
GCGTGTCGTTTTAGTGAGGAGGTGGCTCCTACTGGTACTTGTGTACAGGGCTATGACGTAAGCCATGA
CGTCTAAAACTGGTACTTGTGTACAGGAACCACGTCTACAATTTTTCAACAATGTGTACAGGGCTCAC
TGCTAGGTCTCTCTGCCGACAGTGGTCCCAAAATGCTGGCTTTGTCAAAAGCTAAAAAAGATGATGCA
TGCTGATCTTGCCTGCAAGATTGATGAAAAGTCAAAAACAAAAATCAATTATTTGTGTACAGGGCTCA
CAGCCACTTGTGTGTCTCAGAAGATCAAAGGGCTACCAATTAGGTTGTCTGCACCTCACCTATATAAG
GTTTTGCTATTCATTGAAAGCAGTAGTGACTGATTTGTATATA
>MinSyn_1190|Strength:0.005211617
GCGTGTCGTTTTAGTGAGGCAGCCACTTGTGTCAGCTTAGCAAGACCTCTACATGAGCTAAGCACATA
CGTCAGGACCAGTGGTCCCTCCACCCAATTAGGTTGTCTGCACCTCTGGTGGAGCACGACAACCACTA
TGCCCAATTAGGTTGTCTGCACTCTCTCTGCCGACAGTGGTCCCAAAGTTGAAGCATCTTCCCTACAA
AACTGGTACTTGTGTACAGGGCTCAGAAGATCAAAGGGCTAACTGCTAGGAGGACCGATTTTCAACAA
TAGCAAGACCTCTACAAAACTGGTACCTATATAAGGTTTTGCTATTCATTGAAAGCAGTAGTGACTGA
TTTGTATATA
>MinSyn_1246|Strength:0.006209338
GCGTGTCGTTTTAGTGAGGCTGACGTAAGGGATGACGCACAATCAGCTTATGAAGATAAGATAATAAT

GTTGAAGATAAGACGACCACTATGCCCAATTAGGTTGTCTAGCAAGTGGATGGCTTTGTCAAAAGCTA
AAAAAGATGATGCGTCAGCCACTTGTGTTTGTCTGCACCTCACATGTAGGCTCACTATCAGCTTGCCA
AGATTGATGAAAAGTCAAAAACAAAAATCAATTATGCTATCAGCTTAGCTCCATCAACAAATAATCCA
AGTAAGGTACAGGGCTCACTGCTATATAAGGTTTTGCTATTCATTGAAAGCAGTAGTGACTGATTTGT
ATATA
>MinSyn_1072|Strength:0.006318253
GCGTGTCGTTTTAGTGAGGTTATGACCCCCGCCGATGACGCGGGAACTGGTACTTGTGTACAGGGCTT
CAGAAGATCAAAGGGCTAAGCCAGCCACTTGTGTCCTCTACAAAACTGGTACTGACGTAAGGGATGAC
GCACACTTAGCAAGACCTCTACAAAACTGGTATCTCTCTGCCGACAGTGGTCCCAAATGTGTACAGGG
CTCACTGCTAGGAGGAAAGATTGATGAAAAGTCAAAAACAAAAATCAATTATCTATCAGCTTAGCAAG
ACCTCAAAAATGTCAAAGATACCAATTAGGTTGTCTGCACCTCACATGTATGAAGATAAGATAATAAT
GTTGAAGATAAGACTGGTACTTGTGTACAGGGCTCACTCTATATAAGGTTTTGCTATTCATTGAAAGC
AGTAGTGACTGATTTGTATATA
>MinSyn_1487|Strength:0.00667425
GCGTGTCGTTTTAGTGAGGGCTTTGTCAAAAGCTAAAAAAGATGATGCTATGCCCAATTAGGTTGTCT
GATGAGCTAAGCACATACGTCAGACCACTATGCCCAATTAGGTTGTCAAATATTTCTTGTTATGCCCA
ATTAGGTTGTCTGCATCAGAAGATCAAAGGGCTAGGTCACGACCACTATGATCCTTACCGCTATGGGT
AAGATTTCACTGCTAGGAGGACCGATGCTGATCTCCATCAACAAATAATCCAAGTAAGTTGTCTGGCA
CACCAGCATGTGTTGATCACCAGCTGCCCAATCTATATAAGGTTTTGCTATTCATTGAAAGCAGTAGT
GACTGATTTGTATATA
>MinSyn_1499|Strength:0.006795702
GCGTGTCGTTTTAGTGAGGCTGACGTAAGGGATGACGCACACTGATCTTGCCAGGTGGCTCCTACGCT
ACAAATATTTCTTGTGTAGGCGTGGGAGCCACCAGCCTCTCTCTGCCGACAGTGGTCCCAAACTCACT
GCTAGGAGGACCGATGCTGATGAAGCATCTTCCGGCTCACTGCTAGGAGGACCGATGCTGAATTTCGG
GAAACCTCCTCGTCACTGCTAGGAGGACCGATGCTGATCTTGCTTTGTCAAAAGCTAAAAAAGATGAT
GCATCAGCTTAGCTATATAAGGTTTTGCTATTCATTGAAAGCAGTAGTGACTGATTTGTATATA
>MinSyn_1396|Strength:0.00679873
GCGTGTCGTTTTAGTGAGGAAGATTGATGAAAAGTCAAAAACAAAAATCAATTATGCACCTCACATGT
AGGCTATCAGCTTATGAAGATAAGATAATAATGTTGAAGATAAGACTCAGCCACTTGTGTTACAAAAC
TGGTACTTGTGTATGACGTAAGCCATGACGTCTAACAAAACTGGTACTTGTGTAAAAAATGTCAAAGA
TAAGAATTTCGGGAAACCTCCTCGGCTTAGCAAGACCTCTACAAAACTGGTAATCCTTACCGCTATGG
GTAAGATTTAGCAAGACCTCTACAAAACTGGTCAGTGGTCCCTCCACATGCCCAATTAGGTTGTCTGC
ACCGCACACCAGCATGTGTTGATCACCAGCTGTAGGCTATCAGCTTAGCAAGACCTCTCTATATAAGG
TTTTGCTATTCATTGAAAGCAGTAGTGACTGATTTGTATATA
>MinSyn_1368|Strength:0.006924539
GCGTGTCGTTTTAGTGAGGAATTTCGGGAAACCTCCTCGACCACTATGCCCAATTAGGTTGTCTGATG
AGCTAAGCACATACGTCAGAAACTGGTACTGTGGGAGCCACCATAAAAAATGTCAAAGATATAGGTCA
CGACCACTATGCATCCTTACCGCTATGGGTAAGATTCAAGACCTCTACAAAATGGTGGAGCACGACAC
ACTGCTAGGAGGACCGATGCTGTCAGAAGATCAAAGGGCTACTATGCCCAATTAGGTTGTGAAGATAA
GATAATAATGTTGAAGATAAGACCACTCTATATAAGGTTTTGCTATTCATTGAAAGCAGTAGTGACTG
ATTTGTATATA
>MinSyn_1544|Strength:0.007112572
GCGTGTCGTTTTAGTGAGGCTGACGTAAGGGATGACGCACACCTCACAGCCACTTGTGTTTGCCTGCC
TTGATGATCCTTACCGCTATGGGTAAGATTTGCCTTGGCTTTGTCAAAAGCTAAAAAAGATGATGCTG
CCCAATTAGGTTAAAAATGTCAAAGATATGCCTTGAAACCATTATTGCGAAACTGGTACTTGTGTACA
GGGCAAATATTTCTTGTATGCCCAATTAGGTTGTTCAGAAGATCAAAGGGCTAAACTGGTACTTGTGT
ACAGGGCTCACCTATATAAGGTTTTGCTATTCATTGAAAGCAGTAGTGACTGATTTGTATATA
>MinSyn_1418|Strength:0.007198842
GCGTGTCGTTTTAGTGAGGATCCTTACCGCTATGGGTAAGATTATGCTGATCTTGCCTGCAAATATTT
CTTGTCAGCTTAGCAAGACCTCTACATGACGTAAGCCATGACGTCTATCTGCACCTCAGAAGATCAAA
GGGCTATTAGCAAGACCTCTATCCATCAACAAATAATCCAAGTAAGTAGGAGGACCGATGCTAGGTGG
CTCCTACGACCTCTACAAAACAAGATTGATGAAAAGTCAAAAACAAAAATCAATTATGCTATCAGCTT
AGGGAAAAAGAAGAGGTCTAGGAGGACCGATGCTGATCAGCAAGTGGATGATGCTGATCTTGCCTGCC
TTGATGCTATATAAGGTTTTGCTATTCATTGAAAGCAGTAGTGACTGATTTGTATATA

>MinSyn_1503|Strength:0.007241484
GCGTGTCGTTTTAGTGAGGCAAATATTTCTTGTAAGACCTATCGAAAGGACAGTATAGGTCACGACCA
CTATGCCCAATTATCCATCAACAAATAATCCAAGTAAGATATGACGTAAGCCATGACGTCTAGGGCTC
ACTGCTAGGAGGACCGATGCTGAAGATAAGATAATAATGTTGAAGATAAGATAGGTCACGACCACTAT
GCCCAATTAAACCACGTCTACAAACCGATGCTGATCTTGCCTGCCTTAGGTGGCTCCTACTTGTGTAC
AGGGCTCACTGCTGAAGCATCTTCCGTACTTGTGTACAGGGCTCACTGCTAGGATCAGAAGATCAAAG
GGCTACTGATCTTGCCTGCTATATAAGGTTTTGCTATTCATTGAAAGCAGTAGTGACTGATTTGTATA
TA
>MinSyn_1689|Strength:0.00725493
GCGTGTCGTTTTAGTGAGGAGCAAGTGGATTATCATGAGCTAAGCACATACGTCAGCTGATCTTGCCT
GCCTTGATGAATTTCGGGAAACCTCCTCGTAGGAGGACCGATGCTGATCTTGCCTGCCTGAAGATAAG
ATAATAATGTTGAAGATAAGATGATCTTGTCCATCAACAAATAATCCAAGTAAGTTGTGTACAGGGCT
CACCAGCCACTTGTGTCCCAATGCTTTGTCAAAAGCTAAAAAAGATGATGCGATGCTGATCTTCTATA
TAAGGTTTTGCTATTCATTGAAAGCAGTAGTGACTGATTTGTATATA
>MinSyn_1459|Strength:0.007405516
GCGTGTCGTTTTAGTGAGGATCCTTACCGCTATGGGTAAGATTTGCTAGGAGGACCGATGCTGATCTT
GCCATGACGTAAGCCATGACGTCTACTCACATCCATCAACAAATAATCCAAGTAAGCCGATGCTGATC
TTGCCTGCCTTGATAACCATTATTGCGCTTAGCAAGACCTCTACAAAACTGGTTGAAGCATCTTCCAC
CTCTACAAAACTGGTACTTATGACCCCCGCCGATGACGCGGGACATGTAGGCTCTCTCTGCCGACAGT
GGTCCCAAAATGCTGATCTTGGGAAAAAGAAGAGGTAATTAGGTTGTCTGCACCTCTATATAAGGTTT
TGCTATTCATTGAAAGCAGTAGTGACTGATTTGTATATA
>MinSyn_1048|Strength:0.007982987
GCGTGTCGTTTTAGTGAGGTCAGAAGATCAAAGGGCTAGGTCACGACCACTATGCCCAATTACTGACG
TAAGGGATGACGCACATGCCCTGAAGATAAGATAATAATGTTGAAGATAAGAAGGCTATCAGCTTCAC
TATCAGCCCAGCCACTTGTGTTTAGCAAGACCTCTACAAAATCGAAAGGACAGTACACTAGCACACCA
GCATGTGTTGATCACCAGCTGGTAGAAGATTGATGAAAAGTCAAAAACAAAAATCAATTATGCACCTC
ACATGTAGGCTATCAGCTTAGCAGCAAGTGGATGCACCTCACACTATATAAGGTTTTGCTATTCATTG
AAAGCAGTAGTGACTGATTTGTATATA
>MinSyn_1772|Strength:0.008165953
GCGTGTCGTTTTAGTGAGGAACCATTATTGCGCTTGCCTGCCTTGATGCAGCCACTTGTGTTAGGAGG
ACCGATGCTGATCTTGCCTGCCTGACGTAAGGGATGACGCACAACTATGCCCAATTAGGTTGTCTGTG
AAGCATCTTCCCTGCTAGGAGGACCGATGCTGATTTTCAACAACACTGCTAGGAGGACCGATGCTGTG
GTGGAGCACGACACAAAACTGGTACTTGGCTTTGTCAAAAGCTAAAAAAGATGATGCCGATGCTGATC
TTGCCTGCCTAATTTCGGGAAACCTCCTCGCAGGTGGCTCCTACTCACATGTAGGCTATCCTATATAA
GGTTTTGCTATTCATTGAAAGCAGTAGTGACTGATTTGTATATA
>MinSyn_1174|Strength:0.008625626
GCGTGTCGTTTTAGTGAGGATGACGTAAGCCATGACGTCTACCTGCCTTGATGATCTCTCTGCCGACA
GTGGTCCCAAAAGGTCATCAGAAGATCAAAGGGCTACCCAATTAGGTTGTCTGCACCTGCTTTGTCAA
AAGCTAAAAAAGATGATGCTAGGCTATCAGCTTAAACCATTATTGCGACTATGCCCAATTAGTTATGA
CCCCCGCCGATGACGCGGGAATTAGGTTGTGAAGCATCTTCCATGTAGGCTATCAGCTTAGCAAGACC
CTATATAAGGTTTTGCTATTCATTGAAAGCAGTAGTGACTGATTTGTATATA
>MinSyn_1314|Strength:0.008645313
GCGTGTCGTTTTAGTGAGGATCCTTACCGCTATGGGTAAGATTAAGACCTCTACAATTATGACCCCCG
CCGATGACGCGGGATGTGATGACGTAAGCCATGACGTCTAATGCTGATCTTGCCTGCCTTGTCTCTCT
GCCGACAGTGGTCCCAAATGTCTGCACCTCACCAAATATTTCTTGTGTCACGACCACTATGCCCAATT
AGGTTCAGTGGTCCCTCCACAAGACCTCTAGCACACCAGCATGTGTTGATCACCAGCTCGATGCTGAT
CTTGCCTGCCTTGATGATAAATTTCGGGAAACCTCCTCGCAAAACTGGTACTTGTGCTATATAAGGTT
TTGCTATTCATTGAAAGCAGTAGTGACTGATTTGTATATA
>MinSyn_1479|Strength:0.00871201
GCGTGTCGTTTTAGTGAGGAACCATTATTGCGTGCACCTCACATGTAGGCTATCAGTGGTCCCTCCAC
TTGATGACGTAAGCCATGACGTCTATGCATCAGAAGATCAAAGGGCTAGTGTACAGGGCTCACTGCTA
GGATTTTCAACAATTGTATCGAAAGGACAGTATGTAGGCTATCAGCTTAGCAACAAATATTTCTTGTC
ACTGCTAGGAGGACCGATGCAGCAAGTGGATGCTCACTGCTAGGAAGATTGATGAAAAGTCAAAAACA
AAAATCAATTATAGACCTCTACAAAACTGGTACTTGTGTACACTATATAAGGTTTTGCTATTCATTGA

AAGCAGTAGTGACTGATTTGTATATA
>MinSyn_1454|Strength:0.008969715
GCGTGTCGTTTTAGTGAGGATGACGTAAGCCATGACGTCTAAGACCTCTACAAAACTGGTACTTGAAC
CATTATTGCGTGTAAACCACGTCTACAAGGTACTTGTGTACAGGGCTCACATCCTTACCGCTATGGGT
AAGATTTGGTGGGAGCCACCAAGACCTGAAGCATCTTCCATGCCCAATTAGGTTGTCTAGCAAGTGGA
TCTCTACAAAACTGGTACTTGTGTACAGGGTTTTCAACAAACAAAACTGGTACTTGTGTACAGGCTAT
ATAAGGTTTTGCTATTCATTGAAAGCAGTAGTGACTGATTTGTATATA
>MinSyn_1488|Strength:0.009129715
GCGTGTCGTTTTAGTGAGGAACCACGTCTACAAGACCTCTACAAAACTGGTACTTGTGTGAAGCATCT
TCCACTGACGTAAGGGATGACGCACATCACATGTAGGCTATCAAATATTTCTTGTTATTTTCAACAAG
CTAGGAGGACCGATGCTGATCTTGCCTTCCATCAACAAATAATCCAAGTAAGCTCTACAAATGGTGGA
GCACGACACACTATGCTCTCTCTGCCGACAGTGGTCCCAAACCTCTACAAAACTGGTACTTGTGTCAG
TGGTCCCTCCACTGTGTACAGGGCTCACTGCTAGGAGGACCTATATAAGGTTTTGCTATTCATTGAAA
GCAGTAGTGACTGATTTGTATATA
>MinSyn_1157|Strength:0.009257531
GCGTGTCGTTTTAGTGAGGATGAGCTAAGCACATACGTCAGACGACTTATGACCCCCGCCGATGACGC
GGGAAACTGGTACTTGTTCTCTCTGCCGACAGTGGTCCCAAAGACCTCTACAATGAAGCATCTTCCAA
TTAGGTTGTCTGCACCTCACATTTTTCAACAACTGATCTTGCCTGCCTTGATGGCTTTGTCAAAAGCT
AAAAAAGATGATGCTAGGTCACGACCACTATGCCCACTATATAAGGTTTTGCTATTCATTGAAAGCAG
TAGTGACTGATTTGTATATA
>MinSyn_1851|Strength:0.009264639
GCGTGTCGTTTTAGTGAGGCTGACGTAAGGGATGACGCACAACAGGGCTCCATCAACAAATAATCCAA
GTAAGATTAGGTTGTCTGCACCTCACATGTAGGAAAAATGTCAAAGATATGTACAGGGCTCAAATTTC
GGGAAACCTCCTCGATGTAGGCTATGTGGGAGCCACCAGGTTGTCTGCACCTCACATGTAGGCTATCA
TCACTATCAGCCCGATGCTGATCTTGCAGCAAGTGGATTCACTGCTAAACCATTATTGCGATGCTATA
TAAGGTTTTGCTATTCATTGAAAGCAGTAGTGACTGATTTGTATATA
>MinSyn_1564|Strength:0.0098076
GCGTGTCGTTTTAGTGAGGATGACGTAAGCCATGACGTCTAGCTATCAGCTTAGCAAGACCTCTCACT
ATCAGCCACTGCTAGAGGTGGCTCCTACAACTGGTACTTGTGTACAGAGCAAGTGGATTAGGTTGTCT
GCACCTCACATAACCACGTCTACAAGCACCTCACATGTAGGCTATCAGGAAAAAGAAGAGGTGGGCTC
ACTGCTAGGAGCTTTGTCAAAAGCTAAAAAAGATGATGCAGCAAGACCTCTACAAACTATATAAGGTT
TTGCTATTCATTGAAAGCAGTAGTGACTGATTTGTATATA
>MinSyn_1780|Strength:0.00981818
GCGTGTCGTTTTAGTGAGGTCTCTCTGCCGACAGTGGTCCCAAAATCTTGCCTGCCTTGATAACCATT
ATTGCGTGCCTTGACTGACGTAAGGGATGACGCACATCAGCTTAGCAAGACCTCTACAAAACTGTGAA
GATAAGATAATAATGTTGAAGATAAGATAGGTCACGATCAGAAGATCAAAGGGCTAACCACTGGTGGA
GCACGACATCATCGAAAGGACAGTACTTCACTATCAGCTGTACAGGGCTCACTGCTAGGTTATGACCC
CCGCCGATGACGCGGGAGCTTAGCAAGACCTCTACAAAACTGGTCTATATAAGGTTTTGCTATTCATT
GAAAGCAGTAGTGACTGATTTGTATATA
>MinSyn_1303|Strength:0.010214276
GCGTGTCGTTTTAGTGAGGTTATGACCCCCGCCGATGACGCGGGACCTCACATGTAGGCTATCAGCTT
TGTCAAAAGCTAAAAAAGATGATGCCACTATGCCCAATTATGACGTAAGCCATGACGTCTAAGGTCAC
GAAAGATTGATGAAAAGTCAAAAACAAAAATCAATTATCGACCACTATGAAAAATGTCAAAGATAGCG
GTAGGTCACGACCACTATGCGGAAAAAGAAGAGGTGGTCACGACCACTATGCCCTCAGAAGATCAAAG
GGCTATGTGTACAGGGCTCACTGTCCATCAACAAATAATCCAAGTAAGATGCTGATCTTGCTATATAA
GGTTTTGCTATTCATTGAAAGCAGTAGTGACTGATTTGTATATA
>MinSyn_1270|Strength:0.010526518
GCGTGTCGTTTTAGTGAGGTCAGAAGATCAAAGGGCTACCGATGCTGATCTTGCCTGCCTTATGAGCT
AAGCACATACGTCAGTCACAAATATTTCTTGTTCACATGTAGGCTATCAGCTTAGCAAGACTCACTAT
CAGCCATATTGCGATAAAGGAAAGGTATGCCCAATTAGGTTGTCTGCACGTGGGAGCCACCATGCTAG
GAGGACCGATGCAAAAATGTCAAAGATACCCATTTTCAACAAGACCGATGCTGATCTTGCCTGCCCTA
TATAAGGTTTTGCTATTCATTGAAAGCAGTAGTGACTGATTTGTATATA
>MinSyn_1420|Strength:0.010542649
GCGTGTCGTTTTAGTGAGGCAGTGGTCCCTCCACACTATGCCCAATTAGGTTGTCTGCACTGACGTAA

GGGATGACGCACAAGGGCTCACTGCTAGGAGGACCGATGCTGATCACTATCAGCCTGCACCTCACATG
TAATCGAAAGGACAGTATGTGTGTGGGAGCCACCAACAGGGCTCACTGCTAGGAGGACCGATGAATTT
CGGGAAACCTCCTCGTAGGAGGACCGATGCTGGTGGAGCACGACATCACGACCACTATGCCCAATTAG
GAACCATTATTGCGCTTGCTATATAAGGTTTTGCTATTCATTGAAAGCAGTAGTGACTGATTTGTATA
TA
>MinSyn_1746|Strength:0.010709748
GCGTGTCGTTTTAGTGAGGATTGCGATAAAGGAAAGGGACCACTATGCCCAATTAGGTTGTCTCCATC
AACAAATAATCCAAGTAAGCAAAACTGGTACATCCTTACCGCTATGGGTAAGATTTTGTCTGCACCTG
CTTTGTCAAAAGCTAAAAAAGATGATGCACGACCACTATGCCCTGACGTAAGGGATGACGCACAAAAA
GATTGATGAAAAGTCAAAAACAAAAATCAATTATACATGTAGGCTATCAGCTTAGCAAGACCTTTTCA
ACAAGGGCTCACTGCTAGGAGGACCGATGCTGATCGAAAGGACAGTAGCTTAGCAAGACCTCTACAAA
ACTGGTAAACCATTATTGCGGCACCTCACATGTAGGCTATCCTATATAAGGTTTTGCTATTCATTGAA
AGCAGTAGTGACTGATTTGTATATA
>MinSyn_1227|Strength:0.010722348
GCGTGTCGTTTTAGTGAGGTTATGACCCCCGCCGATGACGCGGGAAAGACCTCTACAACAGTGGTCCC
TCCACGGTACTTGTGTACAGGGCTCACTGCTATGAAGATAAGATAATAATGTTGAAGATAAGACCAAT
TAGGTTGTCTGCATGACGTAAGCCATGACGTCTATGGTACTTGTGTACAGGGCTAAGATTGATGAAAA
GTCAAAAACAAAAATCAATTATATTAGGTTGTCTGCACCTCACATGTATTTTCAACAAATTAGGTTGT
CTGCACCTCACATGTAGGCTTGGTGGAGCACGACAAGCAAGACCTCTACAAAACTGGTACTTGTAATT
TCGGGAAACCTCCTCGACGCTATATAAGGTTTTGCTATTCATTGAAAGCAGTAGTGACTGATTTGTAT
ATA
>MinSyn_1714|Strength:0.010909608
GCGTGTCGTTTTAGTGAGGAAGATTGATGAAAAGTCAAAAACAAAAATCAATTATGACCTCTACAAAA
CTGGTACATGACGTAAGCCATGACGTCTATGCTAGGAGGGCTTTGTCAAAAGCTAAAAAAGATGATGC
TCACTAACCATTATTGCGTCTGCACCTCACATGTAGGCTATCAGCTATCGAAAGGACAGTACTTGATA
TCCTTACCGCTATGGGTAAGATTTGCCCAATTAGGTTGCAGTGGTCCCTCCACGCTGATCTTGCCAGC
CACTTGTGTTACTTGTGTACAGGGCTCACTATATAAGGTTTTGCTATTCATTGAAAGCAGTAGTGACT
GATTTGTATATA
>MinSyn_1061|Strength:0.011355143
GCGTGTCGTTTTAGTGAGGATCCTTACCGCTATGGGTAAGATTCACGACCTGGTGGAGCACGACAATT
AGGTTGTCTGCACCTCATGAGCTAAGCACATACGTCAGAATTAGGTTGTCTTATGACCCCCGCCGATG
ACGCGGGAGCACCTCTCCATCAACAAATAATCCAAGTAAGTACGCACACCAGCATGTGTTGATCACCA
GCTGCTATCAGCTTAGCAAGACCTTTTCAACAAGCTTAGCAAGACCTCTACAAAACTGGTAAAAATGT
CAAAGATAACAGCTATATAAGGTTTTGCTATTCATTGAAAGCAGTAGTGACTGATTTGTATATA
>MinSyn_1434|Strength:0.0117363
GCGTGTCGTTTTAGTGAGGAAGATTGATGAAAAGTCAAAAACAAAAATCAATTATGGAGGACCGATGT
CCATCAACAAATAATCCAAGTAAGTAGGTTGTCTGCACCTCACATGTTATGACCCCCGCCGATGACGC
GGGATACAAAACTGGTACTCTGACGTAAGGGATGACGCACAGGTCACGACCACTATGCAAATATTTCT
TGTACCACTATGCCCAATTAAGCAAGTGGATAAGACCTCTACAAAACTCAGCCACTTGTGTTATCGAA
AGGACAGTATAGGCTATCAGCTTAGCAATCTCTCTGCCGACAGTGGTCCCAAAAAGACCTCTACAAAA
CTGGTACTTGTGTACCTATATAAGGTTTTGCTATTCATTGAAAGCAGTAGTGACTGATTTGTATATA
>MinSyn_1104|Strength:0.011749036
GCGTGTCGTTTTAGTGAGGATGACGTAAGCCATGACGTCTATTGCCTGCTGAAGATAAGATAATAATG
TTGAAGATAAGAGGCTCACTATTGCGATAAAGGAAAGGACAAAACTGGTACTTGTGTAAAGATTGATG
AAAAGTCAAAAACAAAAATCAATTATTCTGCACCTCACATGTAGGCTATTCAGAAGATCAAAGGGCTA
GCCTGTCTCTCTGCCGACAGTGGTCCCAAAGCGGTAGGTCACGACCACTCTATATAAGGTTTTGCTAT
TCATTGAAAGCAGTAGTGACTGATTTGTATATA
>MinSyn_1168|Strength:0.011782049
GCGTGTCGTTTTAGTGAGGTGAAGATAAGATAATAATGTTGAAGATAAGAACCGATGCTGACAAATAT
TTCTTGTGCTTAGCAAGACCTCTACAAAACTGGTAAATTTCGGGAAACCTCCTCGGTCACGACCACTA
TGCCATGAGCTAAGCACATACGTCAGTAGGCTATCAGAGGTGGCTCCTACAAACTGGTACTTGTGTAC
AGGGCTCTGAAGCATCTTCCTCTTGCCTGCCTTGAAGATTGATGAAAAGTCAAAAACAAAAATCAATT
ATACCTCACATGTAGGCTATCAGCTTAGCAATGGTGGAGCACGACACGCGGTACTATATAAGGTTTTG
CTATTCATTGAAAGCAGTAGTGACTGATTTGTATATA

>MinSyn_1668|Strength:0.012095567
GCGTGTCGTTTTAGTGAGGATCGAAAGGACAGTAACATGACGTAAGCCATGACGTCTATTGTGTACAG
GGCTCACTGCTAGGTCCATCAACAAATAATCCAAGTAAGTATCTCAGAAGATCAAAGGGCTAACTTGT
GTACAGGGCTCACTGCTAGTCTCTCTGCCGACAGTGGTCCCAAAACGACCACTATTATGACCCCCGCC
GATGACGCGGGAAGGTCACGACAAATATTTCTTGTTCACATGTAGGCTATCAGCTTAGCCTATATAAG
GTTTTGCTATTCATTGAAAGCAGTAGTGACTGATTTGTATATA
>MinSyn_1792|Strength:0.012237512
GCGTGTCGTTTTAGTGAGGAGCAAGTGGATTCAGCTTAGCAAATGACGTAAGCCATGACGTCTAGTCA
CGACCACTATGCAAAAATGTCAAAGATACTCTCAGTGGTCCCTCCACAAAACTGGTACTTGTGTACAG
GGCTCAGAAGATCAAAGGGCTAACTATGCCCAATTAGGTTGTCTGCAAGGTGGCTCCTACGGAGGACC
GATGCTGCAAATATTTCTTGTACAGGGCTCACTGCTGGTGGAGCACGACACAAGACCTCTACTATATA
AGGTTTTGCTATTCATTGAAAGCAGTAGTGACTGATTTGTATATA
>MinSyn_1121|Strength:0.01231575
GCGTGTCGTTTTAGTGAGGAAAAATGTCAAAGATATGTACAGGGCTCACTGCTAGGAGGCAAATATTT
CTTGTAAAACTGGTACTTGTGTACAGGGCTCACTGATGAGCTAAGCACATACGTCAGATGATATTATG
ACCCCCGCCGATGACGCGGGAGGTTGTCTGCACCTCACAATCGAAAGGACAGTATGGTACTTGTGTAC
AGGGCTCACTGCGCACACCAGCATGTGTTGATCACCAGCTCTTAGCAAGACCTCTACAAATTGCGATA
AAGGAAAGGTAGGTCACGACCTATATAAGGTTTTGCTATTCATTGAAAGCAGTAGTGACTGATTTGTA
TATA
>MinSyn_1020|Strength:0.012581168
GCGTGTCGTTTTAGTGAGGAAAAATGTCAAAGATAACGACCACTATGCCCAATTAGCTGACGTAAGGG
ATGACGCACAAGCAAGAAACCACGTCTACAAAGCTTAGCAATGAAGCATCTTCCCACGACCACTATGC
CCAATTAGGTCAGTGGTCCCTCCACGCAAGACCTCTACAAAACTGGTATGGTGGAGCACGACAACCAC
TATGCCCAATTAGGTTGTCTAGGTGGCTCCTACTCATCGAAAGGACAGTAGTAGGTCACGACCACTAT
GCTATATAAGGTTTTGCTATTCATTGAAAGCAGTAGTGACTGATTTGTATATA
>MinSyn_1263|Strength:0.012762558
GCGTGTCGTTTTAGTGAGGAACCACGTCTACAAACTGGTACTTGTGTAAATTTCGGGAAACCTCCTCG
GGGCTCACTGCCTGACGTAAGGGATGACGCACACACATGTAGGCTATCAGAAAAATGTCAAAGATAAT
CAGGGAAAAAGAAGAGGTAAATGGTGGAGCACGACACACCTCACATTGAAGCATCTTCCGTAGGTCAC
GACCACTATGCTCACTATCAGCACCTCACATGTAGGCTATCAGCTTAGCAAAGATTGATGAAAAGTCA
AAAACAAAAATCAATTATCCTATATAAGGTTTTGCTATTCATTGAAAGCAGTAGTGACTGATTTGTAT
ATA
>MinSyn_1065|Strength:0.012788347
GCGTGTCGTTTTAGTGAGGTGAAGCATCTTCCAGCAAGACCTCTACAAAACTGGTACTTGAAGATTGA
TGAAAAGTCAAAAACAAAAATCAATTATTAGGTTGTCTGCACCTCACATGTAGTCTCTCTGCCGACAG
TGGTCCCAAAGGTTGCTTTGTCAAAAGCTAAAAAAGATGATGCGGCTCATGAGCTAAGCACATACGTC
AGTTAGGTTGTCTGCACCTCACGTGGGAGCCACCAAGGACCGATGCTGATCTTGCCTGCCTTGAATTG
CGATAAAGGAAAGGCGATGCTGATCTTTGAAGATAAGATAATAATGTTGAAGATAAGATATGCCCAAT
TAGGTTGTCTGCTATATAAGGTTTTGCTATTCATTGAAAGCAGTAGTGACTGATTTGTATATA
>MinSyn_1445|Strength:0.012854288
GCGTGTCGTTTTAGTGAGGATGACGTAAGCCATGACGTCTACCAATTAGGTTGTCCAGCCACTTGTGT
TGCAAACCACGTCTACAACAGCTTAGCAAGACCTCATCCTTACCGCTATGGGTAAGATTAGGCTATCA
GCTTAGCAAAAAAATGTCAAAGATATTGTGTATTATGACCCCCGCCGATGACGCGGGATGCTGATCTT
GCCTGCCTTGAATCGAAAGGACAGTATGTACAGGGCTCACTCTATATAAGGTTTTGCTATTCATTGAA
AGCAGTAGTGACTGATTTGTATATA
>MinSyn_1897|Strength:0.012971484
GCGTGTCGTTTTAGTGAGGAGCAAGTGGATGGAGGACTGACGTAAGGGATGACGCACACAATTAGGTT
GTCTGCAGCACACCAGCATGTGTTGATCACCAGCTACCGATGCTTTGTCAAAAGCTAAAAAAGATGAT
GCGGTCACGACCACTATGCCCAATTAATTTCGGGAAACCTCCTCGCTGCACCTCACATGTAGGCTTGA
AGCATCTTCCTAAAAATGTCAAAGATAGCTGATCTTGCCTGCCTTGATGATCTATATAAGGTTTTGCT
ATTCATTGAAAGCAGTAGTGACTGATTTGTATATA
>MinSyn_1114|Strength:0.013122754
GCGTGTCGTTTTAGTGAGGTCCATCAACAAATAATCCAAGTAAGTCAGCTTAGCAAGACCTCTACCTG
ACGTAAGGGATGACGCACATACAAAACTGGTACTTGTGTACAGAACCACGTCTACAATGTGTACAGGG

CTCACTGCTAGGAGGACTGGTGGAGCACGACAGACCACTATGTCTCTCTGCCGACAGTGGTCCCAAAT
ATCAGCTTAGCCAGCCACTTGTGTCGATGCTGATCTTGCCTGCCTTGATGATTTTTCAACAACTCACT
GCTACTATATAAGGTTTTGCTATTCATTGAAAGCAGTAGTGACTGATTTGTATATA
>MinSyn_1849|Strength:0.013222254
GCGTGTCGTTTTAGTGAGGATTGCGATAAAGGAAAGGTAGGAGGACCGATGCTGATCAGCCACTTGTG
TTAGGAGGACCGATGACGTAAGCCATGACGTCTAACTGCTAGGAGGACCGATGCTTCACTATCAGCCA
GGGCTCACTGCTAGATCGAAAGGACAGTACTGCTAGGAGGACCGATTCAGAAGATCAAAGGGCTAACA
AAACTGGTACTTGTGTAATTTCGGGAAACCTCCTCGTTGTGTACAGTTATGACCCCCGCCGATGACGC
GGGACTGCCTTGATGACTATATAAGGTTTTGCTATTCATTGAAAGCAGTAGTGACTGATTTGTATATA
>MinSyn_1495|Strength:0.013454555
GCGTGTCGTTTTAGTGAGGCTGACGTAAGGGATGACGCACACTGGTACTTGTGTACAGGGCTGTGGGA
GCCACCACTGATCTTGCTGAAGATAAGATAATAATGTTGAAGATAAGAGACCGATGCTGGTGGAGCAC
GACAAAGACCTCCAGCCACTTGTGTCAATTAGGTTGTCTGCACCTCACATGTCACTATCAGCATGCTG
ATCTTGCCTTTTTCAACAACCCAATTAGGTTGTCTGCTATATAAGGTTTTGCTATTCATTGAAAGCAG
TAGTGACTGATTTGTATATA
>MinSyn_1975|Strength:0.013672905
GCGTGTCGTTTTAGTGAGGCTGACGTAAGGGATGACGCACACTGGTACTTGTGTACAGTGGTGGAGCA
CGACAAGGGCTCACTGTCTCTCTGCCGACAGTGGTCCCAAACATGTAGATCGAAAGGACAGTACTTGG
GAAAAAGAAGAGGTTGCTGATCTTGCCTGCCTTGATGAAACCACGTCTACAAATCTTGCCAGGTGGCT
CCTACTGTAGGCTATCAGCTTAGCAAGACCTCTACCTATATAAGGTTTTGCTATTCATTGAAAGCAGT
AGTGACTGATTTGTATATA
>MinSyn_1402|Strength:0.013690818
GCGTGTCGTTTTAGTGAGGATGACGTAAGCCATGACGTCTAGCCCAATTAGGTTGTCTGCACCTCACA
TTATGACCCCCGCCGATGACGCGGGATCAGCTTAAAAAATGTCAAAGATAGTCTGCACCTCACATGTA
GGCTATCAGTGAAGCATCTTCCCCTCTACAAAACTGATCGAAAGGACAGTAATGCTGATCTTAGCAAG
TGGATAGGTTGTCTGCACCTCACATGTAGGCTACTATATAAGGTTTTGCTATTCATTGAAAGCAGTAG
TGACTGATTTGTATATA
>MinSyn_1200|Strength:0.014244046
GCGTGTCGTTTTAGTGAGGTCCATCAACAAATAATCCAAGTAAGACTTGTGTACTGAAGATAAGATAA
TAATGTTGAAGATAAGACTAGGAGCTGACGTAAGGGATGACGCACACTGATCTTGCCTCAAATATTTC
TTGTTCACTGCTAGGAGGCACACCAGCATGTGTTGATCACCAGCTCAGCTTAGCAAGACCTCTAAACC
ATTATTGCGCACGACCACTATGCAGCCACTTGTGTGCACCTCACATGTATCACTATCAGCAGGTTGTC
TGCACCTCACATGTAGGCTATATAAGGTTTTGCTATTCATTGAAAGCAGTAGTGACTGATTTGTATAT
A
>MinSyn_1759|Strength:0.014334976
GCGTGTCGTTTTAGTGAGGTCAGAAGATCAAAGGGCTAGCGGTAGGTCACGACCACTATGCCCACTGA
CGTAAGGGATGACGCACACGGTAGGTCACGACCACTATGCTCACTATCAGCTACTTGTGTACAGGGCT
CACTGCTTCCATCAACAAATAATCCAAGTAAGCTTAGCAAGACCTTATGACCCCCGCCGATGACGCGG
GAGGTTGTCTGCACCTCAAAGATTGATGAAAAGTCAAAAACAAAAATCAATTATTCACGACCACTCTA
TATAAGGTTTTGCTATTCATTGAAAGCAGTAGTGACTGATTTGTATATA
>MinSyn_1551|Strength:0.014474524
GCGTGTCGTTTTAGTGAGGTGAAGATAAGATAATAATGTTGAAGATAAGAGCACCTCACATGTAGGCT
ATCAGCTTAGCACAAATATTTCTTGTGGAGGACCGATGCTGATCTCTCTGCCGACAGTGGTCCCAAAC
TGATCTTAGGTGGCTCCTACTAGGCTAATTGCGATAAAGGAAAGGGTCTGCACCTCACATGTAGGCTG
ACGTAAGGGATGACGCACAAGACCTCTACAAAACTGGTACTTGTGTCCATCAACAAATAATCCAAGTA
AGACATGTAGGCTATCAGCTTAGCAAGAAAAATGTCAAAGATAGGTAGGTCACGACCACTATGCCCAA
TTAGGTGGGAGCCACCACTGATCTTGCTATATAAGGTTTTGCTATTCATTGAAAGCAGTAGTGACTGA
TTTGTATATA
>MinSyn_1760|Strength:0.01451124
GCGTGTCGTTTTAGTGAGGTCCATCAACAAATAATCCAAGTAAGAGGCTATCAGCTGAAGCATCTTCC
TGATCTTGCCTGCCTTGATGATTGCGATAAAGGAAAGGGGCTGTGGGAGCCACCACTAGGAGGACATG
ACGTAAGCCATGACGTCTATGAAGATTGATGAAAAGTCAAAAACAAAAATCAATTATAGCACACCAGC
ATGTGTTGATCACCAGCTGGTAGGTCACGACCACTATGCCCAGGAAAAAGAAGAGGTTGCCCAATTAG
GTTGTCTGAACCACGTCTACAATGGTACTTGTGTACAGGGCTCACTCTATATAAGGTTTTGCTATTCA

TTGAAAGCAGTAGTGACTGATTTGTATATA
>MinSyn_1261|Strength:0.014567139
GCGTGTCGTTTTAGTGAGGATCGAAAGGACAGTAGCAAGACCTCTACAAAACTGGTACTTGCAGCCAC
TTGTGTCGGTAGGTCACAACCATTATTGCGCCTCACATGTAGGCTATCAGCTTAGCCTGACGTAAGGG
ATGACGCACAAGGTCACGACCAGTGGTCCCTCCACTCACTGCTAGGAGGGAAAAAGAAGAGGTTTAGC
AAGATTGCGATAAAGGAAAGGACCGATGCTGATCTTGCCTGCCTTGATTCACTATCAGCTCTGCACCT
CACATGTAGGTGGCTCCTACTACTTGTGTACAGGGCTCTATATAAGGTTTTGCTATTCATTGAAAGCA
GTAGTGACTGATTTGTATATA
>MinSyn_1636|Strength:0.014658457
GCGTGTCGTTTTAGTGAGGAACCATTATTGCGGGTACTTGTGTACGGAAAAAGAAGAGGTTTGTCTGC
ACCTCACATCACTATCAGCATTAGGTTGTCTGCACCTCACAGTGGTCCCTCCACACTATGCCCAATTA
GGTTGTCTCAGCCACTTGTGTGTAGGCTAATGAGCTAAGCACATACGTCAGACCTCACATGTAGGCTA
TCAGCTTATCGAAAGGACAGTAATGTAGGCTATCAGCTTAGCTCTCTCTGCCGACAGTGGTCCCAAAG
GTAGGTCACGACCACTATGCAAATATTTCTTGTGCGGTAGGTCACGACCACCTATATAAGGTTTTGCT
ATTCATTGAAAGCAGTAGTGACTGATTTGTATATA
>MinSyn_1853|Strength:0.014973143
GCGTGTCGTTTTAGTGAGGGTGGGAGCCACCAGGAGGACCGATGCTGATCTTTGAAGATAAGATAATA
ATGTTGAAGATAAGACCGATGCTGATCTTGCCTGCCTTGTCCATCAACAAATAATCCAAGTAAGATCT
TGCCTATGACGTAAGCCATGACGTCTATCTACAAAACTGGTACTTGGAAAAAGAAGAGGTCCAATTAG
GTTGTCTGCACCTGGTGGAGCACGACAGCGGTAGGTCTCTCTCTGCCGACAGTGGTCCCAAATGTAGG
CTATCAGCTTAGCAAGACCTAATTTCGGGAAACCTCCTCGCGACCACTCTATATAAGGTTTTGCTATT
CATTGAAAGCAGTAGTGACTGATTTGTATATA
>MinSyn_1955|Strength:0.015018225
GCGTGTCGTTTTAGTGAGGTCCATCAACAAATAATCCAAGTAAGGCTTAGCAAGACCTCTACAAAACT
GGTACTTGAAGATAAGATAATAATGTTGAAGATAAGAGACCACTATGCCCAATTAGGTTGTCCTGACG
TAAGGGATGACGCACAGCAAGACCTCTACAAAACTGGTACTTGTTCAGAAGATCAAAGGGCTACGACC
ACTATGCCCAATTAGGTTGTTGAAGCATCTTCCCTAGGAGGACCGATGCTGATCTTGCCAGCAAGTGG
ATTAGGCTATTGCGATAAAGGAAAGGCTTAGCAAGACCTATATAAGGTTTTGCTATTCATTGAAAGCA
GTAGTGACTGATTTGTATATA
>MinSyn_1754|Strength:0.015468922
GCGTGTCGTTTTAGTGAGGTGAAGATAAGATAATAATGTTGAAGATAAGAGCCCAATTAGGTTGTCAG
AAGATCAAAGGGCTACGATGCTGATCTTGCCTGCCTTTCACTATCAGCTACAAAACTGGTATGACGTA
AGCCATGACGTCTACTCACGCTTTGTCAAAAGCTAAAAAAGATGATGCCCTCTACAAAACTGGTACTT
GTGTACCAAATATTTCTTGTAGGTTGTCTGCACCTCACATGTAGGTCTCTCTGCCGACAGTGGTCCCA
AAACCACTATGCCCAATTAGGTTGTCTGCACCCTATATAAGGTTTTGCTATTCATTGAAAGCAGTAGT
GACTGATTTGTATATA
>MinSyn_1635|Strength:0.015540591
GCGTGTCGTTTTAGTGAGGATTGCGATAAAGGAAAGGGAGGACCGATGCTGATCTTGCCTGCTGAAGC
ATCTTCCTATGCCCAATTAGGTTGTCTGCAGCTTTGTCAAAAGCTAAAAAAGATGATGCCACTGTGAA
GATAAGATAATAATGTTGAAGATAAGAACTGCTAGGAGGACCGATGCTGATATGACGTAAGCCATGAC
GTCTATATCAAACCATTATTGCGCATGTAGGCTATCAGCTTAGAATTTCGGGAAACCTCCTCGTGCTG
ACAGTGGTCCCTCCACATGTAGGCTATCAGCTTAGCAAGACTTATGACCCCCGCCGATGACGCGGGAA
CCTCACATCTATATAAGGTTTTGCTATTCATTGAAAGCAGTAGTGACTGATTTGTATATA
>MinSyn_1896|Strength:0.015933681
GCGTGTCGTTTTAGTGAGGAAGATTGATGAAAAGTCAAAAACAAAAATCAATTATGGTACTTGTGTAC
AGGGCTCACTGCTATGAGCTAAGCACATACGTCAGTTATCGAAAGGACAGTACTCACATGTAGGCTAT
CTCCATCAACAAATAATCCAAGTAAGCTAGGAGGACCGATGTCTCTCTGCCGACAGTGGTCCCAAACC
ACTATGCAAATATTTCTTGTCTGCTAGGAGGACCGATGCTGATCTTGCCTCTATATAAGGTTTTGCTA
TTCATTGAAAGCAGTAGTGACTGATTTGTATATA
>MinSyn_1665|Strength:0.016143435
GCGTGTCGTTTTAGTGAGGAAGATTGATGAAAAGTCAAAAACAAAAATCAATTATGCTGATCTTTGAA
GCATCTTCCATGCCCAATTAGGTTGTCTCAAATATTTCTTGTTGCCCAATTAGGTTGTCTGCACCTCA
CATGCTTTGTCAAAAGCTAAAAAAGATGATGCGTTGTCTGCACCTCACATGTAGGCTATCAGATGACG
TAAGCCATGACGTCTACAGCTTAGCAAGACCTCTACAAATCAGAAGATCAAAGGGCTATACAGCCACT

TGTGTGACCACTAAAAATGTCAAAGATATGTGAAGATAAGATAATAATGTTGAAGATAAGATACAAAA
CTGGTACTTGTCTATATAAGGTTTTGCTATTCATTGAAAGCAGTAGTGACTGATTTGTATATA
>MinSyn_1730|Strength:0.016314165
GCGTGTCGTTTTAGTGAGGTGAAGCATCTTCCAGCTTAGCAAGAAAAAATGTCAAAGATACAAAACTG
GTATTATGACCCCCGCCGATGACGCGGGAATGTAGGCTATCAGCTCTGACGTAAGGGATGACGCACAT
CAGCTTAGCAGTGGTCCCTCCACCGATGCTGATCTTGCCTAGCAAGTGGATTGCTAGGAGGACCGCAG
CCACTTGTGTATCTTGCCTGCCTTGATGATACAAATATTTCTTGTATGCCCAATTAGGTTGTCTATCG
AAAGGACAGTACACATGCTATATAAGGTTTTGCTATTCATTGAAAGCAGTAGTGACTGATTTGTATAT
A
>MinSyn_1292|Strength:0.016341363
GCGTGTCGTTTTAGTGAGGGCTTTGTCAAAAGCTAAAAAAGATGATGCGTAGGTCACGACCACTATGC
CCAATTAAGCAAGTGGATAGGAGGCTGACGTAAGGGATGACGCACACCTCTACAAAACTGGTACTTGT
GTACAGGGTGAAGCATCTTCCGCGGTAGGTCACGACCACTATGCCCTGGTGGAGCACGACAACAAATA
TTTCTTGTTTCACTATCAGCTGTACAGAAAAATGTCAAAGATACTCACATGTAGGCTATCAGCTTAGC
AACTATATAAGGTTTTGCTATTCATTGAAAGCAGTAGTGACTGATTTGTATATA
>MinSyn_1221|Strength:0.01658346
GCGTGTCGTTTTAGTGAGGAACCATTATTGCGTAGCAAGACCTCTACAAAACTGGAGCAAGTGGATTA
ATGAGCTAAGCACATACGTCAGCGACCACTATGCCCAATCTCTCTGCCGACAGTGGTCCCAAAGCTGT
GAAGCATCTTCCGCGGTAGGTCACGACCACTATGCCCAGCACACCAGCATGTGTTGATCACCAGCTTA
GGAGGAATCGAAAGGACAGTATCACATGTAGGCTATCCTATATAAGGTTTTGCTATTCATTGAAAGCA
GTAGTGACTGATTTGTATATA
>MinSyn_1307|Strength:0.016676953
GCGTGTCGTTTTAGTGAGGATTGCGATAAAGGAAAGGGGAAGCAAGTGGATCAAAACTGGTACTTGTG
TACAGGGTCCATCAACAAATAATCCAAGTAAGACTAATGAGCTAAGCACATACGTCAGACCACTATGC
CCAATTAGAACCACGTCTACAATATCAGCTTAGCAAGACCTCTACAAAGGAAAAAGAAGAGGTTGTCT
GCACCTCACATGTAGGCTATCAATTTCGGGAAACCTCCTCGAAGACCTCTACAAAACTGGTCTATATA
AGGTTTTGCTATTCATTGAAAGCAGTAGTGACTGATTTGTATATA
>MinSyn_1858|Strength:0.016710046
GCGTGTCGTTTTAGTGAGGCAGCCACTTGTGTCTTGTGTACAGGGCTCACTGCTAGGAGGACCTGACG
TAAGGGATGACGCACAGTACAGGGCTCACTGCTAGGAGGACCGAAAAAATGTCAAAGATAAGGACCCA
AATATTTCTTGTGTAGGCTATCAGCTTAGCAAGATTATGACCCCCGCCGATGACGCGGGACGATGCTG
ATCTTGCCTGCTTTGTCAAAAGCTAAAAAAGATGATGCTGTCTGCACTATATAAGGTTTTGCTATTCA
TTGAAAGCAGTAGTGACTGATTTGTATATA
>MinSyn_1408|Strength:0.016719292
GCGTGTCGTTTTAGTGAGGAACCATTATTGCGCACGACCACTATGCCCGGAAAAAGAAGAGGTCGATG
CTGATCTTGCCATGACGTAAGCCATGACGTCTACTTAGCAAAAATGTCAAAGATAACAAAACTCACTA
TCAGCTCACTGCTAGGAGGACCGAAGATTGATGAAAAGTCAAAAACAAAAATCAATTATGTACTTATG
ACCCCCGCCGATGACGCGGGATTGTCTGCTTTTCAACAAGCGGTAGGTCACGACCACTATGCTATATA
AGGTTTTGCTATTCATTGAAAGCAGTAGTGACTGATTTGTATATA
>MinSyn_1988|Strength:0.016891437
GCGTGTCGTTTTAGTGAGGTCCATCAACAAATAATCCAAGTAAGTGATGACGTAAGCCATGACGTCTA
AGGTCACTCTCTCTGCCGACAGTGGTCCCAAATACAAAACTGGTACCAAATATTTCTTGTAGGCTATC
AGCTTAGCAATTGCGATAAAGGAAAGGAAAACTGGTACAGCCACTTGTGTTCACGACCACTATGCCCA
ATTGCACACCAGCATGTGTTGATCACCAGCTATCTTGCTATATAAGGTTTTGCTATTCATTGAAAGCA
GTAGTGACTGATTTGTATATA
>MinSyn_1163|Strength:0.016977389
GCGTGTCGTTTTAGTGAGGATGACGTAAGCCATGACGTCTATTGTCTGCACCTCAGTGGGAGCCACCA
ACGACCACTATGCCCAATTAGGTTGTCTGCAGCAAGTGGATTCTACAAAACTGCAGCCACTTGTGTGT
TCCATCAACAAATAATCCAAGTAAGCACGACCACTATGCCCAATTAGGTTGTGGAAAAAGAAGAGGTT
CGCGGTAGGTCACGACCTATATAAGGTTTTGCTATTCATTGAAAGCAGTAGTGACTGATTTGTATATA
>MinSyn_1565|Strength:0.016986154
GCGTGTCGTTTTAGTGAGGATGACGTAAGCCATGACGTCTACGCGGTAGGTCACGACCACTATGTGGT
GGAGCACGACAAGACCTCTACAAAAACCACGTCTACAATTAGCAAGACCTCTACAAAACTGGTACTTA
ATTTCGGGAAACCTCCTCGTGTAGTGAAGCATCTTCCAGGTTGTCTGATCCTTACCGCTATGGGTAAG

ATTTTAGGTTGTCTGCACTATATAAGGTTTTGCTATTCATTGAAAGCAGTAGTGACTGATTTGTATAT
A
>MinSyn_1521|Strength:0.017043856
GCGTGTCGTTTTAGTGAGGTCTCTCTGCCGACAGTGGTCCCAAAGTAGGTCACGACCACTATGCCCAA
TTAGGATGACGTAAGCCATGACGTCTATGCTAGGAGGACCGATGCTGATCTTCAGTGGTCCCTCCACT
TAGCAAGACCTCTACAAAACTGGTACTGCTTTGTCAAAAGCTAAAAAAGATGATGCCACATGTAGGCT
ATCAGCTTTTATGACCCCCGCCGATGACGCGGGAGCGGTAGGTCACGACCACTATATAAGGTTTTGCT
ATTCATTGAAAGCAGTAGTGACTGATTTGTATATA
>MinSyn_1302|Strength:0.017051622
GCGTGTCGTTTTAGTGAGGTGAAGCATCTTCCACTCCATCAACAAATAATCCAAGTAAGGGGCTCACT
GCATGAGCTAAGCACATACGTCAGTGTCTGCACCTCACATGTAGGCTATCAGCATTGCGATAAAGGAA
AGGCCACTATGCCCAATTAGGTCAGTGGTCCCTCCACTAGGAGGACCGATGGCTTTGTCAAAAGCTAA
AAAAGATGATGCACCTCTACAAAACTGGTACTTCTATATAAGGTTTTGCTATTCATTGAAAGCAGTAG
TGACTGATTTGTATATA
>MinSyn_1783|Strength:0.017141418
GCGTGTCGTTTTAGTGAGGCAGTGGTCCCTCCACTGTCTGCACCTCACAACCATTATTGCGAGACCAT
CGAAAGGACAGTAAGATGAGCTAAGCACATACGTCAGTAGGCTATCAGCTTAGAGGTGGCTCCTACTG
CTAGGAGGACCGATGCTGATCTTGCCTAACCACGTCTACAACTGGTACTTGTGTACAGGGCTCACTGC
TAGGGAAAAAGAAGAGGTAGGTCACGATGAAGCATCTTCCTAGGCTATATAAGGTTTTGCTATTCATT
GAAAGCAGTAGTGACTGATTTGTATATA
>MinSyn_1428|Strength:0.017308183
GCGTGTCGTTTTAGTGAGGCAGCCACTTGTGTACAAAACTGGTACTTGTGTACAGGGCTGGAAAAAGA
AGAGGTGTAGGCTATCCAGTGGTCCCTCCACGCTAGGAGGACCGATGCTGATCTTGCCTGCTGACGTA
AGGGATGACGCACATGCCTGCAACCATTATTGCGGTTGTCTGCACCTCACATGTAGGCAAAAATGTCA
AAGATAGCTAAATTTCGGGAAACCTCCTCGATGCTTCCATCAACAAATAATCCAAGTAAGTCTACAAA
ACTGGTACTTGTGTACAGGGCTCTATATAAGGTTTTGCTATTCATTGAAAGCAGTAGTGACTGATTTG
TATATA
>MinSyn_1037|Strength:0.017312637
GCGTGTCGTTTTAGTGAGGCAAATATTTCTTGTAGGCTATCAGCATTGCGATAAAGGAAAGGTTGGTG
GAGCACGACATGCACCCAGCCACTTGTGTACATGTAGGCTATCAGCTTAGCAAGACCTCAACCATTAT
TGCGCTCACATGTAGGCTATATGACGTAAGCCATGACGTCTAATTAGGTTGTCTGCACCAGGTGGCTC
CTACACCGATGCTGATCTTGCACACCAGCATGTGTTGATCACCAGCTGGTTGTCTGCACCTTTATGAC
CCCCGCCGATGACGCGGGATACTTGTGTACAGGGCTCACTGCTATATAAGGTTTTGCTATTCATTGAA
AGCAGTAGTGACTGATTTGTATATA
>MinSyn_1147|Strength:0.01733475
GCGTGTCGTTTTAGTGAGGAGGTGGCTCCTACGTAGGCTATCAGCTTAGCAAGACCTCTAGCTTTGTC
AAAAGCTAAAAAAGATGATGCTGTAGGCTATCAGCTATGAGCTAAGCACATACGTCAGAGGTTGTCTG
CACCTCACATCCATCAACAAATAATCCAAGTAAGATGCCCAATTAGGTTGTCTGCAAACCATTATTGC
GGACCACTATGCCCAATTTGGTGGAGCACGACAGCCCAATTAGGTTGTCTGCACCTCCTATATAAGGT
TTTGCTATTCATTGAAAGCAGTAGTGACTGATTTGTATATA
>MinSyn_1517|Strength:0.017570124
GCGTGTCGTTTTAGTGAGGAACCATTATTGCGGAGGACCGATGCTGATCTTGCCTGCCTTGAACCACG
TCTACAAAGCTTTGTCAAAAGCTAAAAAAGATGATGCCCGATGCTGATCTTGCCTTTTTCAACAAAGC
AAGACCTCTACAAAACTGGTACTTCTGACGTAAGGGATGACGCACAGACCACTATGCCCAATTAGGTT
CAAATATTTCTTGTATTAGGGAAAAAGAAGAGGTCTAGGAGGACCGATGCTGATCTATCCTTACCGCT
ATGGGTAAGATTGGAGGACCGATGCTGATCTTGCCTGCCTCTATATAAGGTTTTGCTATTCATTGAAA
GCAGTAGTGACTGATTTGTATATA
>MinSyn_1663|Strength:0.017581133
GCGTGTCGTTTTAGTGAGGTCAGAAGATCAAAGGGCTATCTACAAAACTGGTACGCACACCAGCATGT
GTTGATCACCAGCTCACATGTAGGCTACAGCCACTTGTGTATTATGACCCCCGCCGATGACGCGGGAT
AGGTTGTCTGCACCTCAGTGGTCCCTCCACGTGTACAGCTGACGTAAGGGATGACGCACATCGCGGTA
GGTCACGGCTTTGTCAAAAGCTAAAAAAGATGATGCTCTACAAAACTGGTACTTGTGTAAAAATGTCA
AAGATATCTTGCAACCATTATTGCGACCTCACATGTAGGCTATCAGCTTACTATATAAGGTTTTGCTA
TTCATTGAAAGCAGTAGTGACTGATTTGTATATA

>MinSyn_1943|Strength:0.017657967
GCGTGTCGTTTTAGTGAGGTCTCTCTGCCGACAGTGGTCCCAAAACAGGGCTCACTGTGGTGGAGCAC
GACATATCCTTACCGCTATGGGTAAGATTGTACAATGAGCTAAGCACATACGTCAGCGCGGTAGGTCA
CGACCGTGGGAGCCACCAAGGACCGATGCTGATCTTGCCTGCAGTGGTCCCTCCACGTGTATCACTAT
CAGCTAGGTCACGACCATTTTCAACAACTGCCTTGATGAAACCATTATTGCGTGCCCTATATAAGGTT
TTGCTATTCATTGAAAGCAGTAGTGACTGATTTGTATATA
>MinSyn_1744|Strength:0.017707944
GCGTGTCGTTTTAGTGAGGTTTTCAACAACTTAGCAAGACCTCTACAAAACTGGTACTTGGAAAAAGA
AGAGGTCGACCACTATGCCCAATAAAAATGTCAAAGATACTATGACGTAAGCCATGACGTCTACTCAC
TGCTAGGAGCAGTGGTCCCTCCACTCACATCAAATATTTCTTGTAACTCTCTCTGCCGACAGTGGTCC
CAAAAGGAGGACCGATGCTGATCTTGCCTGCCTCAGAAGATCAAAGGGCTAGGTAAGCAAGTGGATGT
AGGTCACTATATAAGGTTTTGCTATTCATTGAAAGCAGTAGTGACTGATTTGTATATA
>MinSyn_1912|Strength:0.017786435
GCGTGTCGTTTTAGTGAGGTCCATCAACAAATAATCCAAGTAAGCTCACATGTAGGCTATAGGTGGCT
CCTACGTAGGTCACGACCACTGGTGGAGCACGACACCTCTACGTGGGAGCCACCACACCTCACATGTA
GGCTGACGTAAGGGATGACGCACAGGCTATCAGCTGCACACCAGCATGTGTTGATCACCAGCTCGCGG
TAGGTCACGACCACTATGGGAAAAAGAAGAGGTGGCTCACTGCTAGGAGAAAAATGTCAAAGATATGT
GTACAGGGCTCACTGCTAGGAGGACCTATATAAGGTTTTGCTATTCATTGAAAGCAGTAGTGACTGAT
TTGTATATA
>MinSyn_1059|Strength:0.017803154
GCGTGTCGTTTTAGTGAGGGTGGGAGCCACCAAGGACCGATGCTGATCTGACGTAAGGGATGACGCAC
ATCTACAAAACTGGTACTTGTGTACTTATGACCCCCGCCGATGACGCGGGATTGCCTGCCTTTGAAGC
ATCTTCCTAGGTCACCAGTGGTCCCTCCACTGATATTTTCAACAATTGTGTACAGGGCTCATCCTTAC
CGCTATGGGTAAGATTAAACTGGTACTTGTGTCTATATAAGGTTTTGCTATTCATTGAAAGCAGTAGT
GACTGATTTGTATATA
>MinSyn_1758|Strength:0.018013979
GCGTGTCGTTTTAGTGAGGAAGATTGATGAAAAGTCAAAAACAAAAATCAATTATCTCCATCAACAAA
TAATCCAAGTAAGACATGTAGGCTATCAGCTTAGCAAAAAAATGTCAAAGATAGGGCTCACTGCTAGG
AGGTGAAGCATCTTCCGACCTCTACAAAACAACCATTATTGCGAGGTTGTCTGCACCCTGACGTAAGG
GATGACGCACATATGCCCATGAAGATAAGATAATAATGTTGAAGATAAGACGGTAGGTCACGACCACT
ATGCCCGCTTTGTCAAAAGCTAAAAAAGATGATGCACAGGGCTCACTGCTAGGAGGACCCTATATAAG
GTTTTGCTATTCATTGAAAGCAGTAGTGACTGATTTGTATATA
>MinSyn_1790|Strength:0.018026655
GCGTGTCGTTTTAGTGAGGATTGCGATAAAGGAAAGGAAGACCTCTACAAAACTGGTACTTGTGTAAT
GAGCTAAGCACATACGTCAGTTGTCTGCACCTCAAGATTGATGAAAAGTCAAAAACAAAAATCAATTA
TCTGATCTTGCCTGCAAAAATGTCAAAGATATCGCGGTAGGTCACGACCTCCATCAACAAATAATCCA
AGTAAGTAGGTTGTCTGCACCTCACATGCTATATAAGGTTTTGCTATTCATTGAAAGCAGTAGTGACT
GATTTGTATATA
>MinSyn_1515|Strength:0.018079662
GCGTGTCGTTTTAGTGAGGATGAGCTAAGCACATACGTCAGCTATGCCCAATTAGGTTGTCTGAAAAA
TGTCAAAGATAGTACTCACTATCAGCGTAGGCTATCAGCTTAGCAAGAGGTGGCTCCTACATCTTGCC
TGCCTTCAAATATTTCTTGTTGCTGATCAACCATTATTGCGACCTCACATGTAGGCTATCAGCTATAT
AAGGTTTTGCTATTCATTGAAAGCAGTAGTGACTGATTTGTATATA
>MinSyn_1617|Strength:0.018431341
GCGTGTCGTTTTAGTGAGGTGGTGGAGCACGACAAACTGGTACTTGTGTACAGGGCTCATGAGCTAAG
CACATACGTCAGTCAATGACGTAAGCCATGACGTCTACGCGGTAGGTCTCTCTCTGCCGACAGTGGTC
CCAAACTTAGCAAGACCTCTAAACCACGTCTACAATTGTGTAAGGTGGCTCCTACCGCGGTAGGGGAA
AAAGAAGAGGTCAATTAGGTTGTCTGCACCTCACATGTAGGTTTTCAACAATGGTACTTGTGTACAGG
GCTCACTGCTAGGTGGGAGCCACCAGTCTATATAAGGTTTTGCTATTCATTGAAAGCAGTAGTGACTG
ATTTGTATATA
>MinSyn_1058|Strength:0.018480717
GCGTGTCGTTTTAGTGAGGTGGTGGAGCACGACATAGGTTGAGCAAGTGGATGGACCGATGCTGATCT
TGCCTGCCTTGATTCACTATCAGCCTGATCTTGCCTGCCTTGAATGACGTAAGCCATGACGTCTATAG
GCTATCAGCTTAGCAAGACCTCTAAGGTGGCTCCTACACCACTATGCCCAATTAGGTTGTCTGCATCG

AAAGGACAGTAAGCAAGACCTCTACAAAACTGTGAAGATAAGATAATAATGTTGAAGATAAGAAGACC
TCCTATATAAGGTTTTGCTATTCATTGAAAGCAGTAGTGACTGATTTGTATATA
>MinSyn_1398|Strength:0.018508868
GCGTGTCGTTTTAGTGAGGTCACTATCAGCGATCCTTACCGCTATGGGTAAGATTTAATCGAAAGGAC
AGTACTTGTGTACAGGGCATGAGCTAAGCACATACGTCAGACTTGTTCTCTCTGCCGACAGTGGTCCC
AAAGTTGTCTGCACCTCACATGTAGGCCAGCCACTTGTGTACCTCACATGTAGGCTATTGAAGATAAG
ATAATAATGTTGAAGATAAGACTTAGCAAGACCTCTACAACTATATAAGGTTTTGCTATTCATTGAAA
GCAGTAGTGACTGATTTGTATATA
>MinSyn_1403|Strength:0.018577837
GCGTGTCGTTTTAGTGAGGAAAAATGTCAAAGATATACAAAACTGGTACTTGTGTACAGGGCTGACGT
AAGGGATGACGCACACTATCAGCTTAGCAATGACGTAAGCCATGACGTCTAAATTAGGTTGTCATCCT
TACCGCTATGGGTAAGATTCCTCTACAAAACAGGTGGCTCCTACGTACTTGTGTACAGGGCTCACTGC
TAGGAGGTGGGAGCCACCATCTGCACCTCACACAAATATTTCTTGTAAGACCTCTACAAAACTGGTAA
GATTGATGAAAAGTCAAAAACAAAAATCAATTATAGGCTATTCACTATCAGCCTCACTGCTAGGAGGA
CCGATGCTGATCCTATATAAGGTTTTGCTATTCATTGAAAGCAGTAGTGACTGATTTGTATATA
>MinSyn_1017|Strength:0.018651377
GCGTGTCGTTTTAGTGAGGAGGTGGCTCCTACGTAGGTCACGACCACTATGCCAGCCACTTGTGTAGG
ACCGAGCACACCAGCATGTGTTGATCACCAGCTCCACTATGCCCAATTAGCTGACGTAAGGGATGACG
CACACTTGCCTGCCTTTGGTGGAGCACGACAGCAGTGGTCCCTCCACCAAGACCTCTACAAAACTGGT
ACTTGTGTTGAAGATAAGATAATAATGTTGAAGATAAGAATGCTGATCTTGCCTGCCTTGAGCAAGTG
GATAGGCTATATAAGGTTTTGCTATTCATTGAAAGCAGTAGTGACTGATTTGTATATA
>MinSyn_1391|Strength:0.019058547
GCGTGTCGTTTTAGTGAGGCTGACGTAAGGGATGACGCACACGCTTATGACCCCCGCCGATGACGCGG
GAGCACCTCACATGTAGGCTATCAGCTTAGCAATTTCGGGAAACCTCCTCGTCAGCTTAGCAAGACCT
CTACAAAACTGGTAAGATTGATGAAAAGTCAAAAACAAAAATCAATTATCCGATGCTGTCACTATCAG
CATGCTGCTATATAAGGTTTTGCTATTCATTGAAAGCAGTAGTGACTGATTTGTATATA
>MinSyn_1835|Strength:0.019176719
GCGTGTCGTTTTAGTGAGGTCAGAAGATCAAAGGGCTACGCGGTAGGTCACGACCACTATGCCCAATT
ATCCTTACCGCTATGGGTAAGATTTGTAGGCTATCAGCTTAGCAAGACCTCTAAATTTCGGGAAACCT
CCTCGCTTCACTATCAGCTTGATGATGACGTAAGCCATGACGTCTACCAATTGTGGGAGCCACCAACT
ATGCCCAATTAGGTTGTCTGCACCTAACCACGTCTACAAGCTTAGCAAGACCTCTACAAAACTGCAGC
CACTTGTGTGGTAGGTCACGACCACTATGCCTATATAAGGTTTTGCTATTCATTGAAAGCAGTAGTGA
CTGATTTGTATATA
>MinSyn_1034|Strength:0.019263706
GCGTGTCGTTTTAGTGAGGATGAGCTAAGCACATACGTCAGTAGGTATTGCGATAAAGGAAAGGCAAA
ACTGGTACTAAGATTGATGAAAAGTCAAAAACAAAAATCAATTATCGATGCTGATCTTGCCTGTCCAT
CAACAAATAATCCAAGTAAGACGAATTTCGGGAAACCTCCTCGGCCCAATTAGGTTGTCCTATATAAG
GTTTTGCTATTCATTGAAAGCAGTAGTGACTGATTTGTATATA
>MinSyn_1603|Strength:0.019577595
GCGTGTCGTTTTAGTGAGGGCACACCAGCATGTGTTGATCACCAGCTTACAGGGCTTGGTGGAGCACG
ACACCCAATTAGGTATGACGTAAGCCATGACGTCTATATGCCCAATTAGGTTGTCTTCCATCAACAAA
TAATCCAAGTAAGTCGCTTTGTCAAAAGCTAAAAAAGATGATGCTCACTGCTAGGAGGACCGATGCTG
ATCTTGGAAAAAGAAGAGGTACGACCACTATGCCCAATTAGGTCTATATAAGGTTTTGCTATTCATTG
AAAGCAGTAGTGACTGATTTGTATATA
>MinSyn_1608|Strength:0.019584039
GCGTGTCGTTTTAGTGAGGTGGTGGAGCACGACATTGTGTATTATGACCCCCGCCGATGACGCGGGAG
CTAGGAGGAATGACGTAAGCCATGACGTCTATATCAGCTTAGCAAGACCTCTACAAAATCCATCAACA
AATAATCCAAGTAAGGGCTATCAGCTTAGCCAGCCACTTGTGTTGTCTGCAATTGCGATAAAGGAAAG
GTGCACCTCACATGTAGGCTATCATGAAGCATCTTCCCTGGTCTATATAAGGTTTTGCTATTCATTGA
AAGCAGTAGTGACTGATTTGTATATA
>MinSyn_1111|Strength:0.019819941
GCGTGTCGTTTTAGTGAGGATCGAAAGGACAGTACCAATTAGGTTGTCTGCACCTCACATGTAGAAGA
TTGATGAAAAGTCAAAAACAAAAATCAATTATACTTGTGTACAGGGCTCACTGCTAGGAGGTCAGAAG
ATCAAAGGGCTACACGACCACTATGCCCAATTAGGTTGTCCAGTGGTCCCTCCACCTGCTAGGAGCTG

ACGTAAGGGATGACGCACATACTTGTGTACAGGGCTCACTGCTAGTGGGAGCCACCATCACATGTAGG
CACACCAGCATGTGTTGATCACCAGCTAGGCTATCAGCTTAGCAAGACCTCTACTATATAAGGTTTTG
CTATTCATTGAAAGCAGTAGTGACTGATTTGTATATA
>MinSyn_1802|Strength:0.020251865
GCGTGTCGTTTTAGTGAGGAATTTCGGGAAACCTCCTCGTTTATGACCCCCGCCGATGACGCGGGAGG
TTGTCTGCACCTCACATGTAGCTGACGTAAGGGATGACGCACATACAAAACTGGTACTGGTGGAGCAC
GACAACAGGTGGCTCCTACAGGAGGACCGATGCTGAAAGATTGATGAAAAGTCAAAAACAAAAATCAA
TTATTGACAGCCACTTGTGTAAGACCTCTACAAAACTGGTACTTGTGCTATATAAGGTTTTGCTATTC
ATTGAAAGCAGTAGTGACTGATTTGTATATA
>MinSyn_1917|Strength:0.020273035
GCGTGTCGTTTTAGTGAGGAAGATTGATGAAAAGTCAAAAACAAAAATCAATTATGGTTCACTATCAG
CGTACTTGTGTACAGGGCTCACTAATTTCGGGAAACCTCCTCGGTACTTCAGAAGATCAAAGGGCTAC
ATGTAGGCCTGACGTAAGGGATGACGCACACAAAACTGGTACTTGTGTACAGGGCTATTGCGATAAAG
GAAAGGAGCTTAGCAAGACCTCTACAAAAGCTTTGTCAAAAGCTAAAAAAGATGATGCGCGGTAGAGC
AAGTGGATACCCTATATAAGGTTTTGCTATTCATTGAAAGCAGTAGTGACTGATTTGTATATA
>MinSyn_1274|Strength:0.02048127
GCGTGTCGTTTTAGTGAGGATTGCGATAAAGGAAAGGTCTTGCCTGCCTTGATGACTGACGTAAGGGA
TGACGCACACTACAAAACTGATGAGCTAAGCACATACGTCAGCTGCACCTCACATTCTCTCTGCCGAC
AGTGGTCCCAAATGTCTGCACCTCACATGTACAGTGGTCCCTCCACGCTTAGCAAGACCTCTACTGGT
GGAGCACGACAAAACTGGTACTTGTGAATTTCGGGAAACCTCCTCGCACTACAAATATTTCTTGTAGC
AAGACCTCTACGGAAAAAGAAGAGGTAATTAGGTTGTCCTATATAAGGTTTTGCTATTCATTGAAAGC
AGTAGTGACTGATTTGTATATA
>MinSyn_1786|Strength:0.020632702
GCGTGTCGTTTTAGTGAGGTGAAGATAAGATAATAATGTTGAAGATAAGACACATGTAGAAAAATGTC
AAAGATAGCAAGACCTCGCTTTGTCAAAAGCTAAAAAAGATGATGCACCTCTCCATCAACAAATAATC
CAAGTAAGCTCTACAAAACTGGTACTTGTGTACAAACCATTATTGCGAGCTTAGCAAGACCTCTACAA
AACTGGTATGACGTAAGCCATGACGTCTAGCAAATATTTCTTGTCTGGTACAGTGGTCCCTCCACCAA
GACCTCTGCACACCAGCATGTGTTGATCACCAGCTCCACTATGCCCAATTAGGTTCTATATAAGGTTT
TGCTATTCATTGAAAGCAGTAGTGACTGATTTGTATATA
>MinSyn_1764|Strength:0.020639637
GCGTGTCGTTTTAGTGAGGTGGTGGAGCACGACAGGGCTCACTGCTAGGAGGACATGACGTAAGCCAT
GACGTCTATATGCCCAATTAGGTTGTCTGCAACCATTATTGCGTGTGTACAGGGTGGGAGCCACCAAT
TAGGTTGTCTGCACCTTTTTCAACAAGCAAGACCTCTACAAAACTGGTACTTGTGAATTTCGGGAAAC
CTCCTCGTATGCCCAATTAGCTATATAAGGTTTTGCTATTCATTGAAAGCAGTAGTGACTGATTTGTA
TATA
>MinSyn_1604|Strength:0.020644542
GCGTGTCGTTTTAGTGAGGAAAAATGTCAAAGATACCCAATTAGGTTGTCTGCACCTCACATAGGTGG
CTCCTACTAGGTCACGACCACTATGCCCAATTAGGTAACCACGTCTACAATGATCTTGCCTGCCTTGA
TGAATGACGTAAGCCATGACGTCTAGTAGGCTATCAGCTTAGCTGGTGGAGCACGACAACCGATGCTG
TGGGAGCCACCACTATCAGCTTAGCAAGACCTCTACAAAACGCTTTGTCAAAAGCTAAAAAAGATGAT
GCTATCTATATAAGGTTTTGCTATTCATTGAAAGCAGTAGTGACTGATTTGTATATA
>MinSyn_1579|Strength:0.020700241
GCGTGTCGTTTTAGTGAGGTGAAGATAAGATAATAATGTTGAAGATAAGAACAAAACTGGTACTTGTG
TACAGGGCTCACCAGTGGTCCCTCCACGTTGTCTGCACCTCACATGTAGGCTATATCGAAAGGACAGT
AAGGTCACGACAAATATTTCTTGTGCTCACTGCTAGGAGGACCGATGCATGACGTAAGCCATGACGTC
TAGATGCTGATCTTGCCTGTCAGAAGATCAAAGGGCTAGGTCACGACCACTATGCCCAATGAAGCATC
TTCCCCTATCCTTACCGCTATGGGTAAGATTCTCACACTATATAAGGTTTTGCTATTCATTGAAAGCA
GTAGTGACTGATTTGTATATA
>MinSyn_1158|Strength:0.020721356
GCGTGTCGTTTTAGTGAGGATGACGTAAGCCATGACGTCTAAAGACCTCTACAGCTTTGTCAAAAGCT
AAAAAAGATGATGCTGCCCAATTGAAGATAAGATAATAATGTTGAAGATAAGAGACCACTATGCCCAA
TTAGGTTGTAATTTCGGGAAACCTCCTCGATTAGGTTTGAAGCATCTTCCGATCTTGCCTGCCTTGCT
ATATAAGGTTTTGCTATTCATTGAAAGCAGTAGTGACTGATTTGTATATA
>MinSyn_1942|Strength:0.020967183

GCGTGTCGTTTTAGTGAGGATTGCGATAAAGGAAAGGTGCCTGCCAGGTGGCTCCTACCTGCACCTCA
CATGTAGGCTATCAGCTTAATCGAAAGGACAGTATCACTATGACGTAAGCCATGACGTCTAACTGGTA
CTTGTGTACATGAAGATAAGATAATAATGTTGAAGATAAGAGTCACTATCAGCTAGGAGGACAGCCAC
TTGTGTCTGCTAGGAGGACCGACAAATATTTCTTGTTGTCTGCACCTCACATGTCTATATAAGGTTTT
GCTATTCATTGAAAGCAGTAGTGACTGATTTGTATATA
>MinSyn_1460|Strength:0.020970431
GCGTGTCGTTTTAGTGAGGGCTTTGTCAAAAGCTAAAAAAGATGATGCGACCTCTATCCTTACCGCTA
TGGGTAAGATTGAGGACCGATGCTGAAATTTCGGGAAACCTCCTCGACAAAACTTCAGAAGATCAAAG
GGCTACGGTAGGTCACGACCACTATGCCCAATATCGAAAGGACAGTATGATCTTGCCTGACGTAAGGG
ATGACGCACAGTAGGCTATCAGCTTAGCAAGACCTAACCATTATTGCGATCTTGCCTGCCTCAGCCAC
TTGTGTTACAGGCAAATATTTCTTGTGCTATCAGCTTAGCCTATATAAGGTTTTGCTATTCATTGAAA
GCAGTAGTGACTGATTTGTATATA
>MinSyn_1925|Strength:0.020998491
GCGTGTCGTTTTAGTGAGGTCAGAAGATCAAAGGGCTAACTGGTACTTGTGTACAGGGCTCACGGAAA
AAGAAGAGGTACGACCACTATGCCCAATTAGGTAATTTCGGGAAACCTCCTCGTAAAAAATGTCAAAG
ATAGTCACGACCACTATGCCCAGTGGTCCCTCCACGGTTGGTGGAGCACGACATCTTGCCTGCCTTGA
TGATAATCGAAAGGACAGTATACAGGGCTCACTCTGACGTAAGGGATGACGCACATCACATGTAGGCT
ATCAGCTTAGGCTTTGTCAAAAGCTAAAAAAGATGATGCTCACTGCTAGGAGGACCGATGCTGATCCT
ATATAAGGTTTTGCTATTCATTGAAAGCAGTAGTGACTGATTTGTATATA
>MinSyn_1724|Strength:0.021043932
GCGTGTCGTTTTAGTGAGGTGAAGCATCTTCCTTAGCAAGAATCGAAAGGACAGTAATTAGGTTAGGT
GGCTCCTACTGCACCTCACATGTAGGCAATTTCGGGAAACCTCCTCGACTGGTTCACTATCAGCTCAG
CCTGACGTAAGGGATGACGCACAACTTGTGTACAGGTGGGAGCCACCAATGCCCAATTAGGTTGTCTG
CAAACCATTATTGCGCACCTCACATGTAGGCATTGCGATAAAGGAAAGGACTGCTAGGAGGACCGATG
CTGACTATATAAGGTTTTGCTATTCATTGAAAGCAGTAGTGACTGATTTGTATATA
>MinSyn_1894|Strength:0.021058824
GCGTGTCGTTTTAGTGAGGCTGACGTAAGGGATGACGCACATTGATGATAAGATTGATGAAAAGTCAA
AAACAAAAATCAATTATAAAACTGGTACTTGTGGCACACCAGCATGTGTTGATCACCAGCTGCCCAAT
TTCACTATCAGCCGATGCTGATCTTGCAGCCACTTGTGTCTGCACCTCACATGTAGGCTATCAGCCTA
TATAAGGTTTTGCTATTCATTGAAAGCAGTAGTGACTGATTTGTATATA
>MinSyn_1927|Strength:0.021595007
GCGTGTCGTTTTAGTGAGGCAAATATTTCTTGTCTCACTGCTAGGAGGACCGATGCTGATGTGGGAGC
CACCACCACTATGCCCAATTAGGTTGTCTGCAGCAAGTGGATATGTAGGCTATCAGCTTAGCATGGTG
GAGCACGACATAGGCAGTGGTCCCTCCACTCGCGGTATGACGTAAGCCATGACGTCTATATCAGCTTA
AAAAATGTCAAAGATACATGTAGGCTATCAGCTTTCAGAAGATCAAAGGGCTAGTCTGCACCTCACAT
GTAGTGAAGCATCTTCCGACCGACTATATAAGGTTTTGCTATTCATTGAAAGCAGTAGTGACTGATTT
GTATATA
>MinSyn_1204|Strength:0.021651386
GCGTGTCGTTTTAGTGAGGTGAAGATAAGATAATAATGTTGAAGATAAGATGATCTTGCTGAAGCATC
TTCCGCACCTCACATGTAGGCTATCAGCTTAGCCAGCCACTTGTGTACTGCTAGGAGGACCGATGCTG
ATCAACCACGTCTACAACCCTGACGTAAGGGATGACGCACATCTGCACCTCACATGTAGAAGATTGAT
GAAAAGTCAAAAACAAAAATCAATTATGTCTGCACCTCAATTGCGATAAAGGAAAGGTGCTAGGAGGA
GGTGGCTCCTACCTCTATATAAGGTTTTGCTATTCATTGAAAGCAGTAGTGACTGATTTGTATATA
>MinSyn_1344|Strength:0.02172796
GCGTGTCGTTTTAGTGAGGTGGTGGAGCACGACACTTAGCAAGACCTCTACAAACTGACGTAAGGGAT
GACGCACAGTTATCGAAAGGACAGTACAATTAGTTTTCAACAAATAAGATTGATGAAAAGTCAAAAAC
AAAAATCAATTATGGTAGGTCATCAGAAGATCAAAGGGCTATAAGGTGGCTCCTACCTGCACCTCACA
TGTAGGCTATCAGCTTACTATATAAGGTTTTGCTATTCATTGAAAGCAGTAGTGACTGATTTGTATAT
A
>MinSyn_1115|Strength:0.02175701
GCGTGTCGTTTTAGTGAGGAACCATTATTGCGGCCTTGAACCACGTCTACAATAGGTATGACGTAAGC
CATGACGTCTACGATGCTGATCTTGCCTGCCTTAGGTGGCTCCTACCAATTAGGTTGTCTGCACCTCA
CATATCCTTACCGCTATGGGTAAGATTTTAGGTTGTCTGCACCTCACTTATGACCCCCGCCGATGACG
CGGGAAGACCTCTACAAACTATATAAGGTTTTGCTATTCATTGAAAGCAGTAGTGACTGATTTGTATA

TA
>MinSyn_1576|Strength:0.022107473
GCGTGTCGTTTTAGTGAGGTCTCTCTGCCGACAGTGGTCCCAAAGGTAGGTCTCCATCAACAAATAAT
CCAAGTAAGGTAGGCTATCAGCTTAGCATGACGTAAGCCATGACGTCTATCGCGGTAGGTCACATTGC
GATAAAGGAAAGGCACTGCTAGAGCAAGTGGATGTTGTCTGCACCTCACATGTAGGCTATCGCACACC
AGCATGTGTTGATCACCAGCTATCAGCTTAGCAAGACCTCTATATAAGGTTTTGCTATTCATTGAAAG
CAGTAGTGACTGATTTGTATATA
>MinSyn_1347|Strength:0.022211928
GCGTGTCGTTTTAGTGAGGCTGACGTAAGGGATGACGCACAGGGCTCACTGCGTGGGAGCCACCAGAT
GCTGATCTTAAAAATGTCAAAGATATACTTGTGTACAGTCACTATCAGCTTGTCTGCACCTCACATGT
AGGCTACAGCCACTTGTGTGCACAGCAAGTGGATGAACCATTATTGCGCGGTAGGTCACCTATATAAG
GTTTTGCTATTCATTGAAAGCAGTAGTGACTGATTTGTATATA
>MinSyn_1383|Strength:0.022337491
GCGTGTCGTTTTAGTGAGGGCTTTGTCAAAAGCTAAAAAAGATGATGCCGCGGTAAACCACGTCTACA
AGAATCGAAAGGACAGTAGTGGTGGAGCACGACACACCTCACATGTAGGCTATCAGCTTAGCAACTGA
CGTAAGGGATGACGCACAGTACTTGTGTACAGATTGCGATAAAGGAAAGGCACTATGCCCAATTAGGT
TGTCTGCACCTGCACACCAGCATGTGTTGATCACCAGCTAGGACCGATGCTGATCTTGCCTCTATATA
AGGTTTTGCTATTCATTGAAAGCAGTAGTGACTGATTTGTATATA
>MinSyn_1148|Strength:0.022351469
GCGTGTCGTTTTAGTGAGGATGACGTAAGCCATGACGTCTACACCTCACATGTAGGCTATCAGCTTAG
ATTGCGATAAAGGAAAGGCCGATGCTGATCTTGCCTGCATCGAAAGGACAGTAGCACCTCACATGTAG
GCTATCAAATTTCGGGAAACCTCCTCGAGGACCGATGCTGGTGGAGCACGACAGTACAGCTATATAAG
GTTTTGCTATTCATTGAAAGCAGTAGTGACTGATTTGTATATA
>MinSyn_1233|Strength:0.022693784
GCGTGTCGTTTTAGTGAGGATGACGTAAGCCATGACGTCTAAGCAAGACCTCTACAAAACTGGAAAAA
GAAGAGGTCTGCAATCGAAAGGACAGTATCGCGAGGTGGCTCCTACGGTAGGTGAAGATAAGATAATA
ATGTTGAAGATAAGAGACCTCTACAAAACTGATTGCGATAAAGGAAAGGGCACCTCACACTATATAAG
GTTTTGCTATTCATTGAAAGCAGTAGTGACTGATTTGTATATA
>MinSyn_1430|Strength:0.022780331
GCGTGTCGTTTTAGTGAGGATGACGTAAGCCATGACGTCTACGCGGTAGGTGGGAGCCACCAAGCAAG
AGGAAAAAGAAGAGGTTGGTACTTGTGTACAGGGCTAGCAAGTGGATGAGGTGGCTCCTACACAAAAC
TGGTACTTGTGTACAGGTGAAGATAAGATAATAATGTTGAAGATAAGACCCAATTAGCTATATAAGGT
TTTGCTATTCATTGAAAGCAGTAGTGACTGATTTGTATATA
>MinSyn_1900|Strength:0.023125814
GCGTGTCGTTTTAGTGAGGAACCATTATTGCGTGTACAGGGCTCACTGCTAGGAGGACCTCACTATCA
GCACCTCACATGTAGGCTATCAGCTTAGATCGAAAGGACAGTAACTGACGTAAGGGATGACGCACAGT
TGTCTGCAAATATTTCTTGTATTAGGTTGTCTGCACCTCATCAGAAGATCAAAGGGCTAACCTCACAT
GTAGGCTATCAGCTTAGCAAATTTCGGGAAACCTCCTCGTGTCTGCTATATAAGGTTTTGCTATTCAT
TGAAAGCAGTAGTGACTGATTTGTATATA
>MinSyn_1124|Strength:0.023167549
GCGTGTCGTTTTAGTGAGGATGAGCTAAGCACATACGTCAGTGTGTACAGGGCTCACTGCTAGCAAAT
ATTTCTTGTTCGCGGTATGACGTAAGCCATGACGTCTATAGGATTATGACCCCCGCCGATGACGCGGG
AACTGCTAGGAGGACCGATGCTGATCACTATCAGCGCGGTAGGTCACTGGTGGAGCACGACATTGCCT
GATCGAAAGGACAGTAGACCTCTACAAAACTGGTACTTGTGTACAGGGAAAAAGAAGAGGTGTACAGG
GCTCACTGCTATCCTTACCGCTATGGGTAAGATTTAGGAGGACCGCTATATAAGGTTTTGCTATTCAT
TGAAAGCAGTAGTGACTGATTTGTATATA
>MinSyn_1613|Strength:0.02336254
GCGTGTCGTTTTAGTGAGGATGAGCTAAGCACATACGTCAGGTGTACAGGGCTCACTGCTAGGAACCA
CGTCTACAAAGCTTAGCAAGACCTCTACAATTTCGGGAAACCTCCTCGCCGATGGCTTTGTCAAAAGC
TAAAAAAGATGATGCACTGGTACTTGTGTACAGGGCTCACCTATATAAGGTTTTGCTATTCATTGAAA
GCAGTAGTGACTGATTTGTATATA
>MinSyn_1808|Strength:0.023566348
GCGTGTCGTTTTAGTGAGGATGACGTAAGCCATGACGTCTAACTATGCCCAATTAGGGGAAAAAGAAG
AGGTCTCACTGTCCATCAACAAATAATCCAAGTAAGCATGTAGATCGAAAGGACAGTATGGTACTTGT

GTACAGGGCTCACTGCTAGTCACTATCAGCGACCACTATGCCCAATTAGGTTGCTATATAAGGTTTTG
CTATTCATTGAAAGCAGTAGTGACTGATTTGTATATA
>MinSyn_1979|Strength:0.023684046
GCGTGTCGTTTTAGTGAGGTGGTGGAGCACGACATCTACAAAAATGACGTAAGCCATGACGTCTACAC
GACCACTACTGACGTAAGGGATGACGCACAGACCACTATGCAAGATTGATGAAAAGTCAAAAACAAAA
ATCAATTATGGACCGATGCTGATCTTGCCAGTGGTCCCTCCACCTGCACCTCACATGTAGGAAAAATG
TCAAAGATACCAATTAGGTTGTCTGCACCTCACATGTAGAGGTGGCTCCTACCCAATCTATATAAGGT
TTTGCTATTCATTGAAAGCAGTAGTGACTGATTTGTATATA
>MinSyn_1181|Strength:0.023913541
GCGTGTCGTTTTAGTGAGGCAAATATTTCTTGTAACTGGTACTTGTGTACAGGGTGAAGATAAGATAA
TAATGTTGAAGATAAGACAGCTTAGCACTGACGTAAGGGATGACGCACACACGACCACTATGCCCAAT
CAGAAGATCAAAGGGCTAGTTGTCTGCACATTGCGATAAAGGAAAGGCTCACATGTAGGCTTGGTGGA
GCACGACATAGCAATCGAAAGGACAGTAGCTCCTATATAAGGTTTTGCTATTCATTGAAAGCAGTAGT
GACTGATTTGTATATA
>MinSyn_1998|Strength:0.024347373
GCGTGTCGTTTTAGTGAGGAATTTCGGGAAACCTCCTCGACCTCACATGTAGGCTATCAGCTTAGCAA
ACCATTATTGCGAGGGCTCACTGCTAGGAGGACCGATGCTGATCGAAAGGACAGTATGATCTGAAGCA
TCTTCCAGACCTCTACAAAACTGGTACTTGTGTGCACACCAGCATGTGTTGATCACCAGCTGCTAGGA
GGACCGCTGACGTAAGGGATGACGCACATAGGAGGACCGATGTGAAGATAAGATAATAATGTTGAAGA
TAAGAACTATGCCCAATTAGGTTGTCTGCACTATATAAGGTTTTGCTATTCATTGAAAGCAGTAGTGA
CTGATTTGTATATA
>MinSyn_1482|Strength:0.02464178
GCGTGTCGTTTTAGTGAGGGTGGGAGCCACCACCTTATGACCCCCGCCGATGACGCGGGATGATTGAA
GATAAGATAATAATGTTGAAGATAAGACTGCTAGGTGAAGCATCTTCCAGACCTCTACAAAACTGTCC
ATCAACAAATAATCCAAGTAAGCAAAACTGGTACTTGTGTACAGGGTTTTCAACAAGGCTCATGACGT
AAGCCATGACGTCTAAGGGCTCAGTGGTCCCTCCACTTAGCAAGACCTCTACAAAACTGCACACCAGC
ATGTGTTGATCACCAGCTTCACCTATATAAGGTTTTGCTATTCATTGAAAGCAGTAGTGACTGATTTG
TATATA
>MinSyn_1600|Strength:0.024891757
GCGTGTCGTTTTAGTGAGGCAAATATTTCTTGTGGGCATCCTTACCGCTATGGGTAAGATTCACTATG
CCCAATTAGGTTGAAGATAAGATAATAATGTTGAAGATAAGACACTGCTAGGAGGACCGATGCTGATC
TTATGACGTAAGCCATGACGTCTAGTAACCATTATTGCGAGCAATCGAAAGGACAGTATTGCCTGCCT
TGATGAAAGATTGATGAAAAGTCAAAAACAAAAATCAATTATGCTATCAGCTTCTATATAAGGTTTTG
CTATTCATTGAAAGCAGTAGTGACTGATTTGTATATA
>MinSyn_1342|Strength:0.024902135
GCGTGTCGTTTTAGTGAGGCAGTGGTCCCTCCACACGACCACGTGGGAGCCACCACCCAATTAGGTTG
TCTGCACCTCACATTCACTATCAGCTCTACAAAACTGGTACTTGTCTGACGTAAGGGATGACGCACAG
GTCACGACCACAAGATTGATGAAAAGTCAAAAACAAAAATCAATTATCAGGGCTCACTGCTAGGAGGA
CCGATGATCGAAAGGACAGTAACCAAATATTTCTTGTGCTATATAAGGTTTTGCTATTCATTGAAAGC
AGTAGTGACTGATTTGTATATA
>MinSyn_1595|Strength:0.024922556
GCGTGTCGTTTTAGTGAGGAACCACGTCTACAAAATTAGGTTGTCTGCACCTCCAGTGGTCCCTCCAC
ACCACTATGCCCAATTATGACGTAAGCCATGACGTCTAGGTAGGTCACGACCACTATGCCCAATTAGG
GCACACCAGCATGTGTTGATCACCAGCTATAAGATTGATGAAAAGTCAAAAACAAAAATCAATTATAT
CAGCATCGAAAGGACAGTAGGCTATATAAGGTTTTGCTATTCATTGAAAGCAGTAGTGACTGATTTGT
ATATA
>MinSyn_1231|Strength:0.025061356
GCGTGTCGTTTTAGTGAGGAATTTCGGGAAACCTCCTCGTAGGTCACGATCACTATCAGCCCACTATG
CCCAATTAGGTTGTCTGCACCTCCATCAACAAATAATCCAAGTAAGTCTGCACCTCAACCACGTCTAC
AACCACTATTTTCAACAACTTAGCAAGACCTCTACAAAATCGAAAGGACAGTAGCTTAGCAAGACCTC
ATGACGTAAGCCATGACGTCTAGCAAATATTTCTTGTGCTAGGAGGACCGATGCTGATCTTGTCTCTC
TGCCGACAGTGGTCCCAAAGGAGCTATATAAGGTTTTGCTATTCATTGAAAGCAGTAGTGACTGATTT
GTATATA
>MinSyn_1908|Strength:0.025090813

GCGTGTCGTTTTAGTGAGGCAGTGGTCCCTCCACAAACTGGTACTTGTGTACAGGGATGACGTAAGCC
ATGACGTCTAACATGTAGGCTATCAAGCAAGTGGATTAGGTCACGACCACTATGTCCATCAACAAATA
ATCCAAGTAAGACCGATGCTGATCTTGCAAAAATGTCAAAGATAACCACTATGCCCAATTAGGTTGTC
TCTATATAAGGTTTTGCTATTCATTGAAAGCAGTAGTGACTGATTTGTATATA
>MinSyn_1684|Strength:0.025177688
GCGTGTCGTTTTAGTGAGGAAGATTGATGAAAAGTCAAAAACAAAAATCAATTATACTATGCCCAATT
AGGTTGTCTGCACTCCATCAACAAATAATCCAAGTAAGCGGTAGGTCACGACCACTATGCCCAAAAAA
ATGTCAAAGATAATCTTGCCTGCCTTGATGACTGACGTAAGGGATGACGCACATTGTGTACAGGGCTC
ACTGCTAGTGAAGATAAGATAATAATGTTGAAGATAAGAGCTTAGCAAGACCTCTACAAAACTGGTAC
TATATAAGGTTTTGCTATTCATTGAAAGCAGTAGTGACTGATTTGTATATA
>MinSyn_1958|Strength:0.025389954
GCGTGTCGTTTTAGTGAGGTTATGACCCCCGCCGATGACGCGGGAAACTGGTACTTGTGTACAGGGCT
CAGAAGATCAAAGGGCTATACAGGGCTCACTGATCGAAAGGACAGTAGTTGTCTGCACGTGGGAGCCA
CCACTGGTACTTGTGTACAGGGCTCACTTCTCTCTGCCGACAGTGGTCCCAAAGCTCACTGCTAGGAG
GACCGCTTTGTCAAAAGCTAAAAAAGATGATGCCAGCTTAGCAAGACCTCTAATTTCGGGAAACCTCC
TCGTCACTGCTAGGAGGATGAAGATAAGATAATAATGTTGAAGATAAGAGGCTCACTGCTAGGAATGA
CGTAAGCCATGACGTCTAACAAAACTGGTACTTGTGTACAGGGCTCACTATATAAGGTTTTGCTATTC
ATTGAAAGCAGTAGTGACTGATTTGTATATA
>MinSyn_1569|Strength:0.025869568
GCGTGTCGTTTTAGTGAGGGCTTTGTCAAAAGCTAAAAAAGATGATGCCCTCACATCAGCCACTTGTG
TTGCTGATCATGACGTAAGCCATGACGTCTACATGAGCTAAGCACATACGTCAGAGCAAGACCTCTAC
AAAACTGGATCCTTACCGCTATGGGTAAGATTCCGATGAATTTCGGGAAACCTCCTCGGGCTATCAGC
TTAAGGTGGCTCCTACACCTCACATGTAGGCTATCAGCTTAGAAAAATGTCAAAGATACCAATTACTA
TATAAGGTTTTGCTATTCATTGAAAGCAGTAGTGACTGATTTGTATATA
>MinSyn_1817|Strength:0.026387978
GCGTGTCGTTTTAGTGAGGTCCATCAACAAATAATCCAAGTAAGGCACCTCACATGTAGGCTAATGAC
GTAAGCCATGACGTCTAGACCAGCCACTTGTGTCTGCCTTGATGATTTATGACCCCCGCCGATGACGC
GGGACGACCACTATGCTGAAGATAAGATAATAATGTTGAAGATAAGACCAATTAGGTTGTCTGCACCT
CCTATATAAGGTTTTGCTATTCATTGAAAGCAGTAGTGACTGATTTGTATATA
>MinSyn_1530|Strength:0.026525711
GCGTGTCGTTTTAGTGAGGTCACTATCAGCCAGCTTAGCAAGACCAATTTCGGGAAACCTCCTCGAGG
AAAAAGAAGAGGTTCTGCACCTCTGACGTAAGGGATGACGCACACTGCACCTCACATGTAGGCTATCA
TCCTTACCGCTATGGGTAAGATTTAGGTTGTCTGCACCTCACATTTTTCAACAATATGCCCAATTAGG
TTGTCTGCACCTCACCTATATAAGGTTTTGCTATTCATTGAAAGCAGTAGTGACTGATTTGTATATA
>MinSyn_1003|Strength:0.026710807
GCGTGTCGTTTTAGTGAGGTCACTATCAGCGGTAGGTCACGACCACTGACGTAAGGGATGACGCACAC
ATGTAGGCTATCAGCTTAGCAAGACCTCAGGTGGCTCCTACCTCCATCAACAAATAATCCAAGTAAGC
CTTGATGTCAGAAGATCAAAGGGCTAGTGGTGGAGCACGACAATCTTGCCTGCCTTGACTATATAAGG
TTTTGCTATTCATTGAAAGCAGTAGTGACTGATTTGTATATA
>MinSyn_1964|Strength:0.026810648
GCGTGTCGTTTTAGTGAGGTGAAGATAAGATAATAATGTTGAAGATAAGATCGCGGTAGGTCAAAAAT
GTCAAAGATAGGACCGATGCTGATCTTGTCAGAAGATCAAAGGGCTACCAATTAGGTTGTCTTCACTA
TCAGCCTATGCCCAATTAGGTTGTCTGCACGGAAAAAGAAGAGGTAGACCTCTACAAAACTGGTACTT
GTGTACAGTGGGAGCCACCATCAGCTTAGCAAGACCTCTACAAAACTGGTGGTGGAGCACGACAGCTA
GGAGGACCGATGCTGATCTTGCCTTGAAGCATCTTCCCACTGCTAGGAGGACCGATGCTGATCTTGAT
GAGCTAAGCACATACGTCAGCCCTATATAAGGTTTTGCTATTCATTGAAAGCAGTAGTGACTGATTTG
TATATA
>MinSyn_1322|Strength:0.026849537
GCGTGTCGTTTTAGTGAGGAAGATTGATGAAAAGTCAAAAACAAAAATCAATTATGGTAGGTCACGAC
CAGGTGGCTCCTACTGCACCTCACATGTAGGCTAATGACGTAAGCCATGACGTCTAGGTACTTGTGAT
CGAAAGGACAGTACTACAAAACTGGTACTTGTGTTGAAGATAAGATAATAATGTTGAAGATAAGACTC
ACTGCTAGGAGGACCGATGCTCTATATAAGGTTTTGCTATTCATTGAAAGCAGTAGTGACTGATTTGT
ATATA
>MinSyn_1203|Strength:0.027049334

GCGTGTCGTTTTAGTGAGGATCCTTACCGCTATGGGTAAGATTGACCTCTACAAAACTGGTACTTTTC
AACAATGCCCAATTAGGTTGTCTGTCAGAAGATCAAAGGGCTAGCACCTCACATCAGTGGTCCCTCCA
CTCACATGTAGGCTATCAGCTTAGCATGAAGATAAGATAATAATGTTGAAGATAAGACGATGCTGAAA
TTTCGGGAAACCTCCTCGGGTCACGACCACTATGCCCAATTAAGCAAGTGGATGCCCAATTAGGTTGT
CTGCAGCTTTGTCAAAAGCTAAAAAAGATGATGCAGGGCTCACTGCTAGGAGATGACGTAAGCCATGA
CGTCTACCTGCCTTGACTATATAAGGTTTTGCTATTCATTGAAAGCAGTAGTGACTGATTTGTATATA
>MinSyn_1875|Strength:0.027232371
GCGTGTCGTTTTAGTGAGGTTATGACCCCCGCCGATGACGCGGGAGCCTGACGTAAGGGATGACGCAC
ACTGCACCTCACATGTAGGCTTCCATCAACAAATAATCCAAGTAAGTAGGTCACGACCACTATGCCCA
ATTAGGTTGGTGGAGCACGACACAGGGCTATTGCGATAAAGGAAAGGAGGACCGATCTATATAAGGTT
TTGCTATTCATTGAAAGCAGTAGTGACTGATTTGTATATA
>MinSyn_1914|Strength:0.02765369
GCGTGTCGTTTTAGTGAGGAATTTCGGGAAACCTCCTCGCTCTACAAAACTGGTACTTGTGTACAGGG
TCTCTCTGCCGACAGTGGTCCCAAAACCTCTACAAAAAGATTGATGAAAAGTCAAAAACAAAAATCAA
TTATGCCCAATTAGTCCATCAACAAATAATCCAAGTAAGGATCTTGCCTGCCCTGACGTAAGGGATGA
CGCACAGGAGGACAACCATTATTGCGTTAGCAAGACCTCTACAAATCAGAAGATCAAAGGGCTAGCCT
CTATATAAGGTTTTGCTATTCATTGAAAGCAGTAGTGACTGATTTGTATATA
>MinSyn_1645|Strength:0.027797816
GCGTGTCGTTTTAGTGAGGCTGACGTAAGGGATGACGCACACAGGTGGCTCCTACAGCTTAGCAAGAA
AGATTGATGAAAAGTCAAAAACAAAAATCAATTATAAAACTGGTACTTGTGTACAGGGCTCCAAATAT
TTCTTGTCAACCACGTCTACAACACCTCACATGTAGGCCTATATAAGGTTTTGCTATTCATTGAAAGC
AGTAGTGACTGATTTGTATATA
>MinSyn_1256|Strength:0.027996543
GCGTGTCGTTTTAGTGAGGTCCATCAACAAATAATCCAAGTAAGCTAGGAGGACCGATGCTGATCTTG
CCTATTGCGATAAAGGAAAGGCGATGCTGATCTTCAGTGGTCCCTCCACGCCCAGCAAGTGGATCCTC
ACATGTAGGCTATCATGACGTAAGCCATGACGTCTATAGGAGGACCGATGCTGAAAGATTGATGAAAA
GTCAAAAACAAAAATCAATTATAGGTCACGACCACTATGCCCACTATATAAGGTTTTGCTATTCATTG
AAAGCAGTAGTGACTGATTTGTATATA
>MinSyn_1885|Strength:0.028000408
GCGTGTCGTTTTAGTGAGGATGACGTAAGCCATGACGTCTATTGCCTGCCGGAAAAAGAAGAGGTCAC
GACCACTATGCCCAATTAGTGGTGGAGCACGACACATGTAGGCTAAGATTGATGAAAAGTCAAAAACA
AAAATCAATTATCTGCACCTCACATGTAGGCTATCAGCTATATAAGGTTTTGCTATTCATTGAAAGCA
GTAGTGACTGATTTGTATATA
>MinSyn_1484|Strength:0.028185233
GCGTGTCGTTTTAGTGAGGATCGAAAGGACAGTAAGCAAGACCTCTACAAAACTGGAAGATTGATGAA
AAGTCAAAAACAAAAATCAATTATTACAGGAACCACGTCTACAACTCTACAAAACTGGTACTTGTGTA
CAGGGATCCTTACCGCTATGGGTAAGATTGTCTGCACCTCACATGTAGGCTATCAGCTTAACCATTAT
TGCGGCACCTCACATGTATCACTATCAGCAGCAAGAATTTCGGGAAACCTCCTCGTACAGGGCTCACT
CTGACGTAAGGGATGACGCACAGACCACTATGCCCAATAGGTGGCTCCTACGGTTGTCTGCACCTCAC
ATGTCTATATAAGGTTTTGCTATTCATTGAAAGCAGTAGTGACTGATTTGTATATA
>MinSyn_1924|Strength:0.02819587
GCGTGTCGTTTTAGTGAGGAGCAAGTGGATACAGGGCTCACTGCAAGATTGATGAAAAGTCAAAAACA
AAAATCAATTATGACCGATGCTGATCTTGCCTGCGCACACCAGCATGTGTTGATCACCAGCTGTACTT
GTGTACAGGGCTCACTGCTAGTCTCTCTGCCGACAGTGGTCCCAAAGTAGGTCACGACCATCACTATC
AGCGTACTTTATGACCCCCGCCGATGACGCGGGAACGACCACTATGCATTGCGATAAAGGAAAGGCTA
CATGAGCTAAGCACATACGTCAGTAGGTCACGACCACTATGCCCAAACCATTATTGCGCTACAAAACT
GGTACTCTATATAAGGTTTTGCTATTCATTGAAAGCAGTAGTGACTGATTTGTATATA
>MinSyn_1857|Strength:0.028347089
GCGTGTCGTTTTAGTGAGGTTATGACCCCCGCCGATGACGCGGGAAAGACCTCTACAAAACTGGTACG
CTTTGTCAAAAGCTAAAAAAGATGATGCGCTGATCTTGCCTGGTGGAGCACGACACTGCAAAGATTGA
TGAAAAGTCAAAAACAAAAATCAATTATCCTCACATGTAGGCTATCAGCTCTCTCTGCCGACAGTGGT
CCCAAACTCACTGCTAGGAGGATCCTTACCGCTATGGGTAAGATTTGCCCAATTAGGTTGTCTTTTCA
ACAAAATTAGGTTGTCTGCACCTCACCTGACGTAAGGGATGACGCACACAGCTTAGCAAGACCTCTAC
ACTATATAAGGTTTTGCTATTCATTGAAAGCAGTAGTGACTGATTTGTATATA

>MinSyn_1598|Strength:0.028476233
GCGTGTCGTTTTAGTGAGGATTGCGATAAAGGAAAGGCTTGCATCCTTACCGCTATGGGTAAGATTTG
CCCAATTAGGCTGACGTAAGGGATGACGCACAGCCTTTCCATCAACAAATAATCCAAGTAAGCGGTAG
GTCACGATGAAGATAAGATAATAATGTTGAAGATAAGACTCACATGTAGGGAAAAAGAAGAGGTATTA
GGCTATATAAGGTTTTGCTATTCATTGAAAGCAGTAGTGACTGATTTGTATATA
>MinSyn_1040|Strength:0.028709452
GCGTGTCGTTTTAGTGAGGATCCTTACCGCTATGGGTAAGATTCACTATGCCCAATCTGACGTAAGGG
ATGACGCACAACGACCACTATGCCCAACAGTGGTCCCTCCACATGCTGATCTTGCCTGCCATGAGCTA
AGCACATACGTCAGCGGTAGGTCACGACCACAAGATTGATGAAAAGTCAAAAACAAAAATCAATTATT
TAGCAAGACCTCTACAAAATGAAGATAAGATAATAATGTTGAAGATAAGAACAAAACTGGTAGCACAC
CAGCATGTGTTGATCACCAGCTATGTAGGATTGCGATAAAGGAAAGGCCCAATTAGGTTGTCTGCACC
ATCGAAAGGACAGTAAGGTCACGACCACTATGCCCAATTAGGTCTATATAAGGTTTTGCTATTCATTG
AAAGCAGTAGTGACTGATTTGTATATA
>MinSyn_1749|Strength:0.028765713
GCGTGTCGTTTTAGTGAGGAGGTGGCTCCTACGCAAGACCTCTACAAATTATGACCCCCGCCGATGAC
GCGGGAAACTCTGACGTAAGGGATGACGCACACTTAATTTCGGGAAACCTCCTCGTATCAGCCACTTG
TGTCACTGATCGAAAGGACAGTAAACTGGTAAGCAAGTGGATCACATGTAGGCTATCAGCTTAGCAAC
TATATAAGGTTTTGCTATTCATTGAAAGCAGTAGTGACTGATTTGTATATA
>MinSyn_1447|Strength:0.028801708
GCGTGTCGTTTTAGTGAGGATCGAAAGGACAGTATACAAAGTGGGAGCCACCACCCAGCTTTGTCAAA
AGCTAAAAAAGATGATGCGGACCGATGCTGATCTTGCCTGCCTTGGCACACCAGCATGTGTTGATCAC
CAGCTCTACAATGAAGCATCTTCCGATCTTGCCTGCCTTGATGTTATGACCCCCGCCGATGACGCGGG
ATTAGGTTGTCTGCTCAGAAGATCAAAGGGCTACTGATCTAAGATTGATGAAAAGTCAAAAACAAAAA
TCAATTATCACCTCACATGTAGGCTATATGACGTAAGCCATGACGTCTACTGATCTTGCCTGCCTTCT
ATATAAGGTTTTGCTATTCATTGAAAGCAGTAGTGACTGATTTGTATATA
>MinSyn_1129|Strength:0.028880261
GCGTGTCGTTTTAGTGAGGAGGTGGCTCCTACCTTGCCTGCCTTGAAATTTCGGGAAACCTCCTCGCG
ACCACTATGCCCAATTAGGTTTGAAGATAAGATAATAATGTTGAAGATAAGATTCCATCAACAAATAA
TCCAAGTAAGCCTCTACAAAACTGGTACTTGTGCTTTGTCAAAAGCTAAAAAAGATGATGCGATTGCG
ATAAAGGAAAGGACTGGTTCTCTCTGCCGACAGTGGTCCCAAAAACTGGTACTTGTGTACAGGGCTCA
CTATCAGCCTTAGCAAGACCTCTACAAAACTGGTACATGACGTAAGCCATGACGTCTACCTCCTATAT
AAGGTTTTGCTATTCATTGAAAGCAGTAGTGACTGATTTGTATATA
>MinSyn_1821|Strength:0.028958314
GCGTGTCGTTTTAGTGAGGAATTTCGGGAAACCTCCTCGTGCACCTCTGGTGGAGCACGACAGGACGC
TTTGTCAAAAGCTAAAAAAGATGATGCCAAAACTGGTACTTGTGTACAGGGAAAAATGTCAAAGATAA
TCAGCTTAGCATCCATCAACAAATAATCCAAGTAAGGCTAGGAGGACCGATGCAGTGGTCCCTCCACG
CTAGGAGGACCGATGCTGATCAACCATTATTGCGCCCAATTAGGTTGTCTGACGTAAGGGATGACGCA
CAGGCTCACTGCTAGGAGGACCGATGCTGATCCAGCCACTTGTGTTGATCTTGCCTGCCCTATATAAG
GTTTTGCTATTCATTGAAAGCAGTAGTGACTGATTTGTATATA
>MinSyn_1745|Strength:0.028974012
GCGTGTCGTTTTAGTGAGGATCCTTACCGCTATGGGTAAGATTCTCACATGTAGGCTATCAGCTTAGG
GAAAAAGAAGAGGTCGGTAGGTCACGACCAAGATTGATGAAAAGTCAAAAACAAAAATCAATTATTAT
GAGCAAGTGGATTAGGTTGTCACTATCAGCTACTTGTGTACAGGGCTCACTGCTATCAGAAGATCAAA
GGGCTAAGGACCGATGCTGAGCTTTGTCAAAAGCTAAAAAAGATGATGCCATGTAGGCTATCAGCTTA
GCAAGACATGACGTAAGCCATGACGTCTATTGTCTTGAAGCATCTTCCAGGAGGACCGCTATATAAGG
TTTTGCTATTCATTGAAAGCAGTAGTGACTGATTTGTATATA
>MinSyn_1949|Strength:0.029094295
GCGTGTCGTTTTAGTGAGGTCTCTCTGCCGACAGTGGTCCCAAACTGCTAGGTCAGAAGATCAAAGGG
CTACCAGTGGTCCCTCCACCTGGGAAAAAGAAGAGGTACTATGCCCAATTAGGTTGTCTGCACCTATG
ACGTAAGCCATGACGTCTAACCACAACCACGTCTACAAGTGTACAGGGAAGATTGATGAAAAGTCAAA
AACAAAAATCAATTATCTTGCCTGCCTTGACTATATAAGGTTTTGCTATTCATTGAAAGCAGTAGTGA
CTGATTTGTATATA
>MinSyn_1625|Strength:0.029106419
GCGTGTCGTTTTAGTGAGGAGCAAGTGGATCACGACCACTATGCCCAATTAGCAGTGGTCCCTCCACG

TACTTGTGTACAGATCCTTACCGCTATGGGTAAGATTTGTGTACAGGGCTCACTGCTAGGTTTTCAAC
AAATCAGCTTAGCAAGACCTCTTCCATCAACAAATAATCCAAGTAAGAGGTCACGACCACTGAAGATA
AGATAATAATGTTGAAGATAAGAGGAGGACCGATGCTAACCACGTCTACAATGTCTGCACCATGACGT
AAGCCATGACGTCTATTAGCAAGACCTCTACAAAAAATTTCGGGAAACCTCCTCGACTGCTCTATATA
AGGTTTTGCTATTCATTGAAAGCAGTAGTGACTGATTTGTATATA
>MinSyn_1669|Strength:0.02923485
GCGTGTCGTTTTAGTGAGGCAGTGGTCCCTCCACTTAGGTTGTCTGCACCTCACATCCTTACCGCTAT
GGGTAAGATTTCACTGCAAAAATGTCAAAGATAAACTGGTACTTAATTTCGGGAAACCTCCTCGTCTG
CACCTCACATGTACAAATATTTCTTGTCTATGTGGGAGCCACCACCACTATGCCCAATTAGGTTGTCT
AAGATTGATGAAAAGTCAAAAACAAAAATCAATTATTGCTAGGAGGACCGGAAAAAGAAGAGGTGGTT
GTCTGCACCTCCTGACGTAAGGGATGACGCACATCACTGCTAGGAGGACCGATGCTGACTATATAAGG
TTTTGCTATTCATTGAAAGCAGTAGTGACTGATTTGTATATA
>MinSyn_1076|Strength:0.029263333
GCGTGTCGTTTTAGTGAGGATCGAAAGGACAGTACTAGGAGGACCGATGAAGATTGATGAAAAGTCAA
AAACAAAAATCAATTATTCACATGTAGGCTATCAGCTTAGCAAGACTTTTCAACAATATCCATCAACA
AATAATCCAAGTAAGCTCACATGTACTGACGTAAGGGATGACGCACAGATGCTGATCTTGGGAAAAAG
AAGAGGTCCAAACCATTATTGCGGATCTTGCCTGCCTTGATGATCTATATAAGGTTTTGCTATTCATT
GAAAGCAGTAGTGACTGATTTGTATATA
>MinSyn_1524|Strength:0.029306882
GCGTGTCGTTTTAGTGAGGCAGTGGTCCCTCCACCTTGCTCAGAAGATCAAAGGGCTAGGCTATCAGC
TTAGCAAGACCTTGAAGCATCTTCCGGTACTTGTGTACAGGGCTCACTGCATTGCGATAAAGGAAAGG
ATGCCCAATAGGTGGCTCCTACGTAGGTCACGACCACTAATCCTTACCGCTATGGGTAAGATTTTGCC
TGCAAGATTGATGAAAAGTCAAAAACAAAAATCAATTATAGACCTCTACAAATCGAAAGGACAGTAGT
CTGCACCTCACATGCTGACGTAAGGGATGACGCACAGTCACGACCACTATGCCCAATTCTATATAAGG
TTTTGCTATTCATTGAAAGCAGTAGTGACTGATTTGTATATA
>MinSyn_1290|Strength:0.029376935
GCGTGTCGTTTTAGTGAGGATTGCGATAAAGGAAAGGACTGCTAGGTGGCTCCTACATCTGACGTAAG
GGATGACGCACATGCTAGGAGGACCGATGCAGCCACTTGTGTACGACCACTATGCCCAATTAGGTTGT
CAACCACGTCTACAACACTGCTAGGAGGACCGATGCTGATCTTTTTCAACAATCTATATAAGGTTTTG
CTATTCATTGAAAGCAGTAGTGACTGATTTGTATATA
>MinSyn_1071|Strength:0.029398465
GCGTGTCGTTTTAGTGAGGTGAAGCATCTTCCCATGTAGTCTCTCTGCCGACAGTGGTCCCAAATACA
GGGCTCACTGAATTTCGGGAAACCTCCTCGAGGCTTCCATCAACAAATAATCCAAGTAAGTGCACCTC
ACATCTGACGTAAGGGATGACGCACAAGGACCGATGCTGATCTTGCCTGCCAAAAATGTCAAAGATAA
TCAGAAGATCAAAGGGCTACAATTAGGTTGTCCTATATAAGGTTTTGCTATTCATTGAAAGCAGTAGT
GACTGATTTGTATATA
>MinSyn_1078|Strength:0.029419088
GCGTGTCGTTTTAGTGAGGGCTTTGTCAAAAGCTAAAAAAGATGATGCCTCACTGCTAGGAGGACCAT
GACGTAAGCCATGACGTCTATGTGTACAGGGCTCACTGCTAGGAATCGAAAGGACAGTACATTATGAC
CCCCGCCGATGACGCGGGAAAAACCAAATATTTCTTGTTGCTAGGAGGACCGATGCTGCTATATAAGG
TTTTGCTATTCATTGAAAGCAGTAGTGACTGATTTGTATATA
>MinSyn_1945|Strength:0.029592706
GCGTGTCGTTTTAGTGAGGCTGACGTAAGGGATGACGCACAAGACCTCTACAAAACTGGTACTTGTGT
ACATCCATCAACAAATAATCCAAGTAAGAGACCTCTACAAAACTGGTACTTTCACTATCAGCTGTGAA
GATAAGATAATAATGTTGAAGATAAGACTGCCCTATATAAGGTTTTGCTATTCATTGAAAGCAGTAGT
GACTGATTTGTATATA
>MinSyn_1202|Strength:0.029626197
GCGTGTCGTTTTAGTGAGGAACCACGTCTACAACGACCACTATGCGCACACCAGCATGTGTTGATCAC
CAGCTCGGTAGATTGCGATAAAGGAAAGGCATTTTCAACAACTGCTAGGAGGACCGATGCTGATCTTG
CCTAGGTGGCTCCTACGTCTGCACCTCACATGTAGGCTTGAAGATAAGATAATAATGTTGAAGATAAG
ACAGGGCTCACTGCTTATGACCCCCGCCGATGACGCGGGAGTAGGTCACGATGAGCTAAGCACATACG
TCAGCTGCTAGGAGGACCGATCCTTACCGCTATGGGTAAGATTGCAAGACCTCTACTATATAAGGTTT
TGCTATTCATTGAAAGCAGTAGTGACTGATTTGTATATA
>MinSyn_1419|Strength:0.029656738

GCGTGTCGTTTTAGTGAGGAAGATTGATGAAAAGTCAAAAACAAAAATCAATTATTGCACCTCACATG
TAGGCTATCAGCTTAATCGAAAGGACAGTAGCTAATCCTTACCGCTATGGGTAAGATTGACCGATGCT
GATCTTCCATCAACAAATAATCCAAGTAAGTCTGCACCTCACATGTAGGCTATCAGCTTTTTCAACAA
GTCACGACCACTATGTGGGAGCCACCACTATCAGCTTAGCAAGACCTCTACAAATATTTCTTGTATCA
GATGACGTAAGCCATGACGTCTATAGGCTATCAGCTTAGCAAGACCTCTACCTATATAAGGTTTTGCT
ATTCATTGAAAGCAGTAGTGACTGATTTGTATATA
>MinSyn_1358|Strength:0.029808951
GCGTGTCGTTTTAGTGAGGGCACACCAGCATGTGTTGATCACCAGCTATGCTGATCTTGCCTGCCCAG
CCACTTGTGTAGGTTGTCTGCACCTCACATCTGACGTAAGGGATGACGCACAAGGCTATCAGCTTAGC
ATCCTTACCGCTATGGGTAAGATTCTTAGCAAGACCTCTACAAAACTGGTAACCACGTCTACAAGATG
CTGATCCTATATAAGGTTTTGCTATTCATTGAAAGCAGTAGTGACTGATTTGTATATA
>MinSyn_1948|Strength:0.029948342
GCGTGTCGTTTTAGTGAGGTCCATCAACAAATAATCCAAGTAAGGTCTCAGCCACTTGTGTACAAAAC
TGGTACTTGTGTACAGGGCTCAAAGATTGATGAAAAGTCAAAAACAAAAATCAATTATCTCACATGTA
GGCTGGAAAAAGAAGAGGTATCAGCTATCCTTACCGCTATGGGTAAGATTTAGCTTTGTCAAAAGCTA
AAAAAGATGATGCGGCTTCTCTCTGCCGACAGTGGTCCCAAAGTCTGACGTAAGGGATGACGCACATA
CTTGTGTACATTATGACCCCCGCCGATGACGCGGGATGCCCAATTAGGTTGCTATATAAGGTTTTGCT
ATTCATTGAAAGCAGTAGTGACTGATTTGTATATA
>MinSyn_1282|Strength:0.029986531
GCGTGTCGTTTTAGTGAGGTCTCTCTGCCGACAGTGGTCCCAAATCACTGCTAGGAGGACCGATGCTC
AGCCACTTGTGTCCCAATTAGGTTGTCTGCACCTCAGCTTTGTCAAAAGCTAAAAAAGATGATGCACT
GCTAGGATCACTATCAGCCTATGCCCAATTAGGTTGTCTGAGGTGGCTCCTACGCACCTCACATCCTT
ACCGCTATGGGTAAGATTTCAGCTTAGCAATCGAAAGGACAGTACACTATGCCCAATTAGGTGGAAAA
AGAAGAGGTTCGCATGACGTAAGCCATGACGTCTAAGGACCGATGCTCTATATAAGGTTTTGCTATTC
ATTGAAAGCAGTAGTGACTGATTTGTATATA
>MinSyn_1928|Strength:0.029986847
GCGTGTCGTTTTAGTGAGGATCCTTACCGCTATGGGTAAGATTAAAACTGGTACTTGTGTACAGGGCT
CACTGGCTTTGTCAAAAGCTAAAAAAGATGATGCGTAGGCTATCAGCTTAGCAAGAATTGCGATAAAG
GAAAGGGGTAGGTCACGACTCAGAAGATCAAAGGGCTACCCAATTAGGTTGTCTGCACCTCACATTGG
TGGAGCACGACACTCCAAATATTTCTTGTGTAGGTCACGACCACTATGCCCAATTCTGACGTAAGGGA
TGACGCACAGGTCACAGTGGTCCCTCCACTCACATGTAGGCTATCACTATATAAGGTTTTGCTATTCA
TTGAAAGCAGTAGTGACTGATTTGTATATA
>MinSyn_1177|Strength:0.030180156
GCGTGTCGTTTTAGTGAGGATGACGTAAGCCATGACGTCTACCTCTACAAAACTGGTACTTGTGTACA
GATCCTTACCGCTATGGGTAAGATTCACGACCACTATGCCCAATTAGGTTGTCTGAACCATTATTGCG
TAGATCGAAAGGACAGTAACATGTAGGCTCTATATAAGGTTTTGCTATTCATTGAAAGCAGTAGTGAC
TGATTTGTATATA
>MinSyn_1962|Strength:0.030383533
GCGTGTCGTTTTAGTGAGGTCTCTCTGCCGACAGTGGTCCCAAACACCTCACATGTAGGCTATCATCA
GAAGATCAAAGGGCTAAAACTGGTACTTGTGTACAGGGCTTCACTATCAGCGCAAGACCTCTACAAAA
CTGGTACTTGTGAGCAAGTGGATCTGCTAGGAGGACCGATGCTGATTATGACCCCCGCCGATGACGCG
GGAACTGCTAGGAATGAGCTAAGCACATACGTCAGCCTCACATGTAGGCTATCAGCTTAGCAAGACAA
ATATTTCTTGTTAGGTTGTCTGCACCTCACATGTAGGCTCTATATAAGGTTTTGCTATTCATTGAAAG
CAGTAGTGACTGATTTGTATATA
>MinSyn_1852|Strength:0.030410964
GCGTGTCGTTTTAGTGAGGATCGAAAGGACAGTAGGCTCACTGCTAGGAGGACCGATGCTTGAAGATA
AGATAATAATGTTGAAGATAAGAGGTAGGTCACGACCACTATATGACGTAAGCCATGACGTCTATAAA
AAATGTCAAAGATATAATCCTTACCGCTATGGGTAAGATTGTCACGACCACTATGCCCAATTAGGTTT
TTCAACAAAAACTATATAAGGTTTTGCTATTCATTGAAAGCAGTAGTGACTGATTTGTATATA
>MinSyn_1352|Strength:0.030538666
GCGTGTCGTTTTAGTGAGGTGAAGCATCTTCCTAGGAGGACCGATGAAGATTGATGAAAAGTCAAAAA
CAAAAATCAATTATCTCACATGTAGGCTATCAGCTTAGCAACAGCCACTTGTGTATCAGCTTAGCAAG
ACCTCTACAAAACTGAACCACGTCTACAATAGCAAGACCTCTACAAAACTGGTATCCATCAACAAATA
ATCCAAGTAAGCTGGTTCAGAAGATCAAAGGGCTAGCTTAGCAAGACCTCTACAAAACTGGTACTGTG

GGAGCCACCACTATGACGTAAGCCATGACGTCTATGGTACTCTATATAAGGTTTTGCTATTCATTGAA
AGCAGTAGTGACTGATTTGTATATA
>MinSyn_1822|Strength:0.030571413
GCGTGTCGTTTTAGTGAGGTCTCTCTGCCGACAGTGGTCCCAAATGCTGATCTTGCCTGCCTTGATGA
TGCACACCAGCATGTGTTGATCACCAGCTGCAAGAATTGCGATAAAGGAAAGGAGGTTGTCTGCACCT
CACATGATCCTTACCGCTATGGGTAAGATTCTGCTAGGAGGACGGAAAAAGAAGAGGTCACCTCAATG
AGCTAAGCACATACGTCAGACAAAACTGGTACTTGTGTACAGGGCCAAATATTTCTTGTCACATGATC
GAAAGGACAGTAGCAAGACCTCTACAAAACTGGTACTTGTGCTATATAAGGTTTTGCTATTCATTGAA
AGCAGTAGTGACTGATTTGTATATA
>MinSyn_1768|Strength:0.030662246
GCGTGTCGTTTTAGTGAGGTGGTGGAGCACGACAGCTAGGAGGACCGATGGCTTTGTCAAAAGCTAAA
AAAGATGATGCCTCACTGCTAGGAGGACCGATGCTGACTGACGTAAGGGATGACGCACACGCGGTAGG
TCACGACGCACACCAGCATGTGTTGATCACCAGCTTGAGGTGGCTCCTACCCTCACATGTAGGCTATC
AGCTTACTATATAAGGTTTTGCTATTCATTGAAAGCAGTAGTGACTGATTTGTATATA
>MinSyn_1756|Strength:0.030829521
GCGTGTCGTTTTAGTGAGGTGAAGATAAGATAATAATGTTGAAGATAAGAACAGGGCTCACTGCTAGG
AATTTCGGGAAACCTCCTCGCCACTATGCCAGGTGGCTCCTACGCTATCAGCTTAGCAAGACCTCTTC
TCTCTGCCGACAGTGGTCCCAAACCTGCCTTGATCCTTACCGCTATGGGTAAGATTGTGTACATGAAG
CATCTTCCTCACATGTAGGCTATCAGCTTAGCAATGAGCTAAGCACATACGTCAGCCAATTAGGTTGT
CTGCACCTCAGAAGATCAAAGGGCTAACAGGGCTCACTCTATATAAGGTTTTGCTATTCATTGAAAGC
AGTAGTGACTGATTTGTATATA
>MinSyn_1492|Strength:0.030842137
GCGTGTCGTTTTAGTGAGGTGAAGCATCTTCCTCACGACAATTTCGGGAAACCTCCTCGCAAAACTGA
ACCATTATTGCGTCTACAAAACTGGTACTTGTGTACAGGTGGCTCCTACACCTCACATGTAGGCTATT
TTTCAACAATATCAGCTTAGCAAGACCTCTACAAATGAGCTAAGCACATACGTCAGAGGACCGATTCT
CTCTGCCGACAGTGGTCCCAAAACAGGGCTTTGTCAAAAGCTAAAAAAGATGATGCAGGCTATCAGCT
TAGCAAGACCTCTACAAAAAATGTCAAAGATATCTCTATATAAGGTTTTGCTATTCATTGAAAGCAGT
AGTGACTGATTTGTATATA
>MinSyn_1980|Strength:0.030933655
GCGTGTCGTTTTAGTGAGGAACCACGTCTACAAACCTCTACAAATGAAGATAAGATAATAATGTTGAA
GATAAGAACATGTAGGCTATCAGCTTAGCAAGAATTGCGATAAAGGAAAGGCAAGACCTCTACAAAAC
TGGTACTTGTGTAGGAAAAAGAAGAGGTACCTCACATGTAGGCTATCAGCTATCCTTACCGCTATGGG
TAAGATTACCACTATGCCCAATTATGACGTAAGCCATGACGTCTATGGTATTATGACCCCCGCCGATG
ACGCGGGATGTACAGGGCTCACTGCTAGGAGGACCGCTATATAAGGTTTTGCTATTCATTGAAAGCAG
TAGTGACTGATTTGTATATA
>MinSyn_1441|Strength:0.031032834
GCGTGTCGTTTTAGTGAGGAACCATTATTGCGTGTAGGCTATCAGCTTAGCAAGAGCACACCAGCATG
TGTTGATCACCAGCTCACATGTAGGCTATCAGCTTAGCAAGAGGAAAAAGAAGAGGTAAGTGAAGATA
AGATAATAATGTTGAAGATAAGATGTAGGGTGGGAGCCACCAGATCTTGCCTGCCTTTCCATCAACAA
ATAATCCAAGTAAGAGGAGGACCGATGCTGATCCTTACCGCTATGGGTAAGATTTGATATGAGCTAAG
CACATACGTCAGTGCACCTCACATGTAGGCTATCAGCCTATATAAGGTTTTGCTATTCATTGAAAGCA
GTAGTGACTGATTTGTATATA
>MinSyn_1690|Strength:0.031152887
GCGTGTCGTTTTAGTGAGGTTATGACCCCCGCCGATGACGCGGGACTGATCTTGCCTGCCTTTGAAGC
ATCTTCCTGCCTGCCTTGATGATCTGACGTAAGGGATGACGCACACACGACCACTATGCCCAATTAGG
AGGTGGCTCCTACGTCACGACCACTATGCTCACTATCAGCAGCAAGACCTCTACAAAACTGGTACTAT
ATAAGGTTTTGCTATTCATTGAAAGCAGTAGTGACTGATTTGTATATA
>MinSyn_1956|Strength:0.031224063
GCGTGTCGTTTTAGTGAGGCTGACGTAAGGGATGACGCACACCTCACATGTAGGCTATCAGCTTAGCT
GAAGATAAGATAATAATGTTGAAGATAAGAACAGGGCTCACTGCTAGGAGGACCGAGTGGGAGCCACC
ACATGTAGGCAGTGGTCCCTCCACACCTATATAAGGTTTTGCTATTCATTGAAAGCAGTAGTGACTGA
TTTGTATATA
>MinSyn_1660|Strength:0.031258365
GCGTGTCGTTTTAGTGAGGATGACGTAAGCCATGACGTCTACCGATGCTGATCTTGCCAAGATTGATG

AAAAGTCAAAAACAAAAATCAATTATCTGCCTTGGCTTTGTCAAAAGCTAAAAAAGATGATGCTCACC
AAATATTTCTTGTCTCACTGCTAGGCTATATAAGGTTTTGCTATTCATTGAAAGCAGTAGTGACTGAT
TTGTATATA
>MinSyn_1784|Strength:0.031329136
GCGTGTCGTTTTAGTGAGGGCACACCAGCATGTGTTGATCACCAGCTCGCGGTAGATGACGTAAGCCA
TGACGTCTAAGGTTGTCTATCGAAAGGACAGTATGTACAGGGCTGCTTTGTCAAAAGCTAAAAAAGAT
GATGCACAGCCACTTGTGTTCAGTCAGAAGATCAAAGGGCTAACTCTATATAAGGTTTTGCTATTCAT
TGAAAGCAGTAGTGACTGATTTGTATATA
>MinSyn_1277|Strength:0.031825814
GCGTGTCGTTTTAGTGAGGATCGAAAGGACAGTAGCCTGCCTTGATCAAATATTTCTTGTGTCTGCAC
CTCACATGTAGGCTATATGACGTAAGCCATGACGTCTATATGCCCAATTAGGTTGTCAACCATTATTG
CGATCAGCTTATTGCGATAAAGGAAAGGTTAGCAAGACCTCTACAAAACTGGTACTTGCTATATAAGG
TTTTGCTATTCATTGAAAGCAGTAGTGACTGATTTGTATATA
>MinSyn_1739|Strength:0.031867253
GCGTGTCGTTTTAGTGAGGATCCTTACCGCTATGGGTAAGATTGATCTATTGCGATAAAGGAAAGGCT
ACAAAACTGGTACTCAGAAGATCAAAGGGCTACCATGAAGATAAGATAATAATGTTGAAGATAAGAAC
TATGCCCAATTAGGTTGTCAAATATTTCTTGTACTGGTACTTGTGTACAGGGGCTTTGTCAAAAGCTA
AAAAAGATGATGCCTATGACGTAAGCCATGACGTCTACATGTAGGCTATCAGCTAACCACGTCTACAA
TGCACCTCACATGTAGGCTATCACTATATAAGGTTTTGCTATTCATTGAAAGCAGTAGTGACTGATTT
GTATATA
>MinSyn_1915|Strength:0.032036786
GCGTGTCGTTTTAGTGAGGTCCATCAACAAATAATCCAAGTAAGGGACCGATGCTGATAACCACGTCT
ACAAGACCACTATGCCCAATTAGGTTGTCTAGCAAGTGGATTAAGATTGATGAAAAGTCAAAAACAAA
AATCAATTATCACGCAGTGGTCCCTCCACCTCACATGTAGGCTATCAGCTTAGCAAGACTGAAGATAA
GATAATAATGTTGAAGATAAGACACTATGCCCAATTATGACCCCCGCCGATGACGCGGGAACTGCTAA
TGAGCTAAGCACATACGTCAGCTATCCTATATAAGGTTTTGCTATTCATTGAAAGCAGTAGTGACTGA
TTTGTATATA
>MinSyn_1840|Strength:0.032269512
GCGTGTCGTTTTAGTGAGGTGAAGCATCTTCCAAATGACGTAAGCCATGACGTCTATCACTGCTAGGA
GGGGAAAAAGAAGAGGTATTAGGTTGTCTGCACCTCACATGTAGGCGCTTTGTCAAAAGCTAAAAAAG
ATGATGCCAGCTTAGCAAGACCTCTACACTATATAAGGTTTTGCTATTCATTGAAAGCAGTAGTGACT
GATTTGTATATA
>MinSyn_1457|Strength:0.03249702
GCGTGTCGTTTTAGTGAGGATCGAAAGGACAGTACCGATGCTGATCTTGCCTGCCTTGATTCAGAAGA
TCAAAGGGCTAGATGCTGATCTTGCCTGCCTTGATGCAGCCACTTGTGTAAAACTGGTACTTGTGTAC
AGGGCTCACTTGAAGATAAGATAATAATGTTGAAGATAAGATAGGTTGTCTGCACCTCACATGAACCA
TTATTGCGCATGAGCTAAGCACATACGTCAGATCAGCTGCACACCAGCATGTGTTGATCACCAGCTGC
TCACTGCTAGGAGGACCGACTATATAAGGTTTTGCTATTCATTGAAAGCAGTAGTGACTGATTTGTAT
ATA
>MinSyn_1255|Strength:0.032614602
GCGTGTCGTTTTAGTGAGGTTATGACCCCCGCCGATGACGCGGGAGCCTGCCTTGATGATCAGTGGTC
CCTCCACCTGGTACTTGTGTACAGGGCTCACTGCTAGCAAATATTTCTTGTCTATGCCCAATTAGGTT
GTCTGCACCTTCTCTCTGCCGACAGTGGTCCCAAATAGGAGGAAACCATTATTGCGTTGTGTACAGGG
CTCACTGCTAGGTCAGAAGATCAAAGGGCTACACATGACGTAAGCCATGACGTCTATTGTGTACAGGG
CTCACTGCTAGGAGGACTATATAAGGTTTTGCTATTCATTGAAAGCAGTAGTGACTGATTTGTATATA
>MinSyn_1692|Strength:0.032626337
GCGTGTCGTTTTAGTGAGGATTGCGATAAAGGAAAGGGGCTATCAGCTTAGCAAGACCTCTGGAAAAA
GAAGAGGTCTGGTACTTGTGTACAGCAGCCACTTGTGTCCTGCCTTGATGATCAAATATTTCTTGTGT
TGTCTGCACCTCACATGTAGGCTATCAGGCACACCAGCATGTGTTGATCACCAGCTCGATGCTGATCT
TGCCTGATGAGCTAAGCACATACGTCAGATCAGCTTAGCAACCACGTCTACAATAGCAAGACCTCTAC
AAAACTGGTACTTGTCTATATAAGGTTTTGCTATTCATTGAAAGCAGTAGTGACTGATTTGTATATA
>MinSyn_1332|Strength:0.032714788
GCGTGTCGTTTTAGTGAGGCAAATATTTCTTGTGTTGTCTGCACCTCACATGTAGGCTATCATGGTGG
AGCACGACAAGGTCACGAGGTGGCTCCTACTAGGAGGAAAAATGTCAAAGATAACAGGGCTCACTGCT

ATCTCTCTGCCGACAGTGGTCCCAAAGGTCACGACCACTATGCCAGCAAGTGGATCCACTATGCCCAA
TTAGGCTGACGTAAGGGATGACGCACAGTACAGTTATGACCCCCGCCGATGACGCGGGAGCTTAGCAA
GACCTCTACAAAACTCTATATAAGGTTTTGCTATTCATTGAAAGCAGTAGTGACTGATTTGTATATA
>MinSyn_1707|Strength:0.033309259
GCGTGTCGTTTTAGTGAGGGCACACCAGCATGTGTTGATCACCAGCTATTAGGTTGTCTCTCTCTGCC
GACAGTGGTCCCAAACACTGCTAGGAACCATTATTGCGACTTGTGTACAGGGCTCATGAAGATAAGAT
AATAATGTTGAAGATAAGAATCTTGCCTGCCTTGCAGTGGTCCCTCCACCTGCTAGGAGGACCGATGC
TGATCTTGCATGAGCTAAGCACATACGTCAGGCGGAGGTGGCTCCTACCTCACTGCTAGGAGGACCGA
TGCTGATCTCTATATAAGGTTTTGCTATTCATTGAAAGCAGTAGTGACTGATTTGTATATA
>MinSyn_1019|Strength:0.03370915
GCGTGTCGTTTTAGTGAGGTGAAGCATCTTCCTACAGGGCTCACTGCTAGGAGGACCGATGATGACGT
AAGCCATGACGTCTAGCAAGACCTCTACAAAACGCTTTGTCAAAAGCTAAAAAAGATGATGCACGACC
ATGGTGGAGCACGACACGACCACTATGCCCAATTAGGTCTATATAAGGTTTTGCTATTCATTGAAAGC
AGTAGTGACTGATTTGTATATA
>MinSyn_1107|Strength:0.033780574
GCGTGTCGTTTTAGTGAGGAAGATTGATGAAAAGTCAAAAACAAAAATCAATTATGGTACTTGTGTAC
AGGGCTCACTCACTATCAGCGCTAGGAGGACCGATGCTGATAGGTGGCTCCTACTGTCTGCACCTCAC
ATCTCTCTGCCGACAGTGGTCCCAAATGTGTACAGGTGAAGCATCTTCCAATTAGGTTTTCAACAACA
GCTTAGCAAGACCTCTACAACTGACGTAAGGGATGACGCACACAAGACCTCTACAAAACTGGTACTTG
TGTCTATATAAGGTTTTGCTATTCATTGAAAGCAGTAGTGACTGATTTGTATATA
>MinSyn_1351|Strength:0.03382199
GCGTGTCGTTTTAGTGAGGTCTCTCTGCCGACAGTGGTCCCAAATGATATTGCGATAAAGGAAAGGCA
AATCAGAAGATCAAAGGGCTATGCCCAATTATGACGTAAGCCATGACGTCTAGGTACTTGTGTAATCG
AAAGGACAGTATGTACAGGGCTCACTGAACCATTATTGCGAGACAGTGGTCCCTCCACTACTATATAA
GGTTTTGCTATTCATTGAAAGCAGTAGTGACTGATTTGTATATA
>MinSyn_1172|Strength:0.033825604
GCGTGTCGTTTTAGTGAGGATCGAAAGGACAGTACATGTAGGCTAATCCTTACCGCTATGGGTAAGAT
TCTAGGAGGACCGATGCTGATGAAGCATCTTCCGTAGGAACCACGTCTACAATGTCTCACTATCAGCA
GGACTCTCTCTGCCGACAGTGGTCCCAAATACTTGTGTACAGGGTTATGACCCCCGCCGATGACGCGG
GAACCTCTACAAAACTGGTACTAGGTGGCTCCTACGATGCTGATCATGAGCTAAGCACATACGTCAGG
TCACGACTATATAAGGTTTTGCTATTCATTGAAAGCAGTAGTGACTGATTTGTATATA
>MinSyn_1681|Strength:0.033917155
GCGTGTCGTTTTAGTGAGGCAGTGGTCCCTCCACACAGGGCTCACTGCTAGGAGGACTTATGACCCCC
GCCGATGACGCGGGATCGCGTGGTGGAGCACGACATTGCCTGCCTTGATTGAAGATAAGATAATAATG
TTGAAGATAAGACTGATCTTGCCTGCCTTGATGATAAAAATGTCAAAGATACCAATTAGGTTATGAGC
TAAGCACATACGTCAGCTATGCCCAAGATTGATGAAAAGTCAAAAACAAAAATCAATTATGAGGACCG
ATGCTGCTATATAAGGTTTTGCTATTCATTGAAAGCAGTAGTGACTGATTTGTATATA
>MinSyn_1673|Strength:0.033940344
GCGTGTCGTTTTAGTGAGGTTATGACCCCCGCCGATGACGCGGGATGCCTGCCTTGATGATACTGACG
TAAGGGATGACGCACATTAGCAAGACCTCTACAAAAGTGGGAGCCACCAAGGTCACGACCACTATGCC
CAATTAGATGACGTAAGCCATGACGTCTAACCTCACATGTAGGCTATCAGCTTAGCAAGCAGCCACTT
GTGTCAAAATTTCGGGAAACCTCCTCGCACGACCACTATGCCCAATTAGGTCAGAAGATCAAAGGGCT
AGCCCAATTAGGTTGTCTGCACCTCTGAAGATAAGATAATAATGTTGAAGATAAGAGGGCTCACTGCT
AGGAGGACCGATGCATCGAAAGGACAGTATTAGGTTGTCTGCACCCTATATAAGGTTTTGCTATTCAT
TGAAAGCAGTAGTGACTGATTTGTATATA
>MinSyn_1215|Strength:0.034010572
GCGTGTCGTTTTAGTGAGGTCACTATCAGCTTAGGTTGTCTGCACCTCACATGTGCACACCAGCATGT
GTTGATCACCAGCTGCTTAGCAAGACCTCTACAATCCATCAACAAATAATCCAAGTAAGGTACAGGGC
TCAGTGGGAGCCACCAGATCTTGCCTGCCTTGGAAAAAGAAGAGGTATGCTGATCAAATATTTCTTGT
CTTAGCAAGACCTCTACAAATGACGTAAGCCATGACGTCTAAGACCTCTACAAAACTGGTACTTGTGT
ACTATATAAGGTTTTGCTATTCATTGAAAGCAGTAGTGACTGATTTGTATATA
>MinSyn_1873|Strength:0.034245843
GCGTGTCGTTTTAGTGAGGTTTTCAACAAATTAGGTTGTCTGCACAGTGGTCCCTCCACGTCACGACC
ACTATGCCCAAAGGTGGCTCCTACACCACTATGCCCAATTAGGTGTGGGAGCCACCACAGGGAAAAAT

GTCAAAGATAGCCCAATTAGGTTGTCTGCACCTCACATGTATTGCGATAAAGGAAAGGGCCCAATTAG
GTTGTCTGCACCTCACATGTCTGACGTAAGGGATGACGCACAAGCAAGACCTCTACAAAACTGGTCTA
TATAAGGTTTTGCTATTCATTGAAAGCAGTAGTGACTGATTTGTATATA
>MinSyn_1584|Strength:0.034279473
GCGTGTCGTTTTAGTGAGGCTGACGTAAGGGATGACGCACATTGTCTGCACCTATTGCGATAAAGGAA
AGGTCTGCACCTCACATGTAGGCTATCGGAAAAAGAAGAGGTTACTTGTGTACAGGGCTCACTGCTAG
AGGTGGCTCCTACAGGCTATATAAGGTTTTGCTATTCATTGAAAGCAGTAGTGACTGATTTGTATATA
>MinSyn_1664|Strength:0.03428934
GCGTGTCGTTTTAGTGAGGCAAATATTTCTTGTGGCTCACTGCTAGGAGGACCGATTCAGAAGATCAA
AGGGCTAGCTGATCTTGCCTGCCTTATGACGTAAGCCATGACGTCTACACCTCACATGTAGGCTAGCT
TTGTCAAAAGCTAAAAAAGATGATGCTATCAGCTTAGCAAGACCTCTACAAACTATATAAGGTTTTGC
TATTCATTGAAAGCAGTAGTGACTGATTTGTATATA
>MinSyn_1871|Strength:0.034323268
GCGTGTCGTTTTAGTGAGGTCACTATCAGCATTAGGTTGTCCAAATATTTCTTGTTCTACAAGGAAAA
AGAAGAGGTACGACCACTATGCCCAATCCATCAACAAATAATCCAAGTAAGTGCTGATCTTGCTTATG
ACCCCCGCCGATGACGCGGGACTGAAGCATCTTCCCCTCACATGTAGGCTATCAGCTTAGCAAGTGGA
TCTTGTGTAAGGTGGCTCCTACCCTCTACAAAACTGGTACTTGTATGAGCTAAGCACATACGTCAGCC
TATATAAGGTTTTGCTATTCATTGAAAGCAGTAGTGACTGATTTGTATATA
>MinSyn_1074|Strength:0.034720594
GCGTGTCGTTTTAGTGAGGTCAGAAGATCAAAGGGCTACACTGCTAGGAGGACCGATGCTGATCTTGA
TGACGTAAGCCATGACGTCTAGTCACGACCACTATGCCCAATTAGGCAGTGGTCCCTCCACATCTTTG
AAGCATCTTCCCTAGGAGGACAAAAATGTCAAAGATAACCCTATATAAGGTTTTGCTATTCATTGAAA
GCAGTAGTGACTGATTTGTATATA
>MinSyn_1583|Strength:0.034817439
GCGTGTCGTTTTAGTGAGGTTTTCAACAATTAGCAAGACCTCTACAAAACTGGTACTTTCCATCAACA
AATAATCCAAGTAAGACAAAACTGGTACTTGTGTACTGAAGCATCTTCCCACATGTAGGCTATCAGCT
TAGCAAGACCTAGCAAGTGGATGGAGGACCGACAAATATTTCTTGTTAGCAAGACCTCCTGACGTAAG
GGATGACGCACATGATGATAACCATTATTGCGTACAAAACTGGTACTGGTGGAGCACGACACCTATAT
AAGGTTTTGCTATTCATTGAAAGCAGTAGTGACTGATTTGTATATA
>MinSyn_1288|Strength:0.035027297
GCGTGTCGTTTTAGTGAGGAGGTGGCTCCTACGTAGGTCACGACCACTATGCCCATCCATCAACAAAT
AATCCAAGTAAGTAGCAAGACCTCTACAAAACTGGATTGCGATAAAGGAAAGGTGCACCAAATATTTC
TTGTCACATGTAGGCTATCAGCTTAGCTTTTCAACAATGGTACTTGTGTACAGGGCTATGACGTAAGC
CATGACGTCTATCTTGCCTGCAACCATTATTGCGTTGTCTGCACCTCACATGTAGGCTACTATATAAG
GTTTTGCTATTCATTGAAAGCAGTAGTGACTGATTTGTATATA
>MinSyn_1911|Strength:0.035238308
GCGTGTCGTTTTAGTGAGGATGACGTAAGCCATGACGTCTATGCACCTCACATGTAGGCTATCAGCTT
AGCAATTTCGGGAAACCTCCTCGGCGCTTTGTCAAAAGCTAAAAAAGATGATGCGTAGGTCACGCAGC
CACTTGTGTTACACTATATAAGGTTTTGCTATTCATTGAAAGCAGTAGTGACTGATTTGTATATA
>MinSyn_1449|Strength:0.035319412
GCGTGTCGTTTTAGTGAGGATCGAAAGGACAGTACAAAACTGGTACTTGTAGGTGGCTCCTACAACTG
GTACTTGTGTATTTTCAACAATGCCTTAACCACGTCTACAAACTGGTACTTGTGTACATGAGCTAAGC
ACATACGTCAGGGCTCAAATATTTCTTGTTCACTGCTAGGAGGACCCAGCCACTTGTGTAGGCTATCA
GCTTTGAAGATAAGATAATAATGTTGAAGATAAGAACAGGGCTCACTGCTAGGAGGACCCTATATAAG
GTTTTGCTATTCATTGAAAGCAGTAGTGACTGATTTGTATATA
>MinSyn_1741|Strength:0.035355146
GCGTGTCGTTTTAGTGAGGTCCATCAACAAATAATCCAAGTAAGTCTTGCGCACACCAGCATGTGTTG
ATCACCAGCTTTGTCTGCACCTCACATGTAGGCTATCAGCTGACGTAAGGGATGACGCACAGGTACTT
GTGTACAGGGCTCACTGCTTGGTGGAGCACGACACCGATGCTGATCTTGCCTGCCTTCTATATAAGGT
TTTGCTATTCATTGAAAGCAGTAGTGACTGATTTGTATATA
>MinSyn_1464|Strength:0.035822282
GCGTGTCGTTTTAGTGAGGTCCATCAACAAATAATCCAAGTAAGAAAACTGGTACTTAAGATTGATGA
AAAGTCAAAAACAAAAATCAATTATAATTAGGTTGTCTGCACCTCACGTGGGAGCCACCACATGTAGG
CTATCAGCTTAGCAAGACCTTCTCTCTGCCGACAGTGGTCCCAAATACTTGTGTACAGGCTGACGTAA

GGGATGACGCACAAGGTCACGACCACTATGCCCAATTCACTATCAGCTGCTAGCTATATAAGGTTTTG
CTATTCATTGAAAGCAGTAGTGACTGATTTGTATATA
>MinSyn_1788|Strength:0.035920296
GCGTGTCGTTTTAGTGAGGCAAATATTTCTTGTGCTTAGCAAGACCTCTACATCGAAAGGACAGTATT
GTCTGCACCTCACATGTAGGCTATCTTATGACCCCCGCCGATGACGCGGGAGGTTGTCTGCACCTCAC
ATTTTCAACAATCACGACCACTATGCCCAATTAGGGCACACCAGCATGTGTTGATCACCAGCTGACCT
CTACAAAACTGGTACTTGTGTACAATGAGCTAAGCACATACGTCAGTCAGCTCTATATAAGGTTTTGC
TATTCATTGAAAGCAGTAGTGACTGATTTGTATATA
>MinSyn_1947|Strength:0.036184301
GCGTGTCGTTTTAGTGAGGATCCTTACCGCTATGGGTAAGATTGGTAGGTCACGACCACTATGCCCAA
TTAATTGCGATAAAGGAAAGGGTTGTCTGCACCTCAAATTTCGGGAAACCTCCTCGTATGCCCAATTA
GGTTGAAGATAAGATAATAATGTTGAAGATAAGATAGGTTGTCGCACACCAGCATGTGTTGATCACCA
GCTACCTCTACAAAACCTGACGTAAGGGATGACGCACAGGACCGATGCTGATCTATATAAGGTTTTGC
TATTCATTGAAAGCAGTAGTGACTGATTTGTATATA
>MinSyn_1823|Strength:0.03680938
GCGTGTCGTTTTAGTGAGGTTTTCAACAACTCTACAAAACTGGTACTTGTATGACGTAAGCCATGACG
TCTATGTACAGGGCTCATGAAGCATCTTCCTACAGTGGGAGCCACCAGACCACTATTCTCTCTGCCGA
CAGTGGTCCCAAAAGACCTCTACAACTATATAAGGTTTTGCTATTCATTGAAAGCAGTAGTGACTGAT
TTGTATATA
>MinSyn_1630|Strength:0.036840517
GCGTGTCGTTTTAGTGAGGATCGAAAGGACAGTAGTCTGCACCTCACATGCTTTGTCAAAAGCTAAAA
AAGATGATGCAGTCTCTCTGCCGACAGTGGTCCCAAAACGACCACTATGCCCAATTAGCAAATATTTC
TTGTGGGCTCACTGCTAGGAATGAGCTAAGCACATACGTCAGTAGCAAGACCTCTACAAAACTGGTAT
GAAGCATCTTCCTGCCCAATTAGGTTGTCTGCACCTCACATGCTATATAAGGTTTTGCTATTCATTGA
AAGCAGTAGTGACTGATTTGTATATA
>MinSyn_1723|Strength:0.036946569
GCGTGTCGTTTTAGTGAGGCTGACGTAAGGGATGACGCACATACTTGTGTACAGGGCGGAAAAAGAAG
AGGTACTAATTTCGGGAAACCTCCTCGCAAAACTGGTACTTGTGTACAGGGCAAAAATGTCAAAGATA
TGTACAGGGCTATATAAGGTTTTGCTATTCATTGAAAGCAGTAGTGACTGATTTGTATATA
>MinSyn_1021|Strength:0.037523589
GCGTGTCGTTTTAGTGAGGTTATGACCCCCGCCGATGACGCGGGATATCAGCTTAGCAAGACCTCTAC
AAAATGGTGGAGCACGACAAGGTCACGACCACTATGCCCAATTAGGTATCCTTACCGCTATGGGTAAG
ATTGGAGGACCGATGCTGATCTTGCCTGCATGACGTAAGCCATGACGTCTAGCCCAATTAGGTTGTCT
GCACGTGGGAGCCACCATAGGAGGACCGATGCTGATCCTATATAAGGTTTTGCTATTCATTGAAAGCA
GTAGTGACTGATTTGTATATA
>MinSyn_1939|Strength:0.037632911
GCGTGTCGTTTTAGTGAGGCAGCCACTTGTGTCAAAACTGGTATCACTATCAGCACTTGTGTACAGGG
CTCACTATCCTTACCGCTATGGGTAAGATTAGGGCTCACTGCTAATGAGCTAAGCACATACGTCAGCC
TCACATGTAGGCTATCAAATTTCGGGAAACCTCCTCGGTACAGGGCTTTGTCAAAAGCTAAAAAAGAT
GATGCTAGCAAGACCTCTACAAAACTGGTACTTGTCTATATAAGGTTTTGCTATTCATTGAAAGCAGT
AGTGACTGATTTGTATATA
>MinSyn_1075|Strength:0.03765868
GCGTGTCGTTTTAGTGAGGAAAAATGTCAAAGATATATCAGCTTAGCAAGACCTCTACAAAAATCGAA
AGGACAGTAGCACCTCAAGGTGGCTCCTACCATGTAGGCATGAGCTAAGCACATACGTCAGGACCACT
ATGCCAGCAAGTGGATTCACATCCTTACCGCTATGGGTAAGATTCCTGCCTGAAGATAAGATAATAAT
GTTGAAGATAAGAGACCGATGCTGATCTTGCCTGCCTTGCTATATAAGGTTTTGCTATTCATTGAAAG
CAGTAGTGACTGATTTGTATATA
>MinSyn_1436|Strength:0.037662506
GCGTGTCGTTTTAGTGAGGGCACACCAGCATGTGTTGATCACCAGCTCAAGACCTCTACAAAACTGGT
CAGCCACTTGTGTCTATGCCCAATTAGGTTGAAGCATCTTCCCTACAAAACTGGTACTTGTGTAACCA
CGTCTACAACCACTATGCCCAATTAGGTTGTCTGGTGGGAGCCACCAGGACCATGAGCTAAGCACATA
CGTCAGGGTAGGTCACGACCACTATGCCCAATTAGGCTATATAAGGTTTTGCTATTCATTGAAAGCAG
TAGTGACTGATTTGTATATA
>MinSyn_1340|Strength:0.037677702

GCGTGTCGTTTTAGTGAGGATGACGTAAGCCATGACGTCTACCGCTTTGTCAAAAGCTAAAAAAGATG
ATGCACCTCTACAAAACTGATGAGCTAAGCACATACGTCAGGTACTTGTGTACAGGGCTCCAGCCACT
TGTGTGGTTGTCTGCACCTCACATGTAGGCAGTGGTCCCTCCACGTACAGGGCTCACTGCTAGGAGGA
CCGATCCTTACCGCTATGGGTAAGATTGCTATCACTATCAGCCTCTATATAAGGTTTTGCTATTCATT
GAAAGCAGTAGTGACTGATTTGTATATA
>MinSyn_1291|Strength:0.037694745
GCGTGTCGTTTTAGTGAGGATTGCGATAAAGGAAAGGCCTCTACAAAACTGGTACTTGTATCGAAAGG
ACAGTACATGTAGGTTTTCAACAACGATGCTGATAACCATTATTGCGACCGATGCTGATCTTGCCTAG
CAAGTGGATAAAACTGGTACTTGTGTACAGGGATGACGTAAGCCATGACGTCTAAGGTCACGACCACT
ATGCCCAATTAGGTTGCAAATATTTCTTGTTTGTGCTATATAAGGTTTTGCTATTCATTGAAAGCAGT
AGTGACTGATTTGTATATA
>MinSyn_1097|Strength:0.037756714
GCGTGTCGTTTTAGTGAGGTCCATCAACAAATAATCCAAGTAAGCACTGCTAGGAGGACCGATGCTGA
TCTTGCTGGTGGAGCACGACACACGACCACTATGCCCAATTAGGTTGTCTGATCCTTACCGCTATGGG
TAAGATTAGGACCGATGCTGATCTATGAGCTAAGCACATACGTCAGGCTATCAGCTTAGCAAGACCTC
TACATCACTATCAGCCAATTAGGTTGTCTGCACCTCTATATAAGGTTTTGCTATTCATTGAAAGCAGT
AGTGACTGATTTGTATATA
>MinSyn_1522|Strength:0.037784038
GCGTGTCGTTTTAGTGAGGTGAAGCATCTTCCAGCAAGACCTCTACAAAACTGGTACAGGTGGCTCCT
ACCTGCCTTGATGACAAATATTTCTTGTAGGTCACGACCACTATGCCCAATTAGGTTGTGGGAGCCAC
CACAGGGCGGAAAAAGAAGAGGTCAGCTTAGCAAATCCTTACCGCTATGGGTAAGATTATTAGGTTGT
CTGCACCTGACGTAAGGGATGACGCACAATGTAGGCTCTATATAAGGTTTTGCTATTCATTGAAAGCA
GTAGTGACTGATTTGTATATA
>MinSyn_1523|Strength:0.037792821
GCGTGTCGTTTTAGTGAGGATGACGTAAGCCATGACGTCTATGTGTACAGGGCTCACTGCTAGGAGGA
CCGTCAGAAGATCAAAGGGCTATTGGTGGAGCACGACATCAAGGTGGCTCCTACGACCTCTACAAAAC
TGGTACTCTATATAAGGTTTTGCTATTCATTGAAAGCAGTAGTGACTGATTTGTATATA
>MinSyn_1954|Strength:0.03783838
GCGTGTCGTTTTAGTGAGGAGGTGGCTCCTACGGACCGATGCTTGGTGGAGCACGACATCGCGGTAAT
GACGTAAGCCATGACGTCTATCTACAAAACTGGTACTTGTGTACAGGGCACACCAGCATGTGTTGATC
ACCAGCTAGGACCGATGCTGATCTTGCCTCTATATAAGGTTTTGCTATTCATTGAAAGCAGTAGTGAC
TGATTTGTATATA
>MinSyn_1359|Strength:0.037868422
GCGTGTCGTTTTAGTGAGGTGAAGCATCTTCCCACTTTTTCAACAACTGATCTTGCCTGCAACCATTA
TTGCGTAGGAACCACGTCTACAACAAAACTGGTACTTGTGTACAGGGCTCACAATTTCGGGAAACCTC
CTCGTTGCCTGCCTTAAGATTGATGAAAAGTCAAAAACAAAAATCAATTATTTACAGCCACTTGTGTC
CCAATTAATGACGTAAGCCATGACGTCTAAGGACCGATGCTATATAAGGTTTTGCTATTCATTGAAAG
CAGTAGTGACTGATTTGTATATA
>MinSyn_1069|Strength:0.037875138
GCGTGTCGTTTTAGTGAGGTGAAGCATCTTCCAATTAGGTTGTCTGCACCTCACATTGCGATAAAGGA
AAGGCTTTTCAACAAAGCTTAGCAAGACCTCTACAAAAAATTTCGGGAAACCTCCTCGAATGAGCTAA
GCACATACGTCAGGGTCACGACCACTATGCCCAATTAGTCAGAAGATCAAAGGGCTATTTTATGACCC
CCGCCGATGACGCGGGAACAGGGCTCACTGCTAGGAGGCTATATAAGGTTTTGCTATTCATTGAAAGC
AGTAGTGACTGATTTGTATATA
>MinSyn_1628|Strength:0.038223418
GCGTGTCGTTTTAGTGAGGGCTTTGTCAAAAGCTAAAAAAGATGATGCCACTGCTAGGAGGACCGATG
CTGATGGTGGAGCACGACATCTACAATGACGTAAGCCATGACGTCTAGGACCGATGCTGATCTTGCTG
AAGCATCTTCCCACATGTAGGCTATCAGCTTAGCAAGACCCTATATAAGGTTTTGCTATTCATTGAAA
GCAGTAGTGACTGATTTGTATATA
>MinSyn_1122|Strength:0.038248778
GCGTGTCGTTTTAGTGAGGCTGACGTAAGGGATGACGCACATGATGATAGTGGGAGCCACCAGCACCT
CACTCAGAAGATCAAAGGGCTATGATCTTGCCTGCCTTTTATGACCCCCGCCGATGACGCGGGACTGA
TCTTGCCTATATAAGGTTTTGCTATTCATTGAAAGCAGTAGTGACTGATTTGTATATA
>MinSyn_1432|Strength:0.038521396

GCGTGTCGTTTTAGTGAGGAGGTGGCTCCTACGAGGACCGATGCTGATCTTGCCCTGACGTAAGGGAT
GACGCACAGTGTAATTTCGGGAAACCTCCTCGTGCCTTGATCTCTCTGCCGACAGTGGTCCCAAATTA
GCAAGACCTCTACAAAACTGGTCTATATAAGGTTTTGCTATTCATTGAAAGCAGTAGTGACTGATTTG
TATATA
>MinSyn_1472|Strength:0.038559512
GCGTGTCGTTTTAGTGAGGTCAGAAGATCAAAGGGCTATGGTACTTGTGTACAGGCACACCAGCATGT
GTTGATCACCAGCTAGGCTATGAGCTAAGCACATACGTCAGTTAGCAAGACCTCTACAAAACTGGTAC
TAAGATTGATGAAAAGTCAAAAACAAAAATCAATTATTCACGACCACTATGCCCAATAGGTGGCTCCT
ACGCAAGACCTCTACAAAACTGGTACTTGTGCTATATAAGGTTTTGCTATTCATTGAAAGCAGTAGTG
ACTGATTTGTATATA
>MinSyn_1509|Strength:0.038617148
GCGTGTCGTTTTAGTGAGGTTATGACCCCCGCCGATGACGCGGGATACTTGTGTACAGGGCTCACTGC
AACCACGTCTACAAGCAGCTTTGTCAAAAGCTAAAAAAGATGATGCGGCTATTTTCAACAAGCTTAGC
AAGACCTCTACAAAACTGGCAGCCACTTGTGTGCAAATATTTCTTGTTATCAGCTTAGCAAGACCTCT
ACAAAATGAGCTAAGCACATACGTCAGGGCTATATAAGGTTTTGCTATTCATTGAAAGCAGTAGTGAC
TGATTTGTATATA
>MinSyn_1774|Strength:0.038813273
GCGTGTCGTTTTAGTGAGGAGCAAGTGGATGGGCTTCACTATCAGCCAGCTTAGCAAGATTTTCAACA
AGATGCAAAAATGTCAAAGATACAGCTTAGCAAGACCTCCAGTGGTCCCTCCACTAGGTTGTCTGCAC
CTCACAATGAGCTAAGCACATACGTCAGATCAGCTTAGCAATTGCGATAAAGGAAAGGTGCTAGGAGG
ACCGATGCTGATCTGTGGGAGCCACCACTCTATATAAGGTTTTGCTATTCATTGAAAGCAGTAGTGAC
TGATTTGTATATA
>MinSyn_1173|Strength:0.038841341
GCGTGTCGTTTTAGTGAGGTGGTGGAGCACGACACTGCACCTCACATGTAGGCTATCAGCTTAGCAAG
TGGATCACCTCACATGTAGGCTATCAGCTTAAAAATGTCAAAGATAGTCTGAACCATTATTGCGAGCA
AGACCTCTACAAAACTGGTACTCAGAAGATCAAAGGGCTAACTTGTGTACAGGGCTCACCTGACGTAA
GGGATGACGCACAAATTAGGTTGTCTGCTATATAAGGTTTTGCTATTCATTGAAAGCAGTAGTGACTG
ATTTGTATATA
>MinSyn_1001|Strength:0.038882142
GCGTGTCGTTTTAGTGAGGTCACTATCAGCGCAGCAAGTGGATCACCTCACATGTAGGCTATCAGCTT
AGCAGTGGGAGCCACCACAGGGCTCACTGCTGAAGATAAGATAATAATGTTGAAGATAAGATTAGCAA
GACCTCTACAAAACATGAGCTAAGCACATACGTCAGCTCACATATCCTTACCGCTATGGGTAAGATTC
CTCACATGTAGGCTATCAGCTTAGCAAGCTATATAAGGTTTTGCTATTCATTGAAAGCAGTAGTGACT
GATTTGTATATA
>MinSyn_1406|Strength:0.038906411
GCGTGTCGTTTTAGTGAGGTCCATCAACAAATAATCCAAGTAAGGTGTAATTTCGGGAAACCTCCTCG
CAGGGCTCACTGCTAGGAGGACAGGTGGCTCCTACCTGGTGGAGCACGACACAAATGAGCTAAGCACA
TACGTCAGATCAAACCATTATTGCGCTCTACCAGTGGTCCCTCCACCTCACATGTAGGCTATCAGCTT
AGCAAATCCTTACCGCTATGGGTAAGATTACCTATATAAGGTTTTGCTATTCATTGAAAGCAGTAGTG
ACTGATTTGTATATA
>MinSyn_1839|Strength:0.038968452
GCGTGTCGTTTTAGTGAGGAATTTCGGGAAACCTCCTCGGGTAGGTCACGACCACTATGGCTTTGTCA
AAAGCTAAAAAAGATGATGCGCGGATTGCGATAAAGGAAAGGGACCTCTACATCACTATCAGCTAGGT
CACGACCACTATGCCCAATGAGCTAAGCACATACGTCAGTATCAGTGGTCCCTCCACTACTTGTGTAC
ATTTTCAACAATGCTGATCTTGCCTGCCTATATAAGGTTTTGCTATTCATTGAAAGCAGTAGTGACTG
ATTTGTATATA
>MinSyn_1602|Strength:0.039039844
GCGTGTCGTTTTAGTGAGGTCTCTCTGCCGACAGTGGTCCCAAACACCTCAGAAGATCAAAGGGCTAT
GTACAGGGCTCACTGCTAGGAGTCCATCAACAAATAATCCAAGTAAGAGCAAGACCTCTACAAAACTG
GTAATGAGCTAAGCACATACGTCAGTAGGAGGACCGATGCTGATCTTGCCTTTTTCAACAATGTGTAC
AGGGCTCACAGTGGTCCCTCCACTCACCTATATAAGGTTTTGCTATTCATTGAAAGCAGTAGTGACTG
ATTTGTATATA
>MinSyn_1331|Strength:0.039153927
GCGTGTCGTTTTAGTGAGGAAGATTGATGAAAAGTCAAAAACAAAAATCAATTATCTGATCTTGCCTG

CCTTGCAGCCACTTGTGTGATGATATCGAAAGGACAGTACCAATTAGGTATCCTTACCGCTATGGGTA
AGATTGCTGGTGGAGCACGACAGATATGAGCTAAGCACATACGTCAGCCGATGCTGATCTTGCCTTAT
GACCCCCGCCGATGACGCGGGAACCTCTACTATATAAGGTTTTGCTATTCATTGAAAGCAGTAGTGAC
TGATTTGTATATA
>MinSyn_1769|Strength:0.039244143
GCGTGTCGTTTTAGTGAGGTCCATCAACAAATAATCCAAGTAAGCAAGACCTCTACAAAACTGGTACT
TGTAGGTGGCTCCTACCAGGGCTCACTGCTAATTGCGATAAAGGAAAGGCAAAATCGAAAGGACAGTA
GCAAGACCTCTACAAAACTGGTACTTGTGAGCAAGTGGATCTTAGCAAGACCTCTATGAGCTAAGCAC
ATACGTCAGCAAAACTGGTACTTCTATATAAGGTTTTGCTATTCATTGAAAGCAGTAGTGACTGATTT
GTATATA
>MinSyn_1640|Strength:0.039416757
GCGTGTCGTTTTAGTGAGGAAAAATGTCAAAGATAGCTTGAAGATAAGATAATAATGTTGAAGATAAG
AACAGTGGTCCCTCCACTTAGGTTGTCTGCACCTCACATGTAGTCAGAAGATCAAAGGGCTATATCAG
CTTAGCAAGACCTCAGGTGGCTCCTACTTAGGTTGTCTGCACCTCACATGTAGGATGAGCTAAGCACA
TACGTCAGTAGGTCACGACCACTCTATATAAGGTTTTGCTATTCATTGAAAGCAGTAGTGACTGATTT
GTATATA
>MinSyn_1458|Strength:0.039604634
GCGTGTCGTTTTAGTGAGGTCCATCAACAAATAATCCAAGTAAGCTTAGCAAGACAGGTGGCTCCTAC
GGAGGACAATTTCGGGAAACCTCCTCGAGGTTGTCTGCATCTCTCTGCCGACAGTGGTCCCAAAACGA
CCATGAGCTAAGCACATACGTCAGTCACTGCTAGGAGGACCGATTGGTGGAGCACGACAGACCATTGC
GATAAAGGAAAGGTTGCCTGCCTTGCTATATAAGGTTTTGCTATTCATTGAAAGCAGTAGTGACTGAT
TTGTATATA
>MinSyn_1023|Strength:0.039664696
GCGTGTCGTTTTAGTGAGGTCACTATCAGCGCGGAAAAAGAAGAGGTGCTCACTGCAAAAATGTCAAA
GATAACTGCTAGGAGGACCGATGCTGATCTTGCTTTTCAACAAGCAACAGCCACTTGTGTACCACATG
AGCTAAGCACATACGTCAGGCTATCAGCTTAGCCAAATATTTCTTGTCCTCTACAAAACTGGTACTTG
TGTAATCGAAAGGACAGTACTGCCTATATAAGGTTTTGCTATTCATTGAAAGCAGTAGTGACTGATTT
GTATATA
>MinSyn_1516|Strength:0.039855518
GCGTGTCGTTTTAGTGAGGAGGTGGCTCCTACCCTCACATGTAGGCTATCAGCTTAGCAGCCACTTGT
GTCTTGCCTGCCTTGATAATTTCGGGAAACCTCCTCGAGACCTCTACAAAACTGGTACTTGTGTGGTG
GAGCACGACATGTAGGCTATCAAATATTTCTTGTAAACCAGTGGTCCCTCCACCCTTGATGATGACGT
AAGCCATGACGTCTACTTGTGTCTATATAAGGTTTTGCTATTCATTGAAAGCAGTAGTGACTGATTTG
TATATA
>MinSyn_1606|Strength:0.040005833
GCGTGTCGTTTTAGTGAGGGTGGGAGCCACCATACAGGGCTCACTGCTACAAATATTTCTTGTTGCTA
GGAGTCCATCAACAAATAATCCAAGTAAGTCTGCACCTCACATGTAGGCTATCAGTCACTATCAGCGC
TTAGCAAGATGACGTAAGCCATGACGTCTAACCGATGCTGATCTTGCCTGCCTTGAGGAAAAAGAAGA
GGTTCACATGTAGGCTATCCTATATAAGGTTTTGCTATTCATTGAAAGCAGTAGTGACTGATTTGTAT
ATA
>MinSyn_1421|Strength:0.040179224
GCGTGTCGTTTTAGTGAGGTTTTCAACAATACTTGTGTACAGGGGTGGGAGCCACCAACAGTGGTCCC
TCCACTCTTGCCTGCCTTGATATCCTTACCGCTATGGGTAAGATTGCTCACTGCTAAAAATGTCAAAG
ATAGCTCACTGCTAGGAGGACCGATGGCTTTGTCAAAAGCTAAAAAAGATGATGCAAAACTGACGTAA
GGGATGACGCACATGCTCTATATAAGGTTTTGCTATTCATTGAAAGCAGTAGTGACTGATTTGTATAT
A
>MinSyn_1042|Strength:0.040224872
GCGTGTCGTTTTAGTGAGGTGAAGATAAGATAATAATGTTGAAGATAAGAGTACAGGTTTTCAACAAG
GTAGGTCACGACCACTATGCATGAGCTAAGCACATACGTCAGACTATGCCCTCACTATCAGCGTCACG
ACCACTATGCCCCAGCCACTTGTGTATGTAGGCTATCAGCTTAGCAAGACCTCTCCATCAACAAATAA
TCCAAGTAAGGACCTCTACCTATATAAGGTTTTGCTATTCATTGAAAGCAGTAGTGACTGATTTGTAT
ATA
>MinSyn_1135|Strength:0.040281838
GCGTGTCGTTTTAGTGAGGATCCTTACCGCTATGGGTAAGATTCGCGGTAGGTCACGACCACTATGCT

TTGTCAAAAGCTAAAAAAGATGATGCTCTACAAAACTGGTCACTATCAGCTTAGGTCCATCAACAAAT
AATCCAAGTAAGCCTTGCTGACGTAAGGGATGACGCACAAGGGCTATGACGTAAGCCATGACGTCTAG
GCTCACTGCTAGGAGGACCGATGTTTTCAACAATTGTCTGCACCTCACATGTAGGCTAAAAATGTCAA
AGATAGGAGGACCGATGCTGATCTTGCCTGCCTATATAAGGTTTTGCTATTCATTGAAAGCAGTAGTG
ACTGATTTGTATATA
>MinSyn_1901|Strength:0.040446364
GCGTGTCGTTTTAGTGAGGTTATGACCCCCGCCGATGACGCGGGACAGGGCTCAACCACGTCTACAAT
GGTACTTGTGTACAGGGCTCACTGCTTCTCTCTGCCGACAGTGGTCCCAAACCTCACATGTAGGCTAT
CAGCTTAGCAAGATCAGAAGATCAAAGGGCTATGATCAGCCACTTGTGTCTCAATGACGTAAGCCATG
ACGTCTACAGGGCTCACTCTATATAAGGTTTTGCTATTCATTGAAAGCAGTAGTGACTGATTTGTATA
TA
>MinSyn_1893|Strength:0.040637912
GCGTGTCGTTTTAGTGAGGAACCACGTCTACAAAGACCTCTACAAAACTGGTACTTGTAAAAATGTCA
AAGATATGGTACTTGTGCTTTGTCAAAAGCTAAAAAAGATGATGCATCTTGCAAGATTGATGAAAAGT
CAAAAACAAAAATCAATTATTATGCCCAATTAGGTTATGACGTAAGCCATGACGTCTAACAAAACTGG
TACTTGTGTACAGGCTATATAAGGTTTTGCTATTCATTGAAAGCAGTAGTGACTGATTTGTATATA
>MinSyn_1593|Strength:0.040845949
GCGTGTCGTTTTAGTGAGGTTATGACCCCCGCCGATGACGCGGGACGGTAGGTCACGACCACTATGCC
CAATTAGATGAGCTAAGCACATACGTCAGCAGGGCTCACTGCTAGGAGGACCGATGTCAGAAGATCAA
AGGGCTATTGTGTACAGGGCTCACTGCTAGGAGCAAGTGGATCGGTAGGTCACGACCACTATATTGCG
ATAAAGGAAAGGGCTATATAAGGTTTTGCTATTCATTGAAAGCAGTAGTGACTGATTTGTATATA
>MinSyn_1438|Strength:0.041506704
GCGTGTCGTTTTAGTGAGGGCTTTGTCAAAAGCTAAAAAAGATGATGCTGCACCTCACATGTAGGAGG
TGGCTCCTACGTAGGCTATCAGCTTAGCAAGACCTCTAATGAGCTAAGCACATACGTCAGAAAACTGG
TACTTGATTGCGATAAAGGAAAGGTACAAAACTGGGTGGGAGCCACCAACGACCACTATGCCCATTTT
CAACAAGCCCCTATATAAGGTTTTGCTATTCATTGAAAGCAGTAGTGACTGATTTGTATATA
>MinSyn_1376|Strength:0.041565253
GCGTGTCGTTTTAGTGAGGATTGCGATAAAGGAAAGGCTTAGCAAGACCTCTACAAAACTGGTACTTA
AGATTGATGAAAAGTCAAAAACAAAAATCAATTATTCCAAATATTTCTTGTGGTTGTCTGCACCTCAC
ATGTAGGCATGACGTAAGCCATGACGTCTAGTAGGCTATCAGCTTAGCAAGACCTCTAAGGTGGCTCC
TACAGACCTCTATATAAGGTTTTGCTATTCATTGAAAGCAGTAGTGACTGATTTGTATATA
>MinSyn_1834|Strength:0.041628952
GCGTGTCGTTTTAGTGAGGATTGCGATAAAGGAAAGGCGCAAAAATGTCAAAGATAGGCTCACTGCTA
GGAGGAAATTTCGGGAAACCTCCTCGGGAGGACCGATGCTGATCTTGCCTTATGACCCCCGCCGATGA
CGCGGGACGACCACTATGCCCATGAGCTAAGCACATACGTCAGGTCACTATCAGCCAAAACTGGTACT
TGTGTACAGCTATATAAGGTTTTGCTATTCATTGAAAGCAGTAGTGACTGATTTGTATATA
>MinSyn_1805|Strength:0.041662376
GCGTGTCGTTTTAGTGAGGTCCATCAACAAATAATCCAAGTAAGACCACGCTTTGTCAAAAGCTAAAA
AAGATGATGCCTGCTAGGAGGACCGATGCTGATCTTTCAGAAGATCAAAGGGCTAGGTACTTGTGTAC
AGGGCTCACTGCTAGGATCGAAAGGACAGTAAAGACCTCTAATGACGTAAGCCATGACGTCTAATTCT
GACGTAAGGGATGACGCACAATTAGGTTGTCTGCACCTCACATGTTTTTCAACAACTGCTAGGAGGAC
CGATGGAAAAAGAAGAGGTCATGTAGGCTATCAGCCTATATAAGGTTTTGCTATTCATTGAAAGCAGT
AGTGACTGATTTGTATATA
>MinSyn_1650|Strength:0.041791062
GCGTGTCGTTTTAGTGAGGGGAAAAAGAAGAGGTTTGCCTGCCTTTGGTGGAGCACGACAGACCACTA
TGCCCAATATGAGCTAAGCACATACGTCAGGCTGATAATTTCGGGAAACCTCCTCGGCCCAATTAGGT
TGTCTGAACCATTATTGCGCGATCAGAAGATCAAAGGGCTATCTGCACCTCACATGTTCACTATCAGC
GCCTTGATGACTATATAAGGTTTTGCTATTCATTGAAAGCAGTAGTGACTGATTTGTATATA
>MinSyn_1913|Strength:0.042714657
GCGTGTCGTTTTAGTGAGGAAGATTGATGAAAAGTCAAAAACAAAAATCAATTATGCCCAATTAGGTT
GTCTGCACCTCATTTTCAACAACTACAAAACTGGTACTTGTGTACAGGGCTCAGAAGATCAAAGGGCT
ACTATCATGAGCTAAGCACATACGTCAGCTCTACAAAACTTCCATCAACAAATAATCCAAGTAAGATG
TAGCTATATAAGGTTTTGCTATTCATTGAAAGCAGTAGTGACTGATTTGTATATA
>MinSyn_1223|Strength:0.04273176

GCGTGTCGTTTTAGTGAGGGCTTTGTCAAAAGCTAAAAAAGATGATGCTTAGGTTGTCTGCACCTCAC
ATGTAGATGAGCTAAGCACATACGTCAGGGACCGATGCTGATTTTTCAACAAGCTTAATTTCGGGAAA
CCTCCTCGGATGCTGATCTTGCCTGCGTGGGAGCCACCACTCGCACACCAGCATGTGTTGATCACCAG
CTCCCTATATAAGGTTTTGCTATTCATTGAAAGCAGTAGTGACTGATTTGTATATA
>MinSyn_1267|Strength:0.042949638
GCGTGTCGTTTTAGTGAGGTCTCTCTGCCGACAGTGGTCCCAAAGCTTAGCAAGACCTCTACAAAACT
GGTACTATTGCGATAAAGGAAAGGATCTTGCCTGCCTTGATATGAGCTAAGCACATACGTCAGAGGAC
CGATGCTGATCTTGCCTGCCTTGATCAGTGGTCCCTCCACACTGCTAGGAGGACCGATGCTGATCTTC
TATATAAGGTTTTGCTATTCATTGAAAGCAGTAGTGACTGATTTGTATATA
>MinSyn_1013|Strength:0.043091941
GCGTGTCGTTTTAGTGAGGCTGACGTAAGGGATGACGCACATACAAAACTGGTACTTGTGTACTCTCT
CTGCCGACAGTGGTCCCAAAATTAGGTTGTCTGATCCTTACCGCTATGGGTAAGATTACAGGCTATAT
AAGGTTTTGCTATTCATTGAAAGCAGTAGTGACTGATTTGTATATA
>MinSyn_1411|Strength:0.043127465
GCGTGTCGTTTTAGTGAGGAACCATTATTGCGGCCCAATTAGGTTGTCCTGACGTAAGGGATGACGCA
CATCGCGGTAGGTCACGACCACTATGGCACACCAGCATGTGTTGATCACCAGCTGTCTGCACCTCACA
TGTAGGCTATATAAGGTTTTGCTATTCATTGAAAGCAGTAGTGACTGATTTGTATATA
>MinSyn_1860|Strength:0.0434633
GCGTGTCGTTTTAGTGAGGTCCATCAACAAATAATCCAAGTAAGTGTGTACAGGGCTCACTTGAAGCA
TCTTCCCAGGGCTCACTGCTAGGAGGACCGATGAGCTAAGCACATACGTCAGTGTGTACAGTGGGAGC
CACCAGAGGACCGATGCTTGAAGATAAGATAATAATGTTGAAGATAAGAGAACCACGTCTACAATGAT
CTATATAAGGTTTTGCTATTCATTGAAAGCAGTAGTGACTGATTTGTATATA
>MinSyn_1711|Strength:0.043684269
GCGTGTCGTTTTAGTGAGGCAGCCACTTGTGTGGAGGACCGATGCTGATCTTGCCTGCCTCACTATCA
GCGCGGTAGGTCACGACCACTATGCCCAATTTTTTCAACAACACTGCTAGGAGGGGAAAAAGAAGAGG
TCGCGGTAGGTCACGACCAATGACGTAAGCCATGACGTCTAAGGCTATCAGCTTAGCAAGACCTATAT
AAGGTTTTGCTATTCATTGAAAGCAGTAGTGACTGATTTGTATATA
>MinSyn_1574|Strength:0.04401541
GCGTGTCGTTTTAGTGAGGCTGACGTAAGGGATGACGCACAGTGTACAGGGCTCACTGCTAGGATCGA
AAGGACAGTATAGGAGGACCGATGCTGATCTTGCGTGGGAGCCACCACTGCTAGGAGGACCGATGGTG
GAGCACGACACCCAATATGACGTAAGCCATGACGTCTAGGACCGATGCTGATCTTGCCTGCCTTGATG
AAGATAAGATAATAATGTTGAAGATAAGAAATTAGGTTGTCTGCACCTCACATGTAGGCTCCATCAAC
AAATAATCCAAGTAAGAGACCTCTACAAAACTGGTACTTGTGTAAGGTGGCTCCTACCTACAAAAGAT
TGATGAAAAGTCAAAAACAAAAATCAATTATATCTTGCCTGCTATATAAGGTTTTGCTATTCATTGAA
AGCAGTAGTGACTGATTTGTATATA
>MinSyn_1401|Strength:0.044221534
GCGTGTCGTTTTAGTGAGGAGGTGGCTCCTACCTTAGCAAGACCTCTACAAAACTGGTACTTTCTCTC
TGCCGACAGTGGTCCCAAAGTCTGCACCTCACATGTAGGCTATCAGCTTATGACGTAAGCCATGACGT
CTAGTATTGCGATAAAGGAAAGGAGGGCACACCAGCATGTGTTGATCACCAGCTCTCACATGTCTATA
TAAGGTTTTGCTATTCATTGAAAGCAGTAGTGACTGATTTGTATATA
>MinSyn_1614|Strength:0.044605993
GCGTGTCGTTTTAGTGAGGCTGACGTAAGGGATGACGCACAGCTCACTGCTAGGAGGACCGATATCCT
TACCGCTATGGGTAAGATTGATGCTGATCTTGCCTGCCGGAAAAAGAAGAGGTCTTGTCTATATAAGG
TTTTGCTATTCATTGAAAGCAGTAGTGACTGATTTGTATATA
>MinSyn_1621|Strength:0.044648
GCGTGTCGTTTTAGTGAGGTCCATCAACAAATAATCCAAGTAAGCTTGATCGAAAGGACAGTAATGCC
CAATTAGGTTGTCTGCACCTCACATAATTTCGGGAAACCTCCTCGCGCAGCCACTTGTGTGTTGAAGC
ATCTTCCGGTTGTCTGCACCTCACATGTACTGACGTAAGGGATGACGCACATTGCCTGCCTATATAAG
GTTTTGCTATTCATTGAAAGCAGTAGTGACTGATTTGTATATA
>MinSyn_1877|Strength:0.044670426
GCGTGTCGTTTTAGTGAGGCAAATATTTCTTGTTCACATGTAGGCTATCAGCTTAGCAAGACCAGCAA
GTGGATACCTCTACAAAACTGGTACTATGAGCTAAGCACATACGTCAGGCGGTAGGTCTTTTCAACAA
GACCGATGCTGATCTTGCAACCACGTCTACAAGCTAGGAGGACCGATGCTGATCTTGCTATATAAGGT
TTTGCTATTCATTGAAAGCAGTAGTGACTGATTTGTATATA

>MinSyn_1587|Strength:0.044797655
GCGTGTCGTTTTAGTGAGGAACCACGTCTACAAGTCACGACCACTTCCATCAACAAATAATCCAAGTA
AGACAGGGCTCACTGCTAGGAGGACCGAGGAAAAAGAAGAGGTTTGTGTACAGGGCTCACATGACGTA
AGCCATGACGTCTATTGTCTGCACCTCACATGTAGTGGGAGCCACCAGACCTCTACACTATATAAGGT
TTTGCTATTCATTGAAAGCAGTAGTGACTGATTTGTATATA
>MinSyn_1095|Strength:0.045045225
GCGTGTCGTTTTAGTGAGGCTGACGTAAGGGATGACGCACAAGGCAGCCACTTGTGTCTATGCCCAAT
TAACCACGTCTACAATTGCCTGCATTGCGATAAAGGAAAGGACCGATGCTGATCTTGCTATATAAGGT
TTTGCTATTCATTGAAAGCAGTAGTGACTGATTTGTATATA
>MinSyn_1077|Strength:0.04522081
GCGTGTCGTTTTAGTGAGGGTGGGAGCCACCAGTTTTCAACAACAGGGCTCACTGCTAGGAATGAGCT
AAGCACATACGTCAGCCTCACATGTAGGCTATCAGCTTAGCAAAACCATTATTGCGTCTTGCCTGCCC
TGACGTAAGGGATGACGCACATTAGGTTGTCTGCACCTCACATGTAGGTCCATCAACAAATAATCCAA
GTAAGGGTACATTGCGATAAAGGAAAGGACAGGGCTCACTGCTAGGAGATCGAAAGGACAGTACTCTA
CATGGTGGAGCACGACACTCACATGTAGGCTATCAGCTCTATATAAGGTTTTGCTATTCATTGAAAGC
AGTAGTGACTGATTTGTATATA
>MinSyn_1582|Strength:0.045494801
GCGTGTCGTTTTAGTGAGGAACCACGTCTACAACTGCTAGGAGGACCGATGCTATCGAAAGGACAGTA
TGGTACTTGTGTAAGGTGGCTCCTACTATCAAAGATTGATGAAAAGTCAAAAACAAAAATCAATTATC
CGATGCTGATCTTGCCTGCCTTGATGATAATGAGCTAAGCACATACGTCAGCTGGCTATATAAGGTTT
TGCTATTCATTGAAAGCAGTAGTGACTGATTTGTATATA
>MinSyn_1767|Strength:0.045549509
GCGTGTCGTTTTAGTGAGGTTATGACCCCCGCCGATGACGCGGGACTGATCTTATGAGCTAAGCACAT
ACGTCAGTCTTGCCTGCCTTGATGATCCATCAACAAATAATCCAAGTAAGGTTTTCAACAACCTCTGG
AAAAAGAAGAGGTGGTAGGTCAATTGCGATAAAGGAAAGGGTAGGCTATCAGCTTACTATATAAGGTT
TTGCTATTCATTGAAAGCAGTAGTGACTGATTTGTATATA
>MinSyn_1085|Strength:0.045650172
GCGTGTCGTTTTAGTGAGGGGAAAAAGAAGAGGTGCTAGGAGGACCGATGACGTAAGCCATGACGTCT
ATATGCCCAATTAGGTTGTCATGAGCTAAGCACATACGTCAGTTAGCAAGACAACCACGTCTACAACC
TCACATGTAGGAAAAATGTCAAAGATACTGCACCTCACATGCAGTGGTCCCTCCACTAGGAGGACCGA
TCTATATAAGGTTTTGCTATTCATTGAAAGCAGTAGTGACTGATTTGTATATA
>MinSyn_1605|Strength:0.045811199
GCGTGTCGTTTTAGTGAGGGGAAAAAGAAGAGGTAGGACCGATGAAGATAAGATAATAATGTTGAAGA
TAAGACCTCTACAAAACTGGTCAGTGGTCCCTCCACAGGATCCTTACCGCTATGGGTAAGATTATTAG
GTTGTCTGATGACGTAAGCCATGACGTCTAAAGACAGCAAGTGGATACCGATGCTATATAAGGTTTTG
CTATTCATTGAAAGCAGTAGTGACTGATTTGTATATA
>MinSyn_1253|Strength:0.045880972
GCGTGTCGTTTTAGTGAGGAAGATTGATGAAAAGTCAAAAACAAAAATCAATTATCTAGGAGGACCGA
TGCTGATCTTGCCATCGAAAGGACAGTACTGATCTTGCCTGCCTTGAATGAGCTAAGCACATACGTCA
GTAGGTTGTCTGCAAACCATTATTGCGACAAAACTGGTACTTGTGTACAGGCTATATAAGGTTTTGCT
ATTCATTGAAAGCAGTAGTGACTGATTTGTATATA
>MinSyn_1740|Strength:0.046086879
GCGTGTCGTTTTAGTGAGGTTTTCAACAAACGACATTGCGATAAAGGAAAGGCCTCACATGTAGGCTA
AAGATTGATGAAAAGTCAAAAACAAAAATCAATTATCTCACATGACGTAAGCCATGACGTCTAGGTCA
CGACCACTATGCCCAATTAGGAAAAAGAAGAGGTTTGTCTGCACCTCACATCTATATAAGGTTTTGCT
ATTCATTGAAAGCAGTAGTGACTGATTTGTATATA
>MinSyn_1063|Strength:0.046194565
GCGTGTCGTTTTAGTGAGGATCCTTACCGCTATGGGTAAGATTTCACATGTAGGCTATCAGCTTAGCA
AGACCTTATGACCCCCGCCGATGACGCGGGAATCTTGCCCTGACGTAAGGGATGACGCACATTGTGTA
CAGGGCTCACTGCGCACACCAGCATGTGTTGATCACCAGCTCTGCACCTCACTATATAAGGTTTTGCT
ATTCATTGAAAGCAGTAGTGACTGATTTGTATATA
>MinSyn_1548|Strength:0.046282293
GCGTGTCGTTTTAGTGAGGAGCAAGTGGATGGTACTTGTGTACAGTCACTATCAGCACTGGTACTAAA
AATGTCAAAGATAGCTTAGCAAGACCTCTACTCAGAAGATCAAAGGGCTACTTGTGTACAGGGCTCAC

TGCATGAGCTAAGCACATACGTCAGTAGGCTATCAGCTTAGCAAGACCCTATATAAGGTTTTGCTATT
CATTGAAAGCAGTAGTGACTGATTTGTATATA
>MinSyn_1193|Strength:0.046791942
GCGTGTCGTTTTAGTGAGGCAGCCACTTGTGTTGCCTGCCTTGATGAGCTAAGCACATACGTCAGCTG
CACCTCACATGTAGGCTATCAGCTTTTCAACAAGAGGACCGATAACCATTATTGCGATGCCCAATTAG
GTTGTCTGCACCTCAATGACGTAAGCCATGACGTCTACATGTAGGCCAAATATTTCTTGTTGCACCTC
ACATGTAGGCTATCAGCTTAGCTCACTATCAGCTGCCCAATTAGGTTGTCTGCACCTCACTTATGACC
CCCGCCGATGACGCGGGAGATCTTGCCTGCCTTGATGAATTGCGATAAAGGAAAGGCACTGCTAGGAG
GACCGATGCTGATCTCTATATAAGGTTTTGCTATTCATTGAAAGCAGTAGTGACTGATTTGTATATA
>MinSyn_1830|Strength:0.04735734
GCGTGTCGTTTTAGTGAGGAATTTCGGGAAACCTCCTCGTCGCGGTAGGTCACGACCACTACAGTGGT
CCCTCCACCACTGCTAGGAGGACCTCAGAAGATCAAAGGGCTAACAGGGCTCACTGCCAGCCACTTGT
GTGCGGTAGGTCACGACATGAGCTAAGCACATACGTCAGAATTACTATATAAGGTTTTGCTATTCATT
GAAAGCAGTAGTGACTGATTTGTATATA
>MinSyn_1047|Strength:0.04762969
GCGTGTCGTTTTAGTGAGGAATTTCGGGAAACCTCCTCGACTGGTACTTGTGTACAGGGCTCACTGCT
AAACCATTATTGCGTGCCTGCCAACCACGTCTACAATCACGACCACTATGCCCAATTACTGACGTAAG
GGATGACGCACAACTTGTGTACAGGGCTTTTTCAACAACTTGCTATATAAGGTTTTGCTATTCATTGA
AAGCAGTAGTGACTGATTTGTATATA
>MinSyn_1520|Strength:0.047672452
GCGTGTCGTTTTAGTGAGGGGAAAAAGAAGAGGTCCTCTACAAAACTGGTACTTGTGTACAGCCACTT
GTGTTATGCCCAATTTGAAGATAAGATAATAATGTTGAAGATAAGACGGTAGGTCACGACCACTGACG
TAAGGGATGACGCACAAAACTGGTACTTGTGAAGCATCTTCCACTATATAAGGTTTTGCTATTCATTG
AAAGCAGTAGTGACTGATTTGTATATA
>MinSyn_1208|Strength:0.047701997
GCGTGTCGTTTTAGTGAGGAACCACGTCTACAAGCTTAGCAATGAGCTAAGCACATACGTCAGATCTC
TCTGCCGACAGTGGTCCCAAATTGATGATACAAATATTTCTTGTCCTCCATCAACAAATAATCCAAGT
AAGAGCAGCCACTTGTGTGGGCTCACTGCTAGGAGGACCGATGCTCTATATAAGGTTTTGCTATTCAT
TGAAAGCAGTAGTGACTGATTTGTATATA
>MinSyn_1935|Strength:0.048057973
GCGTGTCGTTTTAGTGAGGTTTTCAACAAAAGACCTCTACAAAACTGGTACTTAGCAAGTGGATTGCT
CAGTGGTCCCTCCACCTGCTAGGATCCTTACCGCTATGGGTAAGATTCATCGAAAGGACAGTAGGTTG
TCTGCACCTCACATGTACTGACGTAAGGGATGACGCACAAGCTATATAAGGTTTTGCTATTCATTGAA
AGCAGTAGTGACTGATTTGTATATA
>MinSyn_1580|Strength:0.048207255
GCGTGTCGTTTTAGTGAGGATGACGTAAGCCATGACGTCTAGCTAGGAAGGTGGCTCCTACAATTAGG
TTGTCAGAAGATCAAAGGGCTATCTACAAAACTGGTACTTGTGTACAGGGCTATATAAGGTTTTGCTA
TTCATTGAAAGCAGTAGTGACTGATTTGTATATA
>MinSyn_1639|Strength:0.048459816
GCGTGTCGTTTTAGTGAGGTGAAGATAAGATAATAATGTTGAAGATAAGATATGCCCAATTAGGTTGT
CTGCACGTGGGAGCCACCATCACGACCACTATGCCCAATTAGGTGAAGCATCTTCCGACCACTATATG
ACGTAAGCCATGACGTCTAATCTTGCCTGCCTTGATGACTATATAAGGTTTTGCTATTCATTGAAAGC
AGTAGTGACTGATTTGTATATA
>MinSyn_1965|Strength:0.048975389
GCGTGTCGTTTTAGTGAGGGGAAAAAGAAGAGGTAAGACCTCTACAAAACTTCCATCAACAAATAATC
CAAGTAAGGGTTGTCTATGAGCTAAGCACATACGTCAGACCAGCCACTTGTGTTAGCAAGTGGTGGAG
CACGACACAATTAGGTTGTCTGCACCTCACATGTAGGCTATATAAGGTTTTGCTATTCATTGAAAGCA
GTAGTGACTGATTTGTATATA
>MinSyn_1103|Strength:0.049011637
GCGTGTCGTTTTAGTGAGGATGACGTAAGCCATGACGTCTACTACAAATGGTGGAGCACGACAGCTTA
GCGCACACCAGCATGTGTTGATCACCAGCTAGGAGGACCGATGCTGATCCTATATAAGGTTTTGCTAT
TCATTGAAAGCAGTAGTGACTGATTTGTATATA
>MinSyn_1374|Strength:0.049039178
GCGTGTCGTTTTAGTGAGGTCACTATCAGCGGTCAATGACGTAAGCCATGACGTCTATCAGCTTAGCA

AGACGCACACCAGCATGTGTTGATCACCAGCTGAGGACCGATGCTGATCTTGCCTCTATATAAGGTTT
TGCTATTCATTGAAAGCAGTAGTGACTGATTTGTATATA
>MinSyn_1043|Strength:0.049100844
GCGTGTCGTTTTAGTGAGGCAGCCACTTGTGTCACATGTAGGCTATCAGGCACACCAGCATGTGTTGA
TCACCAGCTACATGTAGGCTATCAGCTTAGCATGAGCTAAGCACATACGTCAGTATCAGCTTAGCAAG
AAACCATTATTGCGCATGTAGGCTATCAGCTTAGCCTATATAAGGTTTTGCTATTCATTGAAAGCAGT
AGTGACTGATTTGTATATA
>MinSyn_1697|Strength:0.049368436
GCGTGTCGTTTTAGTGAGGTTTTCAACAACTGTGGGAGCCACCAACCTCACATTCAGAAGATCAAAGG
GCTATTGTCTGCACCTCACATGTATCACTATCAGCGGAGGACCGATGCTGATCTTGCCTGACGTAAGG
GATGACGCACAGCTAGGAGGACCGATGCTGATCCTATATAAGGTTTTGCTATTCATTGAAAGCAGTAG
TGACTGATTTGTATATA
>MinSyn_1651|Strength:0.049699898
GCGTGTCGTTTTAGTGAGGAAAAATGTCAAAGATAGACCGATGCTGATCTTGCCTGCATTGCGATAAA
GGAAAGGCAGCTTAGCAAGACCTCTACAAAACTATCCTTACCGCTATGGGTAAGATTTGCTAATGAGC
TAAGCACATACGTCAGCTGCTAGGAGGACCGATCTATATAAGGTTTTGCTATTCATTGAAAGCAGTAG
TGACTGATTTGTATATA
>MinSyn_1869|Strength:0.049778946
GCGTGTCGTTTTAGTGAGGCTGACGTAAGGGATGACGCACATAGGTTGTCTGCACCTCACATGTAGTC
ACTATCAGCTTGTGTACTCTCTCTGCCGACAGTGGTCCCAAACCTCTACAATGACGTAAGCCATGACG
TCTACCACTATGCCCAATTAGGTTGTCTGCACCTAACCACGTCTACAAGCGGTAGGTCACGACCACTA
TGCCCAAATATTTCTTGTTTAGCAAGACCTCTACAAAACTTGAAGCATCTTCCAGGACCGATGCTGAT
CTTGCCTGCTATATAAGGTTTTGCTATTCATTGAAAGCAGTAGTGACTGATTTGTATATA
>MinSyn_1782|Strength:0.049899858
GCGTGTCGTTTTAGTGAGGCAGCCACTTGTGTACCTCTACTGAAGCATCTTCCCTTAGCAAGACCTCT
ACATGAAGATAAGATAATAATGTTGAAGATAAGACTATGCCCAATTAGGTTATGACGTAAGCCATGAC
GTCTAAGGCTTCAGAAGATCAAAGGGCTAACAGCTATATAAGGTTTTGCTATTCATTGAAAGCAGTAG
TGACTGATTTGTATATA
>MinSyn_1619|Strength:0.050130635
GCGTGTCGTTTTAGTGAGGATGAGCTAAGCACATACGTCAGATTAGGTTGTCTGCACCTCACATTTTT
CAACAACCACTATGCCCAATTAGGTTGTCTGCACCCAAATATTTCTTGTGCAAGACCTCTACAAAACT
ATCGAAAGGACAGTATGCCCAATTAGGTTGTCTATATAAGGTTTTGCTATTCATTGAAAGCAGTAGTG
ACTGATTTGTATATA
>MinSyn_1811|Strength:0.050188618
GCGTGTCGTTTTAGTGAGGTTATGACCCCCGCCGATGACGCGGGAACCACTATGCCCAATTAGGTAGG
TGGCTCCTACACTATGATGACGTAAGCCATGACGTCTAGATGCTGATCTTGCCTGAACCATTATTGCG
GTAGGCTATCAGCTTAGCAAGACCTCTACACTATATAAGGTTTTGCTATTCATTGAAAGCAGTAGTGA
CTGATTTGTATATA
>MinSyn_1011|Strength:0.050455516
GCGTGTCGTTTTAGTGAGGCAGTGGTCCCTCCACTGATCTTGCCTGCCTTGATGAATGAGCTAAGCAC
ATACGTCAGTGCTAGGAGGACCGATGCTGATCTTGCCTGAAGATTGATGAAAAGTCAAAAACAAAAAT
CAATTATAGGACCGATGCTGATCTTGCCTGCTATATAAGGTTTTGCTATTCATTGAAAGCAGTAGTGA
CTGATTTGTATATA
>MinSyn_1846|Strength:0.050741623
GCGTGTCGTTTTAGTGAGGTTTTCAACAACTGGTACTTGTGTACAGGGCTCACAAATATTTCTTGTTA
GGTTGTCTGCACCTCAACCACGTCTACAACTCTACATGACGTAAGCCATGACGTCTAGTTGTTCTCTC
TGCCGACAGTGGTCCCAAATACAGGGCTCCTATATAAGGTTTTGCTATTCATTGAAAGCAGTAGTGAC
TGATTTGTATATA
>MinSyn_1049|Strength:0.050789693
GCGTGTCGTTTTAGTGAGGATCCTTACCGCTATGGGTAAGATTATGGTGGAGCACGACACGACCACTA
TGCCCAATTAGAGGTGGCTCCTACCTCTATGAGCTAAGCACATACGTCAGGGTAGGTCACGACCACTA
TAACCATTATTGCGGGAGGACCGATGCTCTATATAAGGTTTTGCTATTCATTGAAAGCAGTAGTGACT
GATTTGTATATA
>MinSyn_1229|Strength:0.050810391

GCGTGTCGTTTTAGTGAGGAGGTGGCTCCTACGCAAGACCTCTACAAAACTGGTACTTGGCACACCAG
CATGTGTTGATCACCAGCTACAAAAAAAAATGTCAAAGATATGCACCTCACATGTAGGCCTGACGTAA
GGGATGACGCACAACTGGTACTTGTGTACTATATAAGGTTTTGCTATTCATTGAAAGCAGTAGTGACT
GATTTGTATATA
>MinSyn_1778|Strength:0.050812244
GCGTGTCGTTTTAGTGAGGCAGTGGTCCCTCCACCTATGCCCAATTAGGTTGTCTGCAATGAGCTAAG
CACATACGTCAGGCTCACTGAAAAATGTCAAAGATAAGCAAGACTGGTGGAGCACGACAGACCTCTAC
AAAACTGGTACTGGAAAAGAAGAGGTGCCTATATAAGGTTTTGCTATTCATTGAAAGCAGTAGTGAC
TGATTTGTATATA
>MinSyn_1056|Strength:0.051147946
GCGTGTCGTTTTAGTGAGGTTTTCAACAAACCGATGCTGATCTTGCCTGCCTTGCTTTGTCAAAAGCT
AAAAAAGATGATGCAGGTCACGACCACTATGCCCAATTAGGGGAAAAAGAAGAGGTAATGACGTAAGC
CATGACGTCTAATGCCCAATTAGGTCTATATAAGGTTTTGCTATTCATTGAAAGCAGTAGTGACTGAT
TTGTATATA
>MinSyn_1241|Strength:0.051335041
GCGTGTCGTTTTAGTGAGGAGCAAGTGGATCATGTAGGCTATCAGCCAAATATTTCTTGTTCTGCACA
GCCACTTGTGTCATCCTTACCGCTATGGGTAAGATTTGTACAGGGCTCACTGCTACTGACGTAAGGGA
TGACGCACACAAGACCTCTACAAAACCTATATAAGGTTTTGCTATTCATTGAAAGCAGTAGTGACTGA
TTTGTATATA
>MinSyn_1937|Strength:0.052045891
GCGTGTCGTTTTAGTGAGGTGAAGCATCTTCCTACAAAACTGGTACTTGTGTACAGGTGAAGATAAGA
TAATAATGTTGAAGATAAGACTTAGCATGACGTAAGCCATGACGTCTACTATGGAAAAAGAAGAGGTA
CTGCTAGGAGGACCGATGCTGATCTATATAAGGTTTTGCTATTCATTGAAAGCAGTAGTGACTGATTT
GTATATA
>MinSyn_1831|Strength:0.052197764
GCGTGTCGTTTTAGTGAGGTCCATCAACAAATAATCCAAGTAAGACTGCTAGGAGGACCGATGCTGAT
CTATGAGCTAAGCACATACGTCAGACATGTAGGCTATGTGGGAGCCACCAACTGCTAGGAGGACCGAT
TTTCAACAACTCACTGCTAGGACTATATAAGGTTTTGCTATTCATTGAAAGCAGTAGTGACTGATTTG
TATATA
>MinSyn_1698|Strength:0.052354216
GCGTGTCGTTTTAGTGAGGAACCATTATTGCGAGGAGGACCGATGCTGATCTTAGCAAGTGGATGGGC
TCACTGCTAATGAGCTAAGCACATACGTCAGAGCCTGACGTAAGGGATGACGCACACGGTAGGTCACG
ACCACTATGCCCTTTTCAACAAGCATTGCGATAAAGGAAAGGGTAGGTCCTATATAAGGTTTTGCTAT
TCATTGAAAGCAGTAGTGACTGATTTGTATATA
>MinSyn_1982|Strength:0.05270437
GCGTGTCGTTTTAGTGAGGATTGCGATAAAGGAAAGGACTGGTACTTGTGTACAGGGCTCACTGCTTT
TCAACAACCAAATATTTCTTGTCCCAATTAGGTTGTCTGCACCTCACATGTGAAGCATCTTCCACTGA
CGTAAGGGATGACGCACAGCCTATATAAGGTTTTGCTATTCATTGAAAGCAGTAGTGACTGATTTGTA
TATA
>MinSyn_1377|Strength:0.052716759
GCGTGTCGTTTTAGTGAGGTGAAGCATCTTCCTGATCTTGTTTTCAACAACTGAGCACACCAGCATGT
GTTGATCACCAGCTACCTCACATGTAGGCTATCAGCTCTGACGTAAGGGATGACGCACATATGCCCAA
TTAGGTTGTCTGCACCTCACTATATAAGGTTTTGCTATTCATTGAAAGCAGTAGTGACTGATTTGTAT
ATA
>MinSyn_1178|Strength:0.052783996
GCGTGTCGTTTTAGTGAGGGGAAAAAGAAGAGGTCTCACATGTAGGCTATCAGCTTGTGGGAGCCACC
AACATGAGCTAAGCACATACGTCAGGGTTGTCTGCACCTCACACAAATATTTCTTGTGTTGTCTGCAC
CTCACATGTAGGCTATCAGCTATATAAGGTTTTGCTATTCATTGAAAGCAGTAGTGACTGATTTGTAT
ATA
>MinSyn_1101|Strength:0.053164342
GCGTGTCGTTTTAGTGAGGGGAAAAAGAAGAGGTTAGGCTATCAAACCACGTCTACAAACCGATGCTG
ATCTTGCCTGCCTTGATAATTTCGGGAAACCTCCTCGGTCTGCACCTCTGACGTAAGGGATGACGCAC
ATGCTAGGAGGACCGATGCTATATAAGGTTTTGCTATTCATTGAAAGCAGTAGTGACTGATTTGTATA
TA

>MinSyn_1286|Strength:0.053267457
GCGTGTCGTTTTAGTGAGGCAGCCACTTGTGTTCACGCAAATATTTCTTGTCTGCATCCTTACCGCTA
TGGGTAAGATTACTTGTGTACAGGGCTCACTGCTAGGAGATGACGTAAGCCATGACGTCTATAGGTTG
AAGCATCTTCCTCTACAACTATATAAGGTTTTGCTATTCATTGAAAGCAGTAGTGACTGATTTGTATA
TA
>MinSyn_1327|Strength:0.053671707
GCGTGTCGTTTTAGTGAGGATGAGCTAAGCACATACGTCAGCGCGGTAGGTCACGACCAGCAAGTGGA
TTTAGCAAGACCTCTTTATGACCCCCGCCGATGACGCGGGAAGGCTATCAGCTTAGTGGGAGCCACCA
CTCACTGCTAGGAGGACCTATATAAGGTTTTGCTATTCATTGAAAGCAGTAGTGACTGATTTGTATAT
A
>MinSyn_1295|Strength:0.053763351
GCGTGTCGTTTTAGTGAGGATCCTTACCGCTATGGGTAAGATTAAAACTGGTACTTGTGTAGCAAGTG
GATCCTGCCTTGAAACCACGTCTACAACTTGATGAATGACGTAAGCCATGACGTCTAGTCACGACCAC
TATGCCCAATTAGGTCTATATAAGGTTTTGCTATTCATTGAAAGCAGTAGTGACTGATTTGTATATA
>MinSyn_1370|Strength:0.053847528
GCGTGTCGTTTTAGTGAGGATGAGCTAAGCACATACGTCAGCTGGTACTTGTGTACAGGGCTCACTGC
TAAACCATTATTGCGACTATGCCCAATTAGGTTGTCTCTGACGTAAGGGATGACGCACAGGCTCACTG
TCCATCAACAAATAATCCAAGTAAGGCAAGAAACCACGTCTACAATTGCCTTGAAGATAAGATAATAA
TGTTGAAGATAAGATCACGACCCAAATATTTCTTGTCACTATATAAGGTTTTGCTATTCATTGAAAGC
AGTAGTGACTGATTTGTATATA
>MinSyn_1452|Strength:0.053867002
GCGTGTCGTTTTAGTGAGGTGAAGATAAGATAATAATGTTGAAGATAAGAACTATGCCCAATTAGGTT
GTCTGCACTGGTGGAGCACGACATTGCCTGCCTATGACGTAAGCCATGACGTCTAGGTCACGACCACT
ATGCCCAATTAGGTTCTATATAAGGTTTTGCTATTCATTGAAAGCAGTAGTGACTGATTTGTATATA
>MinSyn_1959|Strength:0.05397967
GCGTGTCGTTTTAGTGAGGAACCATTATTGCGCGATGCTGATGAGCTAAGCACATACGTCAGGTACTT
GTGTACAGTCTCTCTGCCGACAGTGGTCCCAAAGGTAGGTCACGACCAAGGTGGCTCCTACTCGCGGT
AGGTCACGACCACTATCTATATAAGGTTTTGCTATTCATTGAAAGCAGTAGTGACTGATTTGTATATA
>MinSyn_1012|Strength:0.054010338
GCGTGTCGTTTTAGTGAGGCTGACGTAAGGGATGACGCACACCACTATGCCCAATTAGGTTGTCTGCA
CAACCATTATTGCGAGGCTATCAGCTTAGCAAGACCTCCTATATAAGGTTTTGCTATTCATTGAAAGC
AGTAGTGACTGATTTGTATATA
>MinSyn_1536|Strength:0.054104345
GCGTGTCGTTTTAGTGAGGGCACACCAGCATGTGTTGATCACCAGCTCCACAAGATTGATGAAAAGTC
AAAAACAAAAATCAATTATTTCTCTCTGCCGACAGTGGTCCCAAAGTAGGCAGTGGTCCCTCCACTGT
GTACACAAATATTTCTTGTACTTGTGTACAGGGCTTTTCAACAAGCTGACGTAAGGGATGACGCACAA
ATTAGGTTATGACGTAAGCCATGACGTCTAACTTGTGTACAGGGCTCACTGCTAGGAGAGCAAGTGGA
TTGGTACTTGTGTACAGCTATATAAGGTTTTGCTATTCATTGAAAGCAGTAGTGACTGATTTGTATAT
A
>MinSyn_1752|Strength:0.054240596
GCGTGTCGTTTTAGTGAGGTGGTGGAGCACGACACTATGCCCAATTAGGTTGTCTGCACCTATGAGCT
AAGCACATACGTCAGGACCACTATGCCCAATTAGGTTGTCGGAAAAAGAAGAGGTGGTACTTGTGTAC
AGGGCTCACTGCTCTATATAAGGTTTTGCTATTCATTGAAAGCAGTAGTGACTGATTTGTATATA
>MinSyn_1708|Strength:0.054523992
GCGTGTCGTTTTAGTGAGGCTGACGTAAGGGATGACGCACAGCTAGGAGGACCGATGCTGATCTTGCC
TCACTATCAGCTATCAGCTTAGCAAGACCTCTACAAACTATATAAGGTTTTGCTATTCATTGAAAGCA
GTAGTGACTGATTTGTATATA
>MinSyn_1762|Strength:0.05456669
GCGTGTCGTTTTAGTGAGGCAAATATTTCTTGTTGTATGAGCTAAGCACATACGTCAGGGTTCTCTCT
GCCGACAGTGGTCCCAAAAGCTTAGCATTTTCAACAAACAAAACTGGTACTTGTGTACAGGGGGAAAA
AGAAGAGGTCTATGCTATATAAGGTTTTGCTATTCATTGAAAGCAGTAGTGACTGATTTGTATATA
>MinSyn_1812|Strength:0.054746863
GCGTGTCGTTTTAGTGAGGTCCATCAACAAATAATCCAAGTAAGCCTCACATGTAGCAGTGGTCCCTC
CACGCCTGATGACGTAAGCCATGACGTCTATGCTGATCTTGCCTGCCTTCTCTCTGCCGACAGTGGTC

CCAAATGATCTTGCTATATAAGGTTTTGCTATTCATTGAAAGCAGTAGTGACTGATTTGTATATA
>MinSyn_1688|Strength:0.054748649
GCGTGTCGTTTTAGTGAGGTTTTCAACAAACCGCAAATATTTCTTGTGCAGTGGTCCCTCCACCTGCT
AGGAGGACCATGACGTAAGCCATGACGTCTACCAATTAGGTTGTCTGGGAAAAAGAAGAGGTTATCAG
CTTAGCAAGACCCTATATAAGGTTTTGCTATTCATTGAAAGCAGTAGTGACTGATTTGTATATA
>MinSyn_1631|Strength:0.054784057
GCGTGTCGTTTTAGTGAGGCAAATATTTCTTGTACTGCTAGGAGGACCGATGCTAACCACGTCTACAA
AAACAATTTCGGGAAACCTCCTCGGACCACTATGCCCAATTAGGATGAGCTAAGCACATACGTCAGCT
CACTGCTAGGAGCTATATAAGGTTTTGCTATTCATTGAAAGCAGTAGTGACTGATTTGTATATA
>MinSyn_1073|Strength:0.054796805
GCGTGTCGTTTTAGTGAGGCAAATATTTCTTGTATGGTGGAGCACGACAGGCTCACTGCTAGGAGGAC
CGATATGAGCTAAGCACATACGTCAGGACATCGAAAGGACAGTACAATTAGGTTGTCTGCACCTCACA
TGTATGACGTAAGCCATGACGTCTAAACTGGTACTTGTGTAATTTCGGGAAACCTCCTCGACTGGTAC
TTGTGTACAGGATTGCGATAAAGGAAAGGGCCCAATAAAAATGTCAAAGATAGGTACTTGAGGTGGCT
CCTACCCTATATAAGGTTTTGCTATTCATTGAAAGCAGTAGTGACTGATTTGTATATA
>MinSyn_1575|Strength:0.054811898
GCGTGTCGTTTTAGTGAGGATTGCGATAAAGGAAAGGTCTTGCCTGCATGACGTAAGCCATGACGTCT
AGCTGGTGGAGCACGACATAGCAAGACCTCTACAAAACTGGTACTAACCACGTCTACAAACAAATATT
TCTTGTATGCCCAATTATGAGCTAAGCACATACGTCAGCAATTAGGTTGTCTGCACCTCACATTCTCT
CTGCCGACAGTGGTCCCAAAGCTATCAGCTTAGCAAGACCAATTTCGGGAAACCTCCTCGTCAGCTTA
GCAAGACCTCTACAAAACTGGGAAAAAGAAGAGGTTGCACCTCACATGTAGGCTCTATATAAGGTTTT
GCTATTCATTGAAAGCAGTAGTGACTGATTTGTATATA
>MinSyn_1916|Strength:0.054906116
GCGTGTCGTTTTAGTGAGGCAGTGGTCCCTCCACCTGCCTAACCATTATTGCGTACAGGGCTCACTGC
TAGGAGGATCGAAAGGACAGTAAGGCTATCAGATGACGTAAGCCATGACGTCTAATGTAGGCTATCAG
CTTAGCAAGACCTATATAAGGTTTTGCTATTCATTGAAAGCAGTAGTGACTGATTTGTATATA
>MinSyn_1800|Strength:0.055130407
GCGTGTCGTTTTAGTGAGGTTTTCAACAAGTTGTCTGCACCTCACATGTATGGTGGAGCACGACATCT
GCACCTCACATCCTTACCGCTATGGGTAAGATTATGATATGAGCTAAGCACATACGTCAGAGACCTCT
ACAAAACTGGTCTATATAAGGTTTTGCTATTCATTGAAAGCAGTAGTGACTGATTTGTATATA
>MinSyn_1824|Strength:0.055325315
GCGTGTCGTTTTAGTGAGGGGAAAAAGAAGAGGTTACAAAACTGGTACTTGTGTACAGGGCTCAATGA
CGTAAGCCATGACGTCTAGCTCACTGCTAGGAGGTTATGACCCCCGCCGATGACGCGGGATTAGCAAG
TGGATACAAACTATATAAGGTTTTGCTATTCATTGAAAGCAGTAGTGACTGATTTGTATATA
>MinSyn_1216|Strength:0.055531738
GCGTGTCGTTTTAGTGAGGTTTTCAACAAAAGACCTCTACAAAACTTTATGACCCCCGCCGATGACGC
GGGAACAAAACTGGTACTTGTGTACATCACTATCAGCTCTACAAATGACGTAAGCCATGACGTCTACT
ATGCCCAATCTATATAAGGTTTTGCTATTCATTGAAAGCAGTAGTGACTGATTTGTATATA
>MinSyn_1512|Strength:0.056327692
GCGTGTCGTTTTAGTGAGGTCCATCAACAAATAATCCAAGTAAGCACTGCTAGGAGGACCGATGCATG
AGCTAAGCACATACGTCAGTGCACCTCACAGGTGGCTCCTACACCAAATATTTCTTGTTTAGGTTGTC
TGCACCTCTATATAAGGTTTTGCTATTCATTGAAAGCAGTAGTGACTGATTTGTATATA
>MinSyn_1827|Strength:0.057051082
GCGTGTCGTTTTAGTGAGGATGACGTAAGCCATGACGTCTAAGCTTAGCAAGACCTTTTCAACAAGGA
CCTCTCTCTGCCGACAGTGGTCCCAAACCTCTACCTATATAAGGTTTTGCTATTCATTGAAAGCAGTA
GTGACTGATTTGTATATA
>MinSyn_1338|Strength:0.057923647
GCGTGTCGTTTTAGTGAGGTGAAGCATCTTCCCTGCTTTGTCAAAAGCTAAAAAAGATGATGCCAAAA
CTGGTCTCTCTGCCGACAGTGGTCCCAAACCTGCCTTGATGAATGAGCTAAGCACATACGTCAGCCTC
TCTATATAAGGTTTTGCTATTCATTGAAAGCAGTAGTGACTGATTTGTATATA
>MinSyn_1633|Strength:0.059095224
GCGTGTCGTTTTAGTGAGGGGAAAAAGAAGAGGTTGCCTTGATTCAGAAGATCAAAGGGCTATGCACC
TCACATGCTGACGTAAGGGATGACGCACAAGGCTATCAGCTTAGCAGCAAGTGGATTCACTGCTCTAT
ATAAGGTTTTGCTATTCATTGAAAGCAGTAGTGACTGATTTGTATATA

>MinSyn_1367|Strength:0.059201853
GCGTGTCGTTTTAGTGAGGATTGCGATAAAGGAAAGGACTGGTACTTGTGTACAATCCTTACCGCTAT
GGGTAAGATTCCAATTAGGTTGTCTGCACCTCATGAGCTAAGCACATACGTCAGGACCTCTACAACTA
TATAAGGTTTTGCTATTCATTGAAAGCAGTAGTGACTGATTTGTATATA
>MinSyn_1691|Strength:0.059757227
GCGTGTCGTTTTAGTGAGGATCGAAAGGACAGTATGATCTTGCCTGCCTTAAAAATGTCAAAGATAGT
ACTTGTGTACAGGGCTCACTCTGACGTAAGGGATGACGCACAAGCCAGCCACTTGTGTGTACACTATA
TAAGGTTTTGCTATTCATTGAAAGCAGTAGTGACTGATTTGTATATA
>MinSyn_1864|Strength:0.060126627
GCGTGTCGTTTTAGTGAGGTGAAGATAAGATAATAATGTTGAAGATAAGAAGGAGGACCGATGTGGTG
GAGCACGACAAGCAAGACCTCTACAAAACTGGTACTATGACGTAAGCCATGACGTCTACTCACTATAT
AAGGTTTTGCTATTCATTGAAAGCAGTAGTGACTGATTTGTATATA
>MinSyn_1176|Strength:0.060278119
GCGTGTCGTTTTAGTGAGGAATTTCGGGAAACCTCCTCGAGACCTCTACAAAAGCAAGTGGATGCTGA
TCTTGCCTGCCTTGATGAAACCACGTCTACAAGCCTTATGACGTAAGCCATGACGTCTAAGCTATATA
AGGTTTTGCTATTCATTGAAAGCAGTAGTGACTGATTTGTATATA
>MinSyn_1224|Strength:0.061166366
GCGTGTCGTTTTAGTGAGGCAGCCACTTGTGTCCAATTAGGTTGTCTGCACCATGACGTAAGCCATGA
CGTCTACTCACTGCTAGGAGGACCTCTCTCTGCCGACAGTGGTCCCAAAGCTTAGCAAGCTATATAAG
GTTTTGCTATTCATTGAAAGCAGTAGTGACTGATTTGTATATA
>MinSyn_1375|Strength:0.061540366
GCGTGTCGTTTTAGTGAGGAGGTGGCTCCTACTCACTGCTAGGAGGACCGATGTGAAGCATCTTCCGG
TCGCTTTGTCAAAAGCTAAAAAAGATGATGCCTATGAGCTAAGCACATACGTCAGGCCTATATAAGGT
TTTGCTATTCATTGAAAGCAGTAGTGACTGATTTGTATATA
>MinSyn_1238|Strength:0.06180761
GCGTGTCGTTTTAGTGAGGAGCAAGTGGATAAAACTGGTACTTGTGTACAGGGCTAACCACGTCTACA
ATCTATCTCTCTGCCGACAGTGGTCCCAAAGCTATCAGCTTAGCAAGACCTCTACATCAGAAGATCAA
AGGGCTACTATTGCGATAAAGGAAAGGTTGTCTGCACCTCACATGGTGGGAGCCACCACAAAACTGGT
ACTTGTGTACAGGGCTCATGACGTAAGCCATGACGTCTACCAATTAGGTTGTCTGCACCTCACCTGAC
GTAAGGGATGACGCACAAACTGGGCACACCAGCATGTGTTGATCACCAGCTGATCTTGCCTGCCTTGA
TGACTATATAAGGTTTTGCTATTCATTGAAAGCAGTAGTGACTGATTTGTATATA
>MinSyn_1207|Strength:0.062742407
GCGTGTCGTTTTAGTGAGGTCTCTCTGCCGACAGTGGTCCCAAAAAACTGGTACTTGTGTACAGGGCT
CACTGATGAGCTAAGCACATACGTCAGCCGATGCTGATCTTGCCTGCCTTGATCTATATAAGGTTTTG
CTATTCATTGAAAGCAGTAGTGACTGATTTGTATATA
>MinSyn_1803|Strength:0.062827629
GCGTGTCGTTTTAGTGAGGTTATGACCCCCGCCGATGACGCGGGATTGCCTGCCTTATGACGTAAGCC
ATGACGTCTAACTTGTGTACAGGGCTCAGGAAAAAGAAGAGGTACTGCTAGGAGCTATATAAGGTTTT
GCTATTCATTGAAAGCAGTAGTGACTGATTTGTATATA
>MinSyn_1562|Strength:0.063200827
GCGTGTCGTTTTAGTGAGGCTGACGTAAGGGATGACGCACATAGGCTATCATTATGACCCCCGCCGAT
GACGCGGGACGATGCTGATCTTGCCTGCCAACCACGTCTACAATTAGGTTGTCTGCACCTCATGACGT
AAGCCATGACGTCTACCAATTAGGTTGTTTTCAACAAATGTAGGCTATCATCAGAAGATCAAAGGGCT
ATACAGGGCTCACTGCTAGGAGGATTGCGATAAAGGAAAGGGTCTGCACCTCACATGTAGGCTCTATA
TAAGGTTTTGCTATTCATTGAAAGCAGTAGTGACTGATTTGTATATA
>MinSyn_1036|Strength:0.06327588
GCGTGTCGTTTTAGTGAGGAACCACGTCTACAACAAGACCTGACGTAAGGGATGACGCACACTGATCT
TGCCTGATCCTTACCGCTATGGGTAAGATTCTCTACAAAACTGGTACTTGTGTCTATATAAGGTTTTG
CTATTCATTGAAAGCAGTAGTGACTGATTTGTATATA
>MinSyn_1618|Strength:0.063541768
GCGTGTCGTTTTAGTGAGGGCACACCAGCATGTGTTGATCACCAGCTCTGATCTTGCCTGCCTTCTGA
CGTAAGGGATGACGCACATACAGGGCTCAACCATTATTGCGTACAGGGCTCACTATATAAGGTTTTGC
TATTCATTGAAAGCAGTAGTGACTGATTTGTATATA
>MinSyn_1761|Strength:0.063678396

GCGTGTCGTTTTAGTGAGGGCACACCAGCATGTGTTGATCACCAGCTCCTCTACAAAAATTGCGATAA
AGGAAAGGGGTCACGACCACTATGCCCTGACGTAAGGGATGACGCACAGCACCTATATAAGGTTTTGC
TATTCATTGAAAGCAGTAGTGACTGATTTGTATATA
>MinSyn_1330|Strength:0.06389896
GCGTGTCGTTTTAGTGAGGCAAATATTTCTTGTGCCTGCCTTAAGATTGATGAAAAGTCAAAAACAAA
AATCAATTATATTAGGATGACGTAAGCCATGACGTCTAGTCAAACCACGTCTACAAGATGCTGATGAG
CTAAGCACATACGTCAGCTTTTTCAACAAGAGCACACCAGCATGTGTTGATCACCAGCTCACTATGGG
AAAAAGAAGAGGTCTCTACAACTATATAAGGTTTTGCTATTCATTGAAAGCAGTAGTGACTGATTTGT
ATATA
>MinSyn_1467|Strength:0.064227164
GCGTGTCGTTTTAGTGAGGATCCTTACCGCTATGGGTAAGATTTGTCTGCATGAAGCATCTTCCGGTA
CTTGTGTACAGGGCTCACTGCTAGCTGACGTAAGGGATGACGCACACTAGCTATATAAGGTTTTGCTA
TTCATTGAAAGCAGTAGTGACTGATTTGTATATA
>MinSyn_1287|Strength:0.064876152
GCGTGTCGTTTTAGTGAGGAAAAATGTCAAAGATAACTATGCCCAATTAGGTTGTCTGCACCTCAATG
AGCTAAGCACATACGTCAGTGCTAGGAGGACCGATGCTGATCTTGCCCTATATAAGGTTTTGCTATTC
ATTGAAAGCAGTAGTGACTGATTTGTATATA
>MinSyn_1081|Strength:0.064877495
GCGTGTCGTTTTAGTGAGGAGGTGGCTCCTACCACTGCTAGGAGGACCGATGCGCACACCAGCATGTG
TTGATCACCAGCTGGTACTTGTGTACCTGACGTAAGGGATGACGCACACCTATATAAGGTTTTGCTAT
TCATTGAAAGCAGTAGTGACTGATTTGTATATA
>MinSyn_1755|Strength:0.06499653
GCGTGTCGTTTTAGTGAGGTTTTCAACAAGGACAAATATTTCTTGTTGCCTGCCTTGATGATAGCAAG
TGGATTGATCTTTCAGAAGATCAAAGGGCTAGGTCACGACCACTATGCCCAAGCACACCAGCATGTGT
TGATCACCAGCTTACAGGGCTCACTGCTAGATGAGCTAAGCACATACGTCAGCTGATCTTGCCTGCCT
TGATGCTGACGTAAGGGATGACGCACACAAGACCTCTACAAATCACTATCAGCTTCCATCAACAAATA
ATCCAAGTAAGAGCTATATAAGGTTTTGCTATTCATTGAAAGCAGTAGTGACTGATTTGTATATA
>MinSyn_1844|Strength:0.065012808
GCGTGTCGTTTTAGTGAGGAACCATTATTGCGGCTTAGCAAGACCTCTACAAAAATGAGCTAAGCACA
TACGTCAGGACCGATGTCAGAAGATCAAAGGGCTACACTGCTAGGAGGCTATATAAGGTTTTGCTATT
CATTGAAAGCAGTAGTGACTGATTTGTATATA
>MinSyn_1859|Strength:0.065200022
GCGTGTCGTTTTAGTGAGGTCACTATCAGCACATCTGACGTAAGGGATGACGCACATAATGACGTAAG
CCATGACGTCTAGGTCACGACTCAGAAGATCAAAGGGCTATCGCGGTAGGTCACGACCACTATATCGA
AAGGACAGTAGCCTGTGGTGGAGCACGACAATGCTGATCTTGCATCCTTACCGCTATGGGTAAGATTA
GGTTGTCTGCACCTCACATGTGGGAGCCACCAGTGTACAGGGCACACCAGCATGTGTTGATCACCAGC
TCTATCAGCTTAGCAAGACCTCTATATAAGGTTTTGCTATTCATTGAAAGCAGTAGTGACTGATTTGT
ATATA
>MinSyn_1737|Strength:0.065325978
GCGTGTCGTTTTAGTGAGGTTTTCAACAAGGTACTTGTGTACAGGGCTCACTGCTCAGCCACTTGTGT
TATGCCCAATTAGGTTGTCTGCACCTCACATGAGCTAAGCACATACGTCAGCTACAAAACTGGTACTT
GTGTACAGGGCATGACGTAAGCCATGACGTCTACCTGACGTAAGGGATGACGCACAGCTGATCTTGCC
ATTGCGATAAAGGAAAGGTACAAAACTGGTACTTTCCATCAACAAATAATCCAAGTAAGCGGTAGGTC
TGGTGGAGCACGACACAATTACTATATAAGGTTTTGCTATTCATTGAAAGCAGTAGTGACTGATTTGT
ATATA
>MinSyn_1906|Strength:0.065600057
GCGTGTCGTTTTAGTGAGGAGGTGGCTCCTACTCGCGGTAGGTCATTATGACCCCCGCCGATGACGCG
GGAACCGATCTGACGTAAGGGATGACGCACATGCCTGCCTTGATGATCTATATAAGGTTTTGCTATTC
ATTGAAAGCAGTAGTGACTGATTTGTATATA
>MinSyn_1757|Strength:0.065708603
GCGTGTCGTTTTAGTGAGGGGAAAAAGAAGAGGTAACTGGTACTTGTGGTGGAGCACGACACTGCTAA
GCAAGTGGATGCTATCAGCCTGACGTAAGGGATGACGCACACTAGGCTATATAAGGTTTTGCTATTCA
TTGAAAGCAGTAGTGACTGATTTGTATATA
>MinSyn_1514|Strength:0.065858846

GCGTGTCGTTTTAGTGAGGTCACTATCAGCCCTGAAGATTGATGAAAAGTCAAAAACAAAAATCAATT
ATATCATGAGCTAAGCACATACGTCAGCGCGGTCAGCCACTTGTGTACCTATATAAGGTTTTGCTATT
CATTGAAAGCAGTAGTGACTGATTTGTATATA
>MinSyn_1260|Strength:0.065903815
GCGTGTCGTTTTAGTGAGGTCAGAAGATCAAAGGGCTAATCTTGCCTGCCTTGAAATTTCGGGAAACC
TCCTCGGGTAGGTCTGACGTAAGGGATGACGCACAAGGCTATCAGCCTATATAAGGTTTTGCTATTCA
TTGAAAGCAGTAGTGACTGATTTGTATATA
>MinSyn_1004|Strength:0.066136525
GCGTGTCGTTTTAGTGAGGTGGTGGAGCACGACATGGTACTTGTGTACAGGGCTCACTATGAGCTAAG
CACATACGTCAGCAAAAATGTCAAAGATACACCTCACATGTAGGCCTATATAAGGTTTTGCTATTCAT
TGAAAGCAGTAGTGACTGATTTGTATATA
>MinSyn_1748|Strength:0.066337952
GCGTGTCGTTTTAGTGAGGATGAGCTAAGCACATACGTCAGTTGCCTGAGGTGGCTCCTACCGACGCA
CACCAGCATGTGTTGATCACCAGCTTCTGCACCTCACATGTAGGCTCTATATAAGGTTTTGCTATTCA
TTGAAAGCAGTAGTGACTGATTTGTATATA
>MinSyn_1152|Strength:0.067812143
GCGTGTCGTTTTAGTGAGGTTTTCAACAACGGTAGGTCACGACCCTGACGTAAGGGATGACGCACATT
CAGAAGATCAAAGGGCTAACAGGGCTCACTGCTAGGAGGACCTATATAAGGTTTTGCTATTCATTGAA
AGCAGTAGTGACTGATTTGTATATA
>MinSyn_1675|Strength:0.068105943
GCGTGTCGTTTTAGTGAGGATCGAAAGGACAGTACCATGACGTAAGCCATGACGTCTACGGTAGGTCA
CCAGCCACTTGTGTCTGAGGTGGCTCCTACCACGACCACTACTATATAAGGTTTTGCTATTCATTGAA
AGCAGTAGTGACTGATTTGTATATA
>MinSyn_1395|Strength:0.068111186
GCGTGTCGTTTTAGTGAGGAACCATTATTGCGCCAATTAGGTTGTCTGCACCTCACATGTAGCTGACG
TAAGGGATGACGCACAACCTCTACAAAACTGGTACTTGTCTATATAAGGTTTTGCTATTCATTGAAAG
CAGTAGTGACTGATTTGTATATA
>MinSyn_1810|Strength:0.068286478
GCGTGTCGTTTTAGTGAGGTCCATCAACAAATAATCCAAGTAAGATGTAGGCTATCAGCTTAGCAAGA
CCTCTGTGGGAGCCACCATACAGGGCTCACCAAATATTTCTTGTCTGCACCTCACATGTAGGCTATCA
CTGACGTAAGGGATGACGCACAACCACTATGCCCAATTAGGTTGTCTTATGACCCCCGCCGATGACGC
GGGAGGTACTTGTGTACAGGATGACGTAAGCCATGACGTCTACAATTAGGTTGTCTGCGCTTTGTCAA
AAGCTAAAAAAGATGATGCCTGATCAGAAGATCAAAGGGCTAGCTTAGCAAGACCTCTACAAATTGCG
ATAAAGGAAAGGTGCACCTCTATATAAGGTTTTGCTATTCATTGAAAGCAGTAGTGACTGATTTGTAT
ATA
>MinSyn_1500|Strength:0.068543444
GCGTGTCGTTTTAGTGAGGAACCATTATTGCGGGGCTCACTGCAGCAAGTGGATTGCCCAATTAGATG
AGCTAAGCACATACGTCAGGGCTCACTGCTAGGAGGACCTATATAAGGTTTTGCTATTCATTGAAAGC
AGTAGTGACTGATTTGTATATA
>MinSyn_1750|Strength:0.068597126
GCGTGTCGTTTTAGTGAGGCAAATATTTCTTGTCTTGTGGTGGAGCACGACACAGGGCTCACATGACG
TAAGCCATGACGTCTATGCTAGGAGGACCGATGCTGATCCTATATAAGGTTTTGCTATTCATTGAAAG
CAGTAGTGACTGATTTGTATATA
>MinSyn_1394|Strength:0.069087006
GCGTGTCGTTTTAGTGAGGAATTTCGGGAAACCTCCTCGCACGACCACTATGCATGACGTAAGCCATG
ACGTCTATGCACCTCACATGTAGGCTATCAGCTTAGCCTATATAAGGTTTTGCTATTCATTGAAAGCA
GTAGTGACTGATTTGTATATA
>MinSyn_1154|Strength:0.069399162
GCGTGTCGTTTTAGTGAGGTCACTATCAGCGTCACGACCACTAGCAAGTGGATCTACAAAACTGGTAC
TTGTGTACCTGACGTAAGGGATGACGCACATCGCGGCTATATAAGGTTTTGCTATTCATTGAAAGCAG
TAGTGACTGATTTGTATATA
>MinSyn_1031|Strength:0.070789853
GCGTGTCGTTTTAGTGAGGTGAAGCATCTTCCCCAATTAGGTTGTCTGCACATGACGTAAGCCATGAC
GTCTATTGTCTGCACCTCACATGTAGGCTATCACTATATAAGGTTTTGCTATTCATTGAAAGCAGTAG

TGACTGATTTGTATATA
>MinSyn_1018|Strength:0.070991503
GCGTGTCGTTTTAGTGAGGCTGACGTAAGGGATGACGCACATAGGTTGTCTGCACCTCACATGTATTT
TCAACAATAGGTTGTCTGCACCTCACATGTAGGCTATATAAGGTTTTGCTATTCATTGAAAGCAGTAG
TGACTGATTTGTATATA
>MinSyn_1779|Strength:0.07107987
GCGTGTCGTTTTAGTGAGGGCACACCAGCATGTGTTGATCACCAGCTTTGTGTACAGGGCTCACTGCT
AGGATGACGTAAGCCATGACGTCTATGCCTGCCTCTATATAAGGTTTTGCTATTCATTGAAAGCAGTA
GTGACTGATTTGTATATA
>MinSyn_1833|Strength:0.071149981
GCGTGTCGTTTTAGTGAGGATGACGTAAGCCATGACGTCTACTCACTGCTAGGAGGACCGATGCTGAT
AACCACGTCTACAAACTATGCCCAATTAGGTTGTCTGCACCCTGACGTAAGGGATGACGCACACTAGG
AGGACCGATGCTGATCTTGCCTGCAAATATTTCTTGTTTGTCTGCACCTCACATGTATCACTATCAGC
CACTGCTAGCTATATAAGGTTTTGCTATTCATTGAAAGCAGTAGTGACTGATTTGTATATA
>MinSyn_1098|Strength:0.071728758
GCGTGTCGTTTTAGTGAGGATGACGTAAGCCATGACGTCTACAGGGCAGGTGGCTCCTACCCTCACAT
GTAGGCTAAATTTCGGGAAACCTCCTCGGAGGACTATATAAGGTTTTGCTATTCATTGAAAGCAGTAG
TGACTGATTTGTATATA
>MinSyn_1276|Strength:0.071778609
GCGTGTCGTTTTAGTGAGGAACCACGTCTACAAGGAGGACCGATGCTGATCGCTTTGTCAAAAGCTAA
AAAAGATGATGCCGATGCTGATCTTATGACGTAAGCCATGACGTCTACGCGGTAGGTTGAAGCATCTT
CCTAGGTTGTCTGCACCTCACCTGACGTAAGGGATGACGCACAAGTTATGACCCCCGCCGATGACGCG
GGAGCTAGGAGGACCGATGCTGATCTTGTCACTATCAGCACCGATGCTGATCTATATAAGGTTTTGCT
ATTCATTGAAAGCAGTAGTGACTGATTTGTATATA
>MinSyn_1057|Strength:0.07197451
GCGTGTCGTTTTAGTGAGGCTGACGTAAGGGATGACGCACACAAGTCTCTCTGCCGACAGTGGTCCCA
AAACAAAAATGTCAAAGATAACAAAACTGGTACCTATATAAGGTTTTGCTATTCATTGAAAGCAGTAG
TGACTGATTTGTATATA
>MinSyn_1961|Strength:0.072025166
GCGTGTCGTTTTAGTGAGGATGAGCTAAGCACATACGTCAGGGTAGGTCACGACCACTATGCCCAATT
AGGAACCATTATTGCGGACCACTATGCCCAACTATATAAGGTTTTGCTATTCATTGAAAGCAGTAGTG
ACTGATTTGTATATA
>MinSyn_1696|Strength:0.073162552
GCGTGTCGTTTTAGTGAGGAACCACGTCTACAAACTTGTGTACAGGGCTCACTGCTAGGGCTTTGTCA
AAAGCTAAAAAAGATGATGCCCTTGATCTGACGTAAGGGATGACGCACACTTGTGTACATCTCTCTGC
CGACAGTGGTCCCAAAATGCTAGGTGGCTCCTACAGGTTGTCTGCACCATGAGCTAAGCACATACGTC
AGACCTCACATGTAGGCTATCAGCTTAGCAGGAAAAAGAAGAGGTTGTACAGGGCTCACTGTCCATCA
ACAAATAATCCAAGTAAGGGTACTTGTGTACAGGCTATATAAGGTTTTGCTATTCATTGAAAGCAGTA
GTGACTGATTTGTATATA
>MinSyn_1091|Strength:0.074300133
GCGTGTCGTTTTAGTGAGGTCACTATCAGCGCTCAAACCATTATTGCGGTAGGTCACGACCACTATGC
CCGCACACCAGCATGTGTTGATCACCAGCTTCAGCTTAGCAAGACCTCTACTTATGACCCCCGCCGAT
GACGCGGGACTACAAAACTGGTACTTGTGTACAGGCTGACGTAAGGGATGACGCACAGTTGTCTTCCA
TCAACAAATAATCCAAGTAAGATTAGGTTGTCTGCAATGACGTAAGCCATGACGTCTACACGACCACT
ATGGGAAAAAGAAGAGGTATGTAGGCTATCAGCTTAGCAAGACAGCCACTTGTGTCTATCCTATATAA
GGTTTTGCTATTCATTGAAAGCAGTAGTGACTGATTTGTATATA
>MinSyn_1206|Strength:0.074336735
GCGTGTCGTTTTAGTGAGGATCCTTACCGCTATGGGTAAGATTATCTTGCCTGCCTGACGTAAGGGAT
GACGCACAGTGTACAGGGCTCACTGCTCTATATAAGGTTTTGCTATTCATTGAAAGCAGTAGTGACTG
ATTTGTATATA
>MinSyn_1082|Strength:0.074496599
GCGTGTCGTTTTAGTGAGGATGAGCTAAGCACATACGTCAGACCACTATGCCCAATTAGGTTGGAAAA
AGAAGAGGTGTACGTGGGAGCCACCAACTATATAAGGTTTTGCTATTCATTGAAAGCAGTAGTGACTG
ATTTGTATATA

>MinSyn_1494|Strength:0.074816292
GCGTGTCGTTTTAGTGAGGAGCAAGTGGATCCTCTACAAAACTGGTACTCCATCAACAAATAATCCAA
GTAAGCGCGGTAGGTCACGACCACTATGCCTCTCTCTGCCGACAGTGGTCCCAAAACATGTAGGCTAT
CAGCTTAGCAAGACCTCTCAGAAGATCAAAGGGCTACTGCTTTGTCAAAAGCTAAAAAAGATGATGCC
CACTATGCCCAAGTGGGAGCCACCAAAATGACGTAAGCCATGACGTCTACGACCACTATGCCCAATTA
GGTTGCTGACGTAAGGGATGACGCACATCTGCACCTCACATGTCTATATAAGGTTTTGCTATTCATTG
AAAGCAGTAGTGACTGATTTGTATATA
>MinSyn_1362|Strength:0.075070345
GCGTGTCGTTTTAGTGAGGATGAGCTAAGCACATACGTCAGAGGAGGACCGATGCTGATCTTGCATCG
AAAGGACAGTAGGTAGGTCACGACCCTATATAAGGTTTTGCTATTCATTGAAAGCAGTAGTGACTGAT
TTGTATATA
>MinSyn_1240|Strength:0.076126761
GCGTGTCGTTTTAGTGAGGGCTTTGTCAAAAGCTAAAAAAGATGATGCACTGGTAATGACGTAAGCCA
TGACGTCTATAGCAAGACCTCTCTATATAAGGTTTTGCTATTCATTGAAAGCAGTAGTGACTGATTTG
TATATA
>MinSyn_1866|Strength:0.076279371
GCGTGTCGTTTTAGTGAGGAAGATTGATGAAAAGTCAAAAACAAAAATCAATTATTGCACCTCACAAA
AATGTCAAAGATACTCTACAAAACTTGGTGGAGCACGACACCTTCTCTCTGCCGACAGTGGTCCCAAA
TAGGCTATCAGCTTAGCAAGACCTCTGAAGATAAGATAATAATGTTGAAGATAAGACCTCTACAAAAC
TGGTTCCATCAACAAATAATCCAAGTAAGCACGTGGGAGCCACCACACTGACGTAAGGGATGACGCAC
AACCACTATGCCCAATTAGGTTGTCTATGACGTAAGCCATGACGTCTAACTGGTACCTATATAAGGTT
TTGCTATTCATTGAAAGCAGTAGTGACTGATTTGTATATA
>MinSyn_1046|Strength:0.076679021
GCGTGTCGTTTTAGTGAGGATGAGCTAAGCACATACGTCAGTAGGATCGAAAGGACAGTAAAAAAATG
TCAAAGATATAGGAGGACCGATGCTATATAAGGTTTTGCTATTCATTGAAAGCAGTAGTGACTGATTT
GTATATA
>MinSyn_1716|Strength:0.078037269
GCGTGTCGTTTTAGTGAGGATTGCGATAAAGGAAAGGACGACCACTATGCCCAATTAAAAATGTCAAA
GATACGGTAGGTCAAACCATTATTGCGTTAGGTTGTCTGCACCTCACTGACGTAAGGGATGACGCACA
GGAGGACCGATGCTGATCTTGCCTGCCATGACGTAAGCCATGACGTCTAGCTCACTGCATCCTTACCG
CTATGGGTAAGATTTCAGCTTAGCAAGCTATATAAGGTTTTGCTATTCATTGAAAGCAGTAGTGACTG
ATTTGTATATA
>MinSyn_1705|Strength:0.078542802
GCGTGTCGTTTTAGTGAGGGCACACCAGCATGTGTTGATCACCAGCTTTGCCTGCCTTGATGAAAAAT
GTCAAAGATACTCTACAAAACTGGTACTTGTGTACAGGGCTCACTATCAGCACTATGCCAACCATTAT
TGCGCTCACATGTAGTGAAGATAAGATAATAATGTTGAAGATAAGACGACCACTATGCCCAATTAGGT
TGTCTAATTTCGGGAAACCTCCTCGCACATGTAGGCTATCAGCTATGACGTAAGCCATGACGTCTAGG
TTGTCAACCACGTCTACAATCTGCACCTCACATGTAGATGAGCTAAGCACATACGTCAGAAACTGGTA
CTTGTGTACAGGGCTCACCTATATAAGGTTTTGCTATTCATTGAAAGCAGTAGTGACTGATTTGTATA
TA
>MinSyn_1139|Strength:0.078804062
GCGTGTCGTTTTAGTGAGGATTGCGATAAAGGAAAGGGATGCTGATCTTGCCTGCCTTGATGAGCTAA
GCACATACGTCAGTGACTGACGTAAGGGATGACGCACACAAGACCTCTACAAAACTGGTACTCTATAT
AAGGTTTTGCTATTCATTGAAAGCAGTAGTGACTGATTTGTATATA
>MinSyn_1854|Strength:0.078810145
GCGTGTCGTTTTAGTGAGGAGGTGGCTCCTACGTTGTCTGCACCTCATGAGCTAAGCACATACGTCAG
AGCAAGACCTCTACAAAACTATATAAGGTTTTGCTATTCATTGAAAGCAGTAGTGACTGATTTGTATA
TA
>MinSyn_1320|Strength:0.079385401
GCGTGTCGTTTTAGTGAGGATGAGCTAAGCACATACGTCAGTATGCCCAATTAGGTTGTCTGCACCTC
ACAACCATTATTGCGTACTATATAAGGTTTTGCTATTCATTGAAAGCAGTAGTGACTGATTTGTATAT
A
>MinSyn_1027|Strength:0.079454015
GCGTGTCGTTTTAGTGAGGCAGTGGTCCCTCCACATAATGACGTAAGCCATGACGTCTACGATGCTGA

TCTTGCCTGCCTTGATGCTATATAAGGTTTTGCTATTCATTGAAAGCAGTAGTGACTGATTTGTATAT
A
>MinSyn_1589|Strength:0.079969118
GCGTGTCGTTTTAGTGAGGTGAAGCATCTTCCCTTGTGTACAATGACGTAAGCCATGACGTCTACTCT
ACAAAACTGGTACTTGCTATATAAGGTTTTGCTATTCATTGAAAGCAGTAGTGACTGATTTGTATATA
>MinSyn_1228|Strength:0.080072794
GCGTGTCGTTTTAGTGAGGAAAAATGTCAAAGATATGCCCACTGACGTAAGGGATGACGCACAGACCG
ATGCTGATCTTGCCTGCTATATAAGGTTTTGCTATTCATTGAAAGCAGTAGTGACTGATTTGTATATA
>MinSyn_1590|Strength:0.080418382
GCGTGTCGTTTTAGTGAGGCTGACGTAAGGGATGACGCACAAATTTTCAACAATGCACCTCACATCGA
AAGGACAGTATTGATGCTATATAAGGTTTTGCTATTCATTGAAAGCAGTAGTGACTGATTTGTATATA
>MinSyn_1088|Strength:0.080561481
GCGTGTCGTTTTAGTGAGGAGGTGGCTCCTACGTACTTGTGTACAGGGCTCACTGCATGAGCTAAGCA
CATACGTCAGCCTTGCTATATAAGGTTTTGCTATTCATTGAAAGCAGTAGTGACTGATTTGTATATA
>MinSyn_1855|Strength:0.080974068
GCGTGTCGTTTTAGTGAGGAGCAAGTGGATAATTAGGTATGAGCTAAGCACATACGTCAGCCCAATTA
GGTTGTCTGCACCTCTGAAGCATCTTCCAGGTTGTCTGTCTCTCTGCCGACAGTGGTCCCAAATGGTA
CTTGTGTACAGGGCTCAATGACGTAAGCCATGACGTCTAACCACTATGCCCAATTAGGTTGTCTGACG
TAAGGGATGACGCACAGGTACTTGTGTACAGGGCTAAGATTGATGAAAAGTCAAAAACAAAAATCAAT
TATACCGATGCTGAGGTGGCTCCTACTAGGTTGTCTGCACCTCACATGTAGGCTGTGGGAGCCACCAC
ACCTCACCTATATAAGGTTTTGCTATTCATTGAAAGCAGTAGTGACTGATTTGTATATA
>MinSyn_1969|Strength:0.081162687
GCGTGTCGTTTTAGTGAGGCTGACGTAAGGGATGACGCACACCGATGCTGATCTTGCCTAACCATTAT
TGCGAAAACTGGTACTATATAAGGTTTTGCTATTCATTGAAAGCAGTAGTGACTGATTTGTATATA
>MinSyn_1341|Strength:0.081197761
GCGTGTCGTTTTAGTGAGGCAAATATTTCTTGTCGGTAGGTCACGACCACATGACGTAAGCCATGACG
TCTAATCTTGCCTGCTATATAAGGTTTTGCTATTCATTGAAAGCAGTAGTGACTGATTTGTATATA
>MinSyn_1028|Strength:0.081374051
GCGTGTCGTTTTAGTGAGGCAGTGGTCCCTCCACAAACTGGTACTTGTGTACAGGGCTCATTATGACC
CCCGCCGATGACGCGGGATCTGCACCTCACATGTAGGCTATCAGCTGACGTAAGGGATGACGCACAAC
CTCTACAAAACTGGTACTTGTGTAGCTTTGTCAAAAGCTAAAAAAGATGATGCCCAATTAGGTTGTCT
GCAATGACGTAAGCCATGACGTCTAAAGACCTCTACAAAACTGGTACTTGTGAGCAAGTGGATCACCT
CACAAAAAATGTCAAAGATATCAGATCGAAAGGACAGTACGGTAGCTATATAAGGTTTTGCTATTCAT
TGAAAGCAGTAGTGACTGATTTGTATATA
>MinSyn_1278|Strength:0.083840769
GCGTGTCGTTTTAGTGAGGATGACGTAAGCCATGACGTCTAAATTAGGTTATTGCGATAAAGGAAAGG
TGTACAGGGCCTATATAAGGTTTTGCTATTCATTGAAAGCAGTAGTGACTGATTTGTATATA
>MinSyn_1986|Strength:0.084381395
GCGTGTCGTTTTAGTGAGGTGGTGGAGCACGACACCATGAGCTAAGCACATACGTCAGACGACCACTA
TGCCCAATTCTATATAAGGTTTTGCTATTCATTGAAAGCAGTAGTGACTGATTTGTATATA
>MinSyn_1308|Strength:0.085037447
GCGTGTCGTTTTAGTGAGGAGGTGGCTCCTACTGGTACTTGTGTACAGGGCAGCCACTTGTGTGAGGA
AGCAAGTGGATTAGCAAGACCTCTACAAAGATTGATGAAAAGTCAAAAACAAAAATCAATTATCTATG
CCCAATTAGGTTGTCTGCACCTCATGAGCTAAGCACATACGTCAGACGATGGTGGAGCACGACACACT
ATGCCCAATTAGGTTGTCTGCACCCTGACGTAAGGGATGACGCACACACTATGCCCAATTATGACCCC
CGCCGATGACGCGGGACCTCTACAAAACTATATAAGGTTTTGCTATTCATTGAAAGCAGTAGTGACTG
ATTTGTATATA
>MinSyn_1299|Strength:0.086340054
GCGTGTCGTTTTAGTGAGGGTGGGAGCCACCATGTGTACAGGGCTCACTGCTAGGAGCAGTGGTCCCT
CCACGTCTGACGTAAGGGATGACGCACATGCACCTCACATGTAGGCTATATGAGCTAAGCACATACGT
CAGTATTGCGATAAAGGAAAGGACAGGGCTCACTGCCTATATAAGGTTTTGCTATTCATTGAAAGCAG
TAGTGACTGATTTGTATATA
>MinSyn_1006|Strength:0.086969403
GCGTGTCGTTTTAGTGAGGATGACGTAAGCCATGACGTCTATCCTGACGTAAGGGATGACGCACAGTA

CTTGTGTACAGGGCTCACTGCAAGATTGATGAAAAGTCAAAAACAAAAATCAATTATTGCCTTGATGA
AAAAATGTCAAAGATATCTACAAAACTGGTACTTGTGTAGGAAAAAGAAGAGGTCCCAATTAGGTTGT
CCTATATAAGGTTTTGCTATTCATTGAAAGCAGTAGTGACTGATTTGTATATA
>MinSyn_1798|Strength:0.087032941
GCGTGTCGTTTTAGTGAGGATGACGTAAGCCATGACGTCTACTTGCCTGCCTTGATGATTCACTATCA
GCTGCACCTCACATGTAGGTGAAGATAAGATAATAATGTTGAAGATAAGACTTAGCAAGATGAGCTAA
GCACATACGTCAGGTACTTGTGTACAGGGCTCACTGCTAGGTGAAGCATCTTCCGACCGATGCTGATC
TTGCCTGCCTTGATGACTATATAAGGTTTTGCTATTCATTGAAAGCAGTAGTGACTGATTTGTATATA
>MinSyn_1555|Strength:0.087773317
GCGTGTCGTTTTAGTGAGGATGACGTAAGCCATGACGTCTACCTCTACAAAACTGGTACTTGTCTGAC
GTAAGGGATGACGCACATGTCTGCACCTCACATGTAGGTTTTCAACAAGGAGGACCGATGCTGATCTT
GTCCATCAACAAATAATCCAAGTAAGGTGTACAGGCTTTGTCAAAAGCTAAAAAAGATGATGCAGACC
TCTACTCAGAAGATCAAAGGGCTAGAGGACCGATGCTGATCTTGCCAACCATTATTGCGGGTAGGTCA
GGTGGCTCCTACGCTATCAGCTTAGCAAGACCTCTACACTATATAAGGTTTTGCTATTCATTGAAAGC
AGTAGTGACTGATTTGTATATA
>MinSyn_1799|Strength:0.088162903
GCGTGTCGTTTTAGTGAGGTCCATCAACAAATAATCCAAGTAAGGCTAGATGACGTAAGCCATGACGT
CTAGCTATATAAGGTTTTGCTATTCATTGAAAGCAGTAGTGACTGATTTGTATATA
>MinSyn_1422|Strength:0.089188931
GCGTGTCGTTTTAGTGAGGATCCTTACCGCTATGGGTAAGATTCTGCTAGGAGGACCGATCACTATCA
GCAAAACTTATGACCCCCGCCGATGACGCGGGAGTCTGCACCTCAATGAGCTAAGCACATACGTCAGC
TAAGATTGATGAAAAGTCAAAAACAAAAATCAATTATTGGTACTTGTGTATGACGTAAGCCATGACGT
CTATTTGAAGATAAGATAATAATGTTGAAGATAAGATTAGGTTGTCTGCACCTCACCTATATAAGGTT
TTGCTATTCATTGAAAGCAGTAGTGACTGATTTGTATATA
>MinSyn_1936|Strength:0.0908275
GCGTGTCGTTTTAGTGAGGATTGCGATAAAGGAAAGGTGGTACTTATGACGTAAGCCATGACGTCTAG
CTATATAAGGTTTTGCTATTCATTGAAAGCAGTAGTGACTGATTTGTATATA
>MinSyn_1343|Strength:0.09132062
GCGTGTCGTTTTAGTGAGGCTGACGTAAGGGATGACGCACACGCGGTAGGTCACGACCACTATGCCTC
TCTCTGCCGACAGTGGTCCCAAAACTGGTACTTGTGTACAGGGCTCACTGCAGCAAGTGGATGTGTAC
AGGGCTCACTGCTAGGAGGACCGATGAGCTAAGCACATACGTCAGCAAGACCATCCTTACCGCTATGG
GTAAGATTCAAGACCTCTACAAAACTGGTACTAAAAATGTCAAAGATAACCTCTACAAAACTCTATAT
AAGGTTTTGCTATTCATTGAAAGCAGTAGTGACTGATTTGTATATA
>MinSyn_1923|Strength:0.092955556
GCGTGTCGTTTTAGTGAGGAGGTGGCTCCTACTTGCCTGATGAGCTAAGCACATACGTCAGGGGCCTA
TATAAGGTTTTGCTATTCATTGAAAGCAGTAGTGACTGATTTGTATATA
>MinSyn_1508|Strength:0.095582719
GCGTGTCGTTTTAGTGAGGAAAAATGTCAAAGATAGCAAGACCTCTACAAAACTGGTATGAAGATAAG
ATAATAATGTTGAAGATAAGAGGCAGGTGGCTCCTACTATGAGCTAAGCACATACGTCAGATCACAGT
GGTCCCTCCACGATAACCATTATTGCGTTGTGTACAGGGCTATGACGTAAGCCATGACGTCTATGCTG
ATCTTGCCTGCCTTGTGGTGGAGCACGACAGGACCGATCTATATAAGGTTTTGCTATTCATTGAAAGC
AGTAGTGACTGATTTGTATATA
>MinSyn_1819|Strength:0.09604893
GCGTGTCGTTTTAGTGAGGAAAAATGTCAAAGATATCGCGGTAGGTCACGACCAAAGATTGATGAAAA
GTCAAAAACAAAAATCAATTATATTAGGGCTTTGTCAAAAGCTAAAAAAGATGATGCGCTCACTGCTA
GGAGTCACTATCAGCTGCCCAATTCTGACGTAAGGGATGACGCACACATGTAGGCTATGAAGATAAGA
TAATAATGTTGAAGATAAGATGCTAGGAGGACCGATGCTGATCTATGACGTAAGCCATGACGTCTATT
GTCTGCACCTCACATGTAGGCTATCAGCTGGTGGAGCACGACAACTCTATATAAGGTTTTGCTATTCA
TTGAAAGCAGTAGTGACTGATTTGTATATA
>MinSyn_1325|Strength:0.096907364
GCGTGTCGTTTTAGTGAGGATGACGTAAGCCATGACGTCTAGTCTGCACCTCACATGTAGGCACACCA
GCATGTGTTGATCACCAGCTCACATGTAGGCTATCAGCTTACTGACGTAAGGGATGACGCACATAGGT
CACGACCACTATCAGCCACTTGTGTATCTTGCCTGCCTTGATGCTATATAAGGTTTTGCTATTCATTG
AAAGCAGTAGTGACTGATTTGTATATA

>MinSyn_1694|Strength:0.097166886
GCGTGTCGTTTTAGTGAGGTTATGACCCCCGCCGATGACGCGGGAGCCTGCCTTGATGAATCGAAAGG
ACAGTATGATCTTGCGTGGGAGCCACCACACATGTAGGCTATCAGCTTAGCAAGACATGAGCTAAGCA
CATACGTCAGTATCAGCTTAGCAAATGACGTAAGCCATGACGTCTACTGCTATATAAGGTTTTGCTAT
TCATTGAAAGCAGTAGTGACTGATTTGTATATA
>MinSyn_1506|Strength:0.097421955
GCGTGTCGTTTTAGTGAGGAGCAAGTGGATGGACCGATGCTGATCTTGCCTGTTATGACCCCCGCCGA
TGACGCGGGATATGCCCAATTAGGTTGTCAAGATTGATGAAAAGTCAAAAACAAAAATCAATTATTGT
ACAGGGCTCACTGCTAGGAATGACGTAAGCCATGACGTCTAAGGACCGATGCTGATCTTTTCAACAAG
CTTAGCAAGACCTCTACAAAAAATGTCAAAGATAGTTGTCTGCACCTCACATGTAGGCTATCCTGACG
TAAGGGATGACGCACAATCAGCTCACTATCAGCTTCCATCAACAAATAATCCAAGTAAGTAGCAAGAC
CTCTACAAAACTGGTCTATATAAGGTTTTGCTATTCATTGAAAGCAGTAGTGACTGATTTGTATATA
>MinSyn_1119|Strength:0.101316857
GCGTGTCGTTTTAGTGAGGCTGACGTAAGGGATGACGCACAATGCTGATCTTGCCTGCCTTGATGAAT
GAGCTAAGCACATACGTCAGGGGCTCACTGCAACCACGTCTACAACCACTATGCCCAATTAGGTTGTT
GAAGATAAGATAATAATGTTGAAGATAAGAAATCCTTACCGCTATGGGTAAGATTCTAAAAAATGTCA
AAGATACGACCACTATGCCCAATTAGGTTGTCTCAAATATTTCTTGTAGGACCGATGCTGATCTTGCC
TGCCCTATATAAGGTTTTGCTATTCATTGAAAGCAGTAGTGACTGATTTGTATATA
>MinSyn_1777|Strength:0.104911167
GCGTGTCGTTTTAGTGAGGGCACACCAGCATGTGTTGATCACCAGCTTGCACCTCACATGTAGGCTAT
CAGCGCTTTGTCAAAAGCTAAAAAAGATGATGCAGGCTATCAGCTTAGCAAGACAGCCACTTGTGTAC
ATGTAGGCTATCAGCTATGAGCTAAGCACATACGTCAGCTGCTAGGAGGACCGATGCTGATCACTATC
AGCACTATGCCCAATTAGGTTGTCTCTGACGTAAGGGATGACGCACAGATGCTGATCTTGCCTGCCTT
GATGCTATATAAGGTTTTGCTATTCATTGAAAGCAGTAGTGACTGATTTGTATATA
>MinSyn_1533|Strength:0.108015405
GCGTGTCGTTTTAGTGAGGTCTCTCTGCCGACAGTGGTCCCAAAAGGTCACATGAGCTAAGCACATAC
GTCAGACCTCTACAAAACTGGTACTTGTGTACAGCTGACGTAAGGGATGACGCACAGATCTTGCCTGC
CTTGATGACTATATAAGGTTTTGCTATTCATTGAAAGCAGTAGTGACTGATTTGTATATA
>MinSyn_1131|Strength:0.108118689
GCGTGTCGTTTTAGTGAGGCTGACGTAAGGGATGACGCACAGATCTTGCCTGCCTTGAATGACGTAAG
CCATGACGTCTACACGCTTTGTCAAAAGCTAAAAAAGATGATGCTACAGGGCTCACTGCTAAACCATT
ATTGCGACAAAACTGGTACTTGTGTACAGGGCGTGGGAGCCACCATACTTGTGTACATTTTCAACAAG
CAAGACCTCTACAAACTATATAAGGTTTTGCTATTCATTGAAAGCAGTAGTGACTGATTTGTATATA
>MinSyn_1409|Strength:0.10842386
GCGTGTCGTTTTAGTGAGGATGACGTAAGCCATGACGTCTAATGCTGATCTTGCCTGCCTTGATGACA
GCCACTTGTGTTCAGCTTAGCAAGATCAGAAGATCAAAGGGCTACGCGGTAGGTCACGACCACTATGC
CCAATTTCTCTCTGCCGACAGTGGTCCCAAATGATGATATCCATCAACAAATAATCCAAGTAAGCTCA
CATGTAGCTGACGTAAGGGATGACGCACAATGCTAGCAAGTGGATCGGTAGGTCACGACCACTAACCA
TTATTGCGGCAAGACCTCTACAAAACTGGTACTTGTCACTATCAGCTCTATATAAGGTTTTGCTATTC
ATTGAAAGCAGTAGTGACTGATTTGTATATA
>MinSyn_1865|Strength:0.109941317
GCGTGTCGTTTTAGTGAGGTTTTCAACAACTGCACCTCACATGTAGGCTATCAGCTTAGTCTCTCTGC
CGACAGTGGTCCCAAAAGCTTAGCAAAAAATGTCAAAGATAGACCGATGCTGATCTTGCCTGCCTTGC
AGCCACTTGTGTTGCTGAAGGTGGCTCCTACTAATGACGTAAGCCATGACGTCTAGCCTGCCTTGATG
ATGGAAAAAGAAGAGGTGAGAATTTCGGGAAACCTCCTCGGGCTGACGTAAGGGATGACGCACAGACC
GATGCTGATCCTATATAAGGTTTTGCTATTCATTGAAAGCAGTAGTGACTGATTTGTATATA
>MinSyn_1134|Strength:0.110884416
GCGTGTCGTTTTAGTGAGGATGACGTAAGCCATGACGTCTAAGGCTATCAGCTTAGCAAGACCTCTAC
TGACGTAAGGGATGACGCACACTCTACAAAACTGGTACTTGTGTACAGGGCCAGTGGTCCCTCCACGC
TCACTGCTAGGAGGACCGATGTGGTGGAGCACGACAGGTTGTCTGCACCTCACACAAATATTTCTTGT
CAGCTTAGCAAGACCTCTACAAAGTGGGAGCCACCATAGGCTATCAGCTATATAAGGTTTTGCTATTC
ATTGAAAGCAGTAGTGACTGATTTGTATATA
>MinSyn_1763|Strength:0.114324633
GCGTGTCGTTTTAGTGAGGGCTTTGTCAAAAGCTAAAAAAGATGATGCCTACAAAACTGGTACTTGTG

TACAGGGTGAAGATAAGATAATAATGTTGAAGATAAGAATGCCCAATTAGGTTGTCTGCACCCAGCCA
CTTGTGTCCTCTACAAAACTGGTACTTGTGTAATGACGTAAGCCATGACGTCTAGTACTTGTGTACAG
GGCTAGGTGGCTCCTACGCCCAATTAGGTAACCACGTCTACAACACTGCTAGGAGGACCGATGCTGAT
TGGTGGAGCACGACAAATTATGAGCTAAGCACATACGTCAGCCTCTAGTGGGAGCCACCATCGCGGTA
GGTCACGACCACTATGCTATATAAGGTTTTGCTATTCATTGAAAGCAGTAGTGACTGATTTGTATATA
>MinSyn_1390|Strength:0.115923226
GCGTGTCGTTTTAGTGAGGTTTTCAACAAGCACCTCACATGTAGGCTCACTATCAGCCTCTACAAATG
AGCTAAGCACATACGTCAGCCACTATGCCCAAAACCATTATTGCGATCTTGCCTGCCATGACGTAAGC
CATGACGTCTATGGTACTTGTGTACAGGGCTATATAAGGTTTTGCTATTCATTGAAAGCAGTAGTGAC
TGATTTGTATATA
>MinSyn_1166|Strength:0.118586881
GCGTGTCGTTTTAGTGAGGCAGCCACTTGTGTCTACAAAACTGGTACTTGTGTATGAGCTAAGCACAT
ACGTCAGACCACTATGCCCAATTAGGTTGTCTGCACCTCCATCAACAAATAATCCAAGTAAGCACGAC
CACTATGCCCAATCAAATATTTCTTGTTCGCGGTAGGTCACGACGCTTTGTCAAAAGCTAAAAAAGAT
GATGCCCCAATTAGGTTGTCTGCACCTCACGGAAAAAGAAGAGGTTGTCTGCACCTCACATGTAGGCT
ATGACGTAAGCCATGACGTCTATAGGTCACGACCACTATGCCCAATATCCTTACCGCTATGGGTAAGA
TTTTGCCTGAACCACGTCTACAAGACCACTATGCCCAATTAGGTCTATATAAGGTTTTGCTATTCATT
GAAAGCAGTAGTGACTGATTTGTATATA
>MinSyn_1826|Strength:0.120565767
GCGTGTCGTTTTAGTGAGGATTGCGATAAAGGAAAGGCACTGCTAGGAGGACCGATGCTGATCCTGAC
GTAAGGGATGACGCACAGTCTGCACCTCACATGTAGGCTATCAGTGGGAGCCACCAGGTCACGACCAC
TATGCTGAAGCATCTTCCGATCTATGACGTAAGCCATGACGTCTAACTAGGTGGCTCCTACCACTGCT
AGGAGGACCGCTATATAAGGTTTTGCTATTCATTGAAAGCAGTAGTGACTGATTTGTATATA
>MinSyn_1996|Strength:0.121128935
GCGTGTCGTTTTAGTGAGGTTATGACCCCCGCCGATGACGCGGGATAGGCTAACCACGTCTACAAGGC
TATATGACGTAAGCCATGACGTCTACTCTACAAAACTGGTACTTGATCGAAAGGACAGTATAGGCTAT
CAGCTTAGCAAGACCTCTATGGTGGAGCACGACAGACCGATGCTGATCTTGCCTGCCTCAGCCACTTG
TGTAGGGCTCACAACCATTATTGCGTCTGCACCATGAGCTAAGCACATACGTCAGCCTCTACAAAACT
GGTATCCTTACCGCTATGGGTAAGATTTGTACAGGGCTCACTGCTAGGAGGACTATATAAGGTTTTGC
TATTCATTGAAAGCAGTAGTGACTGATTTGTATATA
>MinSyn_1336|Strength:0.121219204
GCGTGTCGTTTTAGTGAGGTCACTATCAGCATGTAGGCTATCAGCTTAGCAGCCACTTGTGTTGGTAA
TGAGCTAAGCACATACGTCAGTACTTGTGTACAGGGCTGGAAAAAGAAGAGGTGGCCTGACGTAAGGG
ATGACGCACACGACCACTATGCCTATATAAGGTTTTGCTATTCATTGAAAGCAGTAGTGACTGATTTG
TATATA
>MinSyn_1118|Strength:0.127615374
GCGTGTCGTTTTAGTGAGGAATTTCGGGAAACCTCCTCGGATGCTGATCTTGCCTGCCTTGAATGAGC
TAAGCACATACGTCAGGCTAGGAGGACCGATTTTTCAACAAGTCACGACCACTATGCCCAAATATTTC
TTGTCACTGCTAGGAGGACCGATGCTCTCTCTGCCGACAGTGGTCCCAAAGCTAGGAGGACCGATGCT
GATCTTGCATGACGTAAGCCATGACGTCTAGTCTGCACCTCACATGTAGGAAAAATGTCAAAGATATG
CCCAATTAGGTCTATATAAGGTTTTGCTATTCATTGAAAGCAGTAGTGACTGATTTGTATATA
>MinSyn_1279|Strength:0.129843222
GCGTGTCGTTTTAGTGAGGGCACACCAGCATGTGTTGATCACCAGCTCTAGGAGGACCGATGCTGGTG
GAGCACGACACAATTTCGGGAAACCTCCTCGTCAGAAAAATGTCAAAGATATAGCAAGACCATGAGCT
AAGCACATACGTCAGTAGGAGGAATCCTTACCGCTATGGGTAAGATTGTAGGTCACGACCAAGGTGGC
TCCTACATGCTGATCTTGATGACGTAAGCCATGACGTCTATGTACAGGGCTCCTATATAAGGTTTTGC
TATTCATTGAAAGCAGTAGTGACTGATTTGTATATA
>MinSyn_1671|Strength:0.131230415
GCGTGTCGTTTTAGTGAGGAAGATTGATGAAAAGTCAAAAACAAAAATCAATTATATCTTGCCTATGA
GCTAAGCACATACGTCAGCGCGGTAGGTCACGGCACACCAGCATGTGTTGATCACCAGCTGGTTGTCT
GCACCTCACATGTAGGCAGCCACTTGTGTGATCTTGCCTGCCTTGATGATGACGTAAGCCATGACGTC
TAACCTGTGGGAGCCACCAGCGGTAGGTCACGACCCTATATAAGGTTTTGCTATTCATTGAAAGCAGT
AGTGACTGATTTGTATATA
>MinSyn_1045|Strength:0.131618431

GCGTGTCGTTTTAGTGAGGCTGACGTAAGGGATGACGCACAGGTAGGTCACGACCACTATGCCCAATA
TGACGTAAGCCATGACGTCTACCGAGCTTTGTCAAAAGCTAAAAAAGATGATGCTGATCTCTCTGCCG
ACAGTGGTCCCAAAGACCACTATGCCCAATTAGGTTGTCTGCACAACCACGTCTACAAGGTCACGACC
TATATAAGGTTTTGCTATTCATTGAAAGCAGTAGTGACTGATTTGTATATA
>MinSyn_1700|Strength:0.131772013
GCGTGTCGTTTTAGTGAGGATGACGTAAGCCATGACGTCTATACAGGGCTCACTGCTAGGAGGAAAAA
GAAGAGGTAAGACCTCTACAAAACTGGTACTTAAGATTGATGAAAAGTCAAAAACAAAAATCAATTAT
AACTGGTACTTGTGTACAGGGCTCACTTTATGACCCCCGCCGATGACGCGGGACACTATGCCCAATTA
GGTTGTCTGCCTGACGTAAGGGATGACGCACACGATGCTGATCTTGCTCCATCAACAAATAATCCAAG
TAAGCTCACATGTAGGCTACTATATAAGGTTTTGCTATTCATTGAAAGCAGTAGTGACTGATTTGTAT
ATA
>MinSyn_1693|Strength:0.132226001
GCGTGTCGTTTTAGTGAGGTTTTCAACAATGCCCAATTAGGTTGTCTGCTTTGTCAAAAGCTAAAAAA
GATGATGCTAGCAAGACCTCTACAAAACTGTGGTGGAGCACGACAGCACCTCACATGTAGGCTTCTCT
CTGCCGACAGTGGTCCCAAAGTTGTCTGCACCTCACATGTAGATGAGCTAAGCACATACGTCAGGTAG
GCTATCAGCTTAGCGTGGGAGCCACCAACCTCACATGTAGGCTATCAGCTAACCATTATTGCGCTGGT
ACTTGTGTACAGGGCTCACTGCTACAGTGGTCCCTCCACGCTGACTGACGTAAGGGATGACGCACACA
GGGCTCACTGCTAGGAGGACCTATATAAGGTTTTGCTATTCATTGAAAGCAGTAGTGACTGATTTGTA
TATA
>MinSyn_1353|Strength:0.132596813
GCGTGTCGTTTTAGTGAGGATGACGTAAGCCATGACGTCTATACAAAACTGGTACTTGTGTACAGAAC
CACGTCTACAAATGCTGATCTTGCCTGCATGAGCTAAGCACATACGTCAGATCTGACGTAAGGGATGA
CGCACATATCAGCAAGTGGATGGTAGGTCTATATAAGGTTTTGCTATTCATTGAAAGCAGTAGTGACT
GATTTGTATATA
>MinSyn_1731|Strength:0.136219509
GCGTGTCGTTTTAGTGAGGATGACGTAAGCCATGACGTCTAATTAGAAGATTGATGAAAAGTCAAAAA
CAAAAATCAATTATCCAATTAGGTTAGCAAGTGGATTTGTCTGCACCTCAATCCTTACCGCTATGGGT
AAGATTATCACTATCAGCCCTCACATGTAGGCTATCAGCTTATGAGCTAAGCACATACGTCAGCTTGT
GTACAGGGTGGTGGAGCACGACACTGCTAGGAGCTATATAAGGTTTTGCTATTCATTGAAAGCAGTAG
TGACTGATTTGTATATA
>MinSyn_1461|Strength:0.136978696
GCGTGTCGTTTTAGTGAGGTCTCTCTGCCGACAGTGGTCCCAAAAGGGCTCACTGCTAGGAGGACCGA
TGCTTCCATCAACAAATAATCCAAGTAAGGCTATCAGCTTAGCAAGAATGACGTAAGCCATGACGTCT
AGTAGGTCAGTGGTCCCTCCACAGGTCACGACCACTATGCCCAATTAGGTTAGCAAGTGGATGTCACG
ACCACTATGCCCAATTAGGATGAGCTAAGCACATACGTCAGGCTAGGAGGACCGATCTATATAAGGTT
TTGCTATTCATTGAAAGCAGTAGTGACTGATTTGTATATA
>MinSyn_1753|Strength:0.137154545
GCGTGTCGTTTTAGTGAGGATGAGCTAAGCACATACGTCAGTCAATGACGTAAGCCATGACGTCTAGG
ACCGATGCTGATCAACCACGTCTACAACTCACATGTAGGCTATCACTATATAAGGTTTTGCTATTCAT
TGAAAGCAGTAGTGACTGATTTGTATATA
>MinSyn_1861|Strength:0.138050657
GCGTGTCGTTTTAGTGAGGAGGTGGCTCCTACGACCGATGCTGATCTTGTTATGACCCCCGCCGATGA
CGCGGGATGCCTGCCTTGATGATAATGAGCTAAGCACATACGTCAGGGCTCACTGCTAGGAGGACCGA
TGCTGATATCCTTACCGCTATGGGTAAGATTACGACCTGACGTAAGGGATGACGCACATAGGAGCTAT
ATAAGGTTTTGCTATTCATTGAAAGCAGTAGTGACTGATTTGTATATA
>MinSyn_1196|Strength:0.141176443
GCGTGTCGTTTTAGTGAGGATGACGTAAGCCATGACGTCTACTCTACAAAACTGGTACTTAGCAAGTG
GATACAAAACTGGTACTTGTGTACAGTTATGACCCCCGCCGATGACGCGGGATGCCCAATTAGGTTGT
CTGCACCTTGAAGATAAGATAATAATGTTGAAGATAAGATCACGACCACTATGCAAAAATGTCAAAGA
TACTCACTGCTAGGAGGACCGATGCTGATCTTTCAGAAGATCAAAGGGCTATGTCTGCTGACGTAAGG
GATGACGCACATTGCCTGCCTATTGCGATAAAGGAAAGGGAGGACCGATGCTGATCTTGCCTGCCTTG
ACTATATAAGGTTTTGCTATTCATTGAAAGCAGTAGTGACTGATTTGTATATA
>MinSyn_1687|Strength:0.143993719
GCGTGTCGTTTTAGTGAGGCTGACGTAAGGGATGACGCACACAGGGCTCACTGCTAGGGTGGGAGCCA

CCAAAAACTGGTACTTGTGTACAGGGCTCAATCGAAAGGACAGTAAGGGCTCACTGCTAGGAGGACCC
AGTGGTCCCTCCACTGCTAGGGGAAAAAGAAGAGGTAGACCTCTACAAAACTGGTATCACTATCAGCA
GGTTGTCTGCACCTCACAATCCTTACCGCTATGGGTAAGATTTAGATGACGTAAGCCATGACGTCTAC
ATGTAGGCTTGAAGCATCTTCCGAGGACCGATGCTGATCTTGCCTGCCTTCTATATAAGGTTTTGCTA
TTCATTGAAAGCAGTAGTGACTGATTTGTATATA
>MinSyn_1609|Strength:0.144450289
GCGTGTCGTTTTAGTGAGGATGAGCTAAGCACATACGTCAGGTCACGACATGACGTAAGCCATGACGT
CTACGGTAGGTCACAAAAATGTCAAAGATAGGTTGTCTGCACCTCACATGTAGCTATATAAGGTTTTG
CTATTCATTGAAAGCAGTAGTGACTGATTTGTATATA
>MinSyn_1818|Strength:0.147457228
GCGTGTCGTTTTAGTGAGGTCACTATCAGCGCATGACGTAAGCCATGACGTCTATGGTACGCTTTGTC
AAAAGCTAAAAAAGATGATGCCACCTCTGACGTAAGGGATGACGCACAATCTATATAAGGTTTTGCTA
TTCATTGAAAGCAGTAGTGACTGATTTGTATATA
>MinSyn_1801|Strength:0.147739797
GCGTGTCGTTTTAGTGAGGTGAAGCATCTTCCGGTTGTCTGCACCTCACATGTTGGTGGAGCACGACA
GACCACTATGCCCAATCTGACGTAAGGGATGACGCACAGGTTGTCTGCATTATGACCCCCGCCGATGA
CGCGGGAGGCTCACTGCTAGGAGGACCGATGCATGACGTAAGCCATGACGTCTACTCTATATAAGGTT
TTGCTATTCATTGAAAGCAGTAGTGACTGATTTGTATATA
>MinSyn_1087|Strength:0.150171624
GCGTGTCGTTTTAGTGAGGAGGTGGCTCCTACGGCTCACTGCTTCCATCAACAAATAATCCAAGTAAG
CTATCAGCTTTCTCTCTGCCGACAGTGGTCCCAAAATGAATGACGTAAGCCATGACGTCTACGCGGTA
GGTCTGAAGATAAGATAATAATGTTGAAGATAAGAGCACCTCACATGTAGGCTATCAGAACCACGTCT
ACAATGCCCAATTAGGTTGTCTTTATGACCCCCGCCGATGACGCGGGAGCCTATGAGCTAAGCACATA
CGTCAGGATGCTGATCTTGCCTGCCTCTATATAAGGTTTTGCTATTCATTGAAAGCAGTAGTGACTGA
TTTGTATATA
>MinSyn_1909|Strength:0.16568868
GCGTGTCGTTTTAGTGAGGTTATGACCCCCGCCGATGACGCGGGACCCAATTAGGTTGTCTGCACCTC
TGACGTAAGGGATGACGCACAGAGGACCGATGCTGATCTTGCTCAGAAGATCAAAGGGCTAACAAAAC
TGGTACGTGGGAGCCACCAACGTCTCTCTGCCGACAGTGGTCCCAAATGTAGGCTATCAGCTTAGCAA
GACCTCAAAAATGTCAAAGATACATGTAGGCTATCAGCTTAGCAATGACGTAAGCCATGACGTCTACC
ACTATGCCCAATTAGGCTATATAAGGTTTTGCTATTCATTGAAAGCAGTAGTGACTGATTTGTATATA
>MinSyn_1883|Strength:0.165916443
GCGTGTCGTTTTAGTGAGGCAGCCACTTGTGTCACATGTAGGCTGCTTTGTCAAAAGCTAAAAAAGAT
GATGCGGAGCTGACGTAAGGGATGACGCACAGTACAGGGCTTCCATCAACAAATAATCCAAGTAAGCA
ATTAGGTTGTAGCAAGTGGATGAGGACCGATGCTGATCTTGCCTGCCTTAACCATTATTGCGGGTACT
TAAGATTGATGAAAAGTCAAAAACAAAAATCAATTATTGTAGGCTATCAGCTTAATGACGTAAGCCAT
GACGTCTACAAGACCTCTACAAAACCTATATAAGGTTTTGCTATTCATTGAAAGCAGTAGTGACTGAT
TTGTATATA
>MinSyn_1155|Strength:0.175773502
GCGTGTCGTTTTAGTGAGGATGAGCTAAGCACATACGTCAGTTAGGTTGTCTGCACCTCATCACTATC
AGCGTGAAGCATCTTCCTCGCAGCCACTTGTGTCAAAACTGGTACTTGTGTACAGGGCTCAAATTTCG
GGAAACCTCCTCGTGCCTGCCTTGATGACGTAAGCCATGACGTCTACGATCTATATAAGGTTTTGCTA
TTCATTGAAAGCAGTAGTGACTGATTTGTATATA
>MinSyn_1044|Strength:0.176197104
GCGTGTCGTTTTAGTGAGGTGAAGCATCTTCCACCACTATGCCCAATTAGGTTGTCTGCACATGAGCT
AAGCACATACGTCAGGTCACGACCACTATGCCCGCTTTGTCAAAAGCTAAAAAAGATGATGCACAGGG
CTCACTGCTAGGAGGACCGAATCCTTACCGCTATGGGTAAGATTATGCTGATCTTGCCTGCCTTGATG
ATTCTCTCTGCCGACAGTGGTCCCAAAACAGGTGAAGATAAGATAATAATGTTGAAGATAAGAAATGA
CGTAAGCCATGACGTCTAGCTCACTGCTAGGACTATATAAGGTTTTGCTATTCATTGAAAGCAGTAGT
GACTGATTTGTATATA
>MinSyn_1876|Strength:0.194464414
GCGTGTCGTTTTAGTGAGGCTGACGTAAGGGATGACGCACACCACTATGCCCAATTCAAATATTTCTT
GTGAGATCCTTACCGCTATGGGTAAGATTGTCTGCACCTCACATGTAGGCTATCAGCTCACTATCAGC
AAGACCTCTACAGCCACTTGTGTTGTTTTCAACAATCGCGGTAGGTCACGACCACTATGCCCAATTCA

GAAGATCAAAGGGCTACCTTATTGCGATAAAGGAAAGGTGTACAGGGCTCACTGCTAGGATGACGTAA
GCCATGACGTCTACCGACTATATAAGGTTTTGCTATTCATTGAAAGCAGTAGTGACTGATTTGTATAT
A
>MinSyn_1254|Strength:0.196902009
GCGTGTCGTTTTAGTGAGGCTGACGTAAGGGATGACGCACATCACGACCACTATGCCCAATTATCACT
ATCAGCCTACAAAACTGGTACTTGTGTACAGGGCTCAAAAATGTCAAAGATAACCGATGCTGATCTTG
CCTCAGCCACTTGTGTCTGCACCTCACATGTAGTGGTGGAGCACGACAACCACTATGCCCAATTAGGT
TTCTCTCTGCCGACAGTGGTCCCAAATCGCGGTAGTGAAGCATCTTCCCAAGACCTCTACAAAACTGG
TAGGTGGCTCCTACACAAAACTGATGAGCTAAGCACATACGTCAGTGCCTCTATATAAGGTTTTGCTA
TTCATTGAAAGCAGTAGTGACTGATTTGTATATA

Supplementary Data File 8

Expression levels of MinSyns containing C-CREs in different locations relative to the TATA box.

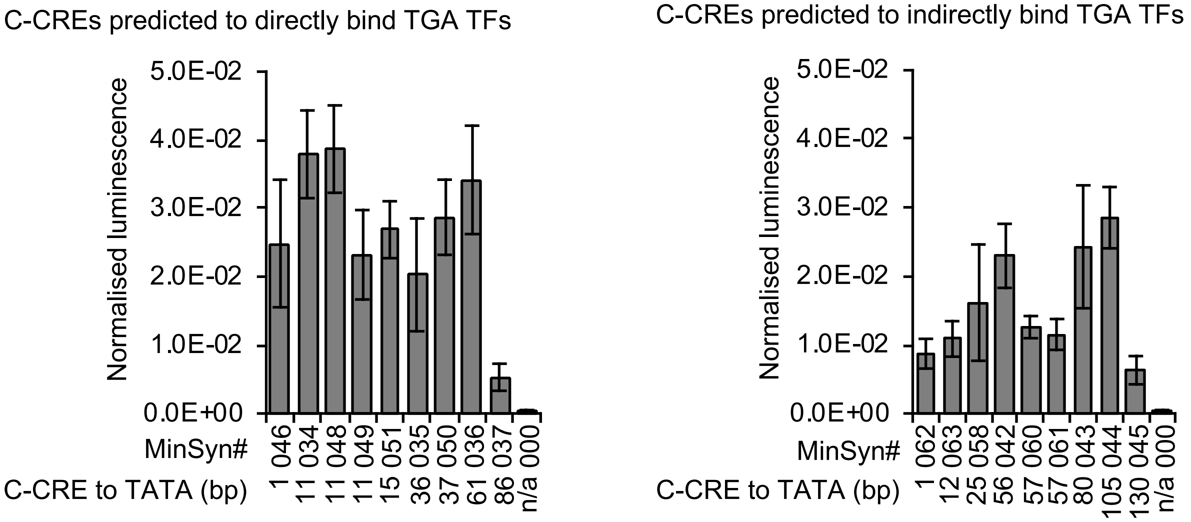

Supplementary Data File 9

Transient expression in mesophyll protoplasts of *Hordeum vulgare* (barley). Both MinSyns and promoters from dicot-infecting viruses show minimal expression compared to the positive control (*Zea mays UBIQUITIN* - *ZmUbi*).

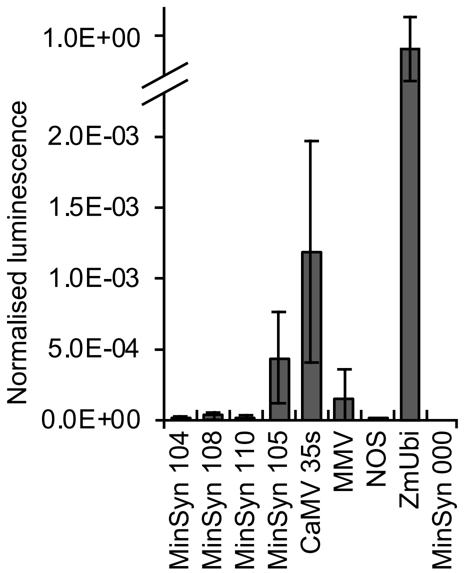

Supplement: gkaa682_Supplemental_File [file gkaa682_supplemental_file.pdf]
